# Systematic Assessment of Exposure Variations on Observed Bioactivity in Zebrafish Chemical Screening

**DOI:** 10.3390/toxics8040087

**Published:** 2020-10-14

**Authors:** Lindsay B. Wilson, Lisa Truong, Michael T. Simonich, Robyn L. Tanguay

**Affiliations:** Department of Environmental and Molecular Toxicology and the Sinnhuber Aquatic Research Laboratory, Oregon State University, Corvallis, OR 97333, USA; wilsonl7@oregonstate.edu (L.B.W.); lisa.truong@oregonstate.edu (L.T.); mtsimonich@oregonstate.edu (M.T.S.)

**Keywords:** zebrafish, high-throughput screening, alternative testing, exposure regimen, bioactivity

## Abstract

The embryonic zebrafish is a powerful tool for high-throughput screening of chemicals. While this model has significant potential for use in safety assessments and chemical prioritization, a lack of exposure protocol harmonized across laboratories has limited full model adoption. To assess the potential that exposure protocols alter chemical bioactivity, we screened a set of eight chemicals and one 2D nanomaterial across four different regimens: (1) the current Tanguay laboratory’s standard protocol of dechorionated embryos and static exposure in darkness; (2) exposure with chorion intact; (3) exposure under a 14 h light: 10 h dark cycle; and (4) exposure with daily chemical renewal. The latter three regimens altered the concentrations, resulting in bioactivity of the test agents compared to that observed with the Tanguay laboratory’s standard regimen, though not directionally the same for each chemical. The results of this study indicate that with the exception for the 2D nanomaterial, the screening design did not change the conclusion regarding chemical bioactivity, just the nominal concentrations producing the observed activity. Since the goal of tier one chemical screening often is to differentiate active from non-active chemicals, researchers could consider the trade-offs regarding cost, labor, and sensitivity in their study design without altering hit rates. Taken further, these results suggest that it is reasonably feasible to reach agreement on a standardized exposure regiment, which will promote data sharing without sacrificing data content.

## 1. Introduction

There are tens of thousands of chemicals available for commerce in the United States, but relatively few have been adequately assessed for health effects [1]. The inability to keep safety information apace with this growing chemical inventory is due, in part, to a lack of reliably predictive structure-activity data. High-throughput bioactivity screening in the whole-animal model is the best available means to generate enough structure-activity data to allow for predicting chemical activity while supporting the goals of the “3 Rs” approach [2,3].

The embryonic zebrafish (*Danio rerio*) is a useful tool for rapid chemical bioactivity screening, capable of producing concentration–response data for hundreds of chemicals in a matter of days [4,5,6,7,8]. Zebrafish embryos develop rapidly and transparently, allowing for visual observation of tissue development in real time. Nearly all organ systems are developed and functioning by five days post fertilization (dpf) [9], and some liver cytochrome P450 enzymes are expressed as early as 72 hpf, indicating phase 1 xenobiotic metabolism [10]. These intrinsic advantages allow us to investigate how chemical exposures might interfere with complex and interacting tissues, an enormous advantage over traditional *in vitro* screening platforms.

The zebrafish model is used extensively to discover novel pharmaceuticals [11], to investigate mechanisms of chemical action [12,13,14,15], and for human disease model research [16,17,18], mostly by leveraging its amenability to high-throughput chemical bioactivity screening. However, a lack of harmonized methods within the field has limited data sharing and adoption of the model to its full potential. Several approaches are common among laboratories which perform zebrafish early life stage bioactivity screening, including exposure in multi-well plates beginning before 8 hpf until 96 or 120 h post fertilization (hpf). The Organisation for Economic Cooperation and Development (OECD) Test No. 236: Fish Embryo Acute Toxicity (FET) test provides guidelines for toxicity testing using early life stage zebrafish [19]. While these guidelines provide helpful information regarding consideration of exposure conditions, the methodology has not been optimized for sensitivity of detection of bioactive compounds in a high-throughput system. Therefore, some aspects of exposure still differ substantially across laboratories that utilize early life stage zebrafish chemical screening for this purpose. A National Toxicology Program initiative, the Systematic Evaluation of the Application of Zebrafish in Toxicology (SEAZIT), recently assessed testing protocols of such laboratories [20,21]. The initiative identified several different exposure conditions that could potentially influence chemical bioactivity, namely the presence or absence of the chorion, light condition, and the specific exposure technique [20,21].

In this study, we used a systematic approach to vary three exposure conditions across a diverse suite of test agents to determine if the bioactivity response profile would vary. With only one exception, each test agent exhibited bioactivity concordantly across the conditions; however, each condition did affect the concentration–response relationship, as determined by the concentration estimated to induce abnormal morphology in 50% of the animals (EC_50_). While some chemical–condition combinations decreased the EC_50_, others increased the EC_50_ when compared to the Tanguay laboratory’s standard exposure regimen.

## 2. Materials and Methods

### 2.1. Test Agent Selection

High-throughput screening should be sensitive enough to detect the bioactivity of compounds across diverse chemical classes and with a range of physiochemical properties. For the soundness of this study, it was important to select test agents that represent this variety. We selected test materials to cover diverse chemical categories and a range of known physiochemical properties, including photolability and octanol–water coefficients (log K_OW_). Table 1 details compound category, CAS registry numbers, supplier information, purity, molecular weight, and log K_OW_.

### 2.2. Zebrafish Husbandry and Embryo Collection

Specific pathogen-free wild type 5D zebrafish (*Danio rerio*) [22] were reared at Sinnhuber Aquatic Research Laboratory (SARL), Corvallis, Oregon in accordance with Institutional Animal Care and Use Committee protocols at Oregon State University [23]. Fish were housed in 50- or 100-gallon brood stock tanks on a recirculating water system under a light cycle of 14 h light: 10 h dark. Water temperature was maintained at 28 ± 1 °C and supplemented with Instant Ocean salts (Spectrum Brands, Blacksburg, VA, USA) and sodium bicarbonate as needed to maintain pH 7.4. Fish were fed Gemma Micro (Skretting, Inc., Fontaine Les Vervins, France) at all life stages twice daily. All experiments were approved by the Oregon State University Animal Care and Use Committee, ACUP# 5113 Approval 11 October 2018.

Embryos were collected using an internal embryo collection apparatus, sorted by similar developmental stage [9] and kept in E2 embryo medium (EM) consisting of 15 mM NaCl, 0.5 mM KCl, 1 mM CaCl_2_, 1 mM MgSO_4_, 0.15 mM KH_2_PO_4_, 0.05 mM Na_2_HPO_4_, and 0.7 mM NaHCO_3_ buffered with 1 M NaOH to pH 7.2 [24]. Embryos were held in a 28 ± 1 °C incubator until dechorionation.

### 2.3. Embryo Dechorionation and Plating

Embryos were enzymatically dechorionated at 4 hpf [25]. Briefly, embryos were placed in glass petri dish bottoms containing 25 mL of EM with 50 µL of 50 mg/mL pronase (#81748; Fluka, St. Louis, MO, USA). Dishes were placed on a modified shaker with constant agitation for 6.5 min followed by several rinses with EM. Dechorionated embryos were examined for pronase or mechanical damage under a dissecting microscope, then robotically loaded into 96-well plates as described in Mandrell, et al. 2012 [25]. Plates were prefilled with 100 µL EM for chemical exposures or 90 µL EM for multi-walled carbon nanotubes (MWCNT) exposures using a 96-well Rainin Liquidator (Mettler-Toledo Rainin, Oakland, CA, USA).

### 2.4. Chemical Exposure

Chemicals were dispensed into 96-well plates pre-loaded with embryos and EM using an HP D300 or D300e Digital Dispenser. For each chemical, embryos were exposed to initial range-finding concentrations of 0, 1, 2.54, 6.45, 16.4, 35, 74.8, and 100 µM (1 plate, *n* = 12 for each concentration). Exposure solutions were normalized to 1% by volume dimethyl sulfoxide (DMSO).

Immediately after chemical addition, plates were heat sealed using an Eppendorf 5390 heat sealer, with pressure-sensitive silicone adhesive backed polyolefin plastic PCR film (Thermaseal RTS) and incubated at 28 ± 1 °C overnight on an orbital shaker at 235 RPM [7]. At 24 and 120 hpf, embryos were screened for morphological effect endpoints (described in Section 2.6) providing the response profiles necessary for selecting the concentration range for definitive testing to cover the range of biological effects from 0% to 100%. When 100% bioactivity was not achieved by the range-finding concentrations, definitive testing concentrations were capped at 100 µM (Table 2). Embryos were exposed in 3 replicate plates for *n* = 36 for each concentration of each chemical.

### 2.5. MWCNT Exposure

Because multi-walled carbon nanotubes (MWCNT) aggregate and cannot be accurately dispensed in the HP D300 or D300e Digital Dispenser, MWCNT master stock solutions were made in EM at 1000 µg/mL. Agglomeration/aggregation of MWCNTs before and after sonication was visually compared between MWCNTs suspended in EM and in ultrapure water. No difference was observed between the two media, so EM was selected as the media for MWCNT exposures to improve background survival of the zebrafish during exposure. To reduce material agglomeration, stock solutions were sonicated using a Fisher Scientific 60 Sonic Dismembrator for 6 min at 14 °C and stored briefly at 4 °C until just before exposure. Immediately before exposure, solutions were again sonicated for 6 min and simple dilutions were made by adding MWCNT solution to EM to achieve concentrations 10× above the test exposure concentrations. A total of 10 µL of the diluted stock was dispensed into each appropriate well of a 96-well plate pre-loaded with 90 µL EM and an embryo as described in Section 2.3.

Embryos were exposed to MWCNTs at initial range-finding concentrations of 0, 10, 23.2, 50, 75, and 100 µg/mL (*n* = 16 for each concentration). Definitive testing concentrations were chosen as in Section 2.4. When 100% bioactivity was not achieved by the range-finding concentrations, definitive testing concentrations did not exceed 100 µg/mL (Table 2). Immediately after solutions were dispensed, plates were sealed, stored, and screened as described above. Embryos were exposed in 3 replicate plates for an *n* = 48 for each concentration.

### 2.6. Standard Exposure Regimen

Under the Tanguay laboratory’s standard exposure regimen, embryos were enzymatically dechorionated on an automated platform at 4 hpf, with static exposure to the test chemical beginning at 6–8 hpf. Plates were kept in the dark at 28 ± 1 °C with gentle shaking until 24 hpf, when embryonic photomotor response (EPR) was assessed and embryos were screened for mortality and 4 morphology endpoints. Plates were then returned to the dark incubator until 120 hpf when larval photomotor response (LPR) was assessed and embryos were screened for additional mortality and incidence of abnormality in 18 morphology endpoints (Table 3).

### 2.7. Photomotor Responses

The embryonic photomotor response (EPR) assay was conducted at 24 hpf, taking care to not expose the test plates to visible light prior to the assay [26]. Briefly, EPR videos were captured only with infrared lighting, while the stimulus consisted of two 1 s pulses of white visible light at 30 and 40 s after video recording began. The nine seconds prior to the first pulse were considered the “background” (B) period; the nine seconds immediately after the first pulse were considered the “excitatory” (E) period; the nine seconds following the second pulse were considered the “refractory” (R) period. During each period, test embryos may exhibit normal or hypo- or hyper-activity relative to the on-plate control animals, indicating chemical-induced effects on non-visual (eyes are not fully developed by 24 hpf) photomotor development [27].

The larval photomotor response (LPR) assay was conducted at 120 hpf when the 96-well plates of larvae were placed into ZebraBox behavioral analysis chambers (Viewpoint Life Sciences) and larval movement tracked with ZebraLab motion analysis software for 18 min across 3 cycles of 3 min light: 3 min dark. The distance moved by each larva was integrated over 6 s binning periods, averaged for the test concentration, and area under the curve was calculated and compared to the on-plate control group as previously described [28].

It should be noted that while both the EPR and LPR assays assess photomotor response, EPR is strictly based on the response of photoreceptors within the hindbrain prior to eye development while LPR, on the other hand, is driven by visual stimulus. While both responses represent a behavioral effect, the mechanism leading to each effect can differ for a single compound. Additionally, the EPR assay takes place at 24 hpf, which may be before the affected system/tissue is developed enough for the chemical to have the effect(s) observed at 5 dpf; therefore, a response in either assay is considered a “hit” independently of the other.

For all assessments, data were uploaded under a unique well-plate barcode into a custom LIMS, Zebrafish Acquisition and Analysis Program (ZAAP), database and analyzed using custom R scripts that were executed in the LIMS background [7].

### 2.8. Experiment Overview

**Figure 1:**
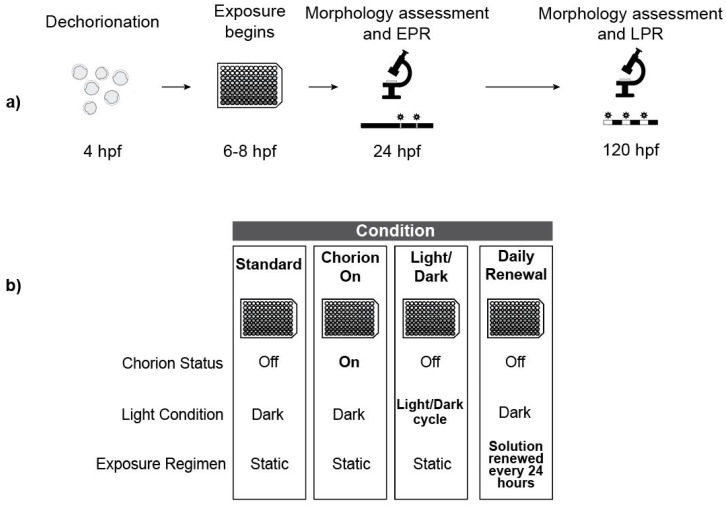
Experimental Design. (**a**) Overview of the Tanguay laboratory’s standard exposure regimen (hpf = hours post fertilization); (**b**) Overview of treatments highlighting exposure conditions that vary from the standard regimen.

Embryos were exposed to each test agent under 4 different conditions: standard exposure regimen (described above), chorion-on, light/dark cycle incubation, and daily solution renewals (Figure 1). Embryos were exposed in three replicate 96-well plates for each chemical under each condition for a total *n* = 36 (chemical exposures) or 48 (MWCNT exposures) for each concentration.

The chorion-on condition was exactly as described for the standard exposure regimen with the omission of the chorion removal process.

For the light/dark incubation treatment, embryos were incubated under a 14-h light: 10-h dark schedule for the entire exposure period with light exposure from bulbs characterized by the following specifications: 34 Watt, 2700 Lumen, Color Rendering Index 62, Color Temperature 4200 K. Light intensity was measured at 120 ± 10 lux. The remainder of the exposure proceeded exactly like the standard exposure regimen.

For the daily renewal treatment, we replenished exposure solutions every 24 h (see Section 2.9). The remainder of the exposure proceeded exactly like the standard exposure regimen.

### 2.9. Daily Solution Renewals

Exposure solutions were renewed at 24, 48, 72, and 96 hpf using a series of repeated 1:1 dilutions with EM using a 96-well Rainin Liquidator. A total of 100 µL of fresh medium was loaded into the wells in addition to the original exposure solution. Then, 100 µL of the diluted solution was removed and discarded, taking care to not disturb the animals. This was repeated 6 times for a total of 7 dilutions to reach a concentration < 1% of the original exposure solution. For MWCNT exposures, 10 dilutions were performed to help disperse agglomerated particles and 110 µL was removed during the tenth dilution for a final volume of 90 µL to accommodate 10 µL of the 10× replenishing solution. Chemical or MWCNT replenishing solutions were dispensed into the wells and plates were freshly sealed and returned to the dark 28 °C incubator. In rare instances of larvae accidentally lost in the renewal procedure, they were omitted from the final analyses.

### 2.10. Data Analysis

To assess effects on morphology, percent incidence of abnormalities for each endpoint was calculated (*n* = 36–48) for every test agent–condition combination. Figure S1 shows concentration–response relationships for all endpoints. The percent incidence of any observed morphological effect was calculated and a logistic regression model was fit to the data and the concentration that resulted in any malformation in 50% of the animals (EC_50_) was estimated using the *drc* package [29]. The EC_50_ for each test agent–condition combination was compared to the EC_50_ for the corresponding standard condition. Fold change was calculated between the standard condition and varied condition for each test agent and any fold change below −1.25 or above 1.25 was considered a true effect of the condition based on the observation that background effects up to 20% can be normally observed in dechorionated embryos. In this study, the highest average rate of background effects for any condition was approximately 12.2%, making the selected fold change cut-off sufficiently conservative.

For behavior data, if two consecutive concentrations elicited the same behavioral response, it was considered a hit for a given test agent–condition combination. Statistical significance was calculated from the computed entropy for LPR and the movement index for each interval of the EPR (when available) [28]. EPR was only measured for the standard and chorion-on treatments as embryos exposed under the other conditions were exposed to visible light before the assay, obviating its use. Only hyperactivity was considered a chemical effect in the B phase of EPR and the L phase of LPR, as hypoactivity in these periods is not associated with sufficient dynamic range. Heatmaps were made using the *ggplot2* [30] package in R and figures were formatted in Adobe Illustrator.

## 3. Results and Discussion

### 3.1. Effect of Chorion Status on Bioactivity

In teleosts, the chorion is an acellular, semipermeable membrane surrounding a developing embryo. It provides some degree of mechanical protection from environmental disturbances and facilitates gas and ion exchange. Guidelines within the OECD FET test address the potential for the chorion to serve as a chemical barrier, though it does not make recommendations for mechanical or enzymatic removal of the chorion [19]. For biomedical discovery research, our group has promoted the removal of the chorion to facilitate high-throughput screening for two main reasons. First, we were concerned that the chorion may present a barrier for chemical uptake. We reasoned that if some chemicals or nanomaterials are not able to reach the developing embryo for the first 48 h encompassing the sensitive period of primary organogenesis, screening may result in an unacceptable false negative rate [8]. In zebrafish, the chorion surrounds the embryo for the first 48 hpf [31] and may also serve as a barrier to some chemicals, though its permeability dynamics are not straightforward or well-understood. Recent studies have indicated increased sensitivity to chemicals with the removal of the chorion, including some organophosphate flame retardants and nanomaterials [32,33,34].

**Figure 2:**
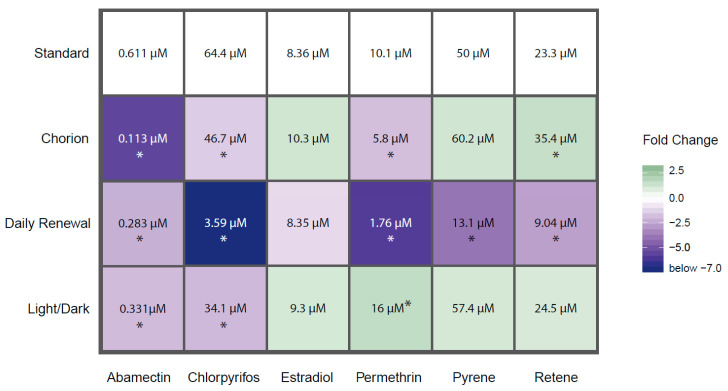
Heatmap of EC_50_ values for each test agent–condition combination that resulted in a calculable EC_50_. Colors refer to fold change between the EC_50_ derived from the varied condition vs. the standard condition. Darker colors indicate greater difference in EC_50_ with blue indicating increased bioactivity and green indicating decreased bioactivity. Values within cells are the EC_50_ for that test agent–condition combination. * = fold change greater than 1.25 or less than −1.25. Chemicals not shown did not produce effects great enough to calculate an EC_50_ under any condition.

**Figure 3:**
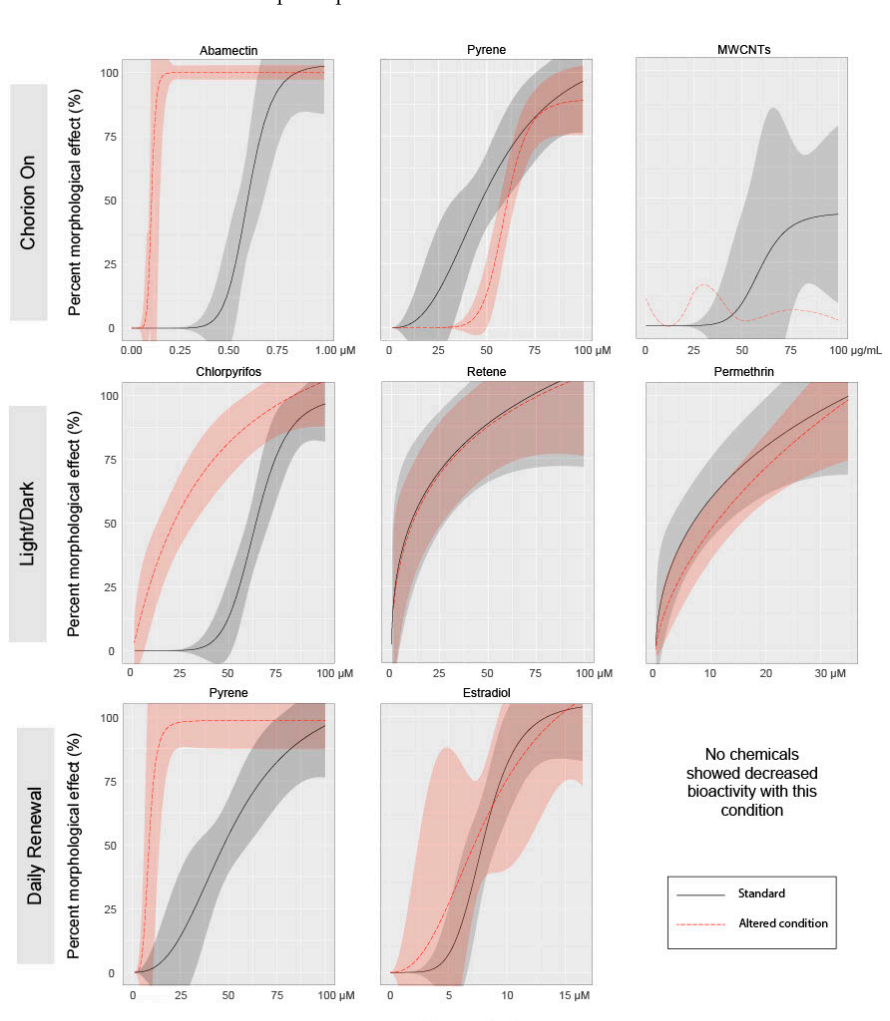
Dose–response curves for select test agents for each condition. Individual figures were selected to display the range of effects on bioactivity of each condition versus the standard regimen. Lines represent mean percent incidence for each concentration fit to a logarithmic regression model. Shaded areas represent 95% confidence intervals for model fit. MWCNT chorion-on exposure did not produce effects great enough to which to fit a logarithmic model. For this instance, a LOESS model was fit to the mean percent incidence values for each concentration.

Our second motivation for advocating the removal of the chorion is to facilitate the measurements of endpoints and to reduce the potential confounding effects of chemical-mediated hatching failure. Non-invasive microscopic imaging of embryonic structures is simply easier when the developing embryo is not encased in a round rolling configuration, as dechorionated embryos generally lay flat and are easily manipulated for consistent orientation for imaging. Additionally, since the EPR assay captures contralateral tail bends, the presence of the chorion may reduce the measurable activity, resulting in a decrease in sensitivity [27]. It is well established by ecotoxicological studies that some chemical exposures affect hatching, and the lack of hatching produces secondary adverse effects that include edemas, body axis curvature, and skeletal deformities. In this study, relative to the Tanguay laboratory’s standard (dechorionated) condition, leaving the chorion intact until natural hatchout increased the incidence of abnormal morphology associated with abamectin, chlorpyrifos, and permethrin exposures, and decreased the incidence of abnormal morphology associated with retene (Figure 2). For each chemical that showed altered responses with an intact chorion, the specific endpoints affected did not change, though the concentration required to elicit a significant response was altered. The exception is mortality, which was significantly reduced for retene and MWCNTs at both time points with intact chorions compared to dechorionated embryos. Figure 3 shows concentration–response curves for select chemical–condition combinations that displayed the range of responses (see Figure S1 for concentration–response for all combinations).

**Figure 4:**
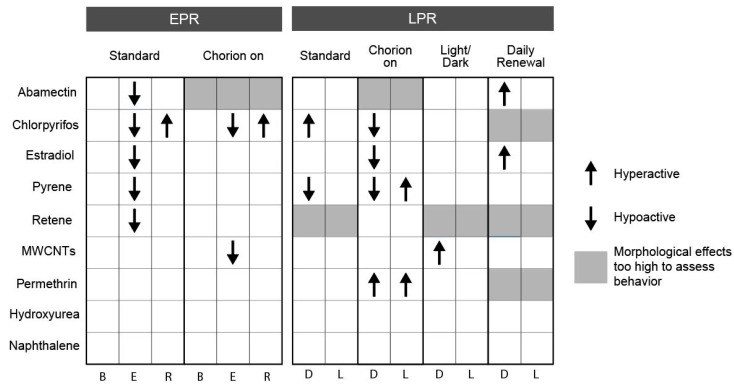
Behavior response associated with exposure to each chemical x condition combination. Combinations with at least two consecutive concentrations associated with significant hyper- or hypo-activity are indicated. Arrows indicate the direction of activity compared to vehicle control for each chemical under each condition. EPR—embryonic photomotor response; LPR—larval photomotor response; B—background; E—excitatory; R—refractory; D—dark; L—light.

An intact chorion influenced early life stage behavior responses at both 24 and 120 hpf. At 24 hpf, the presence of the chorion eliminated E-phase hypoactivity for estradiol, pyrene, and retene and induced hypoactivity for MWCNTs in the E phase (EPR). The observed lack of response with the chorion on further supports the recommendation of conducting bioactivity studies with dechorionated embryos. At 120 hpf, chlorpyrifos and estradiol induced hypoactivity in the D phase, while pyrene induced hyperactivity in the L phase and permethrin induced hyperactivity in both the D and L phases (LPR) with chorionated embryos (Figure 4). Figures S2 and S3 display EPR and LPR responses, respectively, for all test agent–condition combinations.

While an EC_50_ could not be calculated for the MWCNT treatment under any condition, when compared to the standard exposure regime, the presence of the chorion during exposure completely eliminated incidences of abnormal morphology as displayed in Figure 3. Figure S1 displays all endpoints for MWCNTs exposure under each altered condition, showing significant mortality at both 24 and 120 hpf in the standard condition and no significant effects with an intact chorion. The chorion contains pores that allow for water, gas, and ion exchange. By the gastrula stage (5.25–10 hpf [9]) when exposures were initiated in this study, the pores have diameters 0.5–0.7 µM [35], much larger than the chemicals tested, though small enough to prohibit diffusion of large polymers or agglomerated nanomaterials [36,37]. This may explain the decreased bioactivity of multi-walled carbon nanotubes toward chorion-intact embryos.

This study illustrates that chorion status can significantly affect a chemical or nanomaterial’s bioactivity, and that, for traditional small molecules, the effect is more complex than simple size exclusion. The exact mechanism is unknown and likely different for each substance, as indicated by increased bioactivity for some chemicals and decreased bioactivity for others with intact chorions.

**Figure 5:**
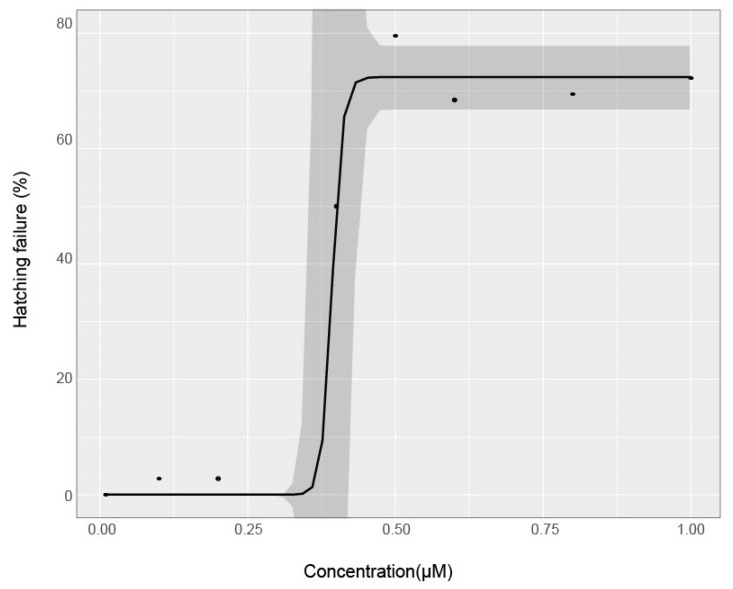
Hatching failure across concentrations for Abamectin chorion-on treatment. Hatching failure was measured at 120 hpf. Points are mean % incidence of hatching failure across replicate plates for each concentration. A logarithmic regression model was fit to the data and shaded areas indicate 95% confidence intervals for model fit.

Some evidence suggests the chorion pores increase in size throughout development, potentially altering chemical uptake at critical developmental periods [38]. Others suggest that the plasma membrane and syncytial layers between the developing embryo and the chorion may play a role in reducing the uptake of chemical agents into embryo tissue [39]. Many chemicals have also been shown to affect hatching ability, including 2,3,7,8-tetrachlorodibenzo-*p*-dioxin (TCDD), benzene [40], trichloroethylene [41], and diethylnitrosamine [42], potentially by weakening or paralyzing the fish, or by prohibiting the production of choriolytic enzymes naturally produced to degrade the chorion. This delay or inhibition of hatching could explain malformations later in development for some compounds. In this study, abamectin altered hatching, with 79.4% of viable larvae still in their chorions at 120 hpf at 1 µM (Figure 5). The hatching rate was measured at 120 hpf by visual observation of live larvae remaining inside an intact chorion. No other test agents induced hatching failure at 120 hpf. Multiple mechanisms of chemical protection by the chorion are likely operant, as might be expected from an evolutionarily costly but successful adaptation. As this study shows, its presence can affect the calculated concentration response profile.

### 3.2. Effect of Light Status on Bioactivity

Some laboratories have expressed concern about the need to conduct chemical exposures under a light:dark (LD) cycle [21] to maintain circadian rhythms during embryonic development. The circadian clock in zebrafish is linked to cell cycle regulation, locomotor activity, and xenobiotic metabolism [43,44,45,46]. In zebrafish, the retina, pineal gland, and many peripheral tissues maintain rhythmic cycles of gene expression and cell proliferation concordant with the LD cycle of their environment. In constant darkness, these tissues maintain their cyclical pattern independently of one another but require light stimulus to synchronize the expression oscillations of these tissues to the environmental light cycle [47,48]. While light exposure is necessary to synchronize circadian rhythms in zebrafish, a constant LD cycle may not be required to maintain them. Carr and Whitmore demonstrated that a single light pulse can trigger the synchronization of cellular clocks for several days [49].

In this study, chemical exposures under a 14 h light: 10 h dark cycle potentiated the incidence of morphological effects associated with abamectin and chlorpyrifos and inhibited the incidence of morphological effects associated with permethrin. The profiles of specific morphological effects for each chemical were not greatly altered under a LD cycle as compared to the standard (dark) exposure condition, though the concentration eliciting a response was altered for some endpoints under this treatment. See Figure S1 for concentration–response data for each individual endpoint. Larval photomotor response was altered under this condition for chlorpyrifos by eliminating hyperactivity in the D phase, for pyrene by eliminating hypoactivity in the D phase, and for MWCNTs by inducing hyperactivity in the D phase (Figure 4).

Chemical photodegradation of labile compounds could be accelerated when using light:dark cycling during screening, which could confound data interpretation. If a chemical rapidly degrades to less toxic intermediates due to co-exposure with light, its bioactivity may be underestimated. Alternatively, if the photo intermediates are more bioactive than the parent compound, its bioactivity may be overestimated. In this study, the incidence of morphological effects was altered for abamectin, chlorpyrifos, and permethrin, all of which are known to be photolabile. Specifically, the EC_50_ for incidence of morphological effects of chlorpyrifos was dramatically lowered from 64.4 µM under dark conditions to 34.1 µM under an LD cycle (Figure 2 and Figure 3). A previous study identified that low concentrations of a metabolite, chlorpyrifos-oxon, but not the parent compound itself, induced developmental toxicity in zebrafish [50]. As we did not measure the chlorpyrifos:chlorpyrifos-oxon ratio in the medium, we cannot be sure that exposure to chlorpyrifos under a LD cycle produced the -oxon form in this study. However, these results indicate that potentiation of bioactivity under a light co-exposure regimen was readily detectable.

### 3.3. Effect of Exposure Regimen on Bioactivity

Exposure techniques such as static, daily renewal, and flow-through exposures each present pros and cons in early life stage chemical bioactivity screening. Static exposures are less labor-intensive, require less test chemicals, and avoid repeated disturbance of the developing organism, a potential but unquantified stressor on development. However, static exposures face limitations from poor chemical solubility, lability, and rapid metabolism, which may each dramatically curtail the intended exposure period. Without a priori knowledge of these chemical limitations, generally the case in high-throughput library screens, static exposures achieve higher throughput and lower cost at the potential expense of fewer bioactivity hits. Daily renewal exposures may reduce throughput, certainly increase costs, but also maximize the likelihood of hit detection.

In this study, daily renewal of the test solution potentiated the incidence of morphological effects associated with abamectin, chlorpyrifos, permethrin, pyrene, and retene. There were no chemicals for which the daily renewal condition reduced the associated bioactivity relative to the standard condition (Figure 2 and Figure 3). For each chemical with potentiated effects, daily chemical renewal increased sensitivity of multiple morphological endpoints, most dramatically craniofacial effects (eye, snout, jaw) and edemas (yolk sac edema, pericardial edema). There were very few differences in the specific endpoints affected by this treatment versus static exposure, though the effective concentration was significantly lowered for the five chemicals listed above. See Figure S1 for all concentration–response data for endpoints. Daily renewal affected larval photomotor response for abamectin by inducing D phase hyperactivity, for estradiol by inducing D phase hyperactivity, and for pyrene by eliminating D phase hypoactivity (Figure 4).

The potential for metabolism and bioaccumulation should be considered when deciding on an exposure regimen. The bioactivity of a chemical that accumulates in the body will likely be potentiated by a renewal regimen while a chemical that is readily metabolized early in the exposure period may no longer be available in the solution to induce effects on later-developing tissues, therefore lowering perceived bioactivity in static exposures. Of the nine test agents, five exhibited greater incidence of morphological effects with chemical renewal. Each of these chemicals has a log K_OW_ value above 4.4 (Table 1), which may indicate potential for bioaccumulation with repeated chemical renewal.

Most chemical screening studies report nominal water concentrations and do not analyze concentrations of parent chemicals or their metabolites in the exposure medium or in the organism’s body. The OECD FET test makes recommendations that exposure regimen may be adapted for volatile, easily degradable, or adsorptive substances and that exposure concentrations should be verified at the beginning and end of the test for static exposures and at the beginning and end of each exposure interval when using a renewal regimen [19]. While this would be the ideal method to ensure consistent concentration throughout the exposure, it is not feasible to collect and analyze these additional samples when conducting high-throughput screening, so physiochemical properties like log K_OW_ may be useful to consider when choosing an exposure regimen.

## 4. Conclusions

This study systematically evaluated the impact of changing exposure conditions on chemical bioactivity by varying the common variables of chorion status, lighting, and chemical renewal. Importantly, we screened the same endpoints in a single laboratory and using the same fish strain, permitting direct data comparisons between experiment designs. Since all three variable conditions altered the concentration-response of the test agents, though not in the same way for each, the results indicate that study design indeed influenced chemical concentration responses. However, in every case except for MWCNTs, each exposure regimen concordantly identified the bioactive compounds, though depending on the chemical, the exposure protocol altered the EC_50_ value, in some cases quite significantly. Therefore, if the goal were to identify active molecules, with the exception of MWCNTs, any of the evaluated protocols in this study would suffice assuming important endpoints are reproducibly measurable and that users of the data understand the EC_50_ values will vary across laboratories. Perhaps predictably, daily chemical renewals altered the EC_50_ to the greatest degree since the total amount of chemical presented to the fish is 400% higher in a 5-day study compared to static non-renewal design. Therefore, for some chemicals, daily renewal would be expected to lead to higher bioaccumulation. However, by utilizing this exposure strategy, researchers may increase the sensitivity of high-throughput screening, by detecting bioactive compounds at lower nominal water concentrations. In some cases, this advantage is offset by the need to use higher quantities of precious or limited chemicals. The chorion represented a perfect barrier to MWCNTs. With chorions removed, MWCNTS are highly toxic, but when left intact, the fish appeared indistinguishable from controls indicating that for nanomaterials, to avoid false negatives, we recommend chorions be removed. Taken further, these results suggest that it is reasonably feasible to reach agreement on a standardized exposure regimen, which will promote data sharing without sacrificing data content. Future refinement of tools capable of predicting the chemical behavior in high-throughput screening zebrafish plates may allow the field to developed assays tailored to chemical characteristics, thus maximizing the reproducibility and translation of zebrafish screening data.

## Figures and Tables

**Table 1:** Test agent information.

Test Material	Category	CAS #	Original Supplier	Purity (%)	MW (g/mol)	Log K_OW_
Abamectin	Pesticide	71751–41–2	Sigma-Aldrich	94.16	873.1	4.400
Chlorpyrifos	Pesticide	2921–88–2	Toronto Research Chemicals	99.57	350.6	4.960
Estradiol	Hormone	50–28–2	Spectrum Chemical Mfg. Corp. *	99.15	272.4	4.010
Hydroxyurea	Pharmaceutical	127–07–1	Sigma-Aldrich	100.0	76.06	−1.800
Naphthalene	Polycyclic aromatic hydrocarbon	91–20–3	Sigma-Aldrich ^†^	99.90	128.2	3.300
Permethrin	Pesticide	52645–53–1	Chem Service, Inc. *	100.0	391.3	6.500
Pyrene	Polycyclic aromatic hydrocarbon	129–00–0	Thermo Fisher *	98.10	202.3	4.880
Retene	Polycyclic aromatic hydrocarbon	483–65–8	Santa Cruz Biotech ^†^	93.00	234.3	6.400
Multi-walled carbon nanotubes < 7 nm (MWCNT)	2D Nanomaterial	99685–96–8	US Research Nanomaterials, Inc.	>97	------	NA

* = Stock solution provided by the National Toxicology Program, NIEHS, Durham, NC, USA. ^†^ = Stock solution provided by the Oregon State University Superfund Research Center Chemical Standards Store.

**Table 2:** Definitive bioactivity testing concentrations for each test agent.

Chemical	Concentrations Tested
Abamectin	0, 0.1, 0.2, 0.4, 0.5, 0.6, 0.8, 1 µM
Chlorpyrifos	0, 2.54, 10, 20, 40, 60, 80, 100 µM
Estradiol	0, 1, 2.54, 5, 7, 9, 12, 16.4 µM
Hydroxyurea	0, 1, 2.54, 6.45, 16.4, 35, 74.8, 100 µM
Naphthalene	0, 1, 2.54, 6.45, 16.4, 35, 74.8, 100 µM
Permethrin	0, 1, 2.54, 5, 8, 12, 16.4, 35 µM
Pyrene	0, 1, 5, 16.5, 30, 50, 65, 100 µM
Retene	0, 1, 5, 20, 30, 45, 65, 100 µM
MWCNT	0, 10, 23.2, 50, 75, 100 µg/mL

**Table 3:** Morphology endpoints assessed at 24 and 120 h post fertilization.

Morphological Endpoints Assessed	
24 hpf	mortality, delayed progression, spontaneous movement, notochord malformations
120 hpf	mortality, yolk sac edema, pericardial edema, bent body axis, touch response, hatching failure (chorion-on condition only), and malformations of the eye, snout, jaw, otic vesicle, brain, somite, pectoral fin, caudal fin, trunk, swim bladder, notochord, pigment, and circulatory system

## Supplementary Materials

The following are available online at https://www.mdpi.com/2305-6304/8/4/87/s1, Table S1: percent incidence for each endpoint from morphology screening; Figure S1: stack plots show concentration–response for each endpoint from morphology screening; Table S2: EPR data for all test agent–condition combinations; Figure S2: plotted EPR response for all test agent–condition combinations; Table S3: LPR data for all test agent–condition combinations; Figure S3: plotted LPR response for all test agent–condition combinations.
