# Supplementary material for: Systematic Assessment of Exposure Variations on Observed Bioactivity in Zebrafish Chemical Screening
Source: Toxics. 2020 Oct 14;8(4):87. doi: 10.3390/toxics8040087 (PMC7712973; doi:10.3390/toxics8040087)
Supplement: Supplementary file 1 [file toxics-08-00087-s001.pdf]

Supplementary Material: Systematic Assessment of Exposure Variations on Observed Bioactivity in Zebrafish Chemical Screening

Lindsay B. Wilson, Lisa Truong, Michael T. Simonich, Robyn L. Tanguay

Table S1. Percent incidence of each endpoint from morphology screening. Table shows data from three replicate plates (n=12 each) for each chemical-condition combination. DR = daily renewal; LD = light/dark cycle. For all chemicals, units are micromolar. For MWCNTs, units are micrograms per milliliter.

Test Agent	Treatment	Replicate plate #	Concentration	Plate Barcode	MO24	DP24	SM24	NC24	MORT	YSE_	AXIS	EYE_	SNOU	JAW_	OTIC	PE__	BRAI	SOMI	PFIN	CFIN	PIG_	CIRC	TRUN	SWIM	NC__	TR__	y.except.MO	any.effect
Abamectin	Chorion	1	0	19231	0	0	0	0	0	0	0	0	0	0	0	0	0	0	0	0	0	0	0	0	0	0	0	0
Abamectin	Chorion	1	0.1	19231	0	0	0	0	0	50	16.67	50	50	50	0	50	0	0	50	0	0	0	0	0	0	16.67	50	50
Abamectin	Chorion	1	0.2	19231	0	0	0	0	0	100	0	100	100	100	0	100	0	0	100	0	0	0	0	0	0	91.67	100	100
Abamectin	Chorion	1	0.4	19231	0	0	0	0	0	100	0	100	100	100	0	100	0	0	100	0	0	0	0	0	0	100	100	100
Abamectin	Chorion	1	0.5	19231	0	0	0	0	0	100	0	100	100	100	0	100	0	0	100	0	0	0	0	0	0	100	100	100
Abamectin	Chorion	1	0.6	19231	0	8.33	0	0	8.33	100	0	100	100	100	0	100	0	0	100	0	0	0	0	0	0	100	100	100
Abamectin	Chorion	1	0.8	19231	0	0	0	0	0	100	0	100	100	100	0	100	0	0	100	0	0	0	0	0	0	100	100	100
Abamectin	Chorion	1	1	19231	0	0	0	0	0	100	0	100	100	100	0	100	0	0	100	0	0	0	0	0	0	100	100	100
Abamectin	Chorion	2	0	19232	0	0	0	0	0	0	0	0	0	0	0	0	0	0	0	0	0	0	0	0	0	0	0	0
Abamectin	Chorion	2	0.1	19232	0	0	0	0	0	8.33	0	8.33	8.33	8.33	0	8.33	0	0	8.33	0	0	0	0	0	0	0	8.33	8.33
Abamectin	Chorion	2	0.2	19232	0	0	0	0	0	75	0	100	100	100	0	100	0	0	75	0	0	0	0	0	0	25	100	100
Abamectin	Chorion	2	0.4	19232	0	0	0	0	0	100	0	100	100	100	0	100	0	0	100	0	0	0	0	0	0	100	100	100
Abamectin	Chorion	2	0.5	19232	8.33	0	0	0	8.33	100	0	100	100	100	0	100	0	0	100	0	0	0	0	0	0	100	100	100
Abamectin	Chorion	2	0.6	19232	0	0	0	0	0	100	0	100	100	100	0	100	0	0	100	0	0	0	0	0	0	100	100	100
Abamectin	Chorion	2	0.8	19232	0	0	0	0	0	100	0	100	100	100	0	100	0	0	100	0	0	0	0	0	0	100	100	100
Abamectin	Chorion	2	1	19232	0	0	0	0	0	100	0	100	100	100	0	100	0	0	100	0	0	0	0	0	0	100	100	100
Abamectin	Chorion	3	0	19233	8.33	0	0	0	8.33	0	0	9.09	0	0	0	0	0	0	0	0	0	0	0	0	0	0	9.09	16.67
Abamectin	Chorion	3	0.1	19233	0	8.33	0	0	8.33	9.09	9.09	9.09	9.09	9.09	0	9.09	0	0	9.09	0	0	0	0	0	0	9.09	16.67	16.67
Abamectin	Chorion	3	0.2	19233	0	0	0	0	0	66.67	0	100	100	100	0	100	0	0	75	0	0	0	0	0	0	33.33	100	100
Abamectin	Chorion	3	0.4	19233	0	0	0	0	0	100	0	100	100	100	0	100	0	0	100	0	0	0	0	0	0	100	100	100
Abamectin	Chorion	3	0.5	19233	0	0	0	0	0	100	0	100	100	100	0	100	0	0	100	0	0	0	0	0	0	100	100	100
Abamectin	Chorion	3	0.6	19233	0	0	0	0	0	100	0	100	100	100	0	100	0	0	100	0	0	0	0	0	0	100	100	100
Abamectin	Chorion	3	0.8	19233	0	0	0	0	0	100	0	100	100	100	0	100	0	0	100	0	0	0	0	0	0	100	100	100
Abamectin	Chorion	3	1	19233	0	0	0	0	0	100	0	100	100	100	0	100	0	0	100	0	0	0	0	0	0	100	100	100
Abamectin	DR	1	0	19215	0	0	0	0	0	0	0	0	0	0	0	0	0	0	0	0	0	0	0	0	0	0	0	0
Abamectin	DR	1	0.1	19215	0	0	0	0	0	0	0	0	0	0	0	0	0	0	0	0	0	0	0	0	0	0	0	0
Abamectin	DR	1	0.2	19215	0	0	0	0	0	0	0	0	0	0	0	0	0	0	0	0	0	0	0	0	0	0	0	0
Abamectin	DR	1	0.4	19215	8.33	0	0	0	8.33	90.91	18.18	100	100	100	0	100	0	0	100	0	0	0	0	0	0	72.73	100	100
Abamectin	DR	1	0.5	19215	0	8.33	0	0	0	100	16.67	100	100	100	0	100	0	0	100	0	0	8.33	0	0	0	91.67	100	100
Abamectin	DR	1	0.6	19215	8.33	9.09	0	0	8.33	90.91	0	100	100	100	0	100	0	0	100	0	0	0	0	0	0	100	100	100
Abamectin	DR	1	0.8	19215	0	8.33	0	0	0	100	16.67	100	100	100	0	100	0	0	100	0	0	0	0	0	0	100	100	100
Abamectin	DR	1	1	19215	0	0	0	0	0	90.91	0	100	100	100	0	100	0	0	100	0	0	0	0	0	0	100	100	100
Abamectin	DR	2	0	19217	0	0	0	0	0	0	0	0	0	0	0	0	0	0	0	0	0	0	0	0	0	0	0	0
Abamectin	DR	2	0.1	19217	0	0	0	0	0	0	0	0	0	0	0	0	0	0	0	0	0	0	0	0	0	0	0	0
Abamectin	DR	2	0.2	19217	0	0	0	0	0	0	0	0	0	0	0	0	0	0	0	0	0	0	0	0	0	0	0	0
Abamectin	DR	2	0.4	19217	0	8.33	0	0	8.33	90.91	9.09	90.91	90.91	90.91	0	90.91	0	0	90.91	0	0	0	0	0	0	54.55	91.67	91.67
Abamectin	DR	2	0.5	19217	0	0	0	0	8.33	100	9.09	100	100	100	0	100	0	0	100	0	0	0	0	0	0	81.82	91.67	100
Abamectin	DR	2	0.6	19217	8.33	9.09	0	0	8.33	100	18.18	100	100	100	0	100	0	0	100	0	0	0	0	0	0	100	100	100
Abamectin	DR	2	0.8	19217	0	8.33	0	0	0	91.67	8.33	100	100	100	0	100	0	8.33	100	0	0	0	8.33	0	0	100	100	100
Abamectin	DR	2	1	19217	0	0	0	0	0	100	9.09	100	100	100	0	100	0	0	100	0	0	0	0	0	0	100	100	100
Abamectin	DR	3	0	19221	0	0	0	0	0	0	0	0	0	0	0	0	0	0	0	0	0	0	0	0	0	0	0	0
Abamectin	DR	3	0.1	19221	0	0	0	0	0	0	0	0	0	0	0	0	0	0	0	0	0	0	0	0	0	0	0	0
Abamectin	DR	3	0.2	19221	8.33	9.09	0	0	8.33	9.09	0	9.09	9.09	9.09	0	9.09	0	0	9.09	0	0	0	9.09	0	0	0	9.09	16.67
Abamectin	DR	3	0.4	19221	0	0	0	0	0	83.33	0	91.67	91.67	91.67	0	91.67	0	0	91.67	0	0	0	0	0	0	41.67	91.67	91.67
Abamectin	DR	3	0.5	19221	0	8.33	0	0	8.33	100	9.09	100	100	100	0	100	0	0	100	0	0	0	0	0	0	100	100	100
Abamectin	DR	3	0.6	19221	0	8.33	0	0	0	100	16.67	100	100	100	0	100	0	0	100	0	0	0	0	0	0	100	100	100
Abamectin	DR	3	0.8	19221	0	8.33	0	0	8.33	100	27.27	100	100	100	0	100	0	0	100	0	0	0	0	0	0	100	91.67	100
Abamectin	DR	3	1	19221	0	9.09	0	0	0	100	18.18	100	100	100	0	100	0	0	100	0	0	0	0	0	0	100	100	100
Abamectin	LD	1	0	19266	0	16.67	0	0	8.33	0	0	0	0	0	0	0	0	0	0	0	0	0	0	0	0	0	16.67	16.67
Abamectin	LD	1	0.1	19266	8.33	0	0	0	8.33	0	0	9.09	9.09	9.09	0	0	9.09	0	0	0	0	0	0	0	0	0	9.09	16.67
Abamectin	LD	1	0.2	19266	16.67	0	0	0	16.67	10	10	10	10	10	0	10	0	0	10	10	0	0	10	0	0	10	10	25
Abamectin	LD	1	0.4	19266	0	0	0	0	41.67	57.14	0	57.14	57.14	57.14	0	57.14	28.57	0	57.14	0	0	0	0	0	0	57.14	33.33	75
Abamectin	LD	1	0.5	19266	0	8.33	0	0	25	77.78	22.22	77.78	77.78	100	0	66.67	22.22	0	77.78	0	0	0	0	0	0	55.56	83.33	100
Abamectin	LD	1	0.6	19266	0	0	0	0	0	100	50	100	100	100	0	100	16.67	0	100	0	0	8.33	0	0	0	91.67	100	100
Abamectin	LD	1	0.8	19266	0	8.33	0	0	0	100	58.33	100	100	100	0	100	8.33	0	100	0	0	0	8.33	0	0	100	100	100
Abamectin	LD	1	1	19266	0	18.18	0	0	0	100	81.82	100	100	100	0	100	9.09	0	100	0	0	0	0	0	0	100	100	100
Abamectin	LD	2	0	19267	0	8.33	0	0	0	0	0	0	0	0	0	8.33	0	0	0	8.33	0	0	8.33	0	0	0	16.67	16.67
Abamectin	LD	2	0.1	19267	25	0	0	0	25	11.11	0	11.11	11.11	11.11	0	11.11	0	0	11.11	0	0	0	0	0	0	11.11	11.11	33.33
Abamectin	LD	2	0.2	19267	0	0	0	0	0	0	0	0	0	0	0	0	0	0	0	0	0	0	0	0	0	0	0	0
Abamectin	LD	2	0.4	19267	8.33	0	0	0	25	11.11	0	11.11	11.11	11.11	0	11.11	0	0	11.11	0	0	0	0	0	0	11.11	9.09	33.33
Abamectin	LD	2	0.5	19267	0	8.33	0	0	33.33	12.5	12.5	12.5	12.5	12.5	0	12.5	12.5	0	12.5	0	0	0	0	0	0	12.5	16.67	41.67
Abamectin	LD	2	0.6	19267	0	0	0	0	8.33	27.27	18.18	36.36	36.36	36.36	0	36.36	27.27	0	27.27	0	0	0	0	0	0	27.27	33.33	41.67
Abamectin	LD	2	0.8	19267	0	8.33	0	0	0	91.67	33.33	91.67	91.67	91.67	0	91.67	41.67	0	91.67	8.33	0	8.33	8.33	0	0	83.33	91.67	91.67
Abamectin	LD	2	1	19267	0	0	0	0	0	100	63.64	100	100	100	0	100	0	0	100	0	0	0	9.09	0	0	81.82	100	100
Abamectin	LD	3	0	19281	0	0	0	0	0	8.33	0	8.33	8.33	8.33	0	8.33	0	0	8.33	0	0	0	0	0	0	0	8.33	8.33
Abamectin	LD	3	0.1	19281	0	0	0	0	0	0	0	0	0	0	0	8.33	0	0	0	0	0	0	0	0	0	0	8.33	8.33
Abamectin	LD	3	0.2	19281	0	8.33	0	0	25	11.11	0	11.11	11.11	11.11	0	11.11	11.11	0	11.11	0	0	0	11.11	0	0	11.11	8.33	33.33
Abamectin	LD	3	0.4	19281	0	16.67	0	0	50	50	16.67	50	50	50	0	50	50	0	50	0	0	0	0	0	0	50	41.67	91.67
Abamectin	LD	3	0.5	19281	8.33	0	0	0	33.33	87.5	50	87.5	87.5	87.5	0	87.5	62.5	0	87.5	0	0	0	0	0	0	87.5	63.64	91.67
Abamectin	LD	3	0.6	19281	0	16.67	0	0	0	100	41.67	100	100	100	0	100	0	0	100	0	0	0	8.33	0	0	75	100	100
Abamectin	LD	3	0.8	19281	0	8.33	0	0	0	100	50	100	100	100	0	100	8.33	0	100	0	0	8.33	0	0	0	100	100	100
Abamectin	LD	3	1	19281	0	9.09	0	0	0	100	54.55	100	90.91	100	0	100	0	0	100	0	0	0	0	0	0	100	100	100
Abamectin	Standard	1	0	19216	0	0	0	0	0	0	0	0	0	0	0	0	0	0	0	0	0	0	0	0	0	0	0	0
Abamectin	Standard	1	0.1	19216	0	0	0	0	0	0	0	0	0	0	0	0	0	0	0	0	0	0	0	0	0	0	0	0
Abamectin	Standard	1	0.2	19216	0	0	0	0	0	8.33	0	8.33	8.33	8.33	0	8.33	0	0	8.33	0	0	0	0	0	0	8.33	8.33	8.33
Abamectin	Standard	1	0.4	19216	0	8.33	0	0	8.33	9.09	9.09	9.09	9.09	9.09	0	9.09	0	0	9.09	0	0	0	9.09	0	0	9.09	8.33	16.67
Abamectin	Standard	1	0.5	19216	8.33	0	0	0	8.33	0	0	0	0	0	0	0	0	0	0	0	0	0	0	0	0	0	0	8.33
Abamectin	Standard	1	0.6	19216	0	0	0	0	0	58.33	8.33	58.33	58.33	75	0	58.33	0	0	58.33	0	0	0	0	0	0	25	75	75

Test Agent	Treatment	Replicate plate #	Concentration	Plate Barcode	MO24	DP24	SM24	NC24	MORT	YSE_	AXIS	EYE_	SNOU	JAW_	OTIC	PE__	BRAI	SOMI	PFIN	CFIN	PIG_	CIRC	TRUN	SWIM	NC__	TR__	.except.M(	any.effect
Abamectin	Standard	1	0.8	19216	8.33	9.09	0	0	8.33	100	18.18	100	100	100	0	100	0	0	100	0	0	0	0	0	0	100	100	100
Abamectin	Standard	1	1	19216	0	0	0	0	0	100	0	100	100	100	0	100	0	0	90.91	0	0	9.09	9.09	0	0	100	100	100
Abamectin	Standard	2	0	19222	8.33	0	0	0	8.33	0	0	0	0	0	0	0	0	0	0	0	0	0	0	0	0	0	0	8.33
Abamectin	Standard	2	0.1	19222	25	0	0	0	25	0	0	0	0	0	0	0	0	0	0	0	0	0	0	0	0	0	0	25
Abamectin	Standard	2	0.2	19222	0	0	0	0	0	16.67	0	16.67	16.67	16.67	0	16.67	0	0	16.67	0	0	0	0	0	0	16.67	16.67	16.67
Abamectin	Standard	2	0.4	19222	8.33	0	0	0	8.33	0	0	0	0	0	0	0	0	0	0	0	0	0	0	0	0	0	0	8.33
Abamectin	Standard	2	0.5	19222	0	0	0	0	0	0	0	0	0	8.33	0	0	0	0	0	0	0	0	8.33	0	0	0	16.67	16.67
Abamectin	Standard	2	0.6	19222	0	0	0	0	0	33.33	0	41.67	41.67	41.67	0	41.67	0	0	33.33	0	0	0	0	0	0	25	41.67	41.67
Abamectin	Standard	2	0.8	19222	0	0	0	0	8.33	100	36.36	100	100	100	0	100	0	0	100	0	0	0	0	0	0	100	91.67	100
Abamectin	Standard	2	1	19222	0	0	0	0	0	100	18.18	100	100	100	0	100	0	0	100	0	0	0	0	0	0	100	100	100
Abamectin	Standard	3	0	19223	0	0	0	0	0	0	0	0	0	0	0	0	0	0	0	0	0	0	0	0	0	0	0	0
Abamectin	Standard	3	0.1	19223	0	0	0	0	0	0	0	0	0	0	0	0	0	0	0	0	0	0	0	0	0	0	0	0
Abamectin	Standard	3	0.2	19223	0	0	0	0	0	8.33	0	8.33	8.33	8.33	0	8.33	0	0	8.33	0	0	0	0	0	0	8.33	8.33	8.33
Abamectin	Standard	3	0.4	19223	8.33	0	0	0	8.33	0	0	0	9.09	9.09	0	0	0	0	0	0	0	0	0	0	0	0	9.09	16.67
Abamectin	Standard	3	0.5	19223	0	0	0	0	0	0	0	0	0	0	0	0	0	0	0	0	0	0	8.33	0	0	0	8.33	8.33
Abamectin	Standard	3	0.6	19223	8.33	0	0	0	8.33	18.18	0	18.18	18.18	18.18	0	18.18	0	0	18.18	0	0	0	0	0	0	18.18	18.18	25
Abamectin	Standard	3	0.8	19223	0	8.33	0	0	0	91.67	25	91.67	100	100	0	100	0	0	91.67	0	0	0	0	0	0	58.33	100	100
Abamectin	Standard	3	1	19223	18.18	0	0	0	18.18	100	0	100	100	100	0	100	0	0	100	0	0	0	0	0	0	100	100	100
Chlorpyrifos	Chorion	1	0	18739	0	0	0	0	0	0	0	0	0	0	0	0	0	0	0	0	0	0	0	0	0	0	0	0
Chlorpyrifos	Chorion	1	2.54	18739	0	0	0	0	0	8.33	8.33	8.33	8.33	8.33	8.33	8.33	8.33	8.33	8.33	8.33	0	0	8.33	8.33	0	8.33	8.33	8.33
Chlorpyrifos	Chorion	1	10	18739	0	0	0	0	0	0	0	0	0	0	0	0	0	0	0	0	0	0	0	0	0	0	0	0
Chlorpyrifos	Chorion	1	20	18739	0	0	0	0	0	0	0	0	0	0	0	0	0	0	0	0	0	0	8.33	0	0	0	8.33	8.33
Chlorpyrifos	Chorion	1	40	18739	0	0	0	0	0	8.33	8.33	0	0	0	0	0	0	0	0	25	0	0	33.33	0	0	0	33.33	33.33
Chlorpyrifos	Chorion	1	60	18739	0	0	0	0	16.67	30	50	10	20	30	0	30	0	0	20	30	0	0	40	0	0	0	50	66.67
Chlorpyrifos	Chorion	1	80	18739	0	0	0	0	58.33	100	100	60	80	100	40	80	20	0	80	100	0	0	100	20	0	20	41.67	100
Chlorpyrifos	Chorion	1	100	18739	0	0	0	0	83.33	100	100	100	100	100	50	100	100	0	100	100	0	0	100	100	0	100	16.67	100
Chlorpyrifos	Chorion	2	0	18740	0	0	0	0	0	0	0	0	0	0	0	0	0	0	0	0	0	0	0	0	0	0	0	0
Chlorpyrifos	Chorion	2	2.54	18740	0	0	0	0	0	0	0	0	0	0	0	0	0	0	0	0	0	0	0	0	0	0	0	0
Chlorpyrifos	Chorion	2	10	18740	0	0	0	0	0	0	0	0	0	0	0	0	0	0	0	0	0	0	0	0	0	0	0	0
Chlorpyrifos	Chorion	2	20	18740	0	0	0	0	0	0	0	0	0	0	0	0	0	0	0	0	0	0	0	0	0	0	0	0
Chlorpyrifos	Chorion	2	40	18740	0	0	0	0	8.33	0	9.09	0	0	0	0	0	0	0	0	9.09	0	0	18.18	0	0	0	16.67	25
Chlorpyrifos	Chorion	2	60	18740	0	0	0	0	25	66.67	77.78	22.22	44.44	44.44	0	44.44	11.11	0	22.22	44.44	0	0	77.78	0	0	11.11	58.33	83.33
Chlorpyrifos	Chorion	2	80	18740	0	0	0	0	83.33	100	100	0	50	50	0	100	0	0	50	100	0	0	100	50	0	50	16.67	100
Chlorpyrifos	Chorion	2	100	18740	0	0	0	0	83.33	100	100	100	100	100	0	100	50	0	100	100	0	0	100	100	0	100	16.67	100
Chlorpyrifos	Chorion	3	0	18741	0	0	0	0	0	0	0	0	0	0	0	0	0	0	0	0	0	0	0	0	0	0	0	0
Chlorpyrifos	Chorion	3	2.54	18741	0	0	0	0	0	0	0	0	0	0	0	0	0	0	0	0	0	0	0	0	0	0	0	0
Chlorpyrifos	Chorion	3	10	18741	0	0	0	0	0	0	0	0	0	0	0	0	0	0	0	0	0	0	0	0	0	0	0	0
Chlorpyrifos	Chorion	3	20	18741	0	0	0	0	0	0	0	0	0	0	0	0	0	0	0	0	0	0	0	0	0	0	0	0
Chlorpyrifos	Chorion	3	40	18741	8.33	0	0	0	8.33	0	9.09	0	0	9.09	0	18.18	0	0	0	9.09	0	0	27.27	0	0	0	36.36	41.67
Chlorpyrifos	Chorion	3	60	18741	0	0	0	0	41.67	42.86	42.86	14.29	14.29	14.29	0	28.57	14.29	0	14.29	57.14	0	0	85.71	14.29	0	14.29	50	91.67
Chlorpyrifos	Chorion	3	80	18741	0	0	0	0	83.33	100	50	0	100	100	0	50	0	0	0	0	0	0	50	0	0	0	16.67	100
Chlorpyrifos	Chorion	3	100	18741	0	0	0	0	58.33	100	100	80	100	100	20	60	20	20	40	20	20	0	100	40	20	40	41.67	100
Chlorpyrifos	DR	1	0	18734	0	0	0	0	0	0	0	0	0	0	0	0	0	0	0	0	0	0	0	0	0	0	0	0
Chlorpyrifos	DR	1	2.54	18734	8.33	0	0	0	16.67	0	20	0	0	0	0	0	0	0	0	40	0	0	30	10	0	0	36.36	50
Chlorpyrifos	DR	1	10	18734	16.67	0	0	0	66.67	50	25	25	25	25	25	25	25	0	25	50	0	0	50	25	0	0	20	83.33
Chlorpyrifos	DR	1	20	18734	8.33	0	0	0	91.67	0	0	0	0	0	0	0	0	0	0	0	0	0	0	0	0	0	0	91.67
Chlorpyrifos	DR	1	40	18734	8.33	9.09	0	0	83.33	50	50	50	50	50	50	50	50	50	50	50	50	0	50	50	0	50	18.18	91.67
Chlorpyrifos	DR	1	60	18734	16.67	10	0	0	91.67	0	0	0	0	0	0	0	0	0	0	0	0	0	0	0	0	0	10	100
Chlorpyrifos	DR	1	80	18734	16.67	30	0	0	91.67	0	0	0	0	0	0	0	0	0	0	0	0	0	0	0	0	0	30	91.67
Chlorpyrifos	DR	1	100	18734	16.67	20	0	0	91.67	0	0	0	0	0	0	0	0	0	0	0	0	0	0	0	0	0	20	91.67
Chlorpyrifos	DR	2	0	18737	0	0	0	0	16.67	0	0	0	0	0	0	0	0	0	0	0	0	0	0	0	0	0	0	16.67
Chlorpyrifos	DR	2	2.54	18737	0	0	0	0	0	8.33	8.33	8.33	8.33	8.33	0	8.33	0	0	8.33	16.67	0	0	16.67	8.33	0	8.33	16.67	16.67
Chlorpyrifos	DR	2	10	18737	8.33	0	0	0	75	100	100	66.67	66.67	100	33.33	100	66.67	33.33	100	66.67	0	0	100	33.33	0	66.67	27.27	100
Chlorpyrifos	DR	2	20	18737	0	8.33	0	0	83.33	50	50	50	50	50	50	50	50	0	50	50	0	0	50	50	0	50	8.33	91.67
Chlorpyrifos	DR	2	40	18737	8.33	18.18	0	0	91.67	0	0	0	0	0	0	0	0	0	0	0	0	0	0	0	0	0	18.18	91.67
Chlorpyrifos	DR	2	60	18737	0	16.67	0	0	91.67	0	0	0	0	0	0	0	0	0	0	0	0	0	0	0	0	0	16.67	91.67
Chlorpyrifos	DR	2	80	18737	0	0	0	0	91.67	0	0	0	0	0	0	0	0	0	0	0	0	0	0	0	0	0	0	91.67
Chlorpyrifos	DR	2	100	18737	0	16.67	0	0	91.67	0	0	0	0	0	0	0	0	0	0	0	0	0	0	0	0	0	16.67	100
Chlorpyrifos	DR	3	0	18776	8.33	0	0	0	8.33	0	0	0	0	0	0	0	0	0	0	0	0	0	0	0	0	0	0	8.33
Chlorpyrifos	DR	3	2.54	18776	0	0	0	0	0	0	8.33	0	0	0	0	0	0	0	0	0	0	0	8.33	0	0	0	8.33	8.33
Chlorpyrifos	DR	3	10	18776	0	8.33	0	0	58.33	100	80	100	100	100	0	100	20	0	80	0	0	0	0	0	0	80	41.67	100
Chlorpyrifos	DR	3	20	18776	0	0	0	0	100	NA	NA	NA	NA	NA	NA	NA	NA	NA	NA	NA	NA	NA	NA	NA	NA	NA	0	100
Chlorpyrifos	DR	3	40	18776	0	25	0	0	100	NA	NA	NA	NA	NA	NA	NA	NA	NA	NA	NA	NA	NA	NA	NA	NA	NA	25	100
Chlorpyrifos	DR	3	60	18776	18.18	33.33	0	0	100	NA	NA	NA	NA	NA	NA	NA	NA	NA	NA	NA	NA	NA	NA	NA	NA	NA	33.33	100
Chlorpyrifos	DR	3	80	18776	0	41.67	0	0	100	NA	NA	NA	NA	NA	NA	NA	NA	NA	NA	NA	NA	NA	NA	NA	NA	NA	41.67	100
Chlorpyrifos	DR	3	100	18776	8.33	36.36	0	0	100	NA	NA	NA	NA	NA	NA	NA	NA	NA	NA	NA	NA	NA	NA	NA	NA	NA	36.36	100
Chlorpyrifos	LD	1	0	18738	8.33	0	0	0	8.33	0	0	0	0	0	0	0	0	0	0	0	0	0	0	0	0	0	0	8.33
Chlorpyrifos	LD	1	2.54	18738	16.67	0	0	0	16.67	10	0	0	0	0	0	0	0	0	0	0	0	0	0	0	0	0	10	25
Chlorpyrifos	LD	1	10	18738	8.33	0	0	0	8.33	18.18	0	0	0	0	0	0	0	0	0	0	0	0	0	0	0	0	18.18	25
Chlorpyrifos	LD	1	20	18738	0	8.33	0	0	0	25	0	0	0	16.67	0	0	0	0	0	0	0	0	0	0	0	0	33.33	33.33
Chlorpyrifos	LD	1	40	18738	16.67	10	0	0	33.33	50	0	0	0	0	0	12.5	0	0	0	0	0	0	0	0	0	0	50	66.67
Chlorpyrifos	LD	1	60	18738	16.67	10	0	0	41.67	100	14.29	14.29	14.29	42.86	14.29	57.14	14.29	0	14.29	14.29	0	0	57.14	14.29	0	14.29	70	100
Chlorpyrifos	LD	1	80	18738	0	0	0	0	66.67	100	100	75	75	75	50	75	75	0	75	75	0	0	100	75	0	75	33.33	100
Chlorpyrifos	LD	1	100	18738	0	0	0	0	83.33	100	100	100	100	100	100	100	100	0	100	100	0	0	100	100	0	100	16.67	100
Chlorpyrifos	LD	2	0	18790	16.67	0	0	0	16.67	0	0	0	0	0	0	0	0	0	0	0	0	0	0	0	0	0	0	16.67
Chlorpyrifos	LD	2	2.54	18790	8.33	0	0	0	8.33	0	0	0	0	0	0	0	0	0	0	0	0	0	0	0	0	0	0	8.33
Chlorpyrifos	LD	2	10	18790	25	11.11	0	0	25	0	0	0	0	11.11	0	11.11	0	0	0	11.11	0	0	11.11	0	0	0	11.11	33.33
Chlorpyrifos	LD	2	20	18790	25	11.11	0	0	33.33	37.5	0	0	0	25	0	12.5	0	0	0	25	0	0	25	0	0	0	44.44	66.67

Test Agent	Treatment	Replicate plate #	Concentration	Plate Barcode	MO24	DP24	SM24	NC24	MORT	YSE_	AXIS	EYE_	SNOU	JAW_	OTIC	PE__	BRAI	SOMI	PFIN	CFIN	PIG_	CIRC	TRUN	SWIM	NC__	TR__	.except.M(	any.effect
Chlorpyrifos	LD	2	40	18790	8.33	9.09	0	0	16.67	40	0	0	0	20	0	30	0	0	0	0	0	0	30	0	0	0	45.45	50
Chlorpyrifos	LD	2	60	18790	0	0	0	0	41.67	85.71	85.71	85.71	100	100	14.29	85.71	42.86	0	71.43	100	0	0	100	57.14	0	85.71	58.33	100
Chlorpyrifos	LD	2	80	18790	8.33	9.09	0	0	83.33	100	50	0	0	50	0	0	0	0	0	50	0	0	50	0	0	0	27.27	100
Chlorpyrifos	LD	2	100	18790	8.33	18.18	0	0	75	66.67	0	0	0	33.33	0	0	0	0	0	0	0	0	0	0	0	0	36.36	100
Chlorpyrifos	LD	3	0	18791	8.33	0	0	0	8.33	0	0	0	0	0	0	0	0	0	0	0	0	0	0	0	0	0	0	8.33
Chlorpyrifos	LD	3	2.54	18791	0	0	0	0	0	0	0	0	0	0	0	0	0	0	0	0	0	0	0	0	0	0	0	0
Chlorpyrifos	LD	3	10	18791	8.33	0	0	0	8.33	9.09	0	9.09	9.09	9.09	0	9.09	0	0	9.09	9.09	0	0	18.18	9.09	0	0	18.18	25
Chlorpyrifos	LD	3	20	18791	16.67	0	0	0	16.67	30	0	0	0	0	0	0	0	0	0	0	0	0	0	0	0	0	30	41.67
Chlorpyrifos	LD	3	40	18791	8.33	0	0	0	8.33	72.73	0	0	0	9.09	0	9.09	0	0	0	0	0	0	27.27	0	0	0	72.73	75
Chlorpyrifos	LD	3	60	18791	0	0	0	0	33.33	100	25	37.5	37.5	50	0	50	12.5	0	37.5	62.5	0	0	87.5	37.5	0	50	66.67	100
Chlorpyrifos	LD	3	80	18791	8.33	0	0	0	58.33	100	100	80	80	80	20	60	60	0	80	80	0	0	100	60	0	80	45.45	100
Chlorpyrifos	LD	3	100	18791	0	0	0	0	75	100	66.67	66.67	66.67	66.67	33.33	66.67	33.33	0	66.67	66.67	0	0	66.67	66.67	0	66.67	25	100
Chlorpyrifos	Standard	1	0	19372	0	0	0	0	0	0	0	0	0	0	0	0	0	0	8.33	0	0	0	0	0	0	0	8.33	8.33
Chlorpyrifos	Standard	1	2.54	19372	0	0	0	0	0	0	0	0	0	0	0	0	0	0	0	0	0	0	0	0	0	0	0	0
Chlorpyrifos	Standard	1	10	19372	0	0	0	0	0	0	0	0	0	0	0	0	0	0	0	0	0	0	0	0	0	0	0	0
Chlorpyrifos	Standard	1	20	19372	8.33	0	0	0	8.33	0	0	0	0	0	0	0	0	0	9.09	0	0	0	0	0	0	0	9.09	16.67
Chlorpyrifos	Standard	1	40	19372	0	0	0	0	0	0	0	0	0	0	0	0	0	0	0	0	0	0	0	0	0	0	0	0
Chlorpyrifos	Standard	1	60	19372	0	16.67	0	0	8.33	9.09	18.18	9.09	9.09	9.09	9.09	9.09	0	9.09	9.09	0	0	0	9.09	0	9.09	9.09	25	25
Chlorpyrifos	Standard	1	80	19372	0	0	0	0	0	16.67	66.67	8.33	16.67	16.67	0	16.67	0	0	16.67	0	0	0	25	0	0	8.33	75	75
Chlorpyrifos	Standard	1	100	19372	8.33	0	0	0	16.67	60	70	40	40	60	0	40	20	0	60	0	0	0	0	0	0	30	81.82	91.67
Chlorpyrifos	Standard	2	0	19376	8.33	0	0	0	8.33	0	0	0	0	0	0	0	0	0	0	0	0	0	0	0	0	0	0	8.33
Chlorpyrifos	Standard	2	2.54	19376	0	0	0	0	0	0	0	0	0	0	0	0	0	0	0	0	0	0	8.33	0	0	0	8.33	8.33
Chlorpyrifos	Standard	2	10	19376	0	0	0	0	0	0	0	0	0	0	0	0	0	0	0	0	0	0	0	0	0	0	0	0
Chlorpyrifos	Standard	2	20	19376	0	0	0	0	0	8.33	8.33	8.33	8.33	8.33	8.33	8.33	8.33	0	8.33	8.33	0	8.33	8.33	0	0	8.33	8.33	8.33
Chlorpyrifos	Standard	2	40	19376	0	0	0	0	0	0	0	0	0	0	0	0	0	0	0	0	0	0	0	0	0	0	0	0
Chlorpyrifos	Standard	2	60	19376	0	0	0	0	0	16.67	0	8.33	25	25	0	8.33	0	0	16.67	0	0	0	8.33	0	0	0	33.33	33.33
Chlorpyrifos	Standard	2	80	19376	0	8.33	0	0	0	16.67	50	8.33	25	25	0	16.67	0	0	25	0	0	0	33.33	0	0	8.33	75	75
Chlorpyrifos	Standard	2	100	19376	8.33	9.09	0	0	16.67	20	80	20	30	30	0	20	10	0	20	0	0	0	40	0	0	20	100	100
Chlorpyrifos	Standard	3	0	19396	0	0	0	0	0	0	0	0	0	0	0	0	0	0	0	0	0	0	0	0	0	0	0	0
Chlorpyrifos	Standard	3	2.54	19396	0	0	0	0	0	0	0	0	0	0	0	0	0	0	0	0	0	0	0	0	0	0	0	0
Chlorpyrifos	Standard	3	10	19396	0	0	0	0	0	0	0	0	0	0	0	0	0	0	0	0	0	0	0	0	0	0	0	0
Chlorpyrifos	Standard	3	20	19396	0	0	0	0	0	0	0	0	8.33	8.33	0	0	0	0	0	0	0	0	0	0	0	0	8.33	8.33
Chlorpyrifos	Standard	3	40	19396	0	0	0	0	0	0	0	0	0	0	0	0	0	0	0	0	0	0	0	0	0	0	0	0
Chlorpyrifos	Standard	3	60	19396	8.33	18.18	0	0	16.67	10	10	10	40	40	0	10	0	0	20	0	0	0	10	0	0	10	54.55	58.33
Chlorpyrifos	Standard	3	80	19396	0	33.33	0	0	8.33	36.36	54.55	36.36	36.36	45.45	9.09	36.36	9.09	0	36.36	0	0	0	63.64	0	0	18.18	91.67	100
Chlorpyrifos	Standard	3	100	19396	0	41.67	0	0	41.67	42.86	71.43	42.86	57.14	57.14	0	42.86	0	0	42.86	0	0	0	57.14	0	0	14.29	75	100
Estradiol	Chorion	1	0	18638	16.67	0	0	0	33.33	0	0	0	0	0	0	0	0	0	0	0	0	0	0	0	0	0	0	33.33
Estradiol	Chorion	1	1	18638	9.09	0	0	0	18.18	0	0	0	0	0	0	11.11	11.11	0	0	0	0	0	0	0	0	0	20	36.36
Estradiol	Chorion	1	2.54	18638	8.33	0	0	0	16.67	0	0	0	0	0	0	0	0	0	0	0	0	0	0	0	0	0	0	16.67
Estradiol	Chorion	1	5	18638	0	0	0	0	8.33	0	0	0	0	0	0	0	0	0	0	0	0	0	0	0	0	0	0	8.33
Estradiol	Chorion	1	7	18638	0	0	0	0	8.33	9.09	9.09	9.09	9.09	9.09	0	9.09	9.09	0	9.09	9.09	0	0	9.09	9.09	0	0	8.33	16.67
Estradiol	Chorion	1	9	18638	8.33	0	0	0	8.33	0	0	0	0	0	0	0	0	0	0	9.09	0	0	9.09	0	0	0	9.09	16.67
Estradiol	Chorion	1	12	18638	0	0	0	0	8.33	72.73	81.82	72.73	81.82	81.82	54.55	72.73	63.64	45.45	63.64	81.82	54.55	0	81.82	63.64	0	81.82	75	83.33
Estradiol	Chorion	1	16.4	18638	0	0	0	0	27.27	87.5	87.5	87.5	87.5	87.5	87.5	87.5	87.5	75	87.5	87.5	62.5	0	87.5	87.5	0	87.5	63.64	90.91
Estradiol	Chorion	2	0	18676	0	0	0	0	0	0	0	0	0	0	0	0	0	0	0	0	0	0	0	0	0	0	0	0
Estradiol	Chorion	2	1	18676	0	0	0	0	0	0	0	0	0	0	0	0	0	0	0	0	0	0	0	0	0	0	0	0
Estradiol	Chorion	2	2.54	18676	8.33	0	0	0	8.33	0	0	0	0	0	0	0	0	0	0	0	0	0	0	0	0	0	0	8.33
Estradiol	Chorion	2	5	18676	0	0	0	0	18.18	0	0	0	0	0	0	0	0	0	0	0	0	0	0	0	0	0	0	18.18
Estradiol	Chorion	2	7	18676	8.33	0	0	0	8.33	0	0	0	0	0	0	0	0	0	0	0	0	0	0	0	0	0	0	8.33
Estradiol	Chorion	2	9	18676	0	0	0	0	0	0	0	0	0	0	0	0	0	0	0	0	0	0	0	0	0	0	0	0
Estradiol	Chorion	2	12	18676	0	0	0	0	33.33	75	75	75	75	87.5	37.5	62.5	62.5	25	75	87.5	0	0	87.5	75	0	87.5	58.33	91.67
Estradiol	Chorion	2	16.4	18676	8.33	0	0	0	75	100	100	100	100	100	100	100	100	100	100	100	33.33	0	100	100	0	100	27.27	100
Estradiol	Chorion	3	0	18677	0	0	0	0	8.33	0	0	0	0	0	0	0	0	0	0	0	0	0	0	0	0	0	0	8.33
Estradiol	Chorion	3	1	18677	0	0	0	0	0	0	0	0	0	0	0	0	0	0	0	0	0	0	0	0	0	0	0	0
Estradiol	Chorion	3	2.54	18677	0	0	0	0	0	0	0	0	0	0	0	0	0	0	0	0	0	0	0	0	0	0	0	0
Estradiol	Chorion	3	5	18677	0	0	0	0	0	0	0	0	0	0	0	0	0	0	0	0	0	0	0	0	0	0	0	0
Estradiol	Chorion	3	7	18677	0	0	0	0	0	0	0	0	0	0	0	0	0	0	0	0	0	0	0	0	0	0	0	0
Estradiol	Chorion	3	9	18677	8.33	0	0	0	8.33	0	9.09	0	9.09	9.09	0	0	0	0	0	27.27	0	0	27.27	0	0	9.09	27.27	33.33
Estradiol	Chorion	3	12	18677	0	0	0	0	8.33	72.73	63.64	63.64	72.73	72.73	63.64	72.73	63.64	54.55	63.64	90.91	27.27	9.09	81.82	54.55	0	54.55	83.33	91.67
Estradiol	Chorion	3	16.4	18677	0	0	0	0	50	83.33	66.67	83.33	83.33	83.33	66.67	83.33	83.33	83.33	83.33	83.33	50	0	66.67	66.67	0	66.67	41.67	91.67
Estradiol	DR	1	0	18668	0	0	0	0	8.33	0	0	0	0	0	0	0	0	0	0	0	0	0	0	0	0	0	0	8.33
Estradiol	DR	1	1	18668	0	0	0	0	16.67	0	0	0	0	0	0	0	0	0	0	0	0	0	0	0	0	0	0	16.67
Estradiol	DR	1	2.54	18668	0	8.33	0	0	25	11.11	11.11	11.11	11.11	11.11	11.11	11.11	0	0	11.11	11.11	0	0	11.11	11.11	0	0	8.33	33.33
Estradiol	DR	1	5	18668	8.33	0	0	0	16.67	0	0	0	0	0	0	0	0	0	0	0	0	0	0	0	0	0	0	16.67
Estradiol	DR	1	7	18668	0	8.33	0	0	8.33	0	0	0	0	0	0	0	0	0	0	0	0	0	0	0	0	0	8.33	16.67
Estradiol	DR	1	9	18668	0	33.33	0	0	25	0	0	0	11.11	11.11	0	0	0	0	0	22.22	0	0	22.22	0	0	0	33.33	58.33
Estradiol	DR	1	12	18668	8.33	72.73	0	0	16.67	100	90	100	100	100	90	100	90	80	100	100	60	20	100	90	20	80	90.91	100
Estradiol	DR	1	16.4	18668	8.33	90.91	0	0	75	100	100	100	100	100	100	100	100	100	100	100	100	0	100	66.67	33.33	66.67	100	100
Estradiol	DR	2	0	18683	8.33	0	0	0	8.33	9.09	0	9.09	9.09	9.09	0	9.09	9.09	0	9.09	9.09	0	0	9.09	9.09	0	9.09	9.09	16.67
Estradiol	DR	2	1	18683	0	8.33	0	0	16.67	0	0	0	0	0	0	0	0	0	0	0	0	0	0	0	0	0	8.33	25
Estradiol	DR	2	2.54	18683	0	0	0	0	16.67	10	0	10	10	10	0	10	10	0	10	10	0	0	10	10	0	10	8.33	25
Estradiol	DR	2	5	18683	8.33	9.09	0	0	16.67	20	0	20	20	20	0	20	10	0	10	20	0	0	20	0	0	20	18.18	33.33
Estradiol	DR	2	7	18683	25	33.33	0	0	58.33	20	20	20	20	20	20	20	20	20	20	20	20	0	20	20	0	20	33.33	66.67
Estradiol	DR	2	9	18683	0	41.67	0	0	25	22.22	55.56	33.33	33.33	33.33	0	44.44	22.22	11.11	33.33	66.67	11.11	0	66.67	22.22	0	33.33	58.33	75
Estradiol	DR	2	12	18683	0	81.82	0	0	36.36	100	100	100	100	100	85.71	100	100	85.71	100	100	57.14	0	100	100	0	100	81.82	100
Estradiol	DR	2	16.4	18683	0	58.33	0	0	75	100	100	100	100	100	100	100	100	100	100	100	66.67	0	100	100	0	100	66.67	100
Estradiol	DR	3	0	18684	8.33	9.09	0	0	16.67	0	0	0	0	0	0	0	0	0	0	0	0	0	0	0	0	0	9.09	25
Estradiol	DR	3	1	18684	0	0	0	0	0	0	0	0	0	0	0	0	0	0	0	0	0	0	0	0	0	0	0	0

Test Agent	Treatment	Replicate plate #	Concentration	Plate Barcode	MO24	DP24	SM24	NC24	MORT	YSE_	AXIS	EYE_	SNOU	JAW_	OTIC	PE__	BRAI	SOMI	PFIN	CFIN	PIG_	CIRC	TRUN	SWIM	NC__	TR__	.except.M(	any.effect
Estradiol	DR	3	2.54	18684	0	0	0	0	8.33	0	0	0	0	0	0	0	0	0	0	0	0	0	0	0	0	0	0	8.33
Estradiol	DR	3	5	18684	0	0	0	0	16.67	0	0	0	0	0	0	0	0	0	0	0	0	0	0	0	0	0	0	16.67
Estradiol	DR	3	7	18684	0	16.67	0	0	8.33	9.09	18.18	9.09	9.09	9.09	0	9.09	9.09	0	9.09	18.18	0	0	18.18	9.09	0	9.09	25	33.33
Estradiol	DR	3	9	18684	0	33.33	0	0	33.33	50	62.5	37.5	37.5	50	25	62.5	37.5	25	50	50	37.5	0	62.5	37.5	0	37.5	50	75
Estradiol	DR	3	12	18684	0	75	0	0	16.67	90	100	100	100	100	90	100	100	100	100	100	100	10	100	100	0	100	91.67	100
Estradiol	DR	3	16.4	18684	0	83.33	0	0	16.67	100	100	100	100	100	90	100	100	100	100	100	100	10	100	100	0	100	91.67	100
Estradiol	LD	1	0	18625	8.33	0	0	0	8.33	0	0	0	0	0	0	0	0	0	0	0	0	0	0	0	0	0	0	8.33
Estradiol	LD	1	1	18625	8.33	0	0	0	8.33	0	0	0	0	0	0	0	0	0	0	0	0	0	0	0	0	0	0	8.33
Estradiol	LD	1	2.54	18625	8.33	0	0	0	8.33	0	0	0	0	0	0	0	0	0	0	0	0	0	0	0	0	0	0	8.33
Estradiol	LD	1	5	18625	8.33	0	0	0	8.33	0	0	0	0	0	0	0	0	0	0	0	0	0	0	0	0	0	0	8.33
Estradiol	LD	1	7	18625	8.33	27.27	0	0	8.33	9.09	9.09	9.09	9.09	9.09	9.09	9.09	9.09	0	9.09	9.09	0	0	9.09	9.09	0	0	36.36	41.67
Estradiol	LD	1	9	18625	16.67	50	0	0	16.67	30	20	10	10	10	10	10	10	10	10	40	10	0	60	10	0	10	70	75
Estradiol	LD	1	12	18625	8.33	81.82	0	0	16.67	100	100	100	100	100	100	100	100	80	90	100	100	0	100	100	0	100	100	100
Estradiol	LD	1	16.4	18625	16.67	60	0	0	25	100	100	100	100	100	100	100	100	88.89	100	100	100	22.22	100	100	0	100	100	100
Estradiol	LD	2	0	18687	8.33	0	0	0	8.33	0	0	0	0	0	0	0	0	0	0	0	0	0	0	0	0	0	0	8.33
Estradiol	LD	2	1	18687	0	0	0	0	0	0	0	0	0	0	0	0	0	0	0	0	0	0	0	0	0	0	0	0
Estradiol	LD	2	2.54	18687	0	0	0	0	8.33	0	0	0	0	0	0	0	0	0	0	0	0	0	0	0	0	0	0	8.33
Estradiol	LD	2	5	18687	8.33	9.09	0	0	8.33	0	0	9.09	9.09	9.09	0	0	0	0	0	9.09	0	0	9.09	0	0	0	18.18	25
Estradiol	LD	2	7	18687	16.67	10	0	0	25	0	0	0	0	0	0	0	0	0	0	11.11	0	0	11.11	0	0	0	10	33.33
Estradiol	LD	2	9	18687	8.33	9.09	0	0	8.33	0	0	0	0	0	0	0	0	0	0	0	0	0	0	0	0	0	9.09	16.67
Estradiol	LD	2	12	18687	8.33	81.82	0	0	8.33	90.91	90.91	90.91	90.91	90.91	72.73	90.91	81.82	63.64	90.91	100	72.73	0	100	90.91	0	81.82	100	100
Estradiol	LD	2	16.4	18687	0	91.67	0	0	0	100	100	100	100	100	100	100	100	100	100	100	100	16.67	100	100	8.33	91.67	100	100
Estradiol	LD	3	0	18690	0	0	0	0	8.33	0	0	0	0	0	0	0	0	0	0	0	0	0	0	0	0	0	0	8.33
Estradiol	LD	3	1	18690	8.33	0	0	0	8.33	0	0	0	0	0	0	0	0	0	0	0	0	0	0	0	0	0	0	8.33
Estradiol	LD	3	2.54	18690	0	0	0	0	0	0	0	0	0	0	0	0	0	0	0	0	0	0	0	0	0	0	0	0
Estradiol	LD	3	5	18690	8.33	0	0	0	8.33	0	0	0	0	0	0	0	0	0	0	0	0	0	0	0	0	0	0	8.33
Estradiol	LD	3	7	18690	8.33	9.09	0	0	8.33	0	0	0	0	0	0	0	0	0	0	9.09	0	0	9.09	0	0	0	9.09	16.67
Estradiol	LD	3	9	18690	0	25	0	0	0	16.67	25	16.67	16.67	16.67	16.67	25	16.67	16.67	16.67	41.67	8.33	0	41.67	16.67	0	25	41.67	41.67
Estradiol	LD	3	12	18690	0	83.33	0	0	33.33	87.5	100	100	100	100	100	100	100	100	100	100	87.5	0	100	100	0	100	83.33	100
Estradiol	LD	3	16.4	18690	8.33	90.91	0	0	50	83.33	100	100	100	100	100	100	100	100	100	100	100	0	100	100	0	100	100	100
Estradiol	Standard	1	0	18620	0	0	0	0	0	0	0	0	0	0	0	0	0	0	0	0	0	0	0	0	0	0	0	0
Estradiol	Standard	1	1	18620	8.33	0	0	0	8.33	0	0	0	0	0	0	0	0	0	0	0	0	0	0	0	0	0	0	8.33
Estradiol	Standard	1	2.54	18620	0	0	0	0	8.33	0	0	0	0	0	0	0	0	0	0	0	0	0	0	0	0	0	0	8.33
Estradiol	Standard	1	5	18620	0	0	0	0	0	8.33	0	8.33	8.33	8.33	0	8.33	0	0	8.33	0	0	0	0	0	0	0	8.33	8.33
Estradiol	Standard	1	7	18620	33.33	12.5	0	0	33.33	25	12.5	25	25	25	0	25	12.5	0	25	0	0	12.5	0	0	0	12.5	25	50
Estradiol	Standard	1	9	18620	8.33	81.82	0	0	8.33	18.18	9.09	27.27	27.27	27.27	0	18.18	18.18	0	18.18	0	0	0	36.36	0	0	18.18	81.82	83.33
Estradiol	Standard	1	12	18620	0	91.67	0	0	8.33	90.91	0	100	100	90.91	18.18	90.91	81.82	9.09	90.91	18.18	0	9.09	100	0	0	90.91	100	100
Estradiol	Standard	1	16.4	18620	8.33	100	0	0	16.67	100	10	100	100	90	10	90	80	0	90	30	0	20	100	0	0	100	100	100
Estradiol	Standard	2	0	18661	0	0	0	0	0	0	0	0	0	0	0	0	0	0	0	0	0	0	0	0	0	0	0	0
Estradiol	Standard	2	1	18661	25	0	0	0	25	0	0	0	0	0	0	0	0	0	0	0	0	0	0	0	0	0	0	25
Estradiol	Standard	2	2.54	18661	8.33	0	0	0	8.33	0	0	0	0	0	0	0	0	0	0	0	0	0	0	0	0	0	0	8.33
Estradiol	Standard	2	5	18661	8.33	0	0	0	8.33	0	0	0	0	0	0	0	0	0	0	0	0	0	0	0	0	0	0	8.33
Estradiol	Standard	2	7	18661	8.33	0	0	0	8.33	0	0	0	0	0	0	0	0	0	0	0	0	0	0	0	0	0	0	8.33
Estradiol	Standard	2	9	18661	0	66.67	0	0	0	25	8.33	16.67	16.67	16.67	0	8.33	8.33	0	16.67	0	0	8.33	25	0	0	8.33	75	75
Estradiol	Standard	2	12	18661	16.67	100	0	0	16.67	100	10	100	90	100	10	100	70	10	100	30	0	40	100	0	10	100	100	100
Estradiol	Standard	2	16.4	18661	0	91.67	0	0	8.33	100	36.36	100	100	100	9.09	100	81.82	18.18	100	9.09	9.09	54.55	90.91	0	9.09	100	100	100
Estradiol	Standard	3	0	18685	8.33	9.09	0	0	8.33	0	0	0	0	0	0	0	0	0	0	0	0	0	0	0	0	0	9.09	16.67
Estradiol	Standard	3	1	18685	0	0	0	0	0	8.33	8.33	8.33	8.33	8.33	0	8.33	8.33	0	8.33	0	0	0	0	0	0	8.33	8.33	8.33
Estradiol	Standard	3	2.54	18685	8.33	0	0	0	8.33	0	0	0	0	0	0	0	0	0	0	0	0	0	0	0	0	0	0	8.33
Estradiol	Standard	3	5	18685	8.33	9.09	0	0	16.67	10	10	10	10	10	0	10	0	0	10	10	0	0	0	0	0	0	18.18	33.33
Estradiol	Standard	3	7	18685	16.67	10	0	0	16.67	10	10	10	10	10	0	10	0	0	10	0	0	0	0	0	0	10	10	25
Estradiol	Standard	3	9	18685	8.33	36.36	0	0	8.33	18.18	0	27.27	27.27	27.27	0	18.18	9.09	9.09	18.18	9.09	0	0	45.45	0	0	18.18	45.45	50
Estradiol	Standard	3	12	18685	8.33	100	0	0	25	88.89	11.11	88.89	88.89	88.89	0	77.78	44.44	0	88.89	11.11	0	0	77.78	0	0	66.67	100	100
Estradiol	Standard	3	16.4	18685	8.33	81.82	0	0	8.33	100	9.09	100	100	100	0	90.91	72.73	0	100	9.09	0	9.09	90.91	0	0	100	100	100
Hydroxyurea	Chorion	1	0	18747	0	0	0	0	0	0	0	0	0	0	0	0	0	0	0	0	0	0	0	0	0	0	0	0
Hydroxyurea	Chorion	1	1	18747	0	0	0	0	0	0	0	0	0	0	0	0	0	0	0	0	0	0	0	0	0	0	0	0
Hydroxyurea	Chorion	1	2.54	18747	0	0	0	0	0	8.33	0	8.33	8.33	8.33	0	8.33	0	0	8.33	0	0	0	8.33	8.33	0	0	8.33	8.33
Hydroxyurea	Chorion	1	6.45	18747	0	0	0	0	0	0	0	0	0	0	0	0	0	0	0	0	0	0	0	0	0	0	0	0
Hydroxyurea	Chorion	1	16.4	18747	0	0	0	0	0	0	0	0	0	0	0	0	0	0	0	0	0	0	0	0	0	0	0	0
Hydroxyurea	Chorion	1	35	18747	0	0	0	0	0	0	0	0	0	0	0	0	0	0	0	0	0	0	0	0	0	0	0	0
Hydroxyurea	Chorion	1	74.8	18747	0	8.33	0	0	8.33	0	0	0	0	0	0	0	0	0	0	0	0	0	0	0	0	0	8.33	8.33
Hydroxyurea	Chorion	1	100	18747	0	0	0	0	0	0	0	0	0	0	0	0	0	0	0	0	0	0	0	0	0	0	0	0
Hydroxyurea	Chorion	2	0	18766	0	0	0	0	0	8.33	0	0	8.33	0	0	8.33	0	0	8.33	8.33	0	0	8.33	0	0	0	8.33	8.33
Hydroxyurea	Chorion	2	1	18766	0	0	0	0	0	0	0	0	0	0	0	0	0	0	0	0	0	0	0	0	0	0	0	0
Hydroxyurea	Chorion	2	2.54	18766	0	0	0	0	0	0	0	0	0	0	0	0	0	0	0	0	0	0	0	0	0	0	0	0
Hydroxyurea	Chorion	2	6.45	18766	0	0	0	0	0	0	0	0	0	0	0	0	0	0	0	0	0	0	0	0	0	0	0	0
Hydroxyurea	Chorion	2	16.4	18766	0	8.33	0	0	8.33	0	0	0	0	0	0	0	0	0	0	0	0	0	0	0	0	0	8.33	8.33
Hydroxyurea	Chorion	2	35	18766	0	0	0	0	0	8.33	0	8.33	8.33	8.33	0	8.33	0	0	8.33	8.33	0	0	8.33	0	0	0	8.33	8.33
Hydroxyurea	Chorion	2	74.8	18766	8.33	0	0	0	8.33	0	0	0	0	0	0	0	0	0	0	0	0	0	0	0	0	0	0	8.33
Hydroxyurea	Chorion	2	100	18766	0	0	0	0	0	8.33	8.33	8.33	8.33	8.33	8.33	8.33	8.33	8.33	8.33	8.33	0	0	8.33	8.33	0	8.33	8.33	8.33
Hydroxyurea	Chorion	3	0	18767	0	8.33	0	0	0	8.33	8.33	8.33	8.33	8.33	8.33	8.33	8.33	8.33	8.33	8.33	0	0	8.33	8.33	0	8.33	8.33	8.33
Hydroxyurea	Chorion	3	1	18767	0	0	0	0	0	0	0	0	0	0	0	0	0	0	0	0	0	0	0	0	0	0	0	0
Hydroxyurea	Chorion	3	2.54	18767	0	0	0	0	0	0	0	0	0	0	0	0	0	0	0	0	0	0	0	0	0	0	0	0
Hydroxyurea	Chorion	3	6.45	18767	0	0	0	0	0	8.33	8.33	8.33	8.33	8.33	8.33	8.33	8.33	0	8.33	8.33	0	0	8.33	8.33	0	0	8.33	8.33
Hydroxyurea	Chorion	3	16.4	18767	0	0	0	0	0	0	0	0	0	0	0	0	0	0	0	0	0	0	0	0	0	0	0	0
Hydroxyurea	Chorion	3	35	18767	0	0	0	0	0	16.67	8.33	8.33	8.33	8.33	8.33	16.67	8.33	8.33	16.67	16.67	0	0	16.67	8.33	0	8.33	16.67	16.67
Hydroxyurea	Chorion	3	74.8	18767	0	0	0	0	0	0	0	0	0	0	0	0	0	0	0	0	0	0	0	0	0	0	0	0
Hydroxyurea	Chorion	3	100	18767	0	0	0	0	0	8.33	0	0	8.33	8.33	0	8.33	0	0	8.33	0	0	0	0	0	0	0	8.33	8.33

Test Agent	Treatment	Replicate plate #	Concentration	Plate Barcode	MO24	DP24	SM24	NC24	MORT	YSE_	AXIS	EYE_	SNOU	JAW_	OTIC	PE__	BRAI	SOMI	PFIN	CFIN	PIG_	CIRC	TRUN	SWIM	NC__	TR__	.except.M(	any.effect
Hydroxyurea	DR	1	0	18753	0	0	0	0	8.33	0	0	0	0	0	0	0	0	0	0	0	0	0	0	0	0	0	0	8.33
Hydroxyurea	DR	1	1	18753	0	0	0	0	16.67	0	0	0	0	0	0	0	0	0	0	0	0	0	0	0	0	0	0	16.67
Hydroxyurea	DR	1	2.54	18753	0	0	0	0	0	0	0	0	0	0	0	0	0	0	0	0	0	0	0	0	0	0	0	0
Hydroxyurea	DR	1	6.45	18753	0	0	0	0	0	0	0	0	0	0	0	0	0	0	0	0	0	0	0	0	0	0	0	0
Hydroxyurea	DR	1	16.4	18753	0	0	0	0	8.33	9.09	0	0	0	0	0	0	0	0	0	0	0	0	0	0	0	0	8.33	16.67
Hydroxyurea	DR	1	35	18753	0	0	0	0	8.33	0	0	0	0	0	0	0	0	0	0	0	0	0	0	0	0	0	0	8.33
Hydroxyurea	DR	1	74.8	18753	0	0	0	0	16.67	0	0	0	0	0	0	0	0	0	0	0	0	0	0	0	0	0	0	16.67
Hydroxyurea	DR	1	100	18753	0	0	0	0	18.18	11.11	0	0	0	0	0	0	0	0	0	0	0	0	0	0	0	0	9.09	27.27
Hydroxyurea	DR	2	0	18769	0	0	0	0	16.67	0	0	0	0	0	0	0	0	0	0	0	0	0	0	0	0	0	0	16.67
Hydroxyurea	DR	2	1	18769	0	0	0	0	8.33	0	0	0	0	0	0	0	0	0	0	0	0	0	0	0	0	0	0	8.33
Hydroxyurea	DR	2	2.54	18769	0	0	0	0	25	0	11.11	0	0	0	0	0	0	0	0	0	0	0	11.11	0	0	0	8.33	33.33
Hydroxyurea	DR	2	6.45	18769	0	8.33	0	0	16.67	10	0	10	10	10	0	10	10	0	10	10	0	0	10	10	0	0	8.33	25
Hydroxyurea	DR	2	16.4	18769	0	0	0	0	8.33	0	0	0	0	0	0	0	0	0	0	0	0	0	0	0	0	0	0	8.33
Hydroxyurea	DR	2	35	18769	0	0	0	0	25	0	0	0	0	0	0	0	0	0	0	0	0	0	0	0	0	0	0	25
Hydroxyurea	DR	2	74.8	18769	0	8.33	0	0	8.33	0	0	0	0	0	0	9.09	0	0	0	0	0	0	9.09	0	0	0	8.33	16.67
Hydroxyurea	DR	2	100	18769	0	8.33	0	0	0	0	0	0	0	0	0	0	0	0	0	8.33	0	0	8.33	0	0	0	8.33	8.33
Hydroxyurea	DR	3	0	19213	0	0	0	0	0	0	0	0	0	0	0	0	0	0	0	0	0	0	8.33	0	0	0	8.33	8.33
Hydroxyurea	DR	3	1	19213	0	8.33	0	0	0	0	0	0	0	0	0	8.33	0	0	0	0	0	0	0	0	0	0	16.67	16.67
Hydroxyurea	DR	3	2.54	19213	0	0	0	0	0	8.33	0	8.33	8.33	8.33	0	8.33	0	0	8.33	0	0	0	0	0	0	0	8.33	8.33
Hydroxyurea	DR	3	6.45	19213	0	0	0	0	0	0	0	0	0	9.09	0	9.09	0	0	0	0	0	0	0	0	0	0	9.09	9.09
Hydroxyurea	DR	3	16.4	19213	0	0	0	0	0	0	0	0	0	0	0	0	0	0	0	0	0	0	0	0	0	0	0	0
Hydroxyurea	DR	3	35	19213	0	0	0	0	0	0	0	0	0	0	0	0	0	0	0	0	0	0	0	0	0	0	0	0
Hydroxyurea	DR	3	74.8	19213	0	0	0	0	0	0	0	0	0	0	0	0	0	0	0	0	0	0	0	0	0	0	0	0
Hydroxyurea	DR	3	100	19213	0	0	0	0	0	0	0	0	0	0	0	0	0	0	0	0	0	0	0	0	0	0	0	0
Hydroxyurea	LD	1	0	18725	25	11.11	0	0	25	11.11	11.11	11.11	11.11	11.11	11.11	11.11	11.11	0	11.11	11.11	0	0	11.11	0	0	11.11	11.11	33.33
Hydroxyurea	LD	1	1	18725	25	0	0	0	25	0	0	0	0	0	0	0	0	0	0	0	0	0	0	0	0	0	0	25
Hydroxyurea	LD	1	2.54	18725	8.33	0	0	0	8.33	9.09	9.09	9.09	9.09	9.09	9.09	9.09	9.09	0	9.09	9.09	0	0	9.09	9.09	0	9.09	9.09	16.67
Hydroxyurea	LD	1	6.45	18725	25	0	0	0	25	0	0	0	0	0	0	0	0	0	0	0	0	0	0	0	0	0	0	25
Hydroxyurea	LD	1	16.4	18725	33.33	12.5	0	0	33.33	12.5	12.5	12.5	12.5	12.5	12.5	12.5	12.5	0	12.5	12.5	0	0	12.5	12.5	0	12.5	12.5	41.67
Hydroxyurea	LD	1	35	18725	0	0	0	0	8.33	0	0	0	0	0	0	0	0	0	0	0	0	0	0	0	0	0	0	8.33
Hydroxyurea	LD	1	74.8	18725	0	0	0	0	0	0	0	0	0	0	0	0	0	0	0	0	0	0	0	0	0	0	0	0
Hydroxyurea	LD	1	100	18725	8.33	0	0	0	8.33	0	0	0	0	0	0	0	0	0	0	0	0	0	0	0	0	0	0	8.33
Hydroxyurea	LD	2	0	18754	0	0	0	0	0	0	0	0	0	0	0	0	0	0	0	0	0	0	0	0	0	0	0	0
Hydroxyurea	LD	2	1	18754	25	0	0	0	25	0	0	0	0	0	0	0	0	0	0	0	0	0	0	0	0	0	0	25
Hydroxyurea	LD	2	2.54	18754	0	0	0	0	0	0	0	0	0	0	0	0	0	0	0	0	0	0	0	0	0	0	0	0
Hydroxyurea	LD	2	6.45	18754	16.67	10	0	0	16.67	10	10	10	10	10	10	10	10	10	10	10	0	0	10	10	0	0	10	25
Hydroxyurea	LD	2	16.4	18754	16.67	20	0	0	25	11.11	11.11	11.11	11.11	11.11	11.11	11.11	11.11	0	11.11	11.11	0	0	11.11	11.11	0	0	20	33.33
Hydroxyurea	LD	2	35	18754	25	11.11	0	0	25	0	0	0	0	0	0	0	0	0	0	0	0	0	0	0	0	0	11.11	33.33
Hydroxyurea	LD	2	74.8	18754	16.67	10	0	0	16.67	10	10	10	10	10	0	10	10	0	10	10	0	0	10	10	0	0	10	25
Hydroxyurea	LD	2	100	18754	16.67	0	0	0	16.67	0	0	0	0	0	0	0	0	0	0	0	0	0	0	0	0	0	0	16.67
Hydroxyurea	LD	3	0	18763	8.33	9.09	0	0	16.67	0	0	0	0	0	0	0	0	0	0	0	0	0	0	0	0	0	9.09	16.67
Hydroxyurea	LD	3	1	18763	16.67	0	0	0	25	0	0	0	0	0	0	0	0	0	0	0	0	0	0	0	0	0	0	25
Hydroxyurea	LD	3	2.54	18763	8.33	0	0	0	8.33	0	0	0	0	0	0	0	0	0	0	0	0	0	0	0	0	0	0	8.33
Hydroxyurea	LD	3	6.45	18763	16.67	0	0	0	25	0	0	0	0	0	0	0	0	0	0	0	0	0	0	0	0	0	0	25
Hydroxyurea	LD	3	16.4	18763	8.33	9.09	0	0	16.67	10	10	10	10	10	10	10	10	10	10	10	0	0	10	10	0	0	9.09	25
Hydroxyurea	LD	3	35	18763	8.33	0	0	0	8.33	0	0	0	0	0	0	0	0	0	0	0	0	0	0	0	0	0	0	8.33
Hydroxyurea	LD	3	74.8	18763	16.67	0	0	0	16.67	0	0	0	0	0	0	0	0	0	0	0	0	0	0	0	0	0	0	16.67
Hydroxyurea	LD	3	100	18763	16.67	10	0	0	25	0	0	0	0	0	0	0	0	0	0	0	0	0	0	0	0	0	10	25
Hydroxyurea	Standard	1	0	18765	16.67	10	0	0	16.67	10	10	10	10	10	10	10	10	10	10	10	10	0	10	10	10	10	10	25
Hydroxyurea	Standard	1	1	18765	16.67	10	0	0	16.67	0	10	0	0	0	0	0	0	0	0	10	0	0	10	0	0	0	10	25
Hydroxyurea	Standard	1	2.54	18765	0	0	0	0	0	0	0	0	0	0	0	0	0	0	0	0	0	0	0	0	0	0	0	0
Hydroxyurea	Standard	1	6.45	18765	16.67	10	0	0	16.67	0	0	0	0	0	0	0	0	0	0	10	0	0	10	0	0	0	10	25
Hydroxyurea	Standard	1	16.4	18765	8.33	0	0	0	8.33	0	0	0	0	0	0	0	0	0	0	0	0	0	0	0	0	0	0	8.33
Hydroxyurea	Standard	1	35	18765	0	8.33	0	0	8.33	0	0	0	0	0	0	0	0	0	0	0	0	0	0	0	0	0	8.33	8.33
Hydroxyurea	Standard	1	74.8	18765	25	0	0	0	25	0	0	0	0	0	0	0	0	0	0	0	0	0	0	0	0	0	0	25
Hydroxyurea	Standard	1	100	18765	0	0	0	0	0	8.33	0	8.33	8.33	8.33	0	8.33	0	0	8.33	8.33	0	0	8.33	8.33	0	0	8.33	8.33
Hydroxyurea	Standard	2	0	18770	8.33	0	0	0	8.33	0	0	0	0	0	0	0	0	0	0	0	0	0	0	0	0	0	0	8.33
Hydroxyurea	Standard	2	1	18770	0	0	0	0	0	0	0	0	0	0	0	0	0	0	0	0	0	0	0	0	0	0	0	0
Hydroxyurea	Standard	2	2.54	18770	0	0	0	0	0	0	0	0	0	0	0	0	0	0	0	0	0	0	0	0	0	0	0	0
Hydroxyurea	Standard	2	6.45	18770	16.67	0	0	0	16.67	0	0	0	0	0	0	0	0	0	0	0	0	0	0	0	0	0	0	16.67
Hydroxyurea	Standard	2	16.4	18770	0	0	0	0	0	8.33	8.33	8.33	8.33	8.33	8.33	8.33	8.33	0	8.33	8.33	0	0	8.33	8.33	0	0	8.33	8.33
Hydroxyurea	Standard	2	35	18770	8.33	0	0	0	8.33	0	0	0	0	0	0	0	0	0	0	0	0	0	0	0	0	0	0	8.33
Hydroxyurea	Standard	2	74.8	18770	0	0	0	0	0	0	0	0	0	0	0	0	0	0	0	0	0	0	0	0	0	0	0	0
Hydroxyurea	Standard	2	100	18770	8.33	18.18	0	0	16.67	0	0	0	0	0	0	0	0	0	0	20	0	0	20	0	0	0	27.27	33.33
Hydroxyurea	Standard	3	0	18822	0	0	0	0	0	0	0	0	0	0	0	0	0	0	0	0	0	0	0	0	0	0	0	0
Hydroxyurea	Standard	3	1	18822	0	8.33	0	0	8.33	0	0	0	0	0	0	0	0	0	0	0	0	0	0	0	0	0	8.33	8.33
Hydroxyurea	Standard	3	2.54	18822	8.33	9.09	0	0	16.67	0	0	0	0	0	0	0	0	0	0	0	0	0	0	0	0	0	9.09	16.67
Hydroxyurea	Standard	3	6.45	18822	0	0	0	0	0	8.33	8.33	8.33	8.33	8.33	0	8.33	8.33	0	8.33	8.33	0	0	8.33	8.33	0	0	8.33	8.33
Hydroxyurea	Standard	3	16.4	18822	8.33	18.18	0	0	16.67	0	0	0	0	0	0	10	0	0	0	0	0	0	10	0	0	0	18.18	25
Hydroxyurea	Standard	3	35	18822	8.33	0	0	0	8.33	0	0	0	0	0	0	0	0	0	0	0	0	0	0	0	0	0	0	8.33
Hydroxyurea	Standard	3	74.8	18822	8.33	0	0	0	16.67	0	0	0	0	0	0	0	0	0	0	0	0	0	0	0	0	0	0	16.67
Hydroxyurea	Standard	3	100	18822	25	0	0	0	25	0	0	0	0	0	0	0	0	0	0	0	0	0	0	0	0	0	0	25
MWCNTs	Chorion	1	0	19297	13.33	0	0	0	13.33	7.69	0	7.69	7.69	7.69	0	7.69	7.69	0	7.69	0	0	0	0	0	0	7.69	7.69	20
MWCNTs	Chorion	1	10	19297	0	0	0	0	0	0	0	0	0	0	0	0	0	0	0	0	0	0	0	0	0	0	0	0
MWCNTs	Chorion	1	23.2	19297	0	0	0	0	6.25	0	0	0	0	0	0	0	0	0	0	0	0	0	0	0	0	0	0	6.25
MWCNTs	Chorion	1	50	19297	0	0	0	0	0	0	0	0	0	0	0	0	0	0	0	0	0	0	0	0	0	0	0	0
MWCNTs	Chorion	1	75	19297	0	0	0	0	0	0	0	0	0	0	0	0	0	0	0	0	0	0	0	0	0	0	0	0
MWCNTs	Chorion	1	100	19297	0	0	0	0	0	0	0	0	0	0	0	0	0	0	0	0	0	0	0	0	0	0	0	0

Test Agent	Treatment	Replicate plate #	Concentration	Plate Barcode	MO24	DP24	SM24	NC24	MORT	YSE_	AXIS	EYE_	SNOU	JAW_	OTIC	PE__	BRAI	SOMI	PFIN	CFIN	PIG_	CIRC	TRUN	SWIM	NC__	TR__	.except.M(	any.effect
MWCNTs	Chorion	2	0	19298	0	0	0	0	0	0	0	0	0	0	0	0	0	0	0	0	0	0	0	0	0	0	0	0
MWCNTs	Chorion	2	10	19298	0	0	0	0	0	0	0	0	0	0	0	0	0	0	0	0	0	0	0	0	0	0	0	0
MWCNTs	Chorion	2	23.2	19298	6.25	0	0	0	6.25	6.67	6.67	6.67	6.67	13.33	0	6.67	0	0	6.67	6.67	0	6.67	6.67	0	0	6.67	13.33	18.75
MWCNTs	Chorion	2	50	19298	0	0	0	0	0	0	0	0	0	0	0	0	0	0	0	0	0	0	0	0	0	0	0	0
MWCNTs	Chorion	2	75	19298	0	0	0	0	0	0	0	0	0	0	0	0	0	0	0	0	0	0	0	0	0	0	0	0
MWCNTs	Chorion	2	100	19298	0	0	0	0	6.67	0	0	0	0	0	0	0	0	0	0	0	0	0	0	0	0	0	0	6.67
MWCNTs	Chorion	3	0	19299	0	0	0	0	0	12.5	6.25	12.5	12.5	12.5	0	12.5	0	0	12.5	0	0	0	6.25	0	0	0	12.5	12.5
MWCNTs	Chorion	3	10	19299	0	0	0	0	0	0	0	0	0	0	0	0	0	0	0	0	0	0	0	0	0	0	0	0
MWCNTs	Chorion	3	23.2	19299	0	0	0	0	0	0	0	0	6.25	6.25	0	0	0	0	0	0	0	0	0	0	0	0	6.25	6.25
MWCNTs	Chorion	3	50	19299	0	0	0	0	6.25	0	0	0	0	0	0	0	0	0	0	0	0	0	0	0	0	0	0	6.25
MWCNTs	Chorion	3	75	19299	6.25	0	0	0	18.75	0	0	0	0	0	0	0	0	0	0	0	0	0	0	0	0	0	0	18.75
MWCNTs	Chorion	3	100	19299	0	0	0	0	0	0	0	0	0	0	0	0	0	0	0	0	0	0	0	0	0	0	0	0
MWCNTs	DR	1	0	19237	0	25	0	0	25	0	0	0	0	0	0	0	0	0	0	0	0	0	0	0	0	0	25	25
MWCNTs	DR	1	10	19237	6.25	6.67	0	0	6.25	6.67	0	6.67	6.67	6.67	0	6.67	0	0	6.67	0	0	0	0	0	0	0	6.67	12.5
MWCNTs	DR	1	23.2	19237	25	16.67	0	0	31.25	0	0	0	0	0	0	0	0	0	0	0	0	0	0	0	0	0	16.67	37.5
MWCNTs	DR	1	50	19237	6.25	13.33	0	0	12.5	0	0	0	0	0	0	0	0	0	0	0	0	0	0	0	0	0	13.33	18.75
MWCNTs	DR	1	75	19237	25	25	0	0	25	0	0	0	0	0	0	0	0	0	0	0	0	0	0	0	0	0	25	43.75
MWCNTs	DR	1	100	19237	18.75	30.77	0	0	25	0	0	0	0	0	0	0	0	0	0	0	0	0	0	0	0	0	30.77	43.75
MWCNTs	DR	2	0	19239	0	0	0	0	0	0	0	0	0	0	0	0	0	0	0	0	0	0	0	0	0	0	0	0
MWCNTs	DR	2	10	19239	0	0	0	0	0	0	0	0	0	0	0	0	0	0	0	0	0	0	0	0	0	0	0	0
MWCNTs	DR	2	23.2	19239	0	0	0	0	0	0	0	0	0	0	0	0	0	0	0	0	0	0	0	0	0	0	0	0
MWCNTs	DR	2	50	19239	12.5	28.57	0	0	12.5	21.43	0	21.43	21.43	21.43	0	21.43	0	0	21.43	0	0	0	7.14	0	0	7.14	28.57	37.5
MWCNTs	DR	2	75	19239	12.5	35.71	0	0	18.75	15.38	0	15.38	15.38	15.38	0	15.38	0	0	15.38	0	0	0	15.38	0	0	0	35.71	43.75
MWCNTs	DR	2	100	19239	12.5	50	0	0	31.25	9.09	0	18.18	27.27	27.27	0	18.18	0	0	9.09	0	0	0	9.09	0	0	9.09	50	56.25
MWCNTs	DR	3	0	19275	0	6.25	0	0	0	6.25	0	6.25	6.25	6.25	6.25	6.25	0	0	6.25	6.25	0	0	6.25	0	0	6.25	6.25	6.25
MWCNTs	DR	3	10	19275	6.25	0	0	0	12.5	0	0	0	0	0	0	0	0	0	0	0	0	0	0	0	0	0	0	12.5
MWCNTs	DR	3	23.2	19275	0	6.25	0	0	6.25	6.67	0	6.67	6.67	6.67	0	6.67	0	0	6.67	0	0	0	0	0	0	0	6.25	12.5
MWCNTs	DR	3	50	19275	6.25	20	0	0	6.25	13.33	6.67	13.33	13.33	13.33	0	13.33	0	0	13.33	6.67	0	0	6.67	0	0	6.67	20	25
MWCNTs	DR	3	75	19275	0	18.75	0	0	6.25	13.33	0	13.33	13.33	13.33	0	13.33	0	0	13.33	6.67	0	0	6.67	0	0	6.67	18.75	18.75
MWCNTs	DR	3	100	19275	0	37.5	0	0	18.75	15.38	0	15.38	15.38	15.38	0	15.38	0	0	15.38	0	0	0	0	0	0	7.69	37.5	37.5
MWCNTs	LD	1	0	19311	0	0	0	0	0	0	0	0	0	0	0	0	0	0	0	0	0	0	0	0	0	0	0	0
MWCNTs	LD	1	10	19311	18.75	0	0	0	18.75	0	0	0	0	0	0	0	0	0	0	0	0	0	0	0	0	0	0	18.75
MWCNTs	LD	1	23.2	19311	0	0	0	0	0	6.25	0	6.25	6.25	6.25	0	6.25	0	0	6.25	0	0	0	0	0	0	0	6.25	6.25
MWCNTs	LD	1	50	19311	12.5	7.14	0	0	12.5	0	0	0	0	0	0	0	0	0	0	0	0	0	0	0	0	0	7.14	18.75
MWCNTs	LD	1	75	19311	0	0	0	0	6.25	0	0	6.67	13.33	13.33	0	6.67	0	0	6.67	0	0	0	13.33	0	0	0	12.5	18.75
MWCNTs	LD	1	100	19311	0	0	0	0	0	0	0	0	0	0	0	0	0	0	0	0	0	0	0	0	0	0	0	0
MWCNTs	LD	2	0	19315	6.25	0	0	0	6.25	0	0	0	0	0	0	0	0	0	0	0	0	0	0	0	0	0	0	6.25
MWCNTs	LD	2	10	19315	6.25	0	0	0	6.25	0	0	6.67	6.67	6.67	0	6.67	0	0	0	0	0	0	0	0	0	0	6.67	12.5
MWCNTs	LD	2	23.2	19315	6.25	6.67	0	0	12.5	0	0	0	0	0	0	0	0	0	0	0	0	0	0	0	0	0	6.67	18.75
MWCNTs	LD	2	50	19315	0	6.25	0	0	0	0	0	0	0	0	0	0	0	0	0	0	0	0	0	0	0	0	6.25	6.25
MWCNTs	LD	2	75	19315	13.33	0	0	0	13.33	0	0	0	0	0	0	0	0	0	0	0	0	0	0	0	0	0	0	13.33
MWCNTs	LD	2	100	19315	23.08	10	0	0	23.08	0	0	0	0	0	0	0	0	0	0	0	0	0	0	0	0	0	10	30.77
MWCNTs	LD	3	0	19323	0	0	0	0	0	0	6.25	0	0	0	0	0	0	0	0	6.25	0	0	6.25	0	0	0	6.25	6.25
MWCNTs	LD	3	10	19323	0	0	0	0	0	6.25	0	6.25	6.25	6.25	0	6.25	0	0	6.25	6.25	0	0	6.25	0	0	0	6.25	6.25
MWCNTs	LD	3	23.2	19323	0	0	0	0	0	6.25	0	6.25	6.25	6.25	0	6.25	0	0	6.25	0	0	0	6.25	0	0	0	6.25	6.25
MWCNTs	LD	3	50	19323	6.25	13.33	0	0	6.25	13.33	0	13.33	13.33	13.33	0	13.33	0	0	13.33	6.67	0	0	13.33	0	0	0	20	25
MWCNTs	LD	3	75	19323	6.25	13.33	0	0	12.5	7.14	7.14	14.29	14.29	14.29	7.14	7.14	7.14	0	7.14	7.14	0	0	14.29	7.14	0	7.14	13.33	25
MWCNTs	LD	3	100	19323	18.75	7.69	0	0	18.75	0	0	23.08	23.08	23.08	0	7.69	0	0	0	7.69	0	0	23.08	0	0	0	23.08	37.5
MWCNTs	Standard	1	0	19309	0	6.25	0	0	6.25	0	0	0	0	0	0	0	0	0	0	0	0	0	0	0	0	0	6.25	6.25
MWCNTs	Standard	1	10	19309	0	0	0	0	0	0	0	0	0	0	0	0	0	0	0	0	0	0	0	0	0	0	0	0
MWCNTs	Standard	1	23.2	19309	0	0	0	0	0	6.25	0	6.25	6.25	6.25	0	6.25	0	0	6.25	0	0	0	6.25	0	0	0	12.5	12.5
MWCNTs	Standard	1	50	19309	6.67	0	0	0	6.67	0	0	0	0	0	0	0	0	0	0	0	0	0	0	0	0	0	0	6.67
MWCNTs	Standard	1	75	19309	37.5	0	0	0	43.75	0	0	0	0	0	0	0	0	0	0	0	0	0	0	0	0	0	0	43.75
MWCNTs	Standard	1	100	19309	46.67	12.5	0	0	46.67	0	0	0	0	0	0	0	0	0	0	0	0	0	0	0	0	0	12.5	53.33
MWCNTs	Standard	2	0	19310	0	0	0	0	0	12.5	0	12.5	12.5	12.5	0	12.5	0	0	12.5	0	0	0	0	0	0	0	12.5	12.5
MWCNTs	Standard	2	10	19310	6.25	0	0	0	6.25	0	0	0	0	0	0	0	0	0	0	0	0	0	0	0	0	0	0	6.25
MWCNTs	Standard	2	23.2	19310	6.25	6.67	0	0	6.25	0	0	0	0	0	0	0	0	0	0	0	0	0	0	0	0	0	6.67	12.5
MWCNTs	Standard	2	50	19310	0	6.67	0	0	6.67	0	0	0	7.14	7.14	0	0	0	0	7.14	0	0	0	0	0	0	0	13.33	20
MWCNTs	Standard	2	75	19310	23.08	10	0	0	30.77	0	0	0	0	0	0	0	0	0	0	0	0	0	0	0	0	0	10	30.77
MWCNTs	Standard	2	100	19310	25	16.67	0	0	37.5	0	0	0	0	0	0	0	0	0	0	0	0	0	0	0	0	0	16.67	37.5
MWCNTs	Standard	3	0	19312	6.25	0	0	0	6.25	0	0	0	0	0	0	0	0	0	0	0	0	0	0	0	0	0	0	6.25
MWCNTs	Standard	3	10	19312	0	0	0	0	0	0	0	0	0	0	0	0	0	0	0	0	0	0	0	0	0	0	0	0
MWCNTs	Standard	3	23.2	19312	0	18.75	0	0	6.25	13.33	0	13.33	13.33	13.33	0	13.33	0	0	13.33	0	0	0	6.67	0	0	0	25	25
MWCNTs	Standard	3	50	19312	0	0	0	0	0	0	0	0	0	0	0	0	0	0	0	0	0	0	0	0	0	0	0	0
MWCNTs	Standard	3	75	19312	20	16.67	0	0	20	0	0	8.33	8.33	8.33	0	8.33	0	0	8.33	0	0	0	8.33	0	0	0	25	40
MWCNTs	Standard	3	100	19312	26.67	18.18	0	0	26.67	0	0	0	0	0	0	0	0	0	0	0	0	0	0	0	0	0	18.18	40
Napthalene	Chorion	1	0	18666	8.33	0	0	0	8.33	0	0	0	0	0	0	0	0	0	0	0	0	0	0	0	0	0	0	8.33
Napthalene	Chorion	1	1	18666	8.33	9.09	0	0	8.33	0	0	0	0	0	0	0	0	0	0	0	0	0	0	0	0	0	9.09	16.67
Napthalene	Chorion	1	2.54	18666	0	0	0	0	0	0	0	0	0	0	0	0	0	0	0	0	0	0	0	0	0	0	0	0
Napthalene	Chorion	1	6.45	18666	0	0	0	0	0	0	0	0	0	0	0	0	0	0	0	0	0	0	0	0	0	0	0	0
Napthalene	Chorion	1	16.4	18666	0	0	0	0	0	8.33	0	8.33	0	0	0	8.33	0	0	8.33	0	0	0	0	0	0	0	8.33	8.33
Napthalene	Chorion	1	35	18666	0	0	0	0	0	16.67	0	0	8.33	8.33	0	16.67	0	0	8.33	0	0	0	0	0	0	8.33	16.67	16.67
Napthalene	Chorion	1	74.8	18666	0	0	0	0	0	0	0	0	0	0	0	0	0	0	0	0	0	0	0	0	0	0	0	0
Napthalene	Chorion	1	100	18666	0	0	0	0	0	0	0	0	0	0	0	0	0	0	0	0	0	0	0	0	0	0	0	0
Napthalene	Chorion	2	0	18721	0	0	0	0	0	0	0	0	0	0	0	0	0	0	0	0	0	0	0	0	0	0	0	0
Napthalene	Chorion	2	1	18721	0	0	0	0	0	0	0	0	0	0	0	0	0	0	0	0	0	0	0	0	0	0	0	0
Napthalene	Chorion	2	2.54	18721	0	0	0	0	0	0	8.33	0	0	0	0	0	0	0	0	0	0	0	0	0	0	0	8.33	8.33
Napthalene	Chorion	2	6.45	18721	0	0	0	0	0	0	0	0	0	0	0	0	0	0	0	0	0	0	0	0	0	0	0	0

Test Agent	Treatment	Replicate plate #	Concentration	Plate Barcode	MO24	DP24	SM24	NC24	MORT	YSE_	AXIS	EYE_	SNOU	JAW_	OTIC	PE__	BRAI	SOMI	PFIN	CFIN	PIG_	CIRC	TRUN	SWIM	NC__	TR__	.except.M(	any.effect
Napthalene	Chorion	2	16.4	18721	0	0	0	0	0	0	0	0	0	0	0	0	0	0	0	0	0	0	0	0	0	0	0	0
Napthalene	Chorion	2	35	18721	0	0	0	0	0	0	0	0	0	0	0	0	0	0	0	0	0	0	0	0	0	8.33	8.33	8.33
Napthalene	Chorion	2	74.8	18721	0	0	0	0	0	16.67	0	16.67	16.67	16.67	0	16.67	0	0	16.67	0	0	0	8.33	0	0	16.67	16.67	16.67
Napthalene	Chorion	2	100	18721	0	0	0	0	0	0	0	0	0	0	0	0	0	0	0	0	0	0	0	0	0	0	0	0
Napthalene	Chorion	3	0	18722	0	0	0	0	8.33	9.09	0	9.09	9.09	9.09	0	9.09	0	0	9.09	0	0	0	0	0	0	9.09	8.33	16.67
Napthalene	Chorion	3	1	18722	0	0	0	0	8.33	0	0	0	0	0	0	0	0	0	0	0	0	0	0	0	0	0	0	8.33
Napthalene	Chorion	3	2.54	18722	0	0	0	0	0	8.33	0	8.33	8.33	8.33	0	8.33	0	0	8.33	0	0	0	0	0	0	0	8.33	8.33
Napthalene	Chorion	3	6.45	18722	0	0	0	0	0	8.33	8.33	8.33	8.33	8.33	0	8.33	0	0	8.33	0	0	0	0	0	0	0	8.33	8.33
Napthalene	Chorion	3	16.4	18722	0	0	0	0	8.33	9.09	9.09	9.09	9.09	9.09	0	9.09	0	0	9.09	0	0	0	0	0	0	9.09	8.33	16.67
Napthalene	Chorion	3	35	18722	0	0	0	0	0	0	0	0	0	0	0	0	0	0	0	0	0	0	0	0	0	0	0	0
Napthalene	Chorion	3	74.8	18722	0	0	0	0	0	0	0	0	0	0	0	0	0	0	8.33	0	0	0	0	0	0	0	8.33	8.33
Napthalene	Chorion	3	100	18722	0	0	0	0	0	0	8.33	0	0	0	0	0	0	0	0	0	0	0	0	0	0	8.33	8.33	8.33
Napthalene	DR	1	0	18720	0	0	0	0	0	0	0	0	0	0	0	0	0	0	0	0	0	0	0	0	0	0	0	0
Napthalene	DR	1	1	18720	0	0	0	0	16.67	0	0	0	0	0	0	0	0	0	0	0	0	0	0	0	0	0	0	16.67
Napthalene	DR	1	2.54	18720	0	0	0	0	16.67	0	0	0	0	0	0	0	0	0	0	0	0	0	0	0	0	0	0	16.67
Napthalene	DR	1	6.45	18720	0	0	0	0	8.33	0	0	0	0	0	0	0	0	0	0	0	0	0	0	0	0	0	0	8.33
Napthalene	DR	1	16.4	18720	0	0	0	0	8.33	9.09	0	9.09	9.09	9.09	0	9.09	0	0	9.09	0	0	0	0	0	0	0	8.33	16.67
Napthalene	DR	1	35	18720	0	0	0	0	8.33	9.09	0	9.09	9.09	9.09	0	9.09	0	0	9.09	0	0	0	0	0	0	0	8.33	16.67
Napthalene	DR	1	74.8	18720	0	0	0	0	0	8.33	8.33	8.33	8.33	8.33	0	8.33	0	0	8.33	8.33	0	0	0	0	0	0	16.67	16.67
Napthalene	DR	1	100	18720	0	0	0	0	0	8.33	0	8.33	8.33	8.33	0	8.33	0	0	8.33	0	0	0	0	0	0	0	8.33	8.33
Napthalene	DR	2	0	19005	8.33	0	0	0	8.33	0	0	0	0	0	0	0	0	0	0	0	0	0	0	0	0	0	0	8.33
Napthalene	DR	2	1	19005	0	0	0	0	0	0	0	0	0	0	0	0	0	0	0	0	0	0	0	0	0	0	0	0
Napthalene	DR	2	2.54	19005	0	8.33	0	0	0	8.33	8.33	8.33	8.33	8.33	0	8.33	0	8.33	8.33	8.33	0	0	8.33	0	0	0	8.33	8.33
Napthalene	DR	2	6.45	19005	0	0	0	0	0	0	0	0	0	0	0	0	0	0	0	0	0	0	0	0	0	0	0	0
Napthalene	DR	2	16.4	19005	0	0	0	0	0	0	0	0	0	0	0	0	0	0	0	0	0	0	0	0	0	0	0	0
Napthalene	DR	2	35	19005	0	0	0	0	0	0	0	0	0	0	0	0	0	0	0	0	0	0	0	0	0	0	0	0
Napthalene	DR	2	74.8	19005	0	0	0	0	0	0	0	0	0	0	0	0	0	0	0	0	0	0	0	0	0	0	0	0
Napthalene	DR	2	100	19005	0	0	0	0	0	0	0	0	0	0	0	0	0	0	0	0	0	0	0	0	0	0	0	0
Napthalene	DR	3	0	19009	0	0	0	0	0	0	0	0	0	0	0	0	0	0	0	0	0	0	8.33	0	0	0	8.33	8.33
Napthalene	DR	3	1	19009	8.33	0	0	0	8.33	0	0	0	0	0	0	0	0	0	0	0	0	0	0	0	0	0	0	8.33
Napthalene	DR	3	2.54	19009	8.33	0	0	0	8.33	0	0	0	0	0	0	0	0	0	0	0	0	0	0	0	0	0	0	8.33
Napthalene	DR	3	6.45	19009	16.67	0	0	0	16.67	0	0	0	0	0	0	0	0	0	0	10	0	0	10	0	0	0	10	25
Napthalene	DR	3	16.4	19009	8.33	0	0	0	8.33	0	0	0	0	0	0	0	0	0	0	0	0	0	0	0	0	0	0	8.33
Napthalene	DR	3	35	19009	0	0	0	0	0	0	0	0	0	0	0	0	0	0	0	8.33	0	0	0	0	0	0	8.33	8.33
Napthalene	DR	3	74.8	19009	8.33	0	0	0	8.33	0	0	0	0	0	0	0	0	0	0	0	0	0	0	0	0	0	0	8.33
Napthalene	DR	3	100	19009	0	0	0	0	0	0	0	0	0	0	0	0	0	0	0	0	0	0	0	0	0	0	0	0
Napthalene	LD	1	0	18674	0	8.33	0	0	8.33	0	9.09	0	0	0	0	0	0	0	0	0	0	0	0	0	0	0	16.67	16.67
Napthalene	LD	1	1	18674	8.33	0	0	0	8.33	0	0	0	0	0	0	0	0	0	0	0	0	0	0	0	0	0	0	8.33
Napthalene	LD	1	2.54	18674	0	0	0	0	8.33	0	0	0	0	0	0	0	0	0	0	0	0	0	0	0	0	0	0	8.33
Napthalene	LD	1	6.45	18674	0	0	0	0	0	8.33	16.67	16.67	16.67	16.67	0	16.67	8.33	0	16.67	0	0	0	0	0	0	8.33	16.67	16.67
Napthalene	LD	1	16.4	18674	0	0	0	0	0	0	0	0	0	0	0	0	0	0	0	0	0	0	0	0	0	0	0	0
Napthalene	LD	1	35	18674	8.33	0	0	0	16.67	10	10	10	10	10	0	10	0	0	10	0	0	0	0	0	0	0	9.09	25
Napthalene	LD	1	74.8	18674	0	8.33	0	0	0	8.33	8.33	8.33	8.33	8.33	0	8.33	0	0	8.33	8.33	0	0	8.33	0	0	8.33	8.33	8.33
Napthalene	LD	1	100	18674	0	0	0	0	0	0	0	0	0	0	0	0	0	0	0	0	0	0	0	0	0	0	0	0
Napthalene	LD	2	0	18704	8.33	0	0	0	8.33	0	0	0	0	0	0	0	0	0	0	0	0	0	0	0	0	0	0	8.33
Napthalene	LD	2	1	18704	0	0	0	0	0	0	0	0	0	0	0	0	0	0	0	0	0	0	0	0	0	0	0	0
Napthalene	LD	2	2.54	18704	0	0	0	0	0	0	0	0	0	0	0	0	0	0	0	0	0	0	0	0	0	0	0	0
Napthalene	LD	2	6.45	18704	0	0	0	0	0	0	0	0	0	0	0	0	0	0	8.33	0	0	0	8.33	0	0	0	8.33	8.33
Napthalene	LD	2	16.4	18704	0	0	0	0	0	0	0	0	0	0	0	0	0	0	0	0	0	0	0	0	0	0	0	0
Napthalene	LD	2	35	18704	0	0	0	0	0	8.33	8.33	0	0	0	0	0	0	0	0	0	0	0	8.33	0	0	0	16.67	16.67
Napthalene	LD	2	74.8	18704	0	0	0	0	0	8.33	0	0	0	0	0	8.33	0	0	8.33	0	0	0	0	0	0	0	8.33	8.33
Napthalene	LD	2	100	18704	0	0	0	0	0	0	0	0	0	0	0	0	0	0	0	0	0	0	0	0	0	0	0	0
Napthalene	LD	3	0	18835	8.33	9.09	0	0	8.33	0	9.09	0	0	0	0	0	0	0	0	9.09	0	0	9.09	0	0	0	9.09	16.67
Napthalene	LD	3	1	18835	0	0	0	0	0	0	0	0	0	0	0	0	0	0	0	0	0	0	0	0	0	0	0	0
Napthalene	LD	3	2.54	18835	0	0	0	0	0	0	0	0	0	0	0	0	0	0	0	0	0	0	0	0	0	0	0	0
Napthalene	LD	3	6.45	18835	16.67	0	0	0	16.67	10	10	10	10	10	0	10	0	0	10	10	0	0	0	10	0	0	10	25
Napthalene	LD	3	16.4	18835	8.33	9.09	0	0	25	11.11	0	11.11	11.11	11.11	11.11	11.11	11.11	0	11.11	11.11	0	0	22.22	11.11	0	0	18.18	41.67
Napthalene	LD	3	35	18835	8.33	0	0	0	8.33	0	0	0	0	0	0	0	0	0	0	0	0	0	0	0	0	0	0	8.33
Napthalene	LD	3	74.8	18835	8.33	0	0	0	8.33	0	0	0	0	0	0	0	0	0	0	0	0	0	0	0	0	0	0	8.33
Napthalene	LD	3	100	18835	0	8.33	0	0	0	0	0	0	0	0	0	0	0	0	0	8.33	0	0	8.33	0	0	0	8.33	8.33
Napthalene	Standard	1	0	18667	33.33	0	0	0	33.33	0	0	0	0	0	0	0	0	0	0	0	0	0	0	0	0	0	0	33.33
Napthalene	Standard	1	1	18667	33.33	0	0	0	33.33	0	0	0	0	0	0	0	0	0	0	0	0	0	0	0	0	0	0	33.33
Napthalene	Standard	1	2.54	18667	50	0	0	0	50	0	0	0	0	0	0	0	0	0	0	0	0	0	0	0	0	0	0	50
Napthalene	Standard	1	6.45	18667	33.33	0	0	0	33.33	0	0	0	0	0	0	0	0	0	0	0	0	0	0	0	0	0	0	33.33
Napthalene	Standard	1	16.4	18667	50	16.67	0	0	50	16.67	16.67	16.67	16.67	16.67	0	16.67	0	0	16.67	0	0	0	0	0	0	0	16.67	58.33
Napthalene	Standard	1	35	18667	41.67	14.29	0	0	41.67	0	14.29	0	0	0	0	0	0	0	0	0	0	0	0	0	0	0	14.29	50
Napthalene	Standard	1	74.8	18667	0	8.33	0	0	8.33	0	0	0	0	0	0	0	0	0	0	0	0	0	0	0	0	0	8.33	8.33
Napthalene	Standard	1	100	18667	16.67	0	0	0	16.67	0	0	0	0	0	0	0	0	0	0	0	0	0	0	0	0	0	0	16.67
Napthalene	Standard	2	0	18691	8.33	0	0	0	8.33	0	0	0	0	0	0	0	0	0	0	0	0	0	0	0	0	0	0	8.33
Napthalene	Standard	2	1	18691	0	0	0	0	0	0	0	0	0	0	0	0	0	0	0	0	0	0	0	0	0	0	0	0
Napthalene	Standard	2	2.54	18691	8.33	0	0	0	8.33	0	0	0	0	0	0	0	0	0	0	0	0	0	0	0	0	0	0	8.33
Napthalene	Standard	2	6.45	18691	8.33	0	0	0	8.33	0	0	0	0	0	0	0	0	0	9.09	0	0	0	0	0	0	0	9.09	16.67
Napthalene	Standard	2	16.4	18691	8.33	0	0	0	8.33	0	0	0	0	0	0	0	0	0	0	0	0	0	0	0	0	0	0	8.33
Napthalene	Standard	2	35	18691	0	8.33	0	0	8.33	9.09	9.09	9.09	9.09	9.09	0	9.09	0	0	9.09	0	0	0	0	0	0	0	16.67	16.67
Napthalene	Standard	2	74.8	18691	8.33	0	0	0	8.33	0	0	0	0	0	0	0	0	0	0	0	0	0	0	0	0	0	0	8.33
Napthalene	Standard	2	100	18691	8.33	0	0	0	8.33	0	0	0	0	0	0	0	0	0	0	0	0	0	0	0	0	0	0	8.33
Napthalene	Standard	3	0	18703	8.33	0	0	0	8.33	0	0	0	0	0	0	0	9.09	0	0	0	0	0	0	0	0	0	9.09	16.67
Napthalene	Standard	3	1	18703	16.67	0	0	0	16.67	0	0	0	0	0	0	0	0	0	0	0	0	0	0	0	0	0	0	16.67

Test Agent	Treatment	Replicate plate #	Concentration	Plate Barcode	MO24	DP24	SM24	NC24	MORT	YSE_	AXIS	EYE_	SNOU	JAW_	OTIC	PE__	BRAI	SOMI	PFIN	CFIN	PIG_	CIRC	TRUN	SWIM	NC__	TR__	.except.M	any.effect
Napthalene	Standard	3	2.54	18703	0	0	0	0	0	8.33	8.33	8.33	8.33	8.33	0	8.33	0	0	8.33	0	0	0	0	0	0	0	8.33	8.33
Napthalene	Standard	3	6.45	18703	0	0	0	0	0	0	0	0	0	0	0	0	0	0	0	0	0	0	0	0	0	0	0	0
Napthalene	Standard	3	16.4	18703	8.33	0	0	0	8.33	0	0	0	0	0	0	9.09	0	0	0	0	0	0	0	0	0	0	9.09	16.67
Napthalene	Standard	3	35	18703	0	0	0	0	0	0	0	0	0	0	0	0	0	0	0	0	0	0	0	0	0	0	0	0
Napthalene	Standard	3	74.8	18703	8.33	0	0	0	8.33	9.09	0	0	0	0	0	9.09	0	0	9.09	0	0	0	0	0	0	0	9.09	16.67
Napthalene	Standard	3	100	18703	0	0	0	0	0	0	0	0	0	0	0	0	0	0	0	0	0	0	0	0	0	0	0	0
Permethrin	Chorion	1	0	18714	0	0	0	0	0	8.33	0	8.33	8.33	8.33	0	8.33	0	0	8.33	8.33	0	0	8.33	8.33	0	0	8.33	8.33
Permethrin	Chorion	1	1	18714	0	0	0	0	0	0	0	0	0	0	0	0	0	0	0	0	0	0	0	0	0	0	0	0
Permethrin	Chorion	1	2.54	18714	0	0	0	0	8.33	0	0	0	0	0	0	0	0	0	0	0	0	0	0	0	0	0	0	8.33
Permethrin	Chorion	1	5	18714	0	0	0	0	0	25	16.67	8.33	33.33	33.33	0	16.67	0	0	8.33	16.67	0	0	33.33	0	0	0	41.67	41.67
Permethrin	Chorion	1	8	18714	8.33	0	0	0	16.67	80	30	10	70	70	0	40	10	0	0	20	0	0	70	0	0	0	81.82	91.67
Permethrin	Chorion	1	12	18714	0	0	0	0	0	58.33	50	16.67	75	75	8.33	41.67	8.33	8.33	16.67	16.67	8.33	0	41.67	8.33	8.33	8.33	83.33	83.33
Permethrin	Chorion	1	16.4	18714	0	0	0	0	8.33	63.64	45.45	9.09	63.64	72.73	0	72.73	0	0	27.27	36.36	0	0	63.64	0	0	0	83.33	91.67
Permethrin	Chorion	1	35	18714	0	8.33	0	0	16.67	100	100	10	90	90	0	60	0	0	30	80	0	0	100	0	0	0	91.67	100
Permethrin	Chorion	2	0	18715	0	0	0	0	8.33	9.09	9.09	9.09	9.09	9.09	9.09	9.09	9.09	9.09	9.09	9.09	0	0	9.09	9.09	9.09	9.09	8.33	16.67
Permethrin	Chorion	2	1	18715	0	0	0	0	0	0	0	0	0	0	0	0	0	0	0	0	0	0	0	0	0	0	0	0
Permethrin	Chorion	2	2.54	18715	0	0	0	0	8.33	0	0	9.09	9.09	9.09	0	9.09	0	0	0	0	0	0	9.09	0	0	0	8.33	16.67
Permethrin	Chorion	2	5	18715	8.33	0	0	0	8.33	9.09	9.09	0	9.09	9.09	0	0	0	0	0	0	0	0	9.09	0	0	0	9.09	16.67
Permethrin	Chorion	2	8	18715	0	0	0	0	0	33.33	33.33	33.33	33.33	50	0	25	8.33	0	8.33	41.67	0	0	66.67	8.33	0	0	83.33	83.33
Permethrin	Chorion	2	12	18715	0	0	0	0	0	75	41.67	50	58.33	58.33	0	41.67	16.67	0	25	50	0	0	83.33	16.67	0	0	83.33	83.33
Permethrin	Chorion	2	16.4	18715	0	0	0	0	25	100	33.33	77.78	77.78	77.78	11.11	88.89	22.22	0	33.33	77.78	0	0	100	22.22	0	0	75	100
Permethrin	Chorion	2	35	18715	0	0	0	0	8.33	100	81.82	100	100	100	0	81.82	18.18	0	63.64	72.73	0	0	90.91	18.18	0	0	91.67	100
Permethrin	Chorion	3	0	18716	0	0	0	0	0	8.33	8.33	8.33	8.33	8.33	8.33	8.33	8.33	8.33	8.33	8.33	0	0	8.33	8.33	0	8.33	8.33	8.33
Permethrin	Chorion	3	1	18716	8.33	0	0	0	16.67	0	0	0	0	0	0	0	0	0	0	0	0	0	0	0	0	0	0	16.67
Permethrin	Chorion	3	2.54	18716	0	0	0	0	0	8.33	0	8.33	8.33	8.33	8.33	8.33	8.33	8.33	8.33	8.33	8.33	0	8.33	8.33	0	8.33	8.33	8.33
Permethrin	Chorion	3	5	18716	0	0	0	0	0	25	8.33	16.67	25	25	0	8.33	0	0	8.33	41.67	0	0	41.67	8.33	0	0	41.67	41.67
Permethrin	Chorion	3	8	18716	0	0	0	0	16.67	50	40	20	40	40	0	10	20	0	10	40	0	0	60	0	0	0	58.33	75
Permethrin	Chorion	3	12	18716	0	0	0	0	8.33	72.73	54.55	27.27	54.55	54.55	0	27.27	9.09	0	0	36.36	0	0	81.82	0	0	0	75	83.33
Permethrin	Chorion	3	16.4	18716	0	0	0	0	8.33	54.55	45.45	45.45	63.64	72.73	9.09	63.64	18.18	9.09	27.27	36.36	9.09	0	63.64	9.09	9.09	9.09	75	83.33
Permethrin	Chorion	3	35	18716	0	0	0	0	33.33	100	87.5	50	100	100	0	62.5	12.5	0	25	62.5	0	0	100	0	0	0	66.67	100
Permethrin	DR	1	0	18708	0	0	0	0	0	0	0	0	0	0	0	0	0	0	0	0	0	0	0	0	0	0	0	0
Permethrin	DR	1	1	18708	0	8.33	0	0	0	16.67	16.67	8.33	16.67	16.67	8.33	8.33	8.33	8.33	8.33	8.33	0	0	16.67	8.33	0	8.33	16.67	16.67
Permethrin	DR	1	2.54	18708	8.33	0	0	0	16.67	70	40	10	80	80	0	40	0	0	20	40	0	0	80	10	0	0	72.73	83.33
Permethrin	DR	1	5	18708	8.33	9.09	0	0	25	77.78	55.56	22.22	77.78	77.78	0	66.67	0	0	33.33	44.44	0	0	77.78	0	0	0	81.82	91.67
Permethrin	DR	1	8	18708	8.33	9.09	0	0	41.67	85.71	57.14	28.57	85.71	85.71	28.57	71.43	42.86	0	42.86	57.14	0	0	100	14.29	0	0	63.64	100
Permethrin	DR	1	12	18708	33.33	0	0	0	33.33	100	37.5	50	100	100	12.5	62.5	25	0	37.5	75	0	0	75	0	0	0	100	100
Permethrin	DR	1	16.4	18708	8.33	9.09	0	0	16.67	90	80	30	80	90	10	60	10	10	50	50	0	0	90	0	0	10	90.91	100
Permethrin	DR	1	35	18708	8.33	18.18	0	0	8.33	90.91	63.64	54.55	81.82	81.82	9.09	54.55	27.27	9.09	63.64	63.64	9.09	0	90.91	27.27	0	27.27	100	100
Permethrin	DR	2	0	18726	0	0	0	0	25	0	0	0	0	0	0	0	0	0	0	0	0	0	0	0	0	0	0	25
Permethrin	DR	2	1	18726	0	0	0	0	16.67	0	0	0	0	0	0	0	0	0	10	0	0	0	0	0	0	0	8.33	25
Permethrin	DR	2	2.54	18726	0	0	0	0	30	57.14	71.43	28.57	71.43	71.43	0	57.14	0	0	28.57	42.86	0	0	85.71	0	0	0	60	90
Permethrin	DR	2	5	18726	0	9.09	0	0	36.36	57.14	42.86	14.29	42.86	42.86	0	28.57	0	0	14.29	0	0	0	57.14	0	0	0	54.55	81.82
Permethrin	DR	2	8	18726	8.33	9.09	0	0	33.33	62.5	100	50	87.5	87.5	12.5	50	12.5	12.5	62.5	37.5	12.5	0	87.5	12.5	0	0	72.73	100
Permethrin	DR	2	12	18726	0	0	0	0	41.67	85.71	85.71	28.57	71.43	85.71	14.29	57.14	28.57	14.29	42.86	57.14	0	0	71.43	14.29	0	0	58.33	100
Permethrin	DR	2	16.4	18726	0	8.33	0	8.33	16.67	70	80	30	70	70	0	40	10	10	40	40	0	0	80	20	0	10	75	91.67
Permethrin	DR	2	35	18726	0	0	0	0	25	88.89	55.56	44.44	77.78	77.78	0	33.33	0	11.11	22.22	33.33	0	0	77.78	11.11	0	0	75	100
Permethrin	DR	3	0	18727	0	0	0	0	16.67	0	0	0	0	0	0	0	0	0	0	0	0	0	0	0	0	0	0	16.67
Permethrin	DR	3	1	18727	0	0	0	0	16.67	10	0	10	10	10	0	10	0	0	10	0	0	0	0	0	0	0	8.33	25
Permethrin	DR	3	2.54	18727	0	0	0	0	8.33	45.45	36.36	27.27	54.55	54.55	0	36.36	9.09	0	18.18	36.36	0	0	54.55	0	0	0	58.33	66.67
Permethrin	DR	3	5	18727	8.33	0	0	0	16.67	50	40	10	60	60	0	20	10	0	20	30	0	0	60	0	0	0	72.73	83.33
Permethrin	DR	3	8	18727	8.33	9.09	0	0	33.33	62.5	87.5	37.5	87.5	87.5	12.5	62.5	12.5	12.5	25	37.5	12.5	0	75	12.5	0	12.5	63.64	91.67
Permethrin	DR	3	12	18727	0	0	0	0	33.33	75	50	12.5	75	75	0	50	0	0	37.5	25	0	0	62.5	0	0	0	58.33	91.67
Permethrin	DR	3	16.4	18727	8.33	0	0	0	41.67	85.71	71.43	57.14	100	85.71	0	57.14	28.57	0	57.14	85.71	0	0	100	28.57	0	14.29	63.64	100
Permethrin	DR	3	35	18727	0	0	0	0	33.33	87.5	37.5	75	75	75	12.5	62.5	25	0	37.5	50	0	0	62.5	25	0	12.5	58.33	91.67
Permethrin	LD	1	0	18710	0	0	0	0	0	0	0	0	0	0	0	0	0	0	0	0	0	0	0	0	0	0	0	0
Permethrin	LD	1	1	18710	0	0	0	0	0	0	0	0	0	0	0	0	0	0	0	0	0	0	0	0	0	0	0	0
Permethrin	LD	1	2.54	18710	8.33	18.18	0	0	16.67	10	0	0	0	0	0	10	0	0	10	0	0	0	0	0	0	0	18.18	33.33
Permethrin	LD	1	5	18710	0	0	0	0	8.33	0	0	0	0	0	0	0	0	0	0	0	0	0	0	0	0	0	0	8.33
Permethrin	LD	1	8	18710	0	0	0	0	0	0	0	0	8.33	8.33	0	0	8.33	0	0	0	0	0	8.33	0	0	0	16.67	16.67
Permethrin	LD	1	12	18710	16.67	10	0	0	25	33.33	22.22	11.11	11.11	22.22	0	22.22	33.33	0	33.33	0	0	0	0	0	0	33.33	60	75
Permethrin	LD	1	16.4	18710	0	0	0	0	0	8.33	25	33.33	41.67	41.67	0	25	25	0	8.33	0	0	0	16.67	0	0	50	75	75
Permethrin	LD	1	35	18710	25	0	0	0	50	83.33	83.33	66.67	83.33	83.33	0	50	83.33	0	83.33	0	0	0	0	0	0	66.67	66.67	100
Permethrin	LD	2	0	18713	0	8.33	0	0	0	8.33	0	8.33	8.33	8.33	0	8.33	0	0	8.33	0	0	0	0	0	0	0	8.33	8.33
Permethrin	LD	2	1	18713	16.67	0	0	0	16.67	0	0	0	0	0	0	0	0	0	0	0	0	0	0	0	0	0	0	16.67
Permethrin	LD	2	2.54	18713	8.33	9.09	0	0	16.67	0	0	0	0	0	0	0	0	0	0	0	0	0	0	0	0	0	9.09	16.67
Permethrin	LD	2	5	18713	8.33	0	0	0	16.67	10	0	20	20	20	0	10	0	0	20	0	0	0	0	0	0	0	18.18	33.33
Permethrin	LD	2	8	18713	25	0	0	0	25	0	0	11.11	11.11	11.11	0	0	0	0	0	0	0	0	0	0	0	11.11	11.11	33.33
Permethrin	LD	2	12	18713	16.67	10	0	0	25	33.33	0	33.33	33.33	33.33	0	22.22	22.22	0	33.33	0	0	0	11.11	0	0	0	50	58.33
Permethrin	LD	2	16.4	18713	25	0	0	0	41.67	14.29	28.57	71.43	71.43	71.43	0	28.57	28.57	0	28.57	0	0	0	14.29	0	0	28.57	66.67	91.67
Permethrin	LD	2	35	18713	8.33	0	0	0	41.67	71.43	42.86	100	100	100	0	71.43	57.14	0	71.43	14.29	0	0	14.29	0	0	42.86	63.64	100
Permethrin	LD	3	0	18719	0	0	0	0	25	0	0	0	0	0	0	0	0	0	0	0	0	0	0	0	0	0	0	25
Permethrin	LD	3	1	18719	8.33	0	0	0	33.33	0	25	0	25	25	0	0	0	0	0	0	0	0	0	0	0	0	18.18	50
Permethrin	LD	3	2.54	18719	0	0	0	0	16.67	0	0	0	20	10	0	0	0	0	0	0	0	0	0	0	0	0	16.67	33.33
Permethrin	LD	3	5	18719	0	8.33	0	0	16.67	0	0	0	10	10	0	0	0	0	0	10	0	0	10	0	0	0	16.67	33.33
Permethrin	LD	3	8	18719	16.67	10	0	0	25	11.11	0	0	0	0	0	0	0	0	11.11	0	0	0	0	0	0	0	20	41.67
Permethrin	LD	3	12	18719	8.33	0	0	0	25	0	0	0	0	0	0	0	0	0	0	0	0	0	0	0	0	0	0	25
Permethrin	LD	3	16.4	18719	16.67	0	0	0	25	0	0	0	0	0	0	0	0	0	0	0	0	0	0	0	0	11.11	10	33.33
Permethrin	LD	3	35	18719	16.67	10	0	0	58.33	60	60	40	40	40	20	40	80	0	60	20	0	0	20	0	0	60	50	100

Test Agent	Treatment	Replicate plate #	Concentration	Plate Barcode	MO24	DP24	SM24	NC24	MORT	YSE_	AXIS	EYE_	SNOU	JAW_	OTIC	PE__	BRAI	SOMI	PFIN	CFIN	PIG_	CIRC	TRUN	SWIM	NC__	TR__	.except.M[illegible]	any.effect
Permethrin	Standard	1	0	18709	8.33	0	0	0	8.33	0	0	0	0	0	0	0	0	0	0	0	0	0	0	0	0	0	0	8.33
Permethrin	Standard	1	1	18709	16.67	10	0	0	25	0	11.11	0	0	0	0	0	0	0	0	11.11	0	0	11.11	0	0	0	10	33.33
Permethrin	Standard	1	2.54	18709	8.33	9.09	0	0	16.67	0	0	0	0	0	0	0	0	0	0	0	0	0	0	0	0	0	9.09	16.67
Permethrin	Standard	1	5	18709	33.33	0	0	0	33.33	12.5	25	25	25	25	0	25	12.5	0	25	12.5	0	0	0	0	0	12.5	50	66.67
Permethrin	Standard	1	8	18709	33.33	0	0	0	50	0	0	0	16.67	16.67	0	0	0	0	0	0	0	0	0	0	0	0	12.5	58.33
Permethrin	Standard	1	12	18709	8.33	0	0	0	8.33	0	27.27	0	27.27	36.36	0	9.09	9.09	0	0	9.09	0	0	18.18	0	0	27.27	63.64	66.67
Permethrin	Standard	1	16.4	18709	16.67	20	0	0	25	0	33.33	0	11.11	11.11	0	0	0	0	0	11.11	0	0	0	0	0	0	60	66.67
Permethrin	Standard	1	35	18709	8.33	0	0	0	16.67	10	70	0	50	50	0	30	20	0	10	0	0	0	10	0	0	10	81.82	91.67
Permethrin	Standard	2	0	18718	8.33	9.09	0	0	8.33	9.09	9.09	9.09	9.09	9.09	0	9.09	0	0	9.09	0	0	0	0	0	0	0	9.09	16.67
Permethrin	Standard	2	1	18718	33.33	12.5	0	0	33.33	12.5	12.5	12.5	12.5	12.5	0	12.5	0	0	12.5	0	0	0	12.5	0	0	0	12.5	41.67
Permethrin	Standard	2	2.54	18718	25	0	0	0	25	0	0	0	0	0	0	0	0	0	0	0	0	0	0	0	0	0	0	25
Permethrin	Standard	2	5	18718	16.67	10	0	0	16.67	0	10	10	0	0	0	0	0	0	0	0	0	0	10	0	0	0	20	33.33
Permethrin	Standard	2	8	18718	0	0	0	0	0	8.33	16.67	8.33	8.33	8.33	0	8.33	8.33	0	8.33	0	0	0	0	0	0	0	16.67	16.67
Permethrin	Standard	2	12	18718	8.33	9.09	0	0	8.33	9.09	27.27	9.09	9.09	27.27	0	9.09	63.64	0	9.09	0	0	0	0	0	0	9.09	81.82	83.33
Permethrin	Standard	2	16.4	18718	8.33	9.09	0	0	16.67	10	70	10	20	30	0	30	40	0	20	10	0	0	0	0	0	10	81.82	83.33
Permethrin	Standard	2	35	18718	16.67	10	0	0	25	55.56	100	22.22	55.56	55.56	0	44.44	88.89	0	66.67	11.11	0	0	11.11	0	0	11.11	90	100
Permethrin	Standard	3	0	18724	16.67	10	0	0	25	0	0	0	0	0	0	0	0	0	0	0	0	0	0	0	0	0	10	25
Permethrin	Standard	3	1	18724	0	0	0	0	0	8.33	8.33	8.33	8.33	8.33	0	8.33	0	0	8.33	0	0	0	8.33	0	0	0	16.67	16.67
Permethrin	Standard	3	2.54	18724	33.33	0	0	0	33.33	0	0	0	0	0	0	0	0	0	0	0	0	0	12.5	0	0	0	12.5	41.67
Permethrin	Standard	3	5	18724	0	0	0	0	0	0	8.33	0	0	0	0	8.33	0	0	0	0	0	0	0	0	0	0	8.33	8.33
Permethrin	Standard	3	8	18724	16.67	0	0	0	16.67	0	40	0	0	0	0	0	0	0	0	0	0	0	0	0	0	0	40	50
Permethrin	Standard	3	12	18724	0	0	0	0	16.67	20	90	10	10	10	0	40	60	0	30	0	0	0	0	0	0	0	75	91.67
Permethrin	Standard	3	16.4	18724	16.67	20	0	0	33.33	12.5	50	25	12.5	37.5	0	12.5	50	0	25	12.5	0	0	12.5	0	0	25	60	75
Permethrin	Standard	3	35	18724	16.67	10	0	0	33.33	12.5	87.5	25	50	62.5	0	12.5	75	0	25	0	0	0	0	0	0	12.5	90	100
Pyrene	Chorion	1	0	18792	0	0	0	0	0	8.33	0	8.33	8.33	8.33	0	8.33	8.33	0	8.33	8.33	0	0	8.33	8.33	0	8.33	8.33	8.33
Pyrene	Chorion	1	1	18792	0	0	0	0	0	0	0	0	0	0	0	0	0	0	0	0	0	0	0	0	0	0	0	0
Pyrene	Chorion	1	5	18792	0	0	0	0	0	0	0	0	0	0	0	0	0	0	0	0	0	0	0	0	0	0	0	0
Pyrene	Chorion	1	16.5	18792	0	0	0	0	0	0	0	0	0	0	0	0	0	0	0	0	0	0	0	0	0	0	0	0
Pyrene	Chorion	1	30	18792	0	0	0	0	0	0	0	0	0	0	0	0	0	0	0	0	0	0	0	0	0	0	0	0
Pyrene	Chorion	1	50	18792	0	0	0	0	0	0	0	0	0	0	0	0	0	0	0	0	0	0	0	0	0	0	0	0
Pyrene	Chorion	1	65	18792	0	0	0	0	41.67	0	0	0	0	42.86	0	14.29	0	0	0	0	0	0	0	0	0	0	25	66.67
Pyrene	Chorion	1	100	18792	0	0	0	0	58.33	20	0	0	0	60	0	40	0	0	0	0	0	0	0	0	0	0	33.33	91.67
Pyrene	Chorion	2	0	18793	0	0	0	0	0	0	0	0	0	0	0	0	0	0	0	0	0	0	0	0	0	0	0	0
Pyrene	Chorion	2	1	18793	8.33	0	0	0	8.33	0	0	0	0	0	0	0	0	0	0	0	0	0	0	0	0	0	0	8.33
Pyrene	Chorion	2	5	18793	0	0	0	0	0	8.33	8.33	8.33	8.33	8.33	0	8.33	8.33	0	8.33	8.33	0	0	8.33	8.33	0	8.33	8.33	8.33
Pyrene	Chorion	2	16.5	18793	0	0	0	0	0	16.67	16.67	16.67	16.67	16.67	0	16.67	8.33	8.33	16.67	16.67	0	0	16.67	16.67	0	16.67	16.67	16.67
Pyrene	Chorion	2	30	18793	0	0	0	0	0	0	0	0	0	0	0	0	0	0	0	0	0	0	0	0	0	0	0	0
Pyrene	Chorion	2	50	18793	0	0	0	0	33.33	0	0	0	0	0	0	0	0	0	0	0	0	0	0	0	0	0	0	33.33
Pyrene	Chorion	2	65	18793	0	0	0	0	25	0	11.11	0	11.11	22.22	0	33.33	0	0	0	0	0	0	0	0	0	0	25	50
Pyrene	Chorion	2	100	18793	0	0	0	0	50	33.33	0	33.33	50	83.33	0	83.33	0	0	33.33	0	0	0	33.33	0	0	0	41.67	91.67
Pyrene	Chorion	3	0	18794	0	0	0	0	0	0	0	0	0	0	0	0	0	0	0	0	0	0	0	0	0	0	0	0
Pyrene	Chorion	3	1	18794	0	0	0	0	8.33	0	0	0	0	0	0	0	0	0	0	0	0	0	0	0	0	0	0	8.33
Pyrene	Chorion	3	5	18794	0	0	0	0	0	0	0	0	0	8.33	0	8.33	0	0	0	0	0	0	0	0	0	0	8.33	8.33
Pyrene	Chorion	3	16.5	18794	0	0	0	0	8.33	0	0	0	0	0	0	0	0	0	0	0	0	0	0	0	0	0	0	8.33
Pyrene	Chorion	3	30	18794	0	0	0	0	0	0	0	0	0	0	0	0	0	0	0	0	0	0	0	0	0	0	0	0
Pyrene	Chorion	3	50	18794	0	0	0	0	8.33	0	0	0	0	0	0	0	0	0	0	0	0	0	0	0	0	0	0	8.33
Pyrene	Chorion	3	65	18794	8.33	0	0	0	33.33	50	0	12.5	37.5	50	0	37.5	0	0	25	0	0	0	12.5	0	0	0	45.45	75
Pyrene	Chorion	3	100	18794	0	0	0	0	25	11.11	0	0	11.11	77.78	0	55.56	11.11	0	0	0	0	0	0	0	0	0	58.33	83.33
Pyrene	DR	1	0	18795	0	0	0	0	0	0	0	0	0	0	0	0	0	0	0	0	0	0	0	0	0	0	0	0
Pyrene	DR	1	1	18795	0	0	0	0	0	8.33	0	8.33	8.33	8.33	0	8.33	0	0	0	0	0	0	0	0	0	0	8.33	8.33
Pyrene	DR	1	5	18795	0	0	0	0	0	0	0	0	0	0	0	0	0	0	0	0	0	0	0	0	0	0	0	0
Pyrene	DR	1	16.5	18795	0	8.33	0	0	41.67	42.86	28.57	42.86	71.43	71.43	0	42.86	28.57	0	57.14	0	0	0	14.29	0	0	28.57	58.33	91.67
Pyrene	DR	1	30	18795	0	8.33	0	0	50	66.67	16.67	50	100	100	0	50	33.33	0	83.33	0	0	0	0	0	0	66.67	58.33	100
Pyrene	DR	1	50	18795	0	8.33	0	0	25	66.67	22.22	77.78	88.89	88.89	0	66.67	33.33	0	88.89	11.11	0	0	11.11	0	0	66.67	66.67	91.67
Pyrene	DR	1	65	18795	0	25	0	0	25	66.67	33.33	55.56	77.78	77.78	0	66.67	22.22	0	66.67	0	0	0	22.22	0	0	33.33	75	91.67
Pyrene	DR	1	100	18795	16.67	10	0	0	58.33	40	80	40	80	80	0	40	20	0	40	0	0	0	0	0	0	40	50	100
Pyrene	DR	2	0	18796	41.67	14.29	0	0	41.67	0	0	0	0	0	0	0	0	0	0	14.29	0	0	0	0	0	0	14.29	50
Pyrene	DR	2	1	18796	0	0	0	0	0	8.33	8.33	8.33	8.33	8.33	0	8.33	0	0	8.33	0	0	0	0	0	0	0	8.33	8.33
Pyrene	DR	2	5	18796	0	0	0	0	0	9.09	9.09	9.09	9.09	9.09	0	9.09	0	0	9.09	9.09	0	0	0	0	0	0	9.09	9.09
Pyrene	DR	2	16.5	18796	8.33	9.09	0	0	58.33	20	0	20	60	60	0	20	0	0	20	0	0	0	0	0	0	0	36.36	91.67
Pyrene	DR	2	30	18796	0	0	0	0	50	20	40	20	80	80	0	80	0	0	20	0	0	0	0	0	0	20	50	100
Pyrene	DR	2	50	18796	0	0	0	0	30	28.57	0	28.57	100	100	0	42.86	0	0	57.14	0	0	0	0	0	0	28.57	70	100
Pyrene	DR	2	65	18796	0	0	0	0	45.45	33.33	33.33	50	83.33	83.33	0	33.33	16.67	0	66.67	0	0	0	0	0	0	33.33	54.55	100
Pyrene	DR	2	100	18796	0	0	0	0	25	44.44	33.33	44.44	77.78	77.78	0	55.56	22.22	0	77.78	0	0	0	0	0	0	44.44	75	100
Pyrene	DR	3	0	18806	0	0	0	0	0	0	0	0	0	0	0	0	0	0	0	0	0	0	0	0	0	0	0	0
Pyrene	DR	3	1	18806	8.33	0	0	0	8.33	0	0	0	0	0	0	0	0	0	0	0	0	0	0	0	0	0	0	8.33
Pyrene	DR	3	5	18806	16.67	0	0	0	16.67	0	0	0	0	0	0	0	0	0	0	0	0	0	0	0	0	0	0	16.67
Pyrene	DR	3	16.5	18806	9.09	0	0	0	81.82	0	50	0	50	50	0	0	0	0	0	0	0	0	0	0	0	0	20	100
Pyrene	DR	3	30	18806	0	8.33	0	0	50	33.33	16.67	33.33	100	100	0	33.33	16.67	0	66.67	0	0	0	0	0	0	33.33	50	100
Pyrene	DR	3	50	18806	0	0	0	0	50	16.67	0	16.67	100	100	0	16.67	16.67	0	33.33	0	0	0	16.67	0	0	16.67	50	100
Pyrene	DR	3	65	18806	0	0	0	0	41.67	28.57	14.29	28.57	85.71	85.71	0	28.57	28.57	0	57.14	0	0	0	14.29	0	0	28.57	58.33	100
Pyrene	DR	3	100	18806	0	0	0	0	50	50	50	33.33	100	100	0	66.67	0	0	66.67	0	0	0	16.67	0	0	16.67	50	100
Pyrene	LD	1	0	18819	0	0	0	0	8.33	0	0	0	0	0	0	0	0	0	0	0	0	0	0	0	0	0	0	8.33
Pyrene	LD	1	1	18819	16.67	0	0	0	16.67	0	0	0	0	0	0	0	0	0	0	0	0	0	0	0	0	0	0	16.67
Pyrene	LD	1	5	18819	0	0	0	0	0	0	0	0	0	0	0	0	0	0	0	0	0	0	0	0	0	0	0	0
Pyrene	LD	1	16.5	18819	8.33	0	0	0	8.33	0	0	0	0	0	0	0	0	0	0	0	0	0	0	0	0	0	0	8.33
Pyrene	LD	1	30	18819	8.33	0	0	0	8.33	9.09	9.09	9.09	9.09	9.09	9.09	9.09	9.09	0	9.09	9.09	0	0	9.09	9.09	0	9.09	9.09	16.67
Pyrene	LD	1	50	18819	0	0	0	0	8.33	9.09	18.18	9.09	9.09	27.27	0	18.18	9.09	0	9.09	27.27	0	0	27.27	9.09	0	9.09	41.67	50

Test Agent	Treatment	Replicate plate #	Concentration	Plate Barcode	MO24	DP24	SM24	NC24	MORT	YSE_	AXIS	EYE_	SNOU	JAW_	OTIC	PE__	BRAI	SOMI	PFIN	CFIN	PIG_	CIRC	TRUN	SWIM	NC__	TR__	.except.M(	any.effect
Pyrene	LD	1	65	18819	0	0	0	0	8.33	18.18	9.09	27.27	27.27	54.55	0	54.55	0	0	9.09	9.09	0	0	18.18	9.09	0	9.09	58.33	66.67
Pyrene	LD	1	100	18819	0	0	0	0	41.67	57.14	28.57	42.86	57.14	100	0	85.71	28.57	14.29	57.14	42.86	0	0	57.14	28.57	0	28.57	58.33	100
Pyrene	LD	2	0	18820	8.33	0	0	0	8.33	0	0	0	0	0	0	0	0	0	0	0	0	0	0	0	0	0	0	8.33
Pyrene	LD	2	1	18820	0	0	0	0	0	0	0	0	0	0	0	0	0	0	0	0	0	0	0	0	0	0	0	0
Pyrene	LD	2	5	18820	0	0	0	0	0	0	0	0	0	0	0	0	0	0	0	0	0	0	0	0	0	0	0	0
Pyrene	LD	2	16.5	18820	0	0	0	0	0	0	0	0	0	0	0	0	0	0	0	0	0	0	0	0	0	0	0	0
Pyrene	LD	2	30	18820	0	0	0	0	0	8.33	8.33	8.33	8.33	8.33	0	8.33	8.33	0	8.33	8.33	0	0	8.33	8.33	0	8.33	8.33	8.33
Pyrene	LD	2	50	18820	0	0	0	0	16.67	0	0	10	10	10	0	10	0	0	0	0	0	0	0	0	0	0	8.33	25
Pyrene	LD	2	65	18820	8.33	9.09	0	0	33.33	37.5	25	50	75	75	12.5	75	12.5	0	50	37.5	0	0	37.5	25	0	37.5	54.55	83.33
Pyrene	LD	2	100	18820	0	0	0	0	33.33	50	25	37.5	62.5	100	0	100	0	0	37.5	12.5	0	0	37.5	12.5	0	37.5	66.67	100
Pyrene	LD	3	0	19202	8.33	0	0	0	16.67	0	0	0	0	0	0	0	0	0	0	0	0	0	0	0	0	0	0	16.67
Pyrene	LD	3	1	19202	16.67	0	0	0	16.67	10	0	0	10	10	0	10	0	0	10	0	0	0	0	0	0	0	10	25
Pyrene	LD	3	5	19202	0	0	0	0	0	0	0	0	0	0	0	0	0	0	0	0	0	0	0	0	0	0	0	0
Pyrene	LD	3	16.5	19202	8.33	0	0	0	25	0	0	0	0	0	0	0	0	0	0	0	0	0	0	0	0	0	0	25
Pyrene	LD	3	30	19202	8.33	0	0	0	25	0	0	0	0	0	0	0	0	0	0	0	0	0	11.11	0	0	0	9.09	33.33
Pyrene	LD	3	50	19202	0	16.67	0	0	25	22.22	0	22.22	11.11	11.11	0	11.11	0	0	11.11	11.11	0	0	11.11	0	0	11.11	33.33	50
Pyrene	LD	3	65	19202	8.33	18.18	0	0	33.33	25	0	25	37.5	37.5	0	37.5	0	0	12.5	0	0	0	25	0	0	0	54.55	75
Pyrene	LD	3	100	19202	0	25	0	0	41.67	57.14	14.29	57.14	85.71	85.71	0	71.43	0	0	57.14	14.29	0	0	71.43	0	0	28.57	66.67	100
Pyrene	Standard	1	0	18808	0	0	0	0	8.33	0	0	0	0	0	0	0	0	0	0	0	0	0	0	0	0	0	0	8.33
Pyrene	Standard	1	1	18808	0	0	0	0	0	0	0	0	0	0	0	0	0	0	0	0	0	0	0	0	0	0	0	0
Pyrene	Standard	1	5	18808	0	0	0	0	0	0	0	0	0	0	0	0	0	0	0	0	0	0	0	0	0	0	0	0
Pyrene	Standard	1	16.5	18808	0	0	0	0	0	8.33	0	0	8.33	8.33	0	8.33	0	0	8.33	8.33	0	0	0	0	0	0	16.67	16.67
Pyrene	Standard	1	30	18808	0	0	0	0	8.33	0	9.09	0	0	0	0	0	0	0	0	0	0	0	0	0	0	0	8.33	16.67
Pyrene	Standard	1	50	18808	0	0	0	0	16.67	0	20	0	30	30	0	0	0	0	10	0	0	0	0	0	0	0	41.67	58.33
Pyrene	Standard	1	65	18808	0	0	0	0	50	16.67	33.33	16.67	83.33	83.33	0	16.67	0	0	16.67	0	0	0	0	0	0	0	41.67	91.67
Pyrene	Standard	1	100	18808	8.33	9.09	0	0	75	33.33	0	33.33	100	100	0	33.33	33.33	0	33.33	0	0	0	0	0	0	0	36.36	100
Pyrene	Standard	2	0	18814	0	0	0	0	0	0	0	0	0	0	0	0	0	0	0	0	0	0	0	0	0	0	0	0
Pyrene	Standard	2	1	18814	0	0	0	0	0	8.33	8.33	8.33	8.33	8.33	0	8.33	0	0	8.33	0	0	0	0	0	0	0	8.33	8.33
Pyrene	Standard	2	5	18814	8.33	0	0	0	8.33	0	0	0	0	0	0	0	0	0	0	0	0	0	0	0	0	0	0	8.33
Pyrene	Standard	2	16.5	18814	0	8.33	0	0	16.67	0	0	0	0	0	0	0	0	0	0	0	0	0	0	0	0	0	8.33	16.67
Pyrene	Standard	2	30	18814	0	8.33	0	0	8.33	0	0	0	9.09	9.09	0	0	9.09	0	0	0	0	0	0	0	0	9.09	16.67	16.67
Pyrene	Standard	2	50	18814	16.67	10	0	0	33.33	0	0	0	25	25	0	0	0	0	0	0	0	0	0	0	0	0	20	50
Pyrene	Standard	2	65	18814	0	0	0	0	50	33.33	33.33	33.33	50	50	0	33.33	0	0	33.33	0	0	0	16.67	0	0	0	25	75
Pyrene	Standard	2	100	18814	8.33	9.09	0	0	25	33.33	33.33	44.44	88.89	88.89	0	33.33	0	0	55.56	11.11	0	0	0	0	0	22.22	72.73	91.67
Pyrene	Standard	3	0	18816	8.33	0	0	0	8.33	0	0	0	0	0	0	0	0	0	0	0	0	0	0	0	0	0	0	8.33
Pyrene	Standard	3	1	18816	0	0	0	0	0	0	0	0	0	0	0	0	0	0	0	0	0	0	0	0	0	0	0	0
Pyrene	Standard	3	5	18816	0	0	0	0	0	0	0	0	0	0	0	0	0	0	0	0	0	0	0	0	0	0	0	0
Pyrene	Standard	3	16.5	18816	0	8.33	0	0	8.33	0	9.09	0	0	0	0	0	0	0	0	9.09	0	0	0	0	0	0	25	25
Pyrene	Standard	3	30	18816	8.33	0	0	0	8.33	0	0	0	0	0	0	0	0	0	0	0	0	0	0	0	0	0	0	8.33
Pyrene	Standard	3	50	18816	0	0	0	0	0	8.33	0	16.67	33.33	33.33	0	8.33	0	8.33	16.67	0	0	0	0	0	8.33	0	50	50
Pyrene	Standard	3	65	18816	8.33	0	0	0	16.67	10	20	10	60	60	0	10	0	0	20	0	0	0	10	0	0	0	54.55	66.67
Pyrene	Standard	3	100	18816	0	0	0	0	50	0	33.33	16.67	16.67	16.67	0	0	16.67	0	16.67	0	0	0	33.33	0	0	33.33	41.67	91.67
Retene	Chorion	1	0	18844	0	0	0	0	0	0	0	0	0	0	0	0	0	0	0	0	0	0	0	0	0	0	0	0
Retene	Chorion	1	1	18844	0	0	0	0	0	0	0	0	0	0	0	0	0	0	0	0	0	0	0	0	0	0	0	0
Retene	Chorion	1	5	18844	0	0	0	0	0	0	0	0	0	0	0	0	0	0	0	0	0	0	0	0	0	0	0	0
Retene	Chorion	1	20	18844	0	0	0	0	0	0	0	0	0	0	0	0	0	0	0	0	0	0	0	0	0	0	0	0
Retene	Chorion	1	30	18844	0	0	0	0	0	16.67	8.33	8.33	25	25	0	8.33	0	0	16.67	0	0	0	0	0	0	8.33	25	25
Retene	Chorion	1	45	18844	0	0	0	0	0	41.67	8.33	50	66.67	75	0	50	0	0	58.33	0	0	0	0	0	0	8.33	83.33	83.33
Retene	Chorion	1	65	18844	0	0	0	0	8.33	72.73	0	63.64	81.82	81.82	0	54.55	0	0	81.82	0	0	0	0	0	0	9.09	75	83.33
Retene	Chorion	1	100	18844	0	0	0	0	0	100	16.67	100	100	100	0	91.67	0	0	100	0	0	0	0	0	0	0	100	100
Retene	Chorion	2	0	18851	8.33	0	0	0	25	0	0	0	0	0	0	0	0	0	0	0	0	0	0	0	0	0	0	25
Retene	Chorion	2	1	18851	0	0	0	0	8.33	0	0	0	0	0	0	0	0	0	0	0	0	0	0	0	0	0	0	8.33
Retene	Chorion	2	5	18851	0	0	0	0	8.33	0	0	0	0	0	0	0	0	0	0	0	0	0	0	0	0	0	0	8.33
Retene	Chorion	2	20	18851	0	0	0	0	0	16.67	8.33	16.67	16.67	16.67	0	16.67	0	0	16.67	0	0	0	0	0	0	8.33	16.67	16.67
Retene	Chorion	2	30	18851	0	0	0	0	8.33	9.09	0	9.09	9.09	9.09	0	9.09	0	0	9.09	0	0	0	0	0	0	0	8.33	16.67
Retene	Chorion	2	45	18851	0	0	0	0	0	66.67	8.33	58.33	66.67	66.67	0	66.67	0	0	75	0	0	25	0	0	0	0	75	75
Retene	Chorion	2	65	18851	0	0	0	0	0	66.67	8.33	58.33	83.33	83.33	0	75	0	0	75	0	0	41.67	0	0	0	8.33	83.33	83.33
Retene	Chorion	2	100	18851	0	0	0	0	0	100	18.18	100	100	100	0	100	0	0	100	0	0	54.55	0	0	0	0	100	100
Retene	Chorion	3	0	18883	0	0	0	0	0	8.33	0	8.33	8.33	8.33	0	0	0	0	8.33	0	0	8.33	8.33	0	0	0	8.33	8.33
Retene	Chorion	3	1	18883	0	0	0	0	0	8.33	0	8.33	8.33	8.33	0	8.33	0	0	8.33	0	0	0	0	0	0	0	8.33	8.33
Retene	Chorion	3	5	18883	0	0	0	0	8.33	0	0	0	0	0	0	0	0	0	0	0	0	0	0	0	0	0	0	8.33
Retene	Chorion	3	20	18883	0	0	0	0	8.33	18.18	0	18.18	18.18	18.18	0	9.09	0	0	18.18	0	0	0	0	0	0	0	16.67	25
Retene	Chorion	3	30	18883	0	0	0	0	0	33.33	0	33.33	33.33	33.33	0	33.33	0	0	33.33	0	0	16.67	0	0	0	0	41.67	41.67
Retene	Chorion	3	45	18883	0	0	0	0	0	66.67	8.33	66.67	66.67	66.67	0	66.67	0	0	58.33	8.33	0	41.67	0	0	0	0	75	75
Retene	Chorion	3	65	18883	0	0	0	0	8.33	81.82	0	81.82	90.91	90.91	0	81.82	9.09	0	81.82	0	0	54.55	0	0	0	0	91.67	100
Retene	Chorion	3	100	18883	0	0	0	0	0	100	8.33	100	100	100	0	100	0	0	100	0	0	41.67	0	0	0	0	100	100
Retene	DR	1	0	18845	16.67	0	0	0	16.67	0	0	0	0	0	0	0	0	0	0	0	0	0	0	0	0	0	0	16.67
Retene	DR	1	1	18845	50	0	0	0	58.33	0	0	0	0	0	0	0	0	0	0	0	0	0	0	0	0	0	0	58.33
Retene	DR	1	5	18845	33.33	0	0	0	33.33	0	0	0	0	0	0	0	0	0	0	0	0	0	0	0	0	0	0	33.33
Retene	DR	1	20	18845	0	0	0	0	0	100	66.67	100	100	100	8.33	100	33.33	0	100	66.67	0	0	83.33	91.67	0	0	100	100
Retene	DR	1	30	18845	8.33	0	0	0	25	100	100	100	100	100	0	100	22.22	0	100	77.78	0	0	100	100	0	0	81.82	100
Retene	DR	1	45	18845	8.33	0	0	0	16.67	100	90	100	100	100	10	100	40	0	100	100	0	0	100	100	0	0	90.91	100
Retene	DR	1	65	18845	33.33	0	0	0	66.67	100	100	100	100	100	0	100	0	0	100	100	0	0	100	100	0	0	50	100
Retene	DR	1	100	18845	50	0	0	0	100	NA	NA	NA	NA	NA	NA	NA	NA	NA	NA	NA	NA	NA	NA	NA	NA	NA	0	100
Retene	DR	2	0	18848	16.67	0	0	0	16.67	0	0	0	0	0	0	0	0	0	0	0	0	0	0	0	0	0	0	16.67
Retene	DR	2	1	18848	8.33	0	0	0	8.33	0	9.09	0	0	0	0	0	0	0	0	9.09	0	0	9.09	0	0	0	9.09	16.67
Retene	DR	2	5	18848	16.67	0	0	0	25	0	0	0	0	0	0	0	0	0	0	0	0	0	0	0	0	0	0	25
Retene	DR	2	20	18848	16.67	0	0	0	33.33	100	100	100	100	100	12.5	100	87.5	0	100	100	0	0	100	100	0	0	80	100

Test Agent	Treatment	Replicate plate #	Concentration	Plate Barcode	MO24	DP24	SM24	NC24	MORT	YSE_	AXIS	EYE_	SNOU	JAW_	OTIC	PE__	BRAI	SOMI	PFIN	CFIN	PIG_	CIRC	TRUN	SWIM	NC__	TR__	.except.M(	any.effect
Retene	DR	2	30	18848	33.33	0	0	0	58.33	100	80	100	100	100	20	100	60	20	100	100	0	0	100	100	0	20	62.5	100
Retene	DR	2	45	18848	33.33	0	0	0	50	100	66.67	100	100	100	0	100	66.67	0	100	100	0	0	100	100	0	16.67	75	100
Retene	DR	2	65	18848	50	0	0	0	83.33	100	100	100	100	100	50	100	100	0	100	100	0	0	100	100	0	0	33.33	100
Retene	DR	2	100	18848	41.67	0	0	0	83.33	100	100	100	100	100	100	100	100	0	50	100	0	0	100	100	0	100	28.57	100
Retene	DR	3	0	19002	8.33	9.09	0	0	16.67	0	0	0	0	0	0	0	0	0	0	0	0	0	0	0	0	0	9.09	16.67
Retene	DR	3	1	19002	33.33	0	0	0	33.33	0	0	0	0	0	0	0	0	0	0	0	0	0	0	0	0	0	0	33.33
Retene	DR	3	5	19002	16.67	0	0	0	16.67	10	0	10	10	10	0	10	0	0	10	0	0	0	0	0	0	0	10	25
Retene	DR	3	20	19002	8.33	0	0	0	8.33	100	81.82	100	100	100	27.27	100	90.91	0	100	100	0	0	100	90.91	0	9.09	100	100
Retene	DR	3	30	19002	16.67	20	0	0	25	100	77.78	100	100	100	11.11	100	100	0	100	100	0	0	100	100	0	11.11	100	100
Retene	DR	3	45	19002	33.33	62.5	0	0	66.67	100	75	100	100	100	50	100	100	25	100	100	0	0	100	75	0	25	100	100
Retene	DR	3	65	19002	58.33	60	0	0	75	100	100	100	100	100	66.67	100	100	33.33	100	100	0	0	100	100	0	66.67	100	100
Retene	DR	3	100	19002	58.33	80	0	0	91.67	0	0	0	0	0	0	0	0	0	0	0	0	0	0	0	0	0	80	100
Retene	LD	1	0	18841	25	0	0	0	25	0	0	0	0	0	0	0	0	0	0	0	0	0	0	0	0	0	0	25
Retene	LD	1	1	18841	25	0	0	0	25	0	0	0	0	0	0	0	0	0	11.11	0	0	0	0	0	0	0	11.11	33.33
Retene	LD	1	5	18841	25	0	0	0	25	0	0	0	0	0	0	0	0	0	0	11.11	0	0	0	0	0	0	11.11	33.33
Retene	LD	1	20	18841	25	0	0	0	25	22.22	11.11	22.22	22.22	22.22	0	22.22	0	0	22.22	11.11	0	0	0	0	0	0	22.22	41.67
Retene	LD	1	30	18841	25	0	0	0	33.33	75	37.5	62.5	75	75	12.5	62.5	0	12.5	75	12.5	0	12.5	12.5	0	12.5	12.5	66.67	83.33
Retene	LD	1	45	18841	8.33	0	0	0	50	83.33	33.33	83.33	83.33	83.33	0	83.33	0	0	83.33	0	0	50	0	0	0	0	45.45	91.67
Retene	LD	1	65	18841	41.67	0	0	0	100	NA	NA	NA	NA	NA	NA	NA	NA	NA	NA	NA	NA	NA	NA	NA	NA	NA	0	100
Retene	LD	1	100	18841	50	0	0	0	91.67	100	100	100	100	100	0	100	100	0	100	0	0	0	0	0	0	0	16.67	100
Retene	LD	2	0	18842	25	0	0	0	25	0	0	0	0	0	0	0	0	0	0	0	0	0	0	0	0	0	0	25
Retene	LD	2	1	18842	8.33	0	0	0	16.67	10	10	10	10	10	0	10	0	0	10	0	0	0	0	0	0	0	9.09	25
Retene	LD	2	5	18842	33.33	0	0	0	33.33	0	0	0	0	0	0	0	0	0	0	0	0	0	0	0	0	0	0	33.33
Retene	LD	2	20	18842	0	0	0	0	8.33	36.36	0	36.36	36.36	36.36	0	36.36	0	0	36.36	0	0	0	0	0	0	0	33.33	41.67
Retene	LD	2	30	18842	16.67	0	0	0	25	77.78	44.44	77.78	77.78	77.78	0	77.78	0	0	77.78	0	0	44.44	0	0	0	0	70	83.33
Retene	LD	2	45	18842	25	0	0	0	58.33	80	20	80	80	80	0	80	0	0	80	0	0	0	0	0	0	0	44.44	91.67
Retene	LD	2	65	18842	16.67	0	0	0	83.33	100	0	100	100	100	50	100	0	0	100	50	0	0	0	0	0	50	20	100
Retene	LD	2	100	18842	66.67	0	0	0	91.67	100	100	100	100	100	0	100	100	0	100	0	0	0	0	0	0	0	25	100
Retene	LD	3	0	18849	8.33	0	0	0	8.33	0	0	0	0	0	0	0	0	0	0	0	0	0	0	0	0	0	0	8.33
Retene	LD	3	1	18849	8.33	0	0	0	8.33	0	0	0	0	0	0	0	0	0	0	0	0	0	0	0	0	0	0	8.33
Retene	LD	3	5	18849	25	0	0	0	25	11.11	11.11	11.11	11.11	11.11	0	11.11	11.11	0	22.22	0	0	0	11.11	0	0	0	22.22	41.67
Retene	LD	3	20	18849	8.33	0	0	0	16.67	10	10	10	20	20	0	10	0	0	20	0	0	0	10	0	0	0	27.27	41.67
Retene	LD	3	30	18849	8.33	0	0	0	16.67	70	20	80	80	70	0	70	0	0	70	10	0	10	0	0	0	0	72.73	83.33
Retene	LD	3	45	18849	8.33	0	0	0	33.33	87.5	0	87.5	100	100	0	87.5	0	0	87.5	12.5	0	62.5	0	0	0	0	72.73	100
Retene	LD	3	65	18849	33.33	0	0	0	75	100	66.67	100	100	100	0	100	0	0	100	0	0	33.33	0	0	0	0	37.5	100
Retene	LD	3	100	18849	50	0	0	0	91.67	100	100	100	100	100	0	100	100	0	100	0	0	0	0	0	0	100	16.67	100
Retene	Standard	1	0	18846	16.67	0	0	0	16.67	10	0	0	0	0	0	0	0	0	0	0	0	0	0	0	0	0	10	25
Retene	Standard	1	1	18846	50	0	0	0	58.33	0	0	0	0	0	0	0	0	0	0	0	0	0	0	0	0	0	0	58.33
Retene	Standard	1	5	18846	25	0	0	0	25	0	0	0	0	0	0	0	0	0	0	0	0	0	0	0	0	0	0	25
Retene	Standard	1	20	18846	16.67	0	0	0	16.67	10	10	10	10	10	0	10	0	0	10	10	0	0	20	10	0	0	20	33.33
Retene	Standard	1	30	18846	25	0	0	0	33.33	50	25	50	50	37.5	0	50	25	0	50	50	0	0	50	50	0	0	44.44	66.67
Retene	Standard	1	45	18846	8.33	0	0	0	41.67	100	71.43	100	100	100	14.29	85.71	85.71	0	100	85.71	0	0	85.71	85.71	0	0	63.64	100
Retene	Standard	1	65	18846	33.33	0	0	0	75	100	100	100	100	100	0	100	100	0	100	100	0	0	100	100	0	0	37.5	100
Retene	Standard	1	100	18846	33.33	0	0	0	91.67	100	100	100	100	100	0	100	100	0	100	100	0	0	100	0	0	0	12.5	100
Retene	Standard	2	0	19006	8.33	0	0	0	8.33	0	0	0	0	0	0	0	0	0	0	0	0	0	0	0	0	0	0	8.33
Retene	Standard	2	1	19006	18.18	0	0	0	18.18	11.11	11.11	11.11	11.11	11.11	0	11.11	0	0	11.11	0	0	0	0	0	0	0	11.11	27.27
Retene	Standard	2	5	19006	16.67	0	0	0	16.67	0	0	0	0	0	0	0	0	0	0	0	0	0	0	0	0	0	0	16.67
Retene	Standard	2	20	19006	16.67	0	0	0	25	44.44	11.11	55.56	66.67	66.67	0	55.56	0	0	55.56	0	0	22.22	11.11	0	0	0	60	75
Retene	Standard	2	30	19006	25	11.11	0	0	50	100	50	100	100	100	0	100	0	0	100	0	0	100	0	0	0	0	77.78	100
Retene	Standard	2	45	19006	16.67	10	0	0	50	100	33.33	100	100	100	0	100	0	0	100	0	0	50	0	0	0	0	70	100
Retene	Standard	2	65	19006	50	33.33	0	0	66.67	100	25	100	100	100	0	100	0	0	100	50	0	25	25	0	0	25	83.33	100
Retene	Standard	2	100	19006	41.67	71.43	0	0	100	NA	NA	NA	NA	NA	NA	NA	NA	NA	NA	NA	NA	NA	NA	NA	NA	NA	71.43	100
Retene	Standard	3	0	19008	8.33	0	0	0	8.33	0	0	0	0	0	0	0	0	0	0	0	0	0	0	0	0	0	0	8.33
Retene	Standard	3	1	19008	33.33	0	0	0	33.33	0	0	0	0	0	0	0	0	0	0	0	0	0	0	0	0	0	0	33.33
Retene	Standard	3	5	19008	8.33	0	0	0	16.67	0	0	0	0	0	0	0	0	0	0	0	0	0	0	0	0	0	0	16.67
Retene	Standard	3	20	19008	0	0	0	0	0	8.33	8.33	8.33	16.67	16.67	0	16.67	0	0	16.67	0	0	8.33	0	0	0	0	25	25
Retene	Standard	3	30	19008	0	0	0	0	9.09	100	40	100	100	100	0	100	0	0	100	0	0	70	0	0	0	0	90.91	100
Retene	Standard	3	45	19008	0	33.33	0	0	41.67	100	71.43	100	100	100	0	100	0	0	100	0	0	57.14	0	0	0	0	91.67	100
Retene	Standard	3	65	19008	25	66.67	0	0	50	100	33.33	100	100	100	0	100	0	16.67	100	33.33	0	66.67	50	0	0	83.33	100	100
Retene	Standard	3	100	19008	58.33	100	0	0	100	NA	NA	NA	NA	NA	NA	NA	NA	NA	NA	NA	NA	NA	NA	NA	NA	NA	100	100

Abamectin

Chorion on

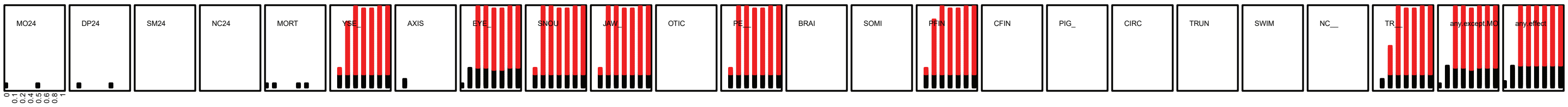

Daily Renewal

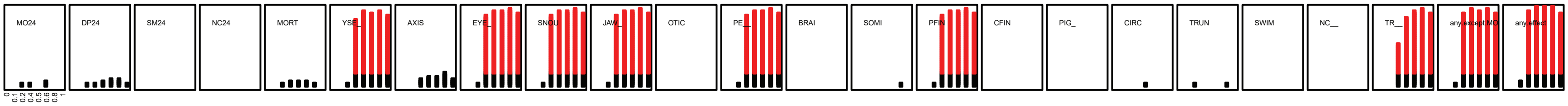

Light/Dark

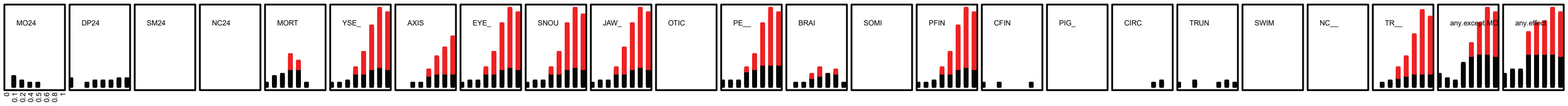

Standard

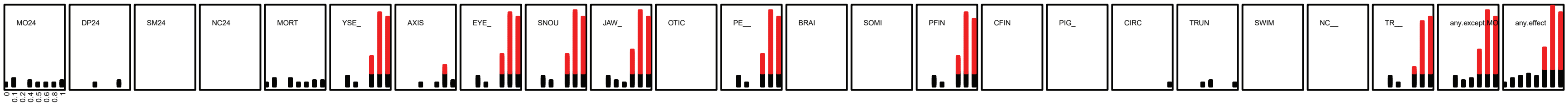

Chlorpyrifos

Chorion on

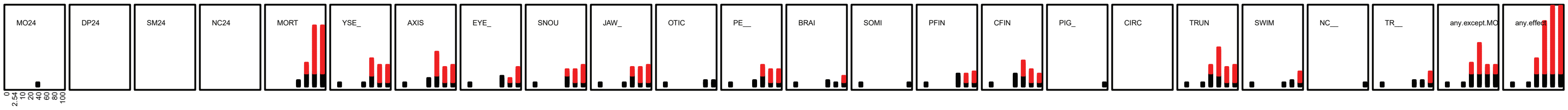

Daily Renewal

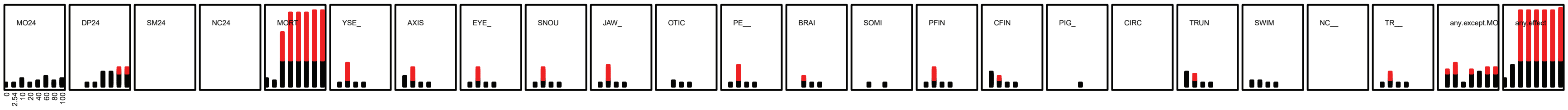

Light/Dark

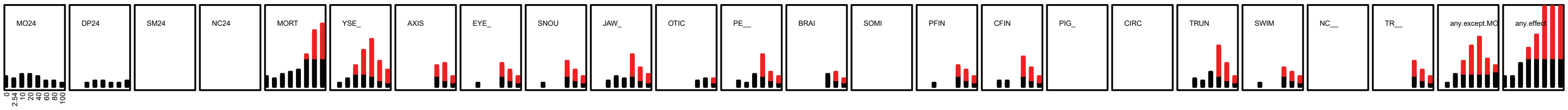

Standard

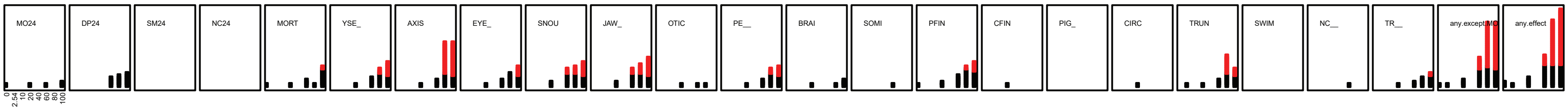

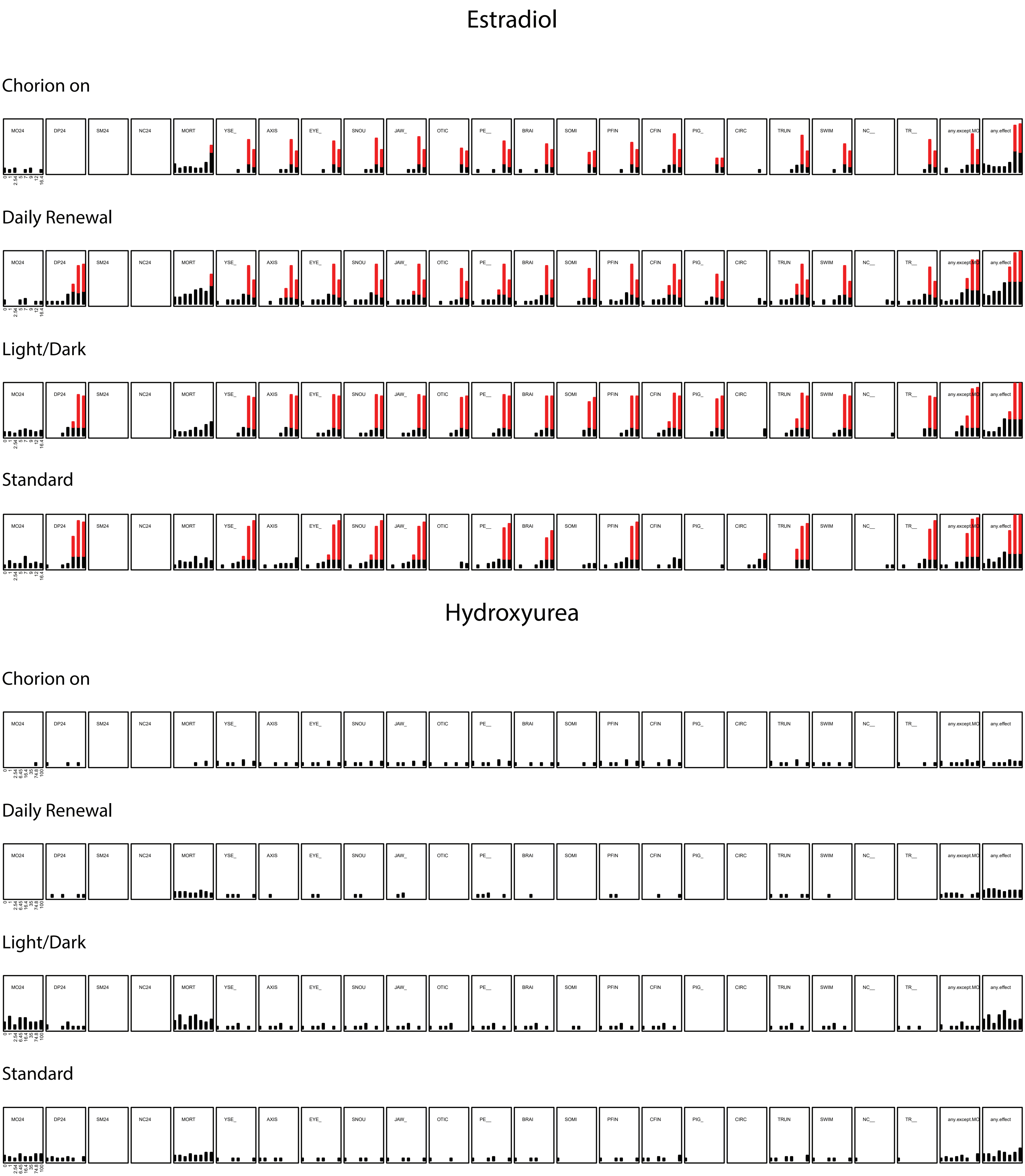
Estradiol
Chorion on
Daily Renewal
Light/Dark
Standard
Hydroxyurea
Chorion on
Daily Renewal
Light/Dark
Standard
MO24
DP24
SM24
NC24
MORT
YSE_
AXIS
EYE_
SNOU
JAW_
OTIC
PE__
BRAI
SOMI
PFIN
CFIN
PIG_
CIRC
TRUN
SWIM
NC__
TR__
any.except.MO
any.effect
0 1 2.54 5 7 9 12 16.4
0 1 2.54 6.45 16.4 35 74.8 100

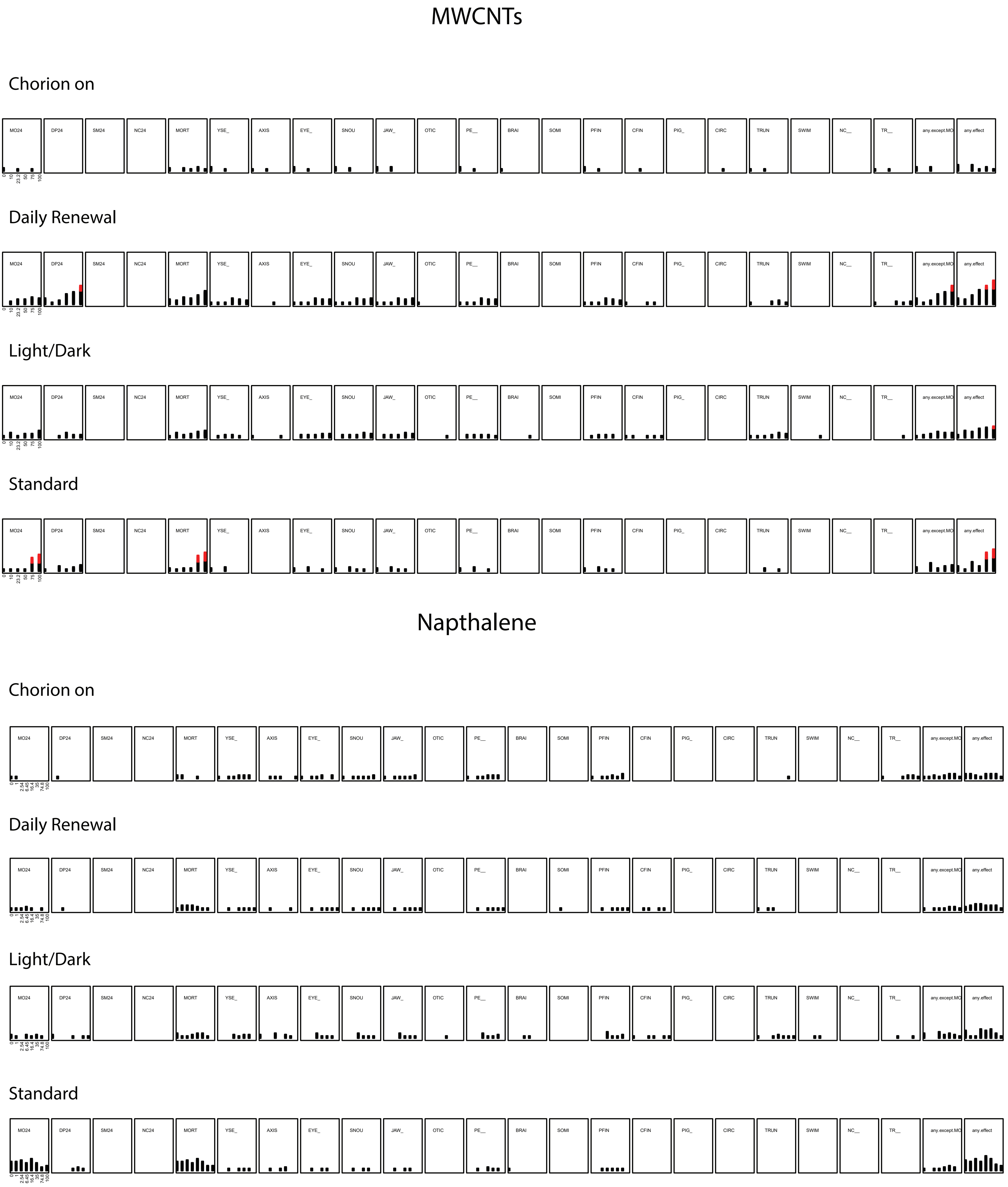

MWCNTs
Chorion on
Daily Renewal
Light/Dark
Standard
MO24
DP24
SM24
NC24
MORT
YSE_
AXIS
EYE_
SNOU
JAW_
OTIC
PE__
BRAI
SOMI
PFIN
CFIN
PIG_
CIRC
TRUN
SWIM
NC__
TR__
any.except.MO
any.effect
0
10
23.2
50
75
100
Napthalene
Chorion on
Daily Renewal
Light/Dark
Standard
0
1
2.54
6.45
16.4
35
74.8
100

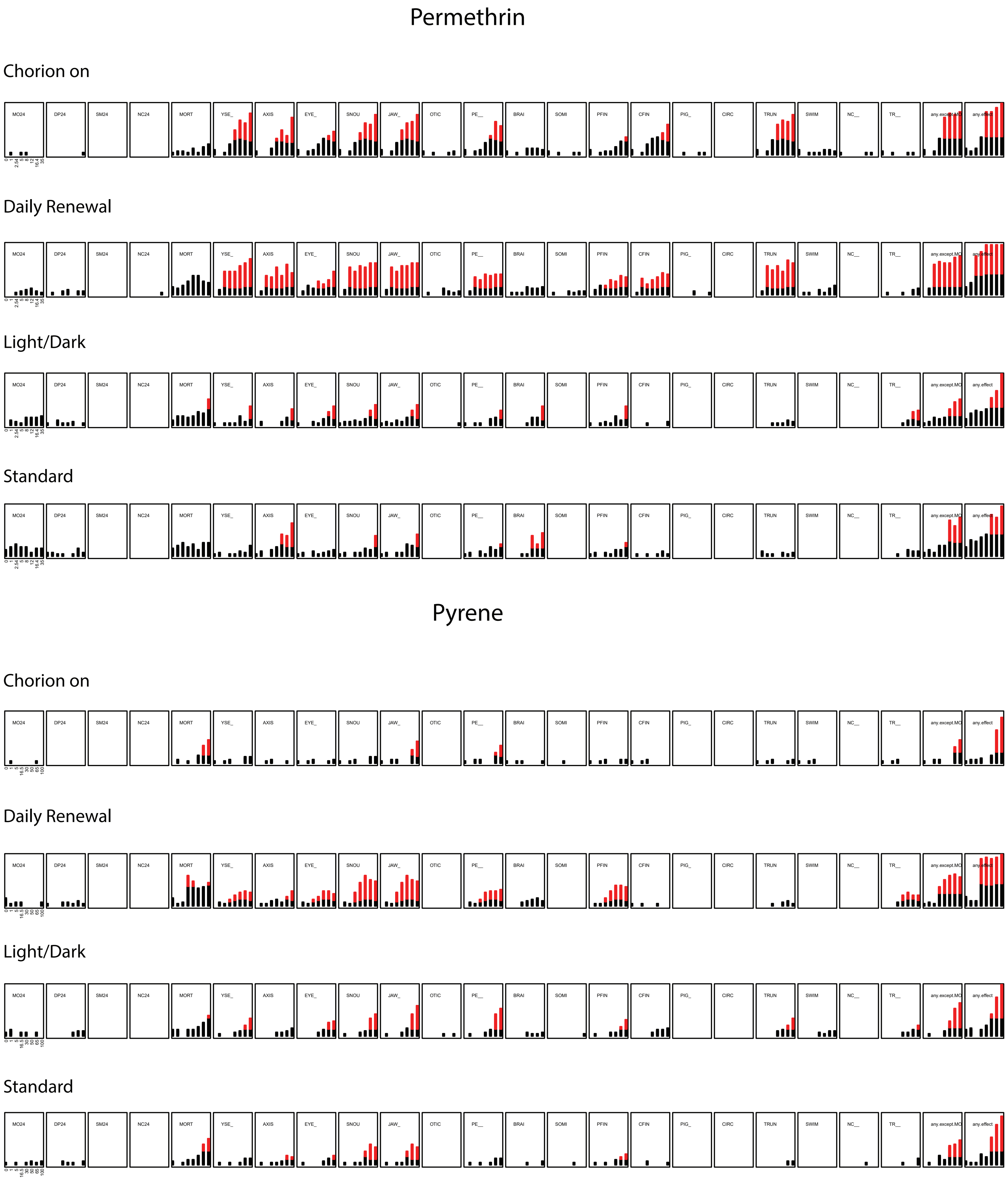
Permethrin
Chorion on
Daily Renewal
Light/Dark
Standard
Pyrene
Chorion on
Daily Renewal
Light/Dark
Standard
MO24
DP24
SM24
NC24
MORT
YSE_
AXIS
EYE_
SNOU
JAW_
OTIC
PE__
BRAI
SOMI
PFIN
CFIN
PIG_
CIRC
TRUN
SWIM
NC__
TR__
any.except.MO
any.effect
0 1 2.54 5 8 12 16.4 35
0 1 5 16.5 30 50 65 100

Retene

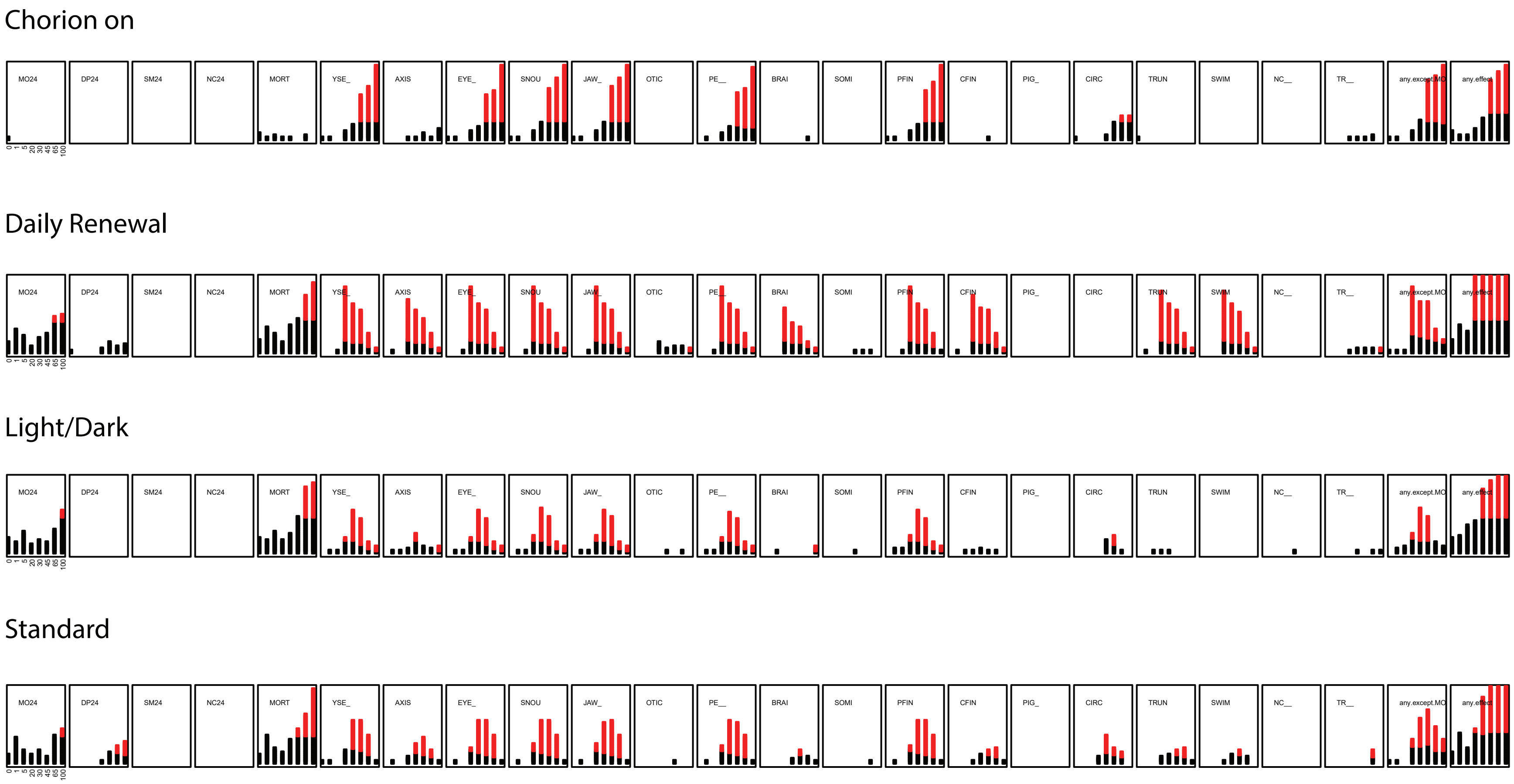

Figure S1. Concentration-response plots for each exposure regimen for each test agent. Each panel represents one endpoint. The horizontal axis shows concentrations (see values under first panel for each condition-chemical combination). For all chemicals, concentration units are micromolar. For MWCNTs, concentration units are micrograms per milliliter. The vertical axis shows incidence counts for each panel. Red indicates statistical significance for each measure ($p < 0.05$). Endpoints for abnormal development at 24hpf: MO24 = mortality; DP24 = delayed progression; SM24 = spontaneous movement; NC24 = notochord. Endpoints at 120hpf: MORT = mortality; YSE_ = yolk sac edema; AXIS = bent body axis; EYE_ = eye; SNOU = snout; JAW_ = jaw; OTIC = otic vesicle; PE_ = pericardial edema; BRAI = brain; SOMI = somites; PFIN = pectoral fin; CFIN = caudal fin; PIG_ = pigment; CIRC = circulatory system; TRUN = trunk; SWIM = swim bladder; NC = notochord; TR_ = touch response; any.exceptMO = any non-lethal effect; any.effect = any effect.

Table S2. Embryonic photomotor response (EPR) compiled data for standard and chorion-on conditions. B = background; E = excitation; R = refractory. For all chemicals, concentration units are micromolar. For MWCNTs, concentration units are micrograms per milliliter. For significance column, 1 = significant, 0 = not significant.

Test Agent	Treatment	Concentration	Phase	Movement.peak	Movement.sd	Movement.n	ks.stat	ks.pval	diff	delta	sig
Abamectin	Chorion	0	B	2.840883833	5.677150491	315	0.205904762	1.34E-10	0	0	0
Abamectin	Chorion	0	E	6.263985996	11.91415409	315	0.313015873	0	0	0	0
Abamectin	Chorion	0	R	0.12757312	1.470988323	280	0.008285714	1	0	0	0
Abamectin	Chorion	0.1	B	1.967324594	5.494205134	324	0.095555556	0.011346103	-0.873559239	-0.307495586	0
Abamectin	Chorion	0.1	E	3.232703488	8.585007185	324	0.115209877	0.001085945	-3.031282509	-0.4839223	0
Abamectin	Chorion	0.1	R	0.223870822	2.255005604	288	0.014805556	0.999999998	0.096297702	0.754843201	0
Abamectin	Chorion	0.2	B	0.51514	2.693240345	324	0.024790123	0.994983465	-2.325743833	-0.818669108	0
Abamectin	Chorion	0.2	E	0.632220496	3.791549056	324	0.047037037	0.557887005	-5.631765501	-0.899070576	0
Abamectin	Chorion	0.2	R	0.121693594	1.365821315	288	0.006416667	1	-0.005879526	-0.0460875	0
Abamectin	Chorion	0.4	B	0.000133959	0.00170238	324	0.068444444	0.140754185	-2.840749874	-0.999952846	0
Abamectin	Chorion	0.4	E	0	0	324	0.094666667	0.01248653	-6.263985996	-1	0
Abamectin	Chorion	0.4	R	0.000150704	0.001805295	288	0.006	1	-0.127422416	-0.998818685	0
Abamectin	Chorion	0.5	B	0	0	315	0.071111111	0.122257802	-2.840883833	-1	0
Abamectin	Chorion	0.5	E	0	0	315	0.094666667	0.014130572	-6.263985996	-1	0
Abamectin	Chorion	0.5	R	0	0	280	0.0095	1	-0.12757312	-1	0
Abamectin	Chorion	0.6	B	0	0	324	0.071111111	0.114020856	-2.840883833	-1	0
Abamectin	Chorion	0.6	E	0	0	324	0.094666667	0.01248653	-6.263985996	-1	0
Abamectin	Chorion	0.6	R	0.000150704	0.001805295	288	0.006	1	-0.127422416	-0.998818685	0
Abamectin	Chorion	0.8	B	0.000133959	0.002411267	324	0.068024691	0.145390979	-2.840749874	-0.999952846	0
Abamectin	Chorion	0.8	E	0.000133959	0.002411264	324	0.092	0.016553676	-6.263852037	-0.999978614	0
Abamectin	Chorion	0.8	R	0	0	288	0.0095	1	-0.12757312	-1	0
Abamectin	Chorion	1	B	0	0	324	0.071111111	0.114020856	-2.840883833	-1	0
Abamectin	Chorion	1	E	0	0	324	0.094666667	0.01248653	-6.263985996	-1	0
Abamectin	Chorion	1	R	0	0	288	0.0095	1	-0.12757312	-1	0
Abamectin	Standard	0	B	7.583705645	14.22250459	315	0.066235548	0.178441492	0	0	0
Abamectin	Standard	0	E	33.1041251	45.96385156	315	0.136246652	7.27E-05	0	0	0
Abamectin	Standard	0	R	0.224919346	3.762319197	280	0.010861258	1	0	0	0
Abamectin	Standard	0.1	B	7.82806486	15.52920055	297	0.051793657	0.484447228	0.244359215	0.032221611	0
Abamectin	Standard	0.1	E	37.06984554	50.49734296	297	0.138338114	9.00E-05	3.965720441	0.119795356	0
Abamectin	Standard	0.1	R	0.300613532	3.459653558	264	0.008511036	1	0.075694186	0.336539238	0
Abamectin	Standard	0.2	B	6.681215257	14.41202168	324	0.024348422	0.996156506	-0.902490388	-0.119003879	0
Abamectin	Standard	0.2	E	32.89374764	51.90012228	324	0.074074074	0.09038117	-0.210377452	-0.006355022	0
Abamectin	Standard	0.2	R	0.41247689	4.124858321	288	0.007587449	1	0.187557544	0.833887999	0
Abamectin	Standard	0.4	B	8.40524579	17.15110593	306	0.089876005	0.026157596	0.821540146	0.108329646	0
Abamectin	Standard	0.4	E	25.11964103	41.80847221	306	0.035369429	0.890210001	-7.984484069	-0.241193025	0
Abamectin	Standard	0.4	R	0.895900281	8.539734817	272	0.007942992	1	0.670980935	2.983206848	0
Abamectin	Standard	0.5	B	5.702987905	13.2121096	315	0.024090404	0.997218318	-1.880717739	-0.247994559	0
Abamectin	Standard	0.5	E	23.439912	40.09082541	315	0.073107322	0.105375373	-9.664213095	-0.291933802	0
Abamectin	Standard	0.5	R	0.598803361	4.747440412	280	0.014138742	1	0.373884015	1.662302602	0
Abamectin	Standard	0.6	B	5.977665263	14.08336905	315	0.024129597	0.997152361	-1.606040382	-0.211775148	0
Abamectin	Standard	0.6	E	14.85298236	32.47182157	315	0.09367039	0.015944711	-18.25114274	-0.551325331	0
Abamectin	Standard	0.6	R	0.000232515	0.00223824	280	0.010288066	1	-0.224686832	-0.998966231	0
Abamectin	Standard	0.8	B	1.734526867	7.777642253	315	0.170161343	2.38E-07	-5.849178777	-0.771282411	1
Abamectin	Standard	0.8	E	1.308077518	7.929896115	315	0.336416487	0	-31.79604758	-0.960485966	1
Abamectin	Standard	0.8	R	0.189499558	1.775587582	280	0.006687243	1	-0.035419788	-0.157477729	0
Abamectin	Standard	1	B	0.118719382	2.069277544	306	0.227184701	1.85E-12	-7.464986262	-0.984345465	1

Test Agent	Treatment	Concentration	Phase	Movement.peak	Movement.sd	Movement.n	ks.stat	ks.pval	diff	delta	sig
Abamectin	Standard	1	E	0.094535753	1.439365191	306	0.364614433	0	-33.00958934	-0.997144291	1
Abamectin	Standard	1	R	0	0	272	0.018004115	0.999998934	-0.224919346	-1	0
Chlorpyrifos	Chorion	0	B	2.175564585	6.215975661	324	0	1	0	0	0
Chlorpyrifos	Chorion	0	E	6.621937966	12.4776157	324	0	1	0	0	0
Chlorpyrifos	Chorion	0	R	0.367190508	2.603513428	288	0	1	0	0	0
Chlorpyrifos	Chorion	2.54	B	2.919707467	7.119270656	324	0.064814815	0.504120853	0.744142882	0.34204587	0
Chlorpyrifos	Chorion	2.54	E	5.679869419	10.42340727	324	0.043209877	0.922844972	-0.942068548	-0.14226478	0
Chlorpyrifos	Chorion	2.54	R	0.982666299	4.748785837	288	0.052083333	0.829553062	0.615475791	1.676175658	0
Chlorpyrifos	Chorion	10	B	3.523059885	8.007986284	324	0.067901235	0.443946011	1.3474953	0.619377291	0
Chlorpyrifos	Chorion	10	E	4.170885821	8.771769186	324	0.111111111	0.036631053	-2.451052145	-0.370141212	0
Chlorpyrifos	Chorion	10	R	1.52708427	5.150247004	288	0.079861111	0.317377205	1.159893762	3.158833731	0
Chlorpyrifos	Chorion	20	B	3.519777888	8.049913838	324	0.083333333	0.210551633	1.344213303	0.617868719	0
Chlorpyrifos	Chorion	20	E	4.247979611	8.445109939	324	0.12654321	0.01116329	-2.373958355	-0.358499033	0
Chlorpyrifos	Chorion	20	R	2.4090803	6.919293759	288	0.159722222	0.00128876	2.041889792	5.560845794	1
Chlorpyrifos	Chorion	40	B	2.533826889	5.679868094	315	0.103174603	0.066717209	0.358262304	0.164675554	0
Chlorpyrifos	Chorion	40	E	2.455494736	5.948779181	315	0.159876543	0.000568895	-4.16644323	-0.629187898	1
Chlorpyrifos	Chorion	40	R	1.806795776	4.847366888	280	0.186507937	0.00010268	1.439605268	3.920594996	1
Chlorpyrifos	Chorion	60	B	2.640000848	6.225057068	324	0.089506173	0.14912849	0.464436263	0.213478499	0
Chlorpyrifos	Chorion	60	E	2.549376632	6.041515815	324	0.154320988	0.000891235	-4.072561334	-0.615010493	1
Chlorpyrifos	Chorion	60	R	1.862928428	5.391386357	288	0.177083333	0.000239226	1.49573792	4.073465647	1
Chlorpyrifos	Chorion	80	B	2.732700478	6.286378345	324	0.114197531	0.029241551	0.557135893	0.256087958	0
Chlorpyrifos	Chorion	80	E	3.146365672	7.106118797	324	0.132716049	0.006646646	-3.475572294	-0.524857272	1
Chlorpyrifos	Chorion	80	R	2.023051374	5.353777839	288	0.197916667	2.52E-05	1.655860866	4.509541587	1
Chlorpyrifos	Chorion	100	B	2.206442181	5.14436284	324	0.080246914	0.247786694	0.030877595	0.014192911	0
Chlorpyrifos	Chorion	100	E	2.511600483	6.235739176	324	0.151234568	0.001209744	-4.110337484	-0.62071519	1
Chlorpyrifos	Chorion	100	R	2.233962183	5.664175317	288	0.208333333	7.45E-06	1.866771674	5.083932274	1
Chlorpyrifos	Standard	0	B	8.727746981	15.56061454	315	0	1	0	0	0
Chlorpyrifos	Standard	0	E	30.81996773	37.00450783	315	0	1	0	0	0
Chlorpyrifos	Standard	0	R	0.075412377	1.261890431	280	0	1	0	0	0
Chlorpyrifos	Standard	2.54	B	7.739425508	13.01655983	324	0.032892416	0.995216982	-0.988321472	-0.113239015	0
Chlorpyrifos	Standard	2.54	E	24.58981607	34.31619027	324	0.102910053	0.067889171	-6.230151666	-0.202146599	0
Chlorpyrifos	Standard	2.54	R	1.298466424	6.899656785	288	0.045039683	0.935580803	1.223054046	16.21821366	0
Chlorpyrifos	Standard	10	B	8.487319373	13.37400684	324	0.054850088	0.722533857	-0.240427607	-0.0275475	0
Chlorpyrifos	Standard	10	E	17.09252372	26.71959311	324	0.23659612	3.43E-08	-13.72744401	-0.445407475	0
Chlorpyrifos	Standard	10	R	1.711470889	6.651658098	288	0.083234127	0.278979336	1.636058511	21.69482745	0
Chlorpyrifos	Standard	20	B	8.860849627	14.45523894	315	0.034920635	0.990716312	0.133102647	0.015250516	0
Chlorpyrifos	Standard	20	E	10.23657928	15.1974654	315	0.301587302	7.21E-13	-20.58338845	-0.667858858	1
Chlorpyrifos	Standard	20	R	3.996930837	10.47431597	280	0.178571429	0.000265094	3.92151846	52.00099246	1
Chlorpyrifos	Standard	40	B	7.471372478	13.47420362	324	0.048324515	0.849772551	-1.256374502	-0.143951756	0
Chlorpyrifos	Standard	40	E	7.687249294	14.80475874	324	0.328571429	2.11E-15	-23.13271844	-0.750575686	1
Chlorpyrifos	Standard	40	R	4.351279465	10.48282515	288	0.222123016	1.65E-06	4.275867088	56.69980506	1
Chlorpyrifos	Standard	60	B	5.81721176	11.01724319	315	0.104761905	0.063037405	-2.910535221	-0.333480706	0
Chlorpyrifos	Standard	60	E	4.760251339	11.29012163	315	0.387301587	0	-26.05971639	-0.845546518	1
Chlorpyrifos	Standard	60	R	3.139338205	8.760782266	280	0.189285714	8.79E-05	3.063925827	40.62895164	1
Chlorpyrifos	Standard	80	B	4.281870989	9.545751409	324	0.149029982	0.001659034	-4.445875992	-0.509395609	1
Chlorpyrifos	Standard	80	E	3.712411807	8.748233732	324	0.401851852	0	-27.10755592	-0.87954524	1

Test Agent	Treatment	Concentration	Phase	Movement.peak	Movement.sd	Movement.n	ks.stat	ks.pval	diff	delta	sig
Chlorpyrifos	Standard	80	R	2.567922051	7.507023314	288	0.159623016	0.001442183	2.492509674	33.0517319	1
Chlorpyrifos	Standard	100	B	2.326658511	6.315099215	306	0.210644258	2.08E-06	-6.40108847	-0.733418199	1
Chlorpyrifos	Standard	100	E	2.473249019	7.000925748	306	0.43286648	0	-28.34671871	-0.919751732	1
Chlorpyrifos	Standard	100	R	1.301205726	4.534114429	272	0.121428571	0.034197769	1.225793349	16.25453797	0
Estradiol	Chorion	0	B	2.931744234	9.388770973	306	0	1	0	0	0
Estradiol	Chorion	0	E	3.8853287	9.956068172	306	0	1	0	0	0
Estradiol	Chorion	0	R	0.777260808	4.233908858	272	0	1	0	0	0
Estradiol	Chorion	1	B	2.942570916	7.134867533	315	0.065546218	0.517360423	0.010826683	0.003692915	0
Estradiol	Chorion	1	E	5.360242862	11.17937865	315	0.071615313	0.40355245	1.474914163	0.379611167	0
Estradiol	Chorion	1	R	0.424726896	3.279231891	280	0.02237395	0.999999833	-0.352533912	-0.453559356	0
Estradiol	Chorion	2.54	B	2.698914971	7.211232211	306	0.026143791	0.99994167	-0.232829262	-0.079416635	0
Estradiol	Chorion	2.54	E	4.186311789	10.54707956	306	0.032679739	0.996738369	0.300983089	0.07746657	0
Estradiol	Chorion	2.54	R	0.582427074	4.124574459	272	0.014705882	1	-0.194833734	-0.250667128	0
Estradiol	Chorion	5	B	2.06056049	5.520859701	324	0.036310821	0.985600275	-0.871183744	-0.297155439	0
Estradiol	Chorion	5	E	3.795800846	8.922149099	324	0.031771968	0.997333967	-0.089527854	-0.023042543	0
Estradiol	Chorion	5	R	0.130886485	1.59845809	288	0.033496732	0.997558956	-0.646374323	-0.831605448	0
Estradiol	Chorion	7	B	2.057843171	5.648026079	315	0.042763772	0.939049148	-0.873901063	-0.2980823	0
Estradiol	Chorion	7	E	3.332644171	7.700770049	315	0.057609711	0.681540148	-0.552684528	-0.142249104	0
Estradiol	Chorion	7	R	0.383494511	3.01894041	280	0.022584034	0.99999977	-0.393766297	-0.506607683	0
Estradiol	Chorion	9	B	1.794264714	4.945235401	306	0.052287582	0.797010175	-1.13747952	-0.387987297	0
Estradiol	Chorion	9	E	3.481796319	8.279801614	306	0.055555556	0.732450588	-0.403532381	-0.103860551	0
Estradiol	Chorion	9	R	0.639153616	3.477943487	272	0.018382353	1	-0.138107193	-0.177684493	0
Estradiol	Chorion	12	B	0.568522804	3.14195854	324	0.106572259	0.056044298	-2.363221429	-0.806080354	0
Estradiol	Chorion	12	E	0.655194644	2.539822184	324	0.129266521	0.010397876	-3.230134056	-0.831366998	0
Estradiol	Chorion	12	R	0.080928067	0.87083413	288	0.03002451	0.999601982	-0.696332741	-0.895880423	0
Estradiol	Chorion	16.4	B	0.027281728	0.32992823	315	0.154061625	0.001262051	-2.904462506	-0.99069437	1
Estradiol	Chorion	16.4	E	0.073853649	0.65898057	315	0.209897292	2.30E-06	-3.811475051	-0.98099166	1
Estradiol	Chorion	16.4	R	0.032552041	0.319028128	280	0.040441176	0.977718411	-0.744708767	-0.958119539	0
Estradiol	Standard	0	B	7.54788138	14.27310548	315	0	1	0	0	0
Estradiol	Standard	0	E	18.00354161	28.86722414	315	0	1	0	0	0
Estradiol	Standard	0	R	0.904482898	5.20484744	280	0	1	0	0	0
Estradiol	Standard	1	B	7.860198027	13.85759465	288	0.035615079	0.99106429	0.312316648	0.041378055	0
Estradiol	Standard	1	E	19.77832864	34.20886478	288	0.036507937	0.988086858	1.774787024	0.098579883	0
Estradiol	Standard	1	R	0.407409538	3.423629288	256	0.020089286	0.999999999	-0.497073361	-0.549566345	0
Estradiol	Standard	2.54	B	6.512473395	12.96199406	306	0.048926237	0.851385221	-1.035407984	-0.137178624	0
Estradiol	Standard	2.54	E	19.03956405	30.40158071	306	0.038935574	0.972692225	1.036022433	0.057545479	0
Estradiol	Standard	2.54	R	0.502403114	3.564155667	272	0.020798319	0.999999989	-0.402079784	-0.444541058	0
Estradiol	Standard	5	B	7.060894826	12.62941685	306	0.034173669	0.993481325	-0.486986554	-0.064519635	0
Estradiol	Standard	5	E	22.55157301	32.84473258	306	0.069747899	0.436978666	4.548031397	0.252618707	0
Estradiol	Standard	5	R	0.572613391	4.515199628	272	0.017542017	1	-0.331869508	-0.366916288	0
Estradiol	Standard	7	B	4.454854211	10.74566844	261	0.127093596	0.019881446	-3.093027169	-0.409787464	0
Estradiol	Standard	7	E	15.48290227	26.19715191	261	0.051997811	0.834993118	-2.52063934	-0.140007971	0
Estradiol	Standard	7	R	0.776760248	5.07936565	232	0.011330049	1	-0.12772265	-0.141210686	0
Estradiol	Standard	9	B	4.008799473	9.813632787	306	0.133986928	0.007597528	-3.539081907	-0.468884145	0
Estradiol	Standard	9	E	8.894235048	20.52793038	306	0.183660131	5.67E-05	-9.109306564	-0.505973034	1
Estradiol	Standard	9	R	0.189488257	2.040087006	272	0.028361345	0.999888151	-0.714994642	-0.790501007	0

Test Agent	Treatment	Concentration	Phase	Movement.peak	Movement.sd	Movement.n	ks.stat	ks.pval	diff	delta	sig
Estradiol	Standard	12	B	0.222786313	3.329281721	297	0.326599327	1.38E-14	-7.325095066	-0.970483596	1
Estradiol	Standard	12	E	0.91555014	4.253400551	297	0.34987975	1.11E-16	-17.08799147	-0.949146109	1
Estradiol	Standard	12	R	0	0	264	0.042857143	0.964213451	-0.904482898	-1	0
Estradiol	Standard	16.4	B	0	0	306	0.336507937	1.11E-15	-7.54788138	-1	1
Estradiol	Standard	16.4	E	0.000567357	0.009924695	306	0.431652661	0	-18.00297425	-0.999968486	1
Estradiol	Standard	16.4	R	7.98E-05	0.001315839	272	0.039285714	0.983449574	-0.904403114	-0.99991179	0
Hydroxyurea	Chorion	0	B	3.276641885	6.962481751	324	0	1	0	0	0
Hydroxyurea	Chorion	0	E	5.271226601	10.66013218	324	0	1	0	0	0
Hydroxyurea	Chorion	0	R	0.720667123	4.437447827	288	0	1	0	0	0
Hydroxyurea	Chorion	1	B	3.443554892	6.815326591	324	0.043209877	0.922844972	0.166913007	0.050940265	0
Hydroxyurea	Chorion	1	E	5.649393742	11.27341288	324	0.021604938	0.999999251	0.378167141	0.071741773	0
Hydroxyurea	Chorion	1	R	0.552933016	3.561209026	288	0.013888889	1	-0.167734107	-0.232748382	0
Hydroxyurea	Chorion	2.54	B	4.007187878	7.614713058	324	0.058641975	0.633246263	0.730545993	0.222955702	0
Hydroxyurea	Chorion	2.54	E	7.14611909	13.14508576	324	0.067901235	0.443946011	1.874892488	0.355684289	0
Hydroxyurea	Chorion	2.54	R	0.561674053	3.38211411	288	0.010416667	1	-0.15899307	-0.220619292	0
Hydroxyurea	Chorion	6.45	B	3.659296324	6.949379436	324	0.049382716	0.824393461	0.382654438	0.116782502	0
Hydroxyurea	Chorion	6.45	E	7.126629271	13.00460493	324	0.067901235	0.443946011	1.855402669	0.351986892	0
Hydroxyurea	Chorion	6.45	R	0.554289577	4.433611145	288	0.024305556	0.999995675	-0.166377546	-0.230866014	0
Hydroxyurea	Chorion	16.4	B	3.961106131	7.346398591	324	0.074074074	0.336404876	0.684464246	0.208891991	0
Hydroxyurea	Chorion	16.4	E	6.083220518	11.77569332	324	0.052469136	0.763912024	0.811993916	0.154042688	0
Hydroxyurea	Chorion	16.4	R	0.514805425	3.610727646	288	0.013888889	1	-0.205861698	-0.28565435	0
Hydroxyurea	Chorion	35	B	3.474767143	6.870747304	324	0.049382716	0.824393461	0.198125258	0.060465948	0
Hydroxyurea	Chorion	35	E	7.196422142	13.85785829	324	0.067901235	0.443946011	1.925195541	0.36522724	0
Hydroxyurea	Chorion	35	R	0.685778959	3.68063026	288	0.013888889	1	-0.034888164	-0.048410928	0
Hydroxyurea	Chorion	74.8	B	4.266561725	8.114568595	315	0.090564374	0.145551129	0.98991984	0.302114138	0
Hydroxyurea	Chorion	74.8	E	5.543154784	11.68585081	315	0.025396825	0.999950829	0.271928182	0.051587269	0
Hydroxyurea	Chorion	74.8	R	0.304672046	2.967210022	280	0.027380952	0.999928919	-0.415995077	-0.577236096	0
Hydroxyurea	Chorion	100	B	4.201563327	7.338696811	324	0.089506173	0.14912849	0.924921441	0.282277244	0
Hydroxyurea	Chorion	100	E	7.350876431	13.25176507	324	0.089506173	0.14912849	2.07964983	0.394528634	0
Hydroxyurea	Chorion	100	R	0.578176176	3.722006902	288	0.010416667	1	-0.142490947	-0.197720893	0
Hydroxyurea	Standard	0	B	7.420997821	13.51054451	297	0	1	0	0	0
Hydroxyurea	Standard	0	E	15.02203802	24.83864492	297	0	1	0	0	0
Hydroxyurea	Standard	0	R	0.979685908	6.59235631	264	0	1	0	0	0
Hydroxyurea	Standard	1	B	9.320107282	15.22625853	306	0.072984749	0.398267371	1.899109461	0.255910257	0
Hydroxyurea	Standard	1	E	25.12985394	39.00203059	306	0.110516934	0.050359538	10.10781592	0.67286582	0
Hydroxyurea	Standard	1	R	0.812366029	6.062700517	272	0.012143494	1	-0.167319879	-0.170789309	0
Hydroxyurea	Standard	2.54	B	7.600929389	14.42859574	315	0.025300625	0.999973184	0.179931569	0.024246277	0
Hydroxyurea	Standard	2.54	E	19.65670375	33.89576119	315	0.068686869	0.466471973	4.63466573	0.308524431	0
Hydroxyurea	Standard	2.54	R	0.795511217	5.899434535	280	0.009307359	1	-0.184174691	-0.187993611	0
Hydroxyurea	Standard	6.45	B	8.081582079	14.47149387	288	0.040509259	0.970075961	0.660584258	0.089015557	0
Hydroxyurea	Standard	6.45	E	26.33667069	40.93427136	288	0.126683502	0.018315821	11.31463267	0.753202239	0
Hydroxyurea	Standard	6.45	R	0.633154889	5.571964582	256	0.011008523	1	-0.346531019	-0.353716447	0
Hydroxyurea	Standard	16.4	B	8.186104428	14.47431631	306	0.062982769	0.588264118	0.765106607	0.103100233	0
Hydroxyurea	Standard	16.4	E	22.73894383	36.40698492	306	0.084373143	0.233563172	7.716905817	0.513705651	0
Hydroxyurea	Standard	16.4	R	1.25006404	7.757347716	272	0.013926025	1	0.270378132	0.275984507	0
Hydroxyurea	Standard	35	B	7.038341651	14.3942711	306	0.047435136	0.886760912	-0.38265617	-0.051563978	0

Test Agent	Treatment	Concentration	Phase	Movement.peak	Movement.sd	Movement.n	ks.stat	ks.pval	diff	delta	sig
Hydroxyurea	Standard	35	E	23.57678451	34.78465868	306	0.108536344	0.057395072	8.554746495	0.569479753	0
Hydroxyurea	Standard	35	R	0.733459162	5.63803774	272	0.011697861	1	-0.246226746	-0.251332334	0
Hydroxyurea	Standard	74.8	B	7.21503374	13.58107905	288	0.026409933	0.999956236	-0.20596408	-0.02775423	0
Hydroxyurea	Standard	74.8	E	23.03150407	35.46373408	288	0.121001684	0.027641045	8.009466056	0.533181054	0
Hydroxyurea	Standard	74.8	R	1.046922419	5.929665472	256	0.012547348	1	0.067236512	0.068630682	0
Hydroxyurea	Standard	100	B	7.171631325	13.84792492	288	0.036300505	0.990541051	-0.249366496	-0.033602826	0
Hydroxyurea	Standard	100	E	27.62202591	42.93286232	288	0.126578283	0.018459107	12.59998789	0.838766875	0
Hydroxyurea	Standard	100	R	0.464375607	4.583847597	256	0.018821023	1	-0.515310301	-0.525995421	0
MWCNTs	Chorion	0	B	2.765616531	7.653992668	414	0	1	0	0	0
MWCNTs	Chorion	0	E	7.930547146	16.16340638	414	0	1	0	0	0
MWCNTs	Chorion	0	R	0.215067842	2.06506541	368	0	1	0	0	0
MWCNTs	Chorion	10	B	3.020361073	8.265081326	432	0.036332528	0.94294535	0.254744542	0.092111303	0
MWCNTs	Chorion	10	E	6.765659125	13.92238223	432	0.051529791	0.628463211	-1.164888021	-0.146886211	0
MWCNTs	Chorion	10	R	0.335580468	2.843978775	384	0.020040761	0.999999274	0.120512626	0.560347029	0
MWCNTs	Chorion	23.2	B	2.369247858	5.680284547	423	0.031966286	0.983095088	-0.396368673	-0.143320185	0
MWCNTs	Chorion	23.2	E	5.607320918	10.70530552	423	0.081149142	0.12711154	-2.323226228	-0.292946525	0
MWCNTs	Chorion	23.2	R	0.606657665	4.832746784	376	0.031510176	0.992682543	0.391589823	1.820773482	0
MWCNTs	Chorion	50	B	2.330940299	5.775777494	432	0.029388084	0.993179176	-0.434676232	-0.157171548	0
MWCNTs	Chorion	50	E	4.194194808	9.026539922	432	0.10205314	0.024469225	-3.736352338	-0.471134245	0
MWCNTs	Chorion	50	R	0.155809191	1.513633944	384	0.009963768	1	-0.059258651	-0.27553469	0
MWCNTs	Chorion	75	B	2.077946396	5.318460231	423	0.036694419	0.940800068	-0.687670135	-0.24864985	0
MWCNTs	Chorion	75	E	3.495360623	8.387882611	423	0.122160551	0.003881669	-4.435186524	-0.559253535	1
MWCNTs	Chorion	75	R	0.248469329	2.264224562	376	0.005550416	1	0.033401487	0.155306749	0
MWCNTs	Chorion	100	B	2.452206085	5.862443396	432	0.062198068	0.386764091	-0.313410447	-0.113323898	0
MWCNTs	Chorion	100	E	3.917250992	9.037523334	432	0.12328905	0.003235336	-4.013296155	-0.506055393	1
MWCNTs	Chorion	100	R	0.308001603	2.323676193	384	0.015172101	1	0.092933761	0.432113702	0
MWCNTs	Standard	0	B	5.129120682	13.4370001	423	0	1	0	0	0
MWCNTs	Standard	0	E	20.10641454	32.86992063	423	0	1	0	0	0
MWCNTs	Standard	0	R	0.342951054	4.111029888	376	0	1	0	0	0
MWCNTs	Standard	10	B	6.861691758	16.97939437	423	0.044917258	0.786987844	1.732571076	0.337791053	0
MWCNTs	Standard	10	E	28.36725477	48.30446835	423	0.082742317	0.110470968	8.260840227	0.410855959	0
MWCNTs	Standard	10	R	0.256549608	4.025816303	376	0.005319149	1	-0.086401446	-0.25193521	0
MWCNTs	Standard	23.2	B	7.082912701	17.68724637	423	0.052009456	0.61644461	1.953792018	0.380921436	0
MWCNTs	Standard	23.2	E	28.05209795	51.01396775	423	0.082742317	0.110470968	7.945683404	0.395181517	0
MWCNTs	Standard	23.2	R	0.69554113	6.791118701	376	0.010638298	1	0.352590076	1.028106114	0
MWCNTs	Standard	50	B	9.054507159	25.8410671	423	0.056737589	0.503838252	3.925386476	0.765313729	0
MWCNTs	Standard	50	E	22.67148804	47.76416878	423	0.085106383	0.093407418	2.565073501	0.127574884	0
MWCNTs	Standard	50	R	1.007844099	12.04550403	376	0.005319149	1	0.664893045	1.938740344	0
MWCNTs	Standard	75	B	5.263391206	18.51106607	324	0.042684529	0.891805819	0.134270524	0.026178078	0
MWCNTs	Standard	75	E	12.11520184	33.21265233	324	0.191423693	2.89E-06	-7.991212701	-0.397445934	0
MWCNTs	Standard	75	R	0.119960398	2.035795454	288	0.007166076	1	-0.222990657	-0.650211317	0
MWCNTs	Standard	100	B	4.550715943	15.76679132	297	0.058313633	0.59315037	-0.57840474	-0.112768791	0
MWCNTs	Standard	100	E	15.16357074	42.80507536	297	0.162045992	0.000209549	-4.9428438	-0.245834173	0
MWCNTs	Standard	100	R	0.068803147	1.117918825	264	0.007978723	1	-0.274147907	-0.799379105	0
Napthalene	Chorion	0	B	2.871334887	6.148425805	315	0	1	0	0	0
Napthalene	Chorion	0	E	6.631255163	12.05587591	315	0	1	0	0	0

Test Agent	Treatment	Concentration	Phase	Movement.peak	Movement.sd	Movement.n	ks.stat	ks.pval	diff	delta	sig
Napthalene	Chorion	0	R	0.26134679	2.265063009	280	0	1	0	0	0
Napthalene	Chorion	1	B	3.12892669	6.557886624	315	0.031746032	0.997349747	0.257591803	0.089711515	0
Napthalene	Chorion	1	E	6.108148828	11.73677407	315	0.053968254	0.748621806	-0.523106335	-0.078884965	0
Napthalene	Chorion	1	R	0.399770794	3.735363302	280	0.007142857	1	0.138424004	0.529656417	0
Napthalene	Chorion	2.54	B	3.060632514	6.150371202	324	0.038977072	0.968486822	0.189297627	0.065926698	0
Napthalene	Chorion	2.54	E	7.325022348	13.79928525	324	0.04356261	0.922270402	0.693767185	0.104620795	0
Napthalene	Chorion	2.54	R	0.240825174	2.066035073	288	0.00406746	1	-0.020521615	-0.078522547	0
Napthalene	Chorion	6.45	B	3.342884755	6.827157964	324	0.050793651	0.804398499	0.471549868	0.164226705	0
Napthalene	Chorion	6.45	E	7.284365483	12.79601412	324	0.044003527	0.916543447	0.65311032	0.098489698	0
Napthalene	Chorion	6.45	R	0.724057944	3.669748208	288	0.034325397	0.996159877	0.462711155	1.770487236	0
Napthalene	Chorion	16.4	B	3.276440511	6.582364998	324	0.03994709	0.960757971	0.405105624	0.141086164	0
Napthalene	Chorion	16.4	E	5.488307609	11.09753743	324	0.054585538	0.728058466	-1.142947554	-0.17235765	0
Napthalene	Chorion	16.4	R	0.681333225	3.487979461	288	0.030853175	0.999260131	0.419986436	1.607008208	0
Napthalene	Chorion	35	B	3.02392759	6.675822998	324	0.031746032	0.997068217	0.152592703	0.053143471	0
Napthalene	Chorion	35	E	6.145378103	10.8148172	324	0.034126984	0.992344456	-0.48587706	-0.073270753	0
Napthalene	Chorion	35	R	0.156280004	1.556630596	288	0.007539683	1	-0.105066785	-0.40202057	0
Napthalene	Chorion	74.8	B	2.831629136	5.853156003	324	0.025749559	0.999932821	-0.039705751	-0.013828325	0
Napthalene	Chorion	74.8	E	7.267754188	13.18242629	324	0.049559083	0.827626187	0.636499025	0.095984698	0
Napthalene	Chorion	74.8	R	0.413456612	2.467957655	288	0.016964286	1	0.152109823	0.582022924	0
Napthalene	Chorion	100	B	3.032166593	6.597306261	324	0.030864198	0.998066056	0.160831706	0.056012869	0
Napthalene	Chorion	100	E	7.141632672	12.84608319	324	0.039417989	0.965107738	0.510377509	0.076965446	0
Napthalene	Chorion	100	R	0.341947414	2.69286413	288	0.010019841	1	0.080600624	0.308404876	0
Napthalene	Standard	0	B	6.118264412	12.74648715	270	0	1	0	0	0
Napthalene	Standard	0	E	26.35207578	37.94672423	270	0	1	0	0	0
Napthalene	Standard	0	R	0.618218205	4.277602693	240	0	1	0	0	0
Napthalene	Standard	1	B	6.488393911	14.01594893	270	0.022222222	0.999999911	0.370129499	0.060495833	0
Napthalene	Standard	1	E	22.58793138	34.52055838	270	0.088888889	0.236490071	-3.764144403	-0.142840527	0
Napthalene	Standard	1	R	0.625180872	4.468344772	240	0.008333333	1	0.006962667	0.011262474	0
Napthalene	Standard	2.54	B	5.247079663	13.62153802	261	0.068965517	0.553137577	-0.871184749	-0.14239083	0
Napthalene	Standard	2.54	E	23.90254164	36.05151607	261	0.07394636	0.462488307	-2.449534137	-0.092954125	0
Napthalene	Standard	2.54	R	0.664417862	6.165235068	232	0.012212644	1	0.046199657	0.074730341	0
Napthalene	Standard	6.45	B	5.689108754	12.17331873	279	0.030107527	0.999650064	-0.429155657	-0.070143366	0
Napthalene	Standard	6.45	E	27.13109227	36.45208787	279	0.041457587	0.972401732	0.779016491	0.029561864	0
Napthalene	Standard	6.45	R	0.999576279	5.809236576	248	0.027419355	0.999988118	0.381358074	0.616866457	0
Napthalene	Standard	16.4	B	6.614617324	14.21305553	252	0.028835979	0.999913274	0.496352912	0.081126424	0
Napthalene	Standard	16.4	E	28.48875597	42.73914448	252	0.05026455	0.896884959	2.136680186	0.081082045	0
Napthalene	Standard	16.4	R	0.634474897	5.298696235	224	0.00922619	1	0.016256692	0.026296043	0
Napthalene	Standard	35	B	6.649959309	13.92764591	279	0.028793309	0.999855069	0.531694897	0.086902896	0
Napthalene	Standard	35	E	30.84623031	46.0198322	279	0.062724014	0.652897511	4.494154527	0.170542714	0
Napthalene	Standard	35	R	0.550497706	4.447570304	248	0.008736559	1	-0.067720499	-0.109541418	0
Napthalene	Standard	74.8	B	7.00515198	14.50773762	306	0.034204793	0.996073762	0.886887569	0.144957378	0
Napthalene	Standard	74.8	E	27.32850191	41.15637658	306	0.077777778	0.350727904	0.976426129	0.037053101	0
Napthalene	Standard	74.8	R	0.520035457	3.61486407	272	0.013480392	1	-0.098182748	-0.158815685	0
Napthalene	Standard	100	B	7.385121021	14.09154085	297	0.046127946	0.92424492	1.266856609	0.207061435	0
Napthalene	Standard	100	E	17.05283384	31.55560497	297	0.140740741	0.007374887	-9.299241941	-0.352884608	0
Napthalene	Standard	100	R	0.983714128	5.734776169	264	0.013257576	1	0.365495923	0.591208606	0

Test Agent	Treatment	Concentration	Phase	Movement.peak	Movement.sd	Movement.n	ks.stat	ks.pval	diff	delta	sig
Permethrin	Chorion	0	B	1.513605017	5.412050155	324	0	1	0	0	0
Permethrin	Chorion	0	E	4.036525437	9.962544628	324	0	1	0	0	0
Permethrin	Chorion	0	R	0.344811031	2.735102199	288	0	1	0	0	0
Permethrin	Chorion	1	B	1.730737133	5.532049852	315	0.038095238	0.974589093	0.217132116	0.143453618	0
Permethrin	Chorion	1	E	4.771757018	10.53350835	315	0.060758377	0.597196894	0.735231581	0.182144667	0
Permethrin	Chorion	1	R	0.911381068	5.622840042	280	0.025793651	0.999982659	0.566570037	1.643131997	0
Permethrin	Chorion	2.54	B	2.138524895	5.837370601	324	0.067901235	0.443946011	0.624919878	0.41286853	0
Permethrin	Chorion	2.54	E	4.410472022	10.26977344	324	0.052469136	0.763912024	0.373946584	0.092640711	0
Permethrin	Chorion	2.54	R	0.531081144	3.920650738	288	0.010416667	1	0.186270113	0.540209263	0
Permethrin	Chorion	5	B	2.664241827	7.911682004	315	0.069753086	0.418722242	1.150636809	0.760196218	0
Permethrin	Chorion	5	E	3.99415816	9.632278364	315	0.034567901	0.9910585	-0.042367277	-0.010495977	0
Permethrin	Chorion	5	R	1.530335364	6.767851775	280	0.057738095	0.731173045	1.185524333	3.438185635	0
Permethrin	Chorion	8	B	1.696911187	5.49883506	315	0.028835979	0.999364433	0.18330617	0.121105683	0
Permethrin	Chorion	8	E	4.1050071	9.66234369	315	0.051058201	0.799296433	0.068481662	0.016965498	0
Permethrin	Chorion	8	R	1.166837365	6.273096395	280	0.039781746	0.978189912	0.822026334	2.383990826	0
Permethrin	Chorion	12	B	1.208244968	4.67955911	324	0.021604938	0.999999251	-0.305360049	-0.20174355	0
Permethrin	Chorion	12	E	3.878855155	10.25682294	324	0.049382716	0.824393461	-0.157670282	-0.039060891	0
Permethrin	Chorion	12	R	1.447663523	6.811647045	288	0.0625	0.627167051	1.102852492	3.198425785	0
Permethrin	Chorion	16.4	B	2.533235209	7.449899825	324	0.043209877	0.922844972	1.019630192	0.673643507	0
Permethrin	Chorion	16.4	E	3.875238582	11.31534525	324	0.052469136	0.763912024	-0.161286855	-0.039956853	0
Permethrin	Chorion	16.4	R	1.464315855	6.634470949	288	0.079861111	0.317377205	1.119504824	3.246719867	0
Permethrin	Chorion	35	B	2.159690016	6.930719622	324	0.027777778	0.999633292	0.646084998	0.426851782	0
Permethrin	Chorion	35	E	3.286018611	9.144490641	324	0.064814815	0.504120853	-0.750506826	-0.185928923	0
Permethrin	Chorion	35	R	1.499505465	5.605226943	288	0.083333333	0.269999672	1.154694434	3.348774636	0
Permethrin	Standard	0	B	3.981978441	12.17282364	288	0	1	0	0	0
Permethrin	Standard	0	E	26.59565513	43.24530967	288	0	1	0	0	0
Permethrin	Standard	0	R	0.565168611	4.708985416	256	0	1	0	0	0
Permethrin	Standard	1	B	4.682194658	12.36950197	270	0.064814815	0.601765435	0.700216217	0.17584631	0
Permethrin	Standard	1	E	28.66480082	41.99796858	270	0.050925926	0.86272487	2.069145689	0.07780014	0
Permethrin	Standard	1	R	0.280400209	3.115286534	240	0.011458333	1	-0.284768402	-0.503864505	0
Permethrin	Standard	2.54	B	4.742356326	12.34494934	252	0.056547619	0.783347385	0.760377885	0.190954797	0
Permethrin	Standard	2.54	E	27.00789505	43.14962364	252	0.038690476	0.987860766	0.412239924	0.015500273	0
Permethrin	Standard	2.54	R	1.703365072	9.321852768	224	0.0390625	0.99324665	1.138196461	2.013906006	0
Permethrin	Standard	5	B	6.163034656	14.3104551	270	0.124537037	0.026529564	2.181056216	0.547731799	0
Permethrin	Standard	5	E	26.32724001	45.08177057	270	0.063888889	0.620101412	-0.26841512	-0.010092443	0
Permethrin	Standard	5	R	1.417914369	7.628081814	240	0.0390625	0.9915635	0.852745757	1.508834249	0
Permethrin	Standard	8	B	5.815972229	13.05666155	270	0.109027778	0.072807325	1.833993788	0.46057351	0
Permethrin	Standard	8	E	28.53290615	43.1152614	270	0.049074074	0.890432699	1.937251025	0.072840884	0
Permethrin	Standard	8	R	2.483633138	11.3924747	240	0.047395833	0.94358924	1.918464527	3.394499426	0
Permethrin	Standard	12	B	6.86771016	17.2678292	306	0.089869281	0.181932108	2.885731719	0.724697977	0
Permethrin	Standard	12	E	22.70057426	39.52810757	306	0.062908497	0.599884719	-3.895080871	-0.146455534	0
Permethrin	Standard	12	R	2.204685785	10.88839188	272	0.04296875	0.967982405	1.639517173	2.900934589	0
Permethrin	Standard	16.4	B	5.302916749	13.95675689	279	0.058243728	0.722266211	1.320938309	0.331729146	0
Permethrin	Standard	16.4	E	23.51823742	42.93596019	279	0.103494624	0.096057727	-3.077417706	-0.115711295	0
Permethrin	Standard	16.4	R	3.567603232	14.2953962	248	0.073336694	0.507058943	3.002434621	5.312458195	0
Permethrin	Standard	35	B	4.425216226	12.79889351	279	0.036290323	0.992186965	0.443237785	0.111310945	0

Test Agent	Treatment	Concentration	Phase	Movement.peak	Movement.sd	Movement.n	ks.stat	ks.pval	diff	delta	sig
Permethrin	Standard	35	E	18.81541363	37.5244494	279	0.109879032	0.065295339	-7.780241498	-0.292538065	0
Permethrin	Standard	35	R	3.310511795	13.86375947	248	0.065146169	0.658910254	2.745343184	4.857564855	0
Pyrene	Chorion	0	B	1.795454861	5.501947052	324	0	1	0	0	0
Pyrene	Chorion	0	E	6.042363714	12.13017618	324	0	1	0	0	0
Pyrene	Chorion	0	R	0.096224513	1.339747527	288	0	1	0	0	0
Pyrene	Chorion	1	B	1.800595213	5.437052586	315	0.024074074	0.999986581	0.005140352	0.00286298	0
Pyrene	Chorion	1	E	4.79083989	11.26059774	315	0.05952381	0.623363362	-1.251523824	-0.207124874	0
Pyrene	Chorion	1	R	0.312190063	2.490359344	280	0.01468254	1	0.215965549	2.244392231	0
Pyrene	Chorion	5	B	2.926672858	7.146281455	324	0.092592593	0.124323157	1.131217997	0.630045356	0
Pyrene	Chorion	5	E	4.609669764	10.14556626	324	0.070987654	0.387885061	-1.43269395	-0.237108194	0
Pyrene	Chorion	5	R	0.208725246	2.008892096	288	0.013888889	1	0.112500733	1.169148375	0
Pyrene	Chorion	16.5	B	2.664515423	6.248550629	324	0.12654321	0.01116329	0.869060562	0.484033646	0
Pyrene	Chorion	16.5	E	3.21207292	7.402067669	324	0.108024691	0.045605413	-2.830290794	-0.468407883	0
Pyrene	Chorion	16.5	R	0.816062783	3.988543993	288	0.069444444	0.490980372	0.71983827	7.480820093	0
Pyrene	Chorion	30	B	2.491306641	5.832888513	324	0.098765432	0.084803124	0.695851779	0.387562948	0
Pyrene	Chorion	30	E	2.907919122	6.689823327	324	0.108024691	0.045605413	-3.134444592	-0.518744773	0
Pyrene	Chorion	30	R	0.708459954	3.078336681	288	0.079861111	0.317377205	0.612235441	6.362572506	0
Pyrene	Chorion	50	B	2.145088787	5.531225675	324	0.083333333	0.210551633	0.349633926	0.194732785	0
Pyrene	Chorion	50	E	2.781930877	6.672909748	324	0.111111111	0.036631053	-3.260432837	-0.539595594	0
Pyrene	Chorion	50	R	1.10127001	4.412560427	288	0.097222222	0.131419728	1.005045497	10.44479692	0
Pyrene	Chorion	65	B	2.091324889	4.900183608	315	0.095943563	0.105666448	0.295870027	0.164788341	0
Pyrene	Chorion	65	E	3.033234177	6.570228515	315	0.119135802	0.021477993	-3.009129537	-0.498005363	0
Pyrene	Chorion	65	R	0.738854659	3.258229322	280	0.08968254	0.203587694	0.642630146	6.678445296	0
Pyrene	Chorion	100	B	1.888623909	4.612374844	324	0.111111111	0.036631053	0.093169047	0.051891612	0
Pyrene	Chorion	100	E	2.58976634	5.98406805	324	0.12962963	0.008640478	-3.452597374	-0.571398469	1
Pyrene	Chorion	100	R	0.476375556	2.296321479	288	0.083333333	0.269999672	0.380151043	3.950667366	0
Pyrene	Standard	0	B	6.817061581	14.88713194	315	0	1	0	0	0
Pyrene	Standard	0	E	26.69346483	43.53260302	315	0	1	0	0	0
Pyrene	Standard	0	R	1.195668819	7.165270118	280	0	1	0	0	0
Pyrene	Standard	1	B	6.199162825	15.57272893	324	0.045679012	0.892849947	-0.617898756	-0.090640043	0
Pyrene	Standard	1	E	28.947843	45.10049932	324	0.044091711	0.915372032	2.254378166	0.08445431	0
Pyrene	Standard	1	R	1.327703364	8.502607141	288	0.015079365	1	0.132034545	0.110427354	0
Pyrene	Standard	5	B	7.339960382	15.26551542	315	0.034920635	0.990716312	0.522898801	0.076704427	0
Pyrene	Standard	5	E	17.59837998	29.62113266	315	0.085714286	0.19748266	-9.095084847	-0.340723278	0
Pyrene	Standard	5	R	0.627170199	4.788795588	280	0.017857143	1	-0.568498621	-0.475464954	0
Pyrene	Standard	16.5	B	7.886043958	15.1053472	324	0.092328042	0.13131409	1.068982378	0.156809846	0
Pyrene	Standard	16.5	E	8.314579151	15.20181748	324	0.210846561	1.36E-06	-18.37888568	-0.688516301	1
Pyrene	Standard	16.5	R	1.445478356	7.382318444	288	0.023214286	0.9999991	0.249809537	0.208928704	0
Pyrene	Standard	30	B	7.133280653	13.48575118	315	0.095238095	0.114843478	0.316219072	0.046386419	0
Pyrene	Standard	30	E	6.90448631	14.06391401	315	0.219047619	5.46E-07	-19.78897852	-0.741341697	1
Pyrene	Standard	30	R	2.148902724	9.71001492	280	0.028571429	0.999847967	0.953233904	0.797239076	0
Pyrene	Standard	50	B	6.786931911	12.30052908	306	0.098039216	0.101187039	-0.03012967	-0.004419744	0
Pyrene	Standard	50	E	6.37708852	13.14305127	306	0.236321195	5.91E-08	-20.31637631	-0.761099259	1
Pyrene	Standard	50	R	2.40398751	7.88942321	272	0.078151261	0.368403171	1.20831869	1.010579745	0
Pyrene	Standard	65	B	5.790619369	12.09688596	315	0.06984127	0.425981642	-1.026442212	-0.150569597	0
Pyrene	Standard	65	E	6.003499059	12.47512073	315	0.241269841	2.18E-08	-20.68996577	-0.7750948	1

Test Agent	Treatment	Concentration	Phase	Movement.peak	Movement.sd	Movement.n	ks.stat	ks.pval	diff	delta	sig
Pyrene	Standard	65	R	1.454613031	5.942695346	280	0.032142857	0.998697677	0.258944212	0.216568508	0
Pyrene	Standard	100	B	4.403112699	9.877537322	306	0.085434174	0.207249321	-2.413948882	-0.354104016	0
Pyrene	Standard	100	E	4.363469201	10.29318276	306	0.278711485	6.73E-11	-22.32999563	-0.836534177	1
Pyrene	Standard	100	R	1.233149085	5.747329089	272	0.026680672	0.99997196	0.037480265	0.031346695	0
Retene	Chorion	0	B	2.532104802	6.899548366	315	0	1	0	0	0
Retene	Chorion	0	E	5.799644472	11.596248	315	0	1	0	0	0
Retene	Chorion	0	R	0.233134969	1.823314661	280	0	1	0	0	0
Retene	Chorion	1	B	2.781126796	7.292364562	324	0.030070547	0.998713475	0.249021994	0.098345848	0
Retene	Chorion	1	E	6.215236928	12.70760248	324	0.029717813	0.998938421	0.415592456	0.071658264	0
Retene	Chorion	1	R	0.115288623	1.578525163	288	0.014484127	1	-0.117846345	-0.505485496	0
Retene	Chorion	5	B	3.032367727	8.170703023	324	0.026719577	0.999851456	0.500262924	0.197568017	0
Retene	Chorion	5	E	6.820598913	12.83890317	324	0.056084656	0.696546508	1.020954441	0.176037418	0
Retene	Chorion	5	R	0.63220375	4.174253245	288	0.017261905	1	0.399068781	1.711749995	0
Retene	Chorion	20	B	3.121449831	7.27842646	324	0.063756614	0.534809345	0.589345029	0.232749066	0
Retene	Chorion	20	E	5.956830663	12.07894186	324	0.029717813	0.998938421	0.157186191	0.027102729	0
Retene	Chorion	20	R	0.46175696	2.839182787	288	0.020734127	0.999999983	0.228621992	0.980642213	0
Retene	Chorion	30	B	2.866191388	8.185021731	324	0.020546737	0.999999891	0.334086585	0.131940268	0
Retene	Chorion	30	E	5.611014907	12.20918857	324	0.031128748	0.997800426	-0.188629565	-0.032524332	0
Retene	Chorion	30	R	0.273301914	2.505093921	288	0.009821429	1	0.040166946	0.172290524	0
Retene	Chorion	45	B	2.814683987	6.458077659	324	0.057848325	0.65905591	0.282579184	0.111598534	0
Retene	Chorion	45	E	5.643768249	11.19376047	324	0.028218695	0.999569347	-0.155876223	-0.026876858	0
Retene	Chorion	45	R	0.679826096	3.373366064	288	0.037599206	0.988024825	0.446691127	1.91601942	0
Retene	Chorion	65	B	2.790235461	6.893397203	324	0.05696649	0.677834504	0.258130659	0.101943118	0
Retene	Chorion	65	E	5.997352823	12.75480947	324	0.028659612	0.99942981	0.197708351	0.034089736	0
Retene	Chorion	65	R	0.416093923	3.104190081	288	0.016765873	1	0.182958954	0.78477697	0
Retene	Chorion	100	B	2.663845385	6.629556556	324	0.041534392	0.945745673	0.131740582	0.052028092	0
Retene	Chorion	100	E	5.165800788	10.37004089	324	0.028395062	0.999517396	-0.633843684	-0.109290093	0
Retene	Chorion	100	R	0.583224957	4.00938456	288	0.017261905	1	0.350089989	1.501662281	0
Retene	Standard	0	B	4.393852808	12.26609985	288	0	1	0	0	0
Retene	Standard	0	E	20.04974683	36.30153242	288	0	1	0	0	0
Retene	Standard	0	R	0.047471814	0.759549023	256	0	1	0	0	0
Retene	Standard	1	B	5.463926667	13.54054663	216	0.045138889	0.962986917	1.070073859	0.24353885	0
Retene	Standard	1	E	23.48331481	42.36033778	216	0.072916667	0.527812621	3.433567978	0.171252436	0
Retene	Standard	1	R	0.002373589	0.023433324	192	0.006510417	1	-0.045098225	-0.950000039	0
Retene	Standard	5	B	4.971306422	13.14688453	270	0.036805556	0.991626622	0.577453614	0.131423067	0
Retene	Standard	5	E	17.45643664	36.02403205	270	0.083101852	0.290944013	-2.593310191	-0.129343787	0
Retene	Standard	5	R	1.88413259	12.08899558	240	0.041927083	0.9813987	1.836660776	38.68950063	0
Retene	Standard	20	B	4.867667155	12.83402959	288	0.027777778	0.999886732	0.473814347	0.107835735	0
Retene	Standard	20	E	10.63465993	23.27327232	288	0.128472222	0.017243402	-9.415086901	-0.469586324	0
Retene	Standard	20	R	8.48E-05	0.001356336	256	0.00390625	1	-0.047387043	-0.998214288	0
Retene	Standard	30	B	4.095614435	9.980906751	270	0.044444444	0.945950808	-0.298238372	-0.067876277	0
Retene	Standard	30	E	6.807242647	15.72111411	270	0.15	0.003780797	-13.24250418	-0.660482364	1
Retene	Standard	30	R	0	0	240	0.00390625	1	-0.047471814	-1	0
Retene	Standard	45	B	3.248120313	9.363279752	297	0.053135522	0.803510777	-1.145732495	-0.260758051	0
Retene	Standard	45	E	8.970711312	23.67013311	297	0.179292929	0.000165371	-11.07903552	-0.552577327	1
Retene	Standard	45	R	8.22E-05	0.001335627	264	0.00390625	1	-0.047389612	-0.9982684	0

Test Agent	Treatment	Concentration	Phase	Movement.peak	Movement.sd	Movement.n	ks.stat	ks.pval	diff	delta	sig
Retene	Standard	65	B	2.03406005	7.014705706	207	0.070048309	0.595740327	-2.359792758	-0.537066866	0
Retene	Standard	65	E	2.567682773	10.0701865	207	0.272795894	3.28E-08	-17.48206405	-0.871934404	1
Retene	Standard	65	R	0.467287681	3.19965479	184	0.02173913	1	0.419815867	8.84347642	0
Retene	Standard	100	B	1.57467679	5.125940106	180	0.081944444	0.446619876	-2.819176018	-0.64161822	0
Retene	Standard	100	E	1.194299861	7.627606909	180	0.309722222	1.18E-09	-18.85544697	-0.94043317	1
Retene	Standard	100	R	0.1357694	1.513866324	160	0.00859375	1	0.088297586	1.860000262	0

Abamectin

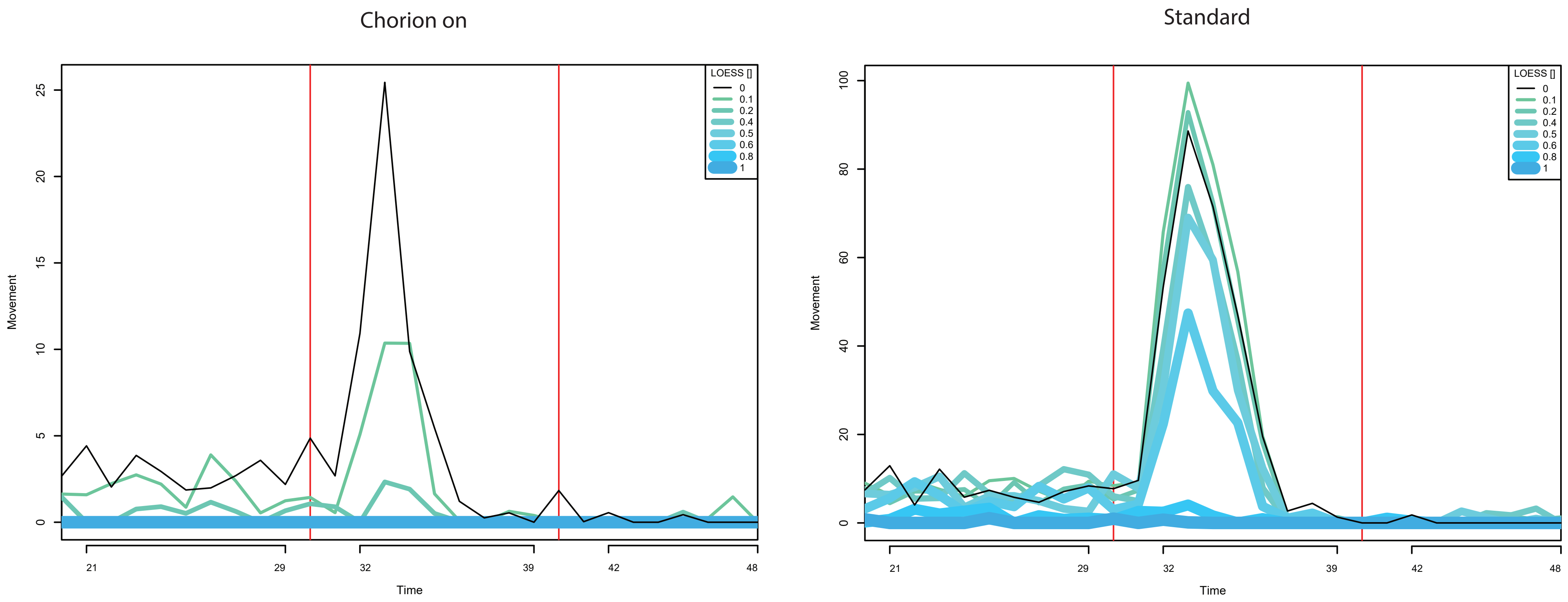

Chorion on
Standard
LOESS []
0
0.1
0.2
0.4
0.5
0.6
0.8
1
Movement
Time
21
29
32
39
42
48
0
5
10
15
20
25
40
60
80
100

Chlorpyrifos

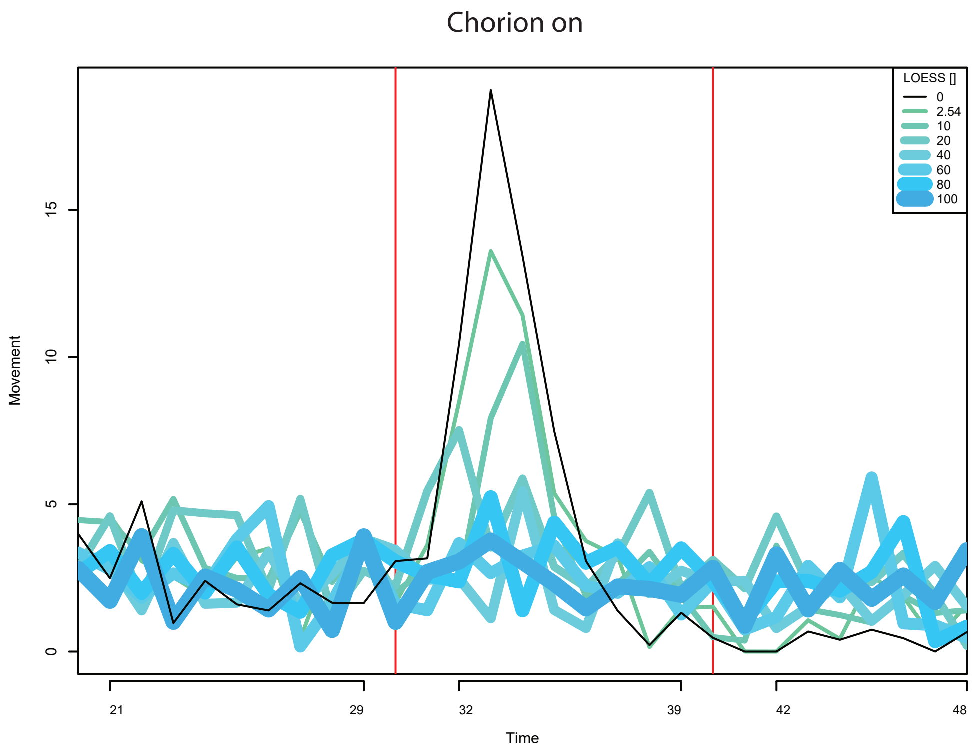

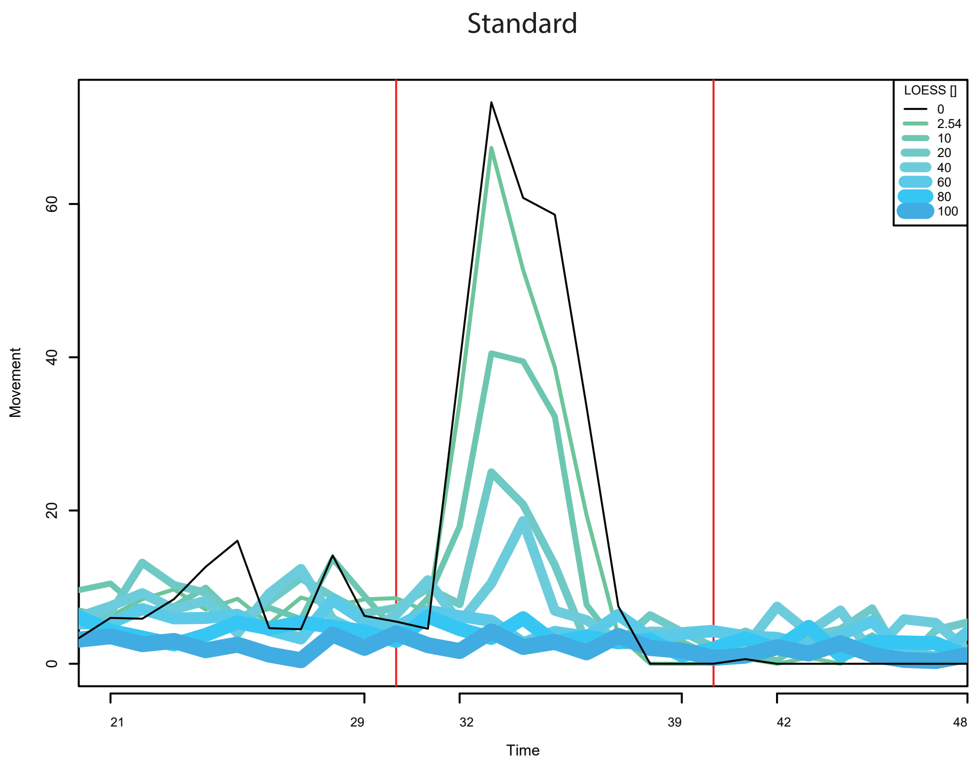

Estradiol

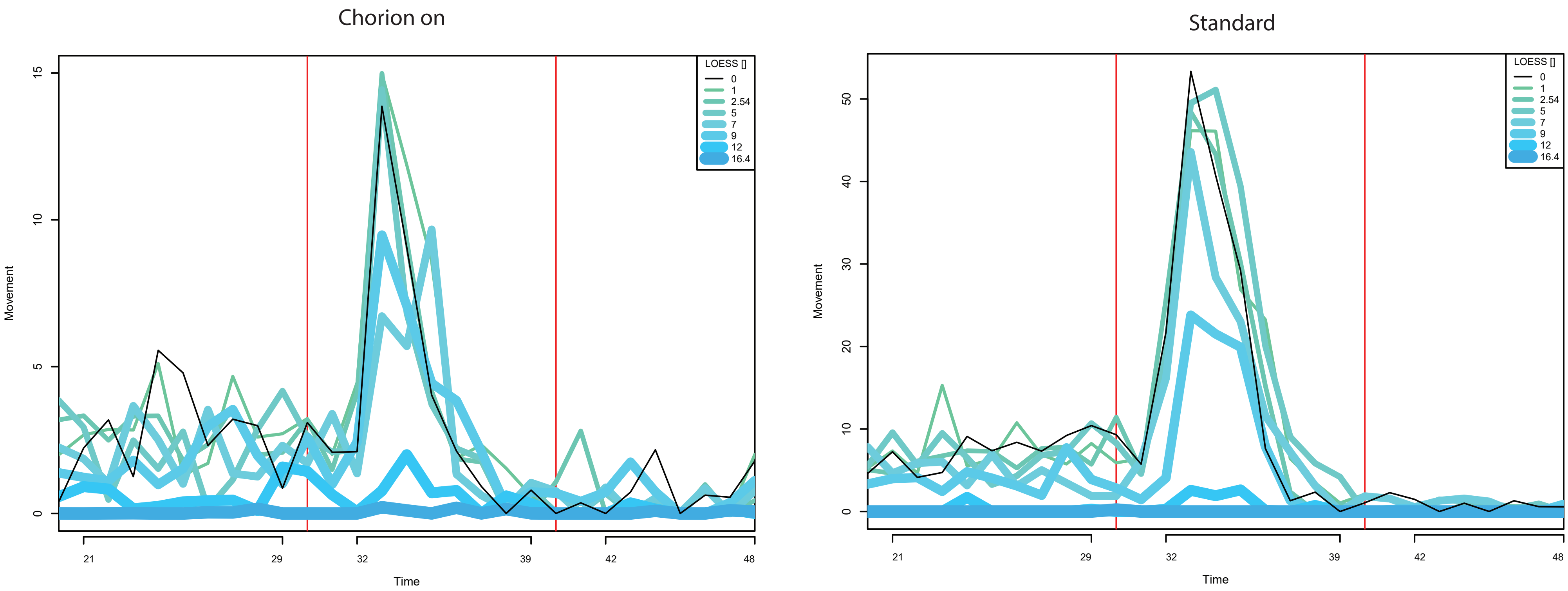
Chorion on
Standard
LOESS []
0
1
2.54
5
7
9
12
16.4
Movement
Time
0
5
10
15
20
30
40
50
21
29
32
39
42
48

Hydroxyurea

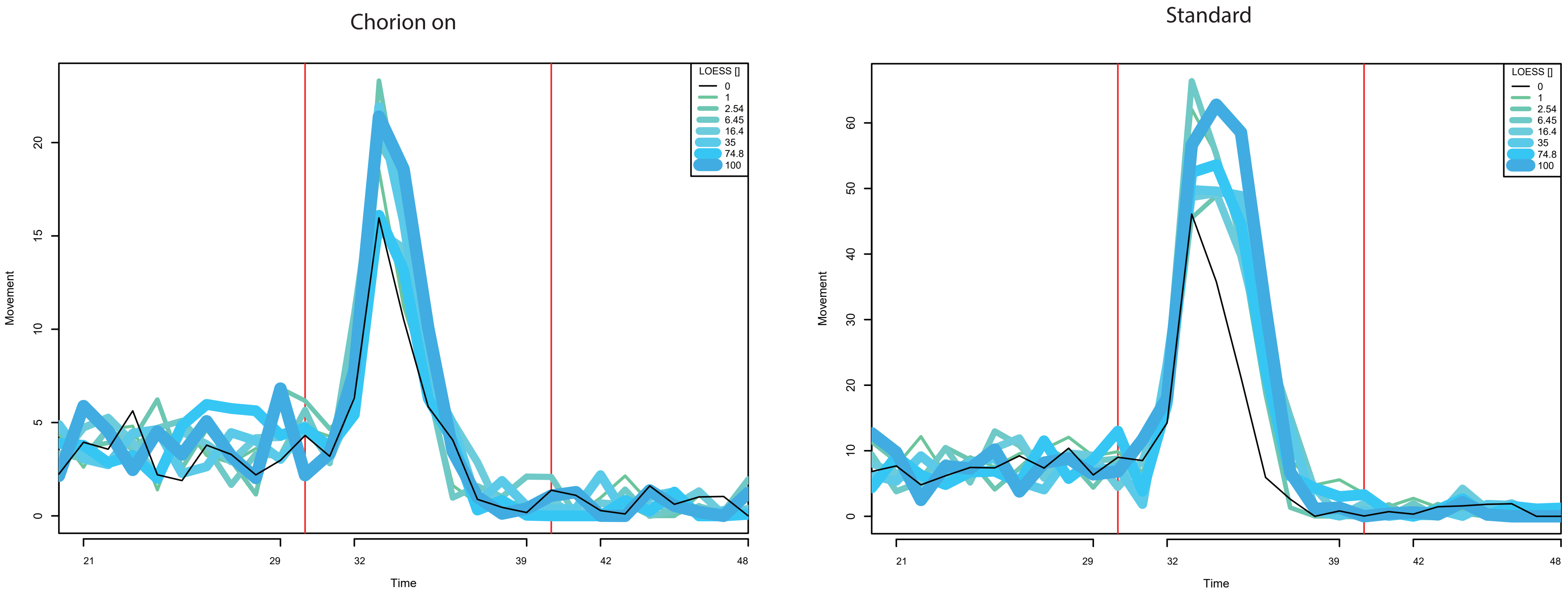

MWCNTs

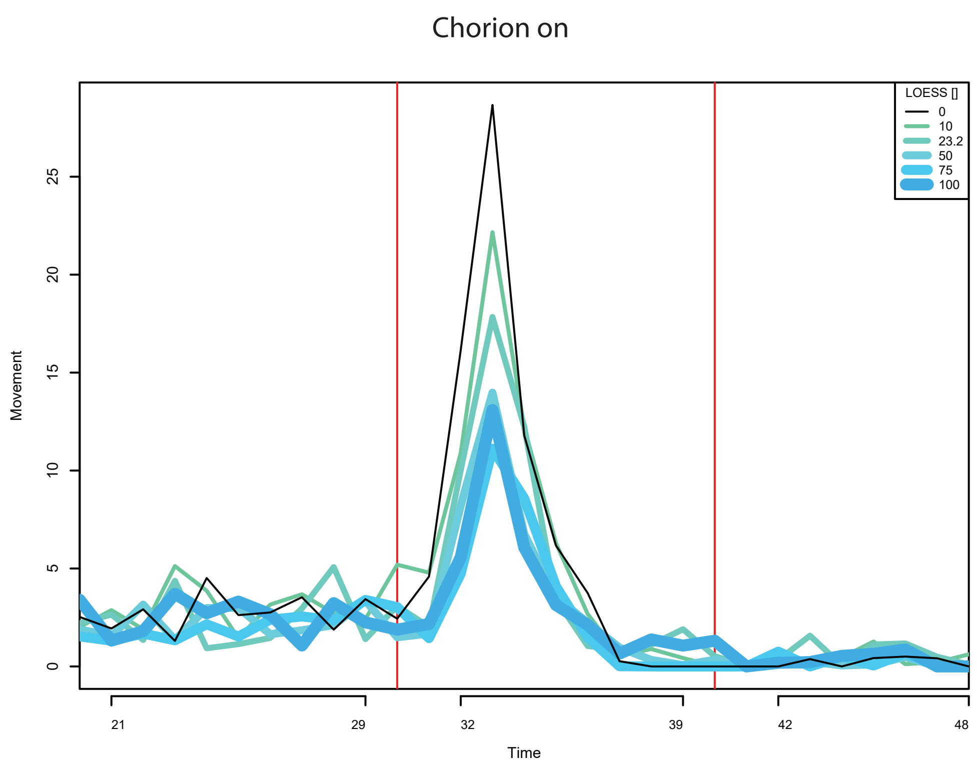

Chorion on
Movement
Time
LOESS []
0
10
23.2
50
75
100
0
5
10
15
20
25
21
29
32
39
42
48

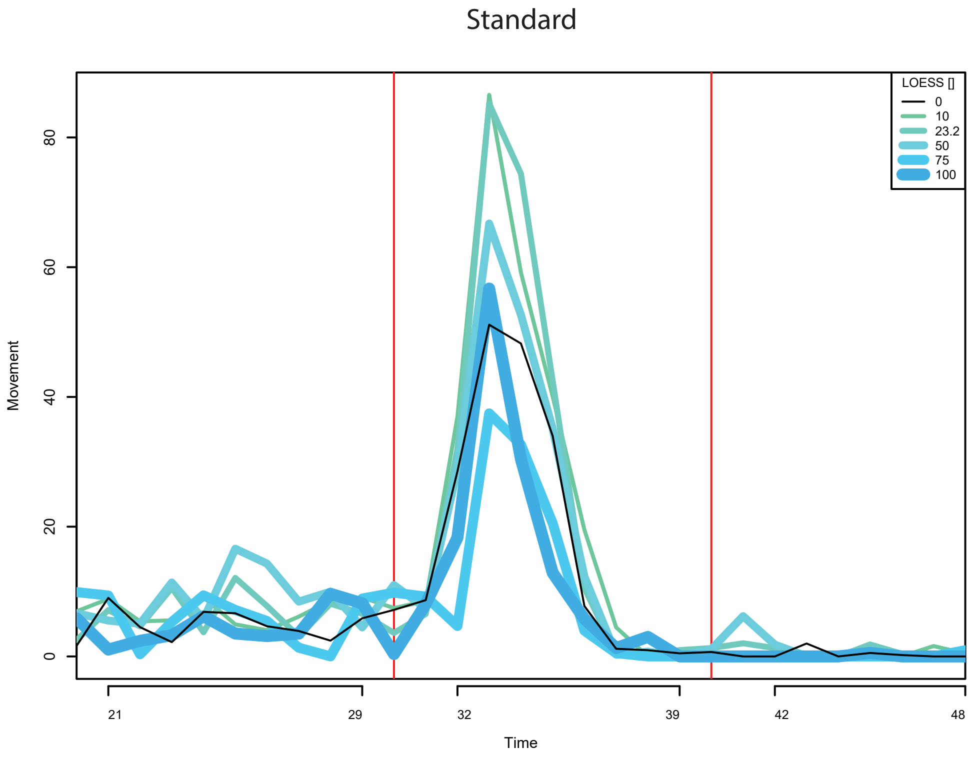

Standard
Movement
Time
LOESS []
0
10
23.2
50
75
100
0
20
40
60
80
21
29
32
39
42
48

Napthalene

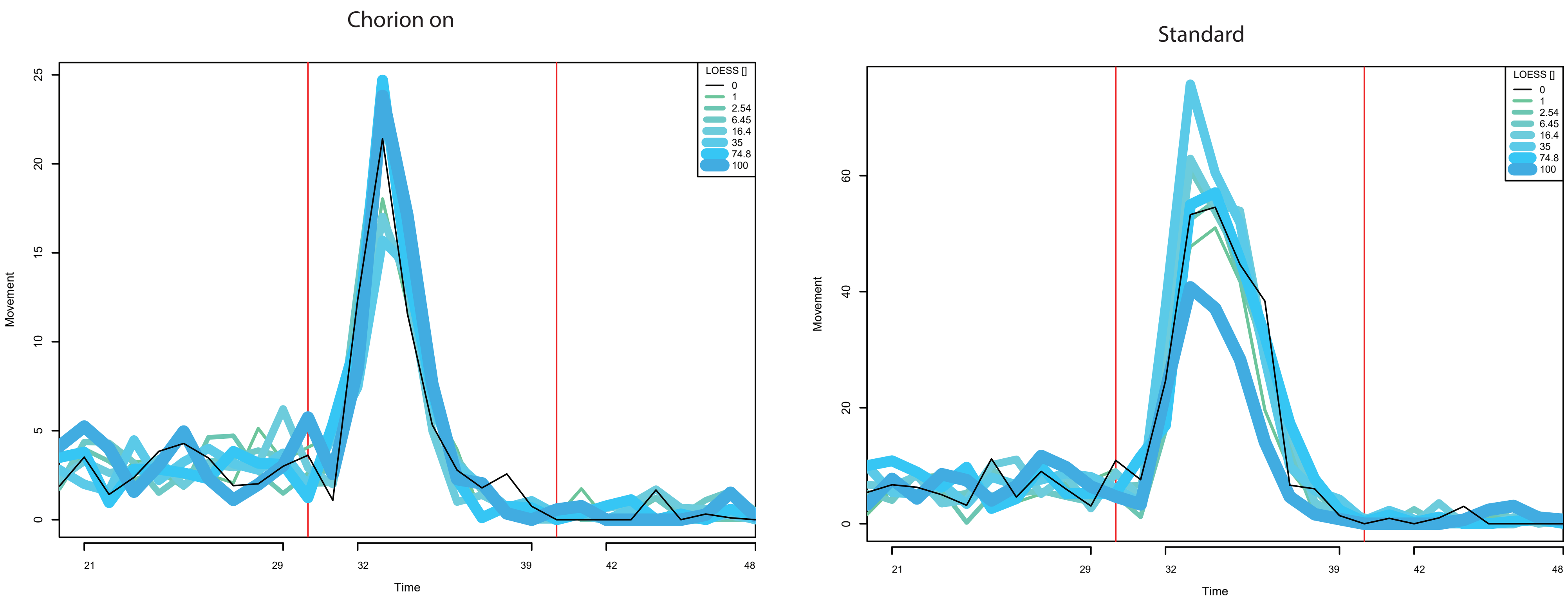

Chorion on
Standard
LOESS []
0
1
2.54
6.45
16.4
35
74.8
100
Movement
Time
0
5
10
15
20
25
40
60
21
29
32
39
42
48

Permethrin

Chorion on

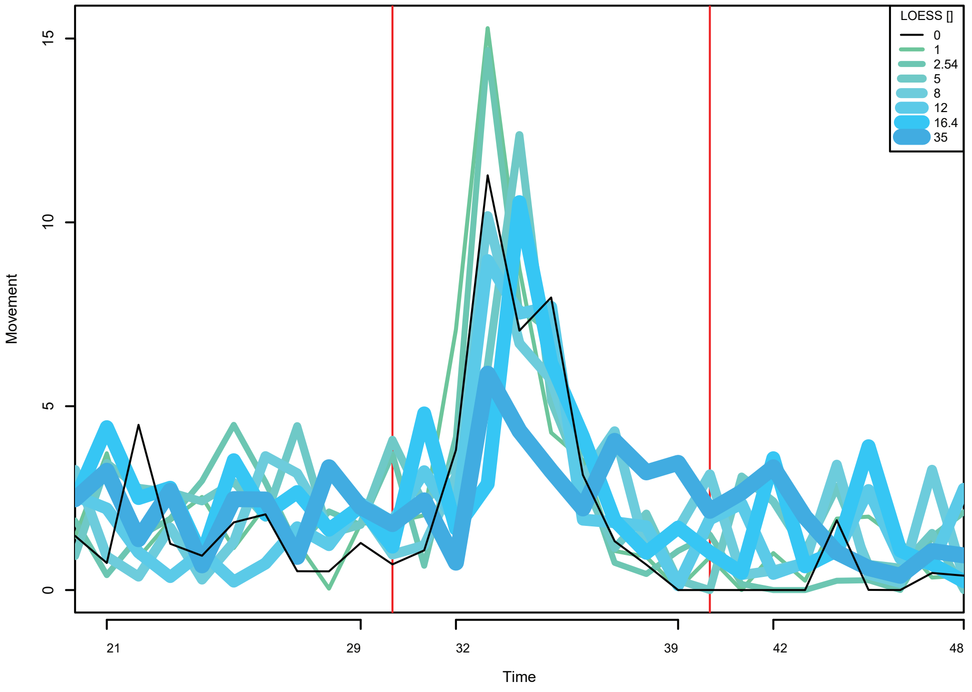

Standard

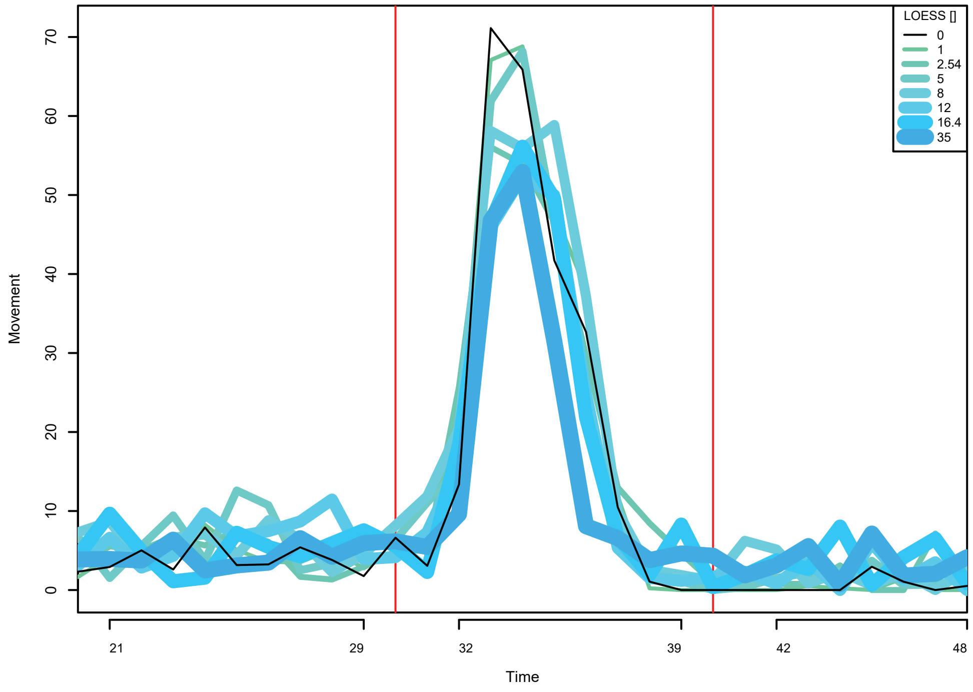

Pyrene

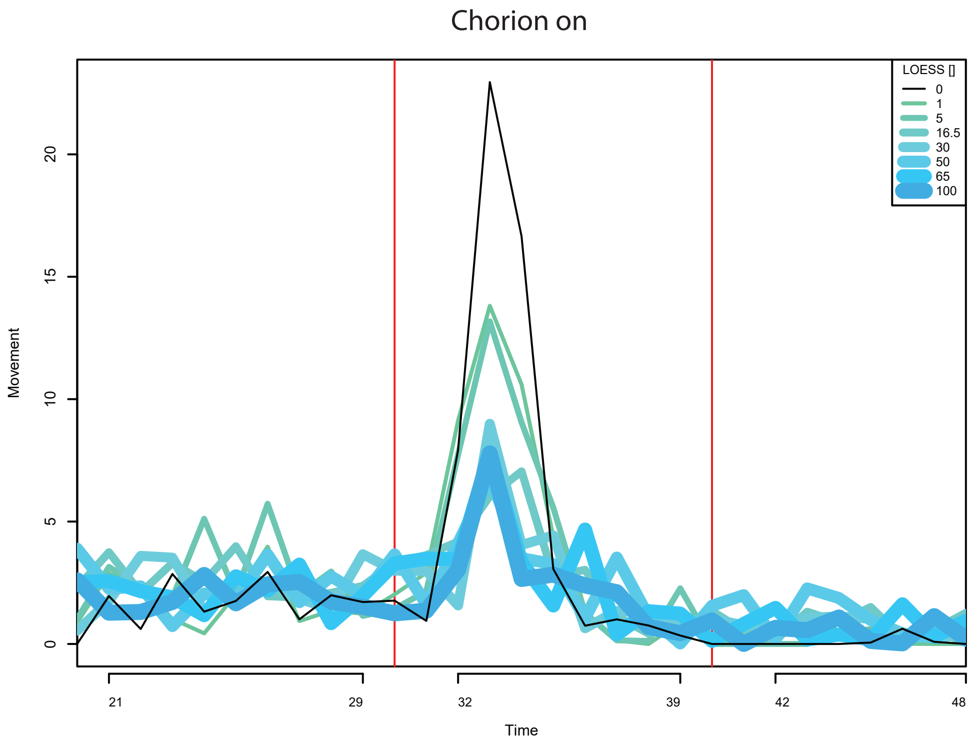

Chorion on
Movement
0
5
10
15
20
LOESS []
0
1
5
16.5
30
50
65
100
21
29
32
39
42
48
Time

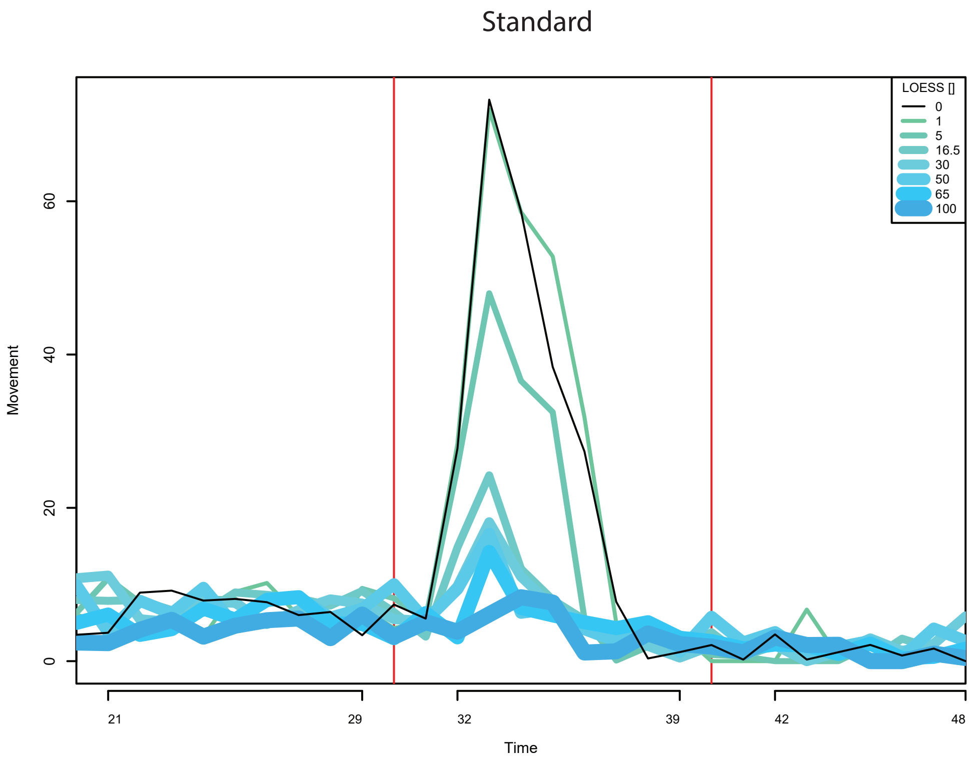

Standard
Movement
0
20
40
60
LOESS []
0
1
5
16.5
30
50
65
100
21
29
32
39
42
48
Time

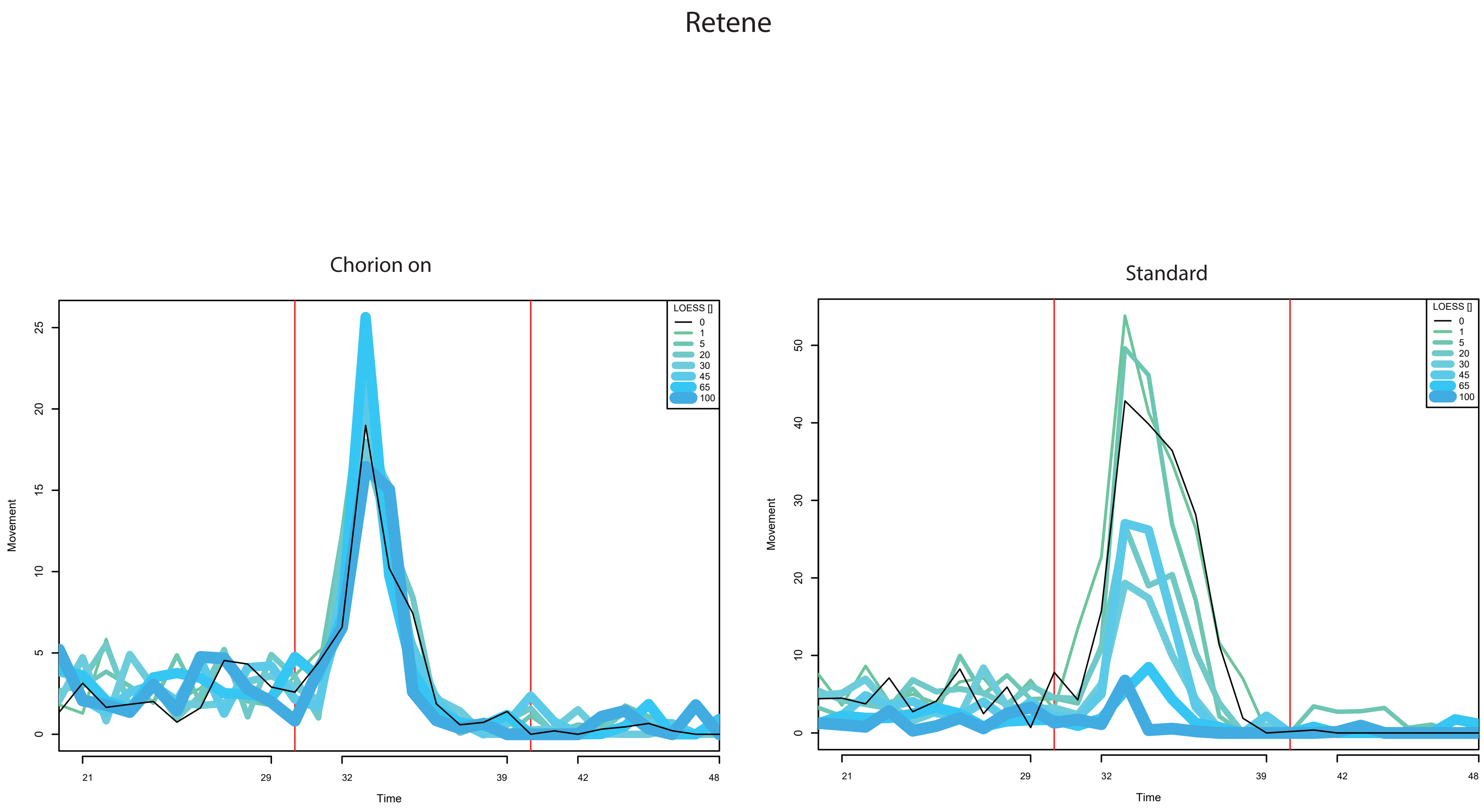

Figure S2. Embryonic photomotor response (EPR) compiled data for each concentration of each test agent under chorion-on and standard conditions. Each panel shows movement (dimensionless index) versus time (sec). For all chemicals, concentration units are micromolar. For MWCNTs, concentration units are micrograms per milliliter.

Table S3. Larval photomotor response (LPR) compiled data. DR = daily renewal; LD = light/dark cycle. For all chemicals, concentration units are micromolar. For MWCNTs, concentration units are micrograms per milliliter.

Test Agent	Treatment	Concentration	Phase	# viable animals	# total animals	% viable animals	More than 70% viable	interval	AUC	Pval	RelativeRatio	Significance p<0.01	activity	Sig
Abamectin	Chorion	0	Dark	34	36	0.94	TRUE	Dark	18.788	1	0	NA	NA	NA
Abamectin	Chorion	0.1	Dark	27	36	0.75	TRUE	Dark	8.913	0	-1.197	YES	HYPO	YES
Abamectin	Chorion	0.2	Dark	0	36	0	FALSE	Dark	NA	NA	NA	NA	NA	NA
Abamectin	Chorion	0.4	Dark	0	36	0	FALSE	Dark	NA	NA	NA	NA	NA	NA
Abamectin	Chorion	0.5	Dark	0	36	0	FALSE	Dark	NA	NA	NA	NA	NA	NA
Abamectin	Chorion	0.6	Dark	0	36	0	FALSE	Dark	NA	NA	NA	NA	NA	NA
Abamectin	Chorion	0.8	Dark	0	36	0	FALSE	Dark	NA	NA	NA	NA	NA	NA
Abamectin	Chorion	1	Dark	0	36	0	FALSE	Dark	NA	NA	NA	NA	NA	NA
Abamectin	Chorion	0	Light	34	36	0.94	TRUE	Light	-4.074	1	0	NA	NA	NA
Abamectin	Chorion	0.1	Light	27	36	0.75	TRUE	Light	14.617	0.02	-2.54	NA	NA	NA
Abamectin	Chorion	0.2	Light	0	36	0	FALSE	Light	NA	NA	NA	NA	NA	NA
Abamectin	Chorion	0.4	Light	0	36	0	FALSE	Light	NA	NA	NA	NA	NA	NA
Abamectin	Chorion	0.5	Light	0	36	0	FALSE	Light	NA	NA	NA	NA	NA	NA
Abamectin	Chorion	0.6	Light	0	36	0	FALSE	Light	NA	NA	NA	NA	NA	NA
Abamectin	Chorion	0.8	Light	0	36	0	FALSE	Light	NA	NA	NA	NA	NA	NA
Abamectin	Chorion	1	Light	0	36	0	FALSE	Light	NA	NA	NA	NA	NA	NA
Abamectin	DR	0	Dark	36	36	1	TRUE	Dark	15.007	1	0	NA	NA	NA
Abamectin	DR	0.1	Dark	36	36	1	TRUE	Dark	35.944	0	0.237	YES	HYPER	YES
Abamectin	DR	0.2	Dark	34	36	0.94	TRUE	Dark	40.985	0	0.101	YES	HYPER	YES
Abamectin	DR	0.4	Dark	2	36	0.06	FALSE	Dark	NA	NA	NA	NA	NA	NA
Abamectin	DR	0.5	Dark	0	36	0	FALSE	Dark	NA	NA	NA	NA	NA	NA
Abamectin	DR	0.6	Dark	0	36	0	FALSE	Dark	NA	NA	NA	NA	NA	NA
Abamectin	DR	0.8	Dark	0	36	0	FALSE	Dark	NA	NA	NA	NA	NA	NA
Abamectin	DR	1	Dark	3	36	0.08	FALSE	Dark	NA	NA	NA	NA	NA	NA
Abamectin	DR	0	Light	36	36	1	TRUE	Light	-38.31	1	0	NA	NA	NA
Abamectin	DR	0.1	Light	36	36	1	TRUE	Light	19.752	0	-1.763	YES	HYPO	YES
Abamectin	DR	0.2	Light	34	36	0.94	TRUE	Light	34.14	0	-1.703	YES	HYPO	YES
Abamectin	DR	0.4	Light	2	36	0.06	FALSE	Light	NA	NA	NA	NA	NA	NA
Abamectin	DR	0.5	Light	0	36	0	FALSE	Light	NA	NA	NA	NA	NA	NA
Abamectin	DR	0.6	Light	0	36	0	FALSE	Light	NA	NA	NA	NA	NA	NA
Abamectin	DR	0.8	Light	0	36	0	FALSE	Light	NA	NA	NA	NA	NA	NA
Abamectin	DR	1	Light	3	36	0.08	FALSE	Light	NA	NA	NA	NA	NA	NA
Abamectin	LD	0	Dark	31	36	0.86	TRUE	Dark	29.135	1	0	NA	NA	NA
Abamectin	LD	0.1	Dark	29	36	0.81	TRUE	Dark	39.385	0	-0.169	YES	HYPO	YES
Abamectin	LD	0.2	Dark	29	36	0.81	TRUE	Dark	38.23	0	-0.097	YES	NA	NA
Abamectin	LD	0.4	Dark	12	36	0.33	FALSE	Dark	NA	NA	NA	NA	NA	NA
Abamectin	LD	0.5	Dark	8	36	0.22	FALSE	Dark	NA	NA	NA	NA	NA	NA
Abamectin	LD	0.6	Dark	7	36	0.19	FALSE	Dark	NA	NA	NA	NA	NA	NA
Abamectin	LD	0.8	Dark	1	36	0.03	FALSE	Dark	NA	NA	NA	NA	NA	NA
Abamectin	LD	1	Dark	3	36	0.08	FALSE	Dark	NA	NA	NA	NA	NA	NA
Abamectin	LD	0	Light	31	36	0.86	TRUE	Light	8.145	1	0	NA	NA	NA
Abamectin	LD	0.1	Light	29	36	0.81	TRUE	Light	38.621	0	6.071	YES	HYPER	YES
Abamectin	LD	0.2	Light	29	36	0.81	TRUE	Light	36.826	0.151	2.264	NA	NA	NA
Abamectin	LD	0.4	Light	12	36	0.33	FALSE	Light	NA	NA	NA	NA	NA	NA
Abamectin	LD	0.5	Light	8	36	0.22	FALSE	Light	NA	NA	NA	NA	NA	NA
Abamectin	LD	0.6	Light	7	36	0.19	FALSE	Light	NA	NA	NA	NA	NA	NA
Abamectin	LD	0.8	Light	1	36	0.03	FALSE	Light	NA	NA	NA	NA	NA	NA
Abamectin	LD	1	Light	3	36	0.08	FALSE	Light	NA	NA	NA	NA	NA	NA
Abamectin	Standard	0	Dark	35	36	0.97	TRUE	Dark	17.691	1	0	NA	NA	NA
Abamectin	Standard	0.1	Dark	33	36	0.92	TRUE	Dark	20.252	0	0.043	YES	NA	NA
Abamectin	Standard	0.2	Dark	32	36	0.89	TRUE	Dark	31.294	0.227	0.366	NA	NA	NA
Abamectin	Standard	0.4	Dark	31	36	0.86	TRUE	Dark	38.047	0	0.015	YES	NA	NA
Abamectin	Standard	0.5	Dark	32	36	0.89	TRUE	Dark	40.69	0	0.014	YES	NA	NA
Abamectin	Standard	0.6	Dark	19	36	0.53	FALSE	Dark	NA	NA	NA	NA	NA	NA

Test Agent	Treatment	Concentration	Phase	# viable animals	# total animals	% viable animals	More than 70% viable	interval	AUC	Pval	RelativeRatio	Significance p<0.01	activity	Sig
Abamectin	Chorion	0	Dark	34	36	0.94	TRUE	Dark	18.788	1	0	NA	NA	NA
Abamectin	Chorion	0.1	Dark	27	36	0.75	TRUE	Dark	8.913	0	-1.197	YES	HYPO	YES
Abamectin	Chorion	0.2	Dark	0	36	0	FALSE	Dark	NA	NA	NA	NA	NA	NA
Abamectin	Chorion	0.4	Dark	0	36	0	FALSE	Dark	NA	NA	NA	NA	NA	NA
Abamectin	Chorion	0.5	Dark	0	36	0	FALSE	Dark	NA	NA	NA	NA	NA	NA
Abamectin	Chorion	0.6	Dark	0	36	0	FALSE	Dark	NA	NA	NA	NA	NA	NA
Abamectin	Chorion	0.8	Dark	0	36	0	FALSE	Dark	NA	NA	NA	NA	NA	NA
Abamectin	Chorion	1	Dark	0	36	0	FALSE	Dark	NA	NA	NA	NA	NA	NA
Abamectin	Chorion	0	Light	34	36	0.94	TRUE	Light	-4.074	1	0	NA	NA	NA
Abamectin	Chorion	0.1	Light	27	36	0.75	TRUE	Light	14.617	0.02	-2.54	NA	NA	NA
Abamectin	Chorion	0.2	Light	0	36	0	FALSE	Light	NA	NA	NA	NA	NA	NA
Abamectin	Chorion	0.4	Light	0	36	0	FALSE	Light	NA	NA	NA	NA	NA	NA
Abamectin	Chorion	0.5	Light	0	36	0	FALSE	Light	NA	NA	NA	NA	NA	NA
Abamectin	Chorion	0.6	Light	0	36	0	FALSE	Light	NA	NA	NA	NA	NA	NA
Abamectin	Chorion	0.8	Light	0	36	0	FALSE	Light	NA	NA	NA	NA	NA	NA
Abamectin	Chorion	1	Light	0	36	0	FALSE	Light	NA	NA	NA	NA	NA	NA
Abamectin	DR	0	Dark	36	36	1	TRUE	Dark	15.007	1	0	NA	NA	NA
Abamectin	DR	0.1	Dark	36	36	1	TRUE	Dark	35.944	0	0.237	YES	HYPER	YES
Abamectin	DR	0.2	Dark	34	36	0.94	TRUE	Dark	40.985	0	0.101	YES	HYPER	YES
Abamectin	DR	0.4	Dark	2	36	0.06	FALSE	Dark	NA	NA	NA	NA	NA	NA
Abamectin	DR	0.5	Dark	0	36	0	FALSE	Dark	NA	NA	NA	NA	NA	NA
Abamectin	DR	0.6	Dark	0	36	0	FALSE	Dark	NA	NA	NA	NA	NA	NA
Abamectin	DR	0.8	Dark	0	36	0	FALSE	Dark	NA	NA	NA	NA	NA	NA
Abamectin	DR	1	Dark	3	36	0.08	FALSE	Dark	NA	NA	NA	NA	NA	NA
Abamectin	DR	0	Light	36	36	1	TRUE	Light	-38.311	1	0	NA	NA	NA
Abamectin	DR	0.1	Light	36	36	1	TRUE	Light	19.752	0	-1.763	YES	HYPO	YES
Abamectin	DR	0.2	Light	34	36	0.94	TRUE	Light	34.14	0	-1.703	YES	HYPO	YES
Abamectin	DR	0.4	Light	2	36	0.06	FALSE	Light	NA	NA	NA	NA	NA	NA
Abamectin	DR	0.5	Light	0	36	0	FALSE	Light	NA	NA	NA	NA	NA	NA
Abamectin	DR	0.6	Light	0	36	0	FALSE	Light	NA	NA	NA	NA	NA	NA
Abamectin	DR	0.8	Light	0	36	0	FALSE	Light	NA	NA	NA	NA	NA	NA
Abamectin	DR	1	Light	3	36	0.08	FALSE	Light	NA	NA	NA	NA	NA	NA
Abamectin	LD	0	Dark	31	36	0.86	TRUE	Dark	29.135	1	0	NA	NA	NA
Abamectin	LD	0.1	Dark	29	36	0.81	TRUE	Dark	39.385	0	-0.169	YES	HYPO	YES
Abamectin	LD	0.2	Dark	29	36	0.81	TRUE	Dark	38.23	0	-0.097	YES	NA	NA
Abamectin	LD	0.4	Dark	12	36	0.33	FALSE	Dark	NA	NA	NA	NA	NA	NA
Abamectin	LD	0.5	Dark	8	36	0.22	FALSE	Dark	NA	NA	NA	NA	NA	NA
Abamectin	LD	0.6	Dark	7	36	0.19	FALSE	Dark	NA	NA	NA	NA	NA	NA
Abamectin	LD	0.8	Dark	1	36	0.03	FALSE	Dark	NA	NA	NA	NA	NA	NA
Abamectin	LD	1	Dark	3	36	0.08	FALSE	Dark	NA	NA	NA	NA	NA	NA
Abamectin	LD	0	Light	31	36	0.86	TRUE	Light	8.145	1	0	NA	NA	NA
Abamectin	LD	0.1	Light	29	36	0.81	TRUE	Light	38.621	0	6.071	YES	HYPER	YES
Abamectin	LD	0.2	Light	29	36	0.81	TRUE	Light	36.826	0.151	2.264	NA	NA	NA
Abamectin	LD	0.4	Light	12	36	0.33	FALSE	Light	NA	NA	NA	NA	NA	NA
Abamectin	LD	0.5	Light	8	36	0.22	FALSE	Light	NA	NA	NA	NA	NA	NA
Abamectin	LD	0.6	Light	7	36	0.19	FALSE	Light	NA	NA	NA	NA	NA	NA
Abamectin	LD	0.8	Light	1	36	0.03	FALSE	Light	NA	NA	NA	NA	NA	NA
Abamectin	LD	1	Light	3	36	0.08	FALSE	Light	NA	NA	NA	NA	NA	NA
Abamectin	Standard	0	Dark	35	36	0.97	TRUE	Dark	17.691	1	0	NA	NA	NA
Abamectin	Standard	0.1	Dark	33	36	0.92	TRUE	Dark	20.252	0	0.043	YES	NA	NA
Abamectin	Standard	0.2	Dark	32	36	0.89	TRUE	Dark	31.294	0.227	0.366	NA	NA	NA
Abamectin	Standard	0.4	Dark	31	36	0.86	TRUE	Dark	38.047	0	0.015	YES	NA	NA
Abamectin	Standard	0.5	Dark	32	36	0.89	TRUE	Dark	40.69	0	0.014	YES	NA	NA
Abamectin	Standard	0.6	Dark	19	36	0.53	FALSE	Dark	NA	NA	NA	NA	NA	NA
Abamectin	Standard	0.8	Dark	0	36	0	FALSE	Dark	NA	NA	NA	NA	NA	NA
Abamectin	Standard	1	Dark	3	36	0.08	FALSE	Dark	NA	NA	NA	NA	NA	NA
Abamectin	Standard	0	Light	35	36	0.97	TRUE	Light	-24.634	1	0	NA	NA	NA
Abamectin	Standard	0.1	Light	33	36	0.92	TRUE	Light	6.146	0.986	-0.312	NA	NA	NA
Abamectin	Standard	0.2	Light	32	36	0.89	TRUE	Light	21.16	0.151	-2.006	NA	NA	NA
Abamectin	Standard	0.4	Light	31	36	0.86	TRUE	Light	34.78	0.74	-3.261	NA	NA	NA
Abamectin	Standard	0.5	Light	32	36	0.89	TRUE	Light	38.453	0.003	-2.693	YES	HYPO	YES
Abamectin	Standard	0.6	Light	19	36	0.53	FALSE	Light	NA	NA	NA	NA	NA	NA
Abamectin	Standard	0.8	Light	0	36	0	FALSE	Light	NA	NA	NA	NA	NA	NA
Abamectin	Standard	1	Light	3	36	0.08	FALSE	Light	NA	NA	NA	NA	NA	NA
Chlorpyrifos	Chorion	0	Dark	36	36	1	TRUE	Dark	42.466	1	0	NA	NA	NA

Test Agent	Treatment	Concentration	Phase	# viable animals	# total animals	% viable animals	More than 70% viable	interval	AUC	Pval	RelativeRatio	Significance p<0.01	activity	Sig
Chlorpyrifos	Chorion	2.54	Dark	35	36	0.97	TRUE	Dark	49.252	0	-0.244	YES	HYPO	YES
Chlorpyrifos	Chorion	10	Dark	36	36	1	TRUE	Dark	32.58	0	-1.302	YES	HYPO	YES
Chlorpyrifos	Chorion	20	Dark	35	36	0.97	TRUE	Dark	22.445	0	-1.248	YES	HYPO	YES
Chlorpyrifos	Chorion	40	Dark	24	36	0.67	FALSE	Dark	21.326	0	-1.11	NA	NA	NA
Chlorpyrifos	Chorion	60	Dark	7	36	0.19	FALSE	Dark	NA	NA	NA	NA	NA	NA
Chlorpyrifos	Chorion	80	Dark	0	36	0	FALSE	Dark	NA	NA	NA	NA	NA	NA
Chlorpyrifos	Chorion	100	Dark	0	36	0	FALSE	Dark	NA	NA	NA	NA	NA	NA
Chlorpyrifos	Chorion	0	Light	36	36	1	TRUE	Light	36.859	1	0	NA	NA	NA
Chlorpyrifos	Chorion	2.54	Light	35	36	0.97	TRUE	Light	53.549	0.286	1.054	NA	NA	NA
Chlorpyrifos	Chorion	10	Light	36	36	1	TRUE	Light	41.997	0	-0.648	YES	HYPO	YES
Chlorpyrifos	Chorion	20	Light	35	36	0.97	TRUE	Light	27.352	0.032	-1.109	NA	NA	NA
Chlorpyrifos	Chorion	40	Light	24	36	0.67	FALSE	Light	24.281	0.002	0.057	NA	NA	NA
Chlorpyrifos	Chorion	60	Light	7	36	0.19	FALSE	Light	NA	NA	NA	NA	NA	NA
Chlorpyrifos	Chorion	80	Light	0	36	0	FALSE	Light	NA	NA	NA	NA	NA	NA
Chlorpyrifos	Chorion	100	Light	0	36	0	FALSE	Light	NA	NA	NA	NA	NA	NA
Chlorpyrifos	DR	0	Dark	33	36	0.92	TRUE	Dark	42.995	1	0	NA	NA	NA
Chlorpyrifos	DR	2.54	Dark	27	36	0.75	TRUE	Dark	31.232	0	-0.648	YES	HYPO	YES
Chlorpyrifos	DR	10	Dark	2	36	0.06	FALSE	Dark	NA	NA	NA	NA	NA	NA
Chlorpyrifos	DR	20	Dark	2	36	0.06	FALSE	Dark	NA	NA	NA	NA	NA	NA
Chlorpyrifos	DR	40	Dark	2	36	0.06	FALSE	Dark	NA	NA	NA	NA	NA	NA
Chlorpyrifos	DR	60	Dark	2	36	0.06	FALSE	Dark	NA	NA	NA	NA	NA	NA
Chlorpyrifos	DR	80	Dark	2	36	0.06	FALSE	Dark	NA	NA	NA	NA	NA	NA
Chlorpyrifos	DR	100	Dark	1	36	0.03	FALSE	Dark	NA	NA	NA	NA	NA	NA
Chlorpyrifos	DR	0	Light	33	36	0.92	TRUE	Light	25.051	1	0	NA	NA	NA
Chlorpyrifos	DR	2.54	Light	27	36	0.75	TRUE	Light	34.502	0.286	-0.301	NA	NA	NA
Chlorpyrifos	DR	10	Light	2	36	0.06	FALSE	Light	NA	NA	NA	NA	NA	NA
Chlorpyrifos	DR	20	Light	2	36	0.06	FALSE	Light	NA	NA	NA	NA	NA	NA
Chlorpyrifos	DR	40	Light	2	36	0.06	FALSE	Light	NA	NA	NA	NA	NA	NA
Chlorpyrifos	DR	60	Light	2	36	0.06	FALSE	Light	NA	NA	NA	NA	NA	NA
Chlorpyrifos	DR	80	Light	2	36	0.06	FALSE	Light	NA	NA	NA	NA	NA	NA
Chlorpyrifos	DR	100	Light	1	36	0.03	FALSE	Light	NA	NA	NA	NA	NA	NA
Chlorpyrifos	LD	0	Dark	32	36	0.89	TRUE	Dark	22.684	1	0	NA	NA	NA
Chlorpyrifos	LD	2.54	Dark	32	36	0.89	TRUE	Dark	28.754	0.872	0.099	NA	NA	NA
Chlorpyrifos	LD	10	Dark	26	36	0.72	TRUE	Dark	27.93	0	-0.22	YES	HYPO	YES
Chlorpyrifos	LD	20	Dark	19	36	0.53	FALSE	Dark	NA	NA	NA	NA	NA	NA
Chlorpyrifos	LD	40	Dark	13	36	0.36	FALSE	Dark	NA	NA	NA	NA	NA	NA
Chlorpyrifos	LD	60	Dark	0	36	0	FALSE	Dark	NA	NA	NA	NA	NA	NA
Chlorpyrifos	LD	80	Dark	0	36	0	FALSE	Dark	NA	NA	NA	NA	NA	NA
Chlorpyrifos	LD	100	Dark	0	36	0	FALSE	Dark	NA	NA	NA	NA	NA	NA
Chlorpyrifos	LD	0	Light	32	36	0.89	TRUE	Light	-8.238	1	0	NA	NA	NA
Chlorpyrifos	LD	2.54	Light	32	36	0.89	TRUE	Light	18.7	0.151	-2.303	NA	NA	NA
Chlorpyrifos	LD	10	Light	26	36	0.72	TRUE	Light	25.148	0	-2.302	YES	HYPO	YES
Chlorpyrifos	LD	20	Light	19	36	0.53	FALSE	Light	NA	NA	NA	NA	NA	NA
Chlorpyrifos	LD	40	Light	13	36	0.36	FALSE	Light	NA	NA	NA	NA	NA	NA
Chlorpyrifos	LD	60	Light	0	36	0	FALSE	Light	NA	NA	NA	NA	NA	NA
Chlorpyrifos	LD	80	Light	0	36	0	FALSE	Light	NA	NA	NA	NA	NA	NA
Chlorpyrifos	LD	100	Light	0	36	0	FALSE	Light	NA	NA	NA	NA	NA	NA
Chlorpyrifos	Standard	0	Dark	34	36	0.94	TRUE	Dark	-9.436	1	0	NA	NA	NA
Chlorpyrifos	Standard	2.54	Dark	35	36	0.97	TRUE	Dark	-8.463	0.055	0.548	NA	NA	NA
Chlorpyrifos	Standard	10	Dark	36	36	1	TRUE	Dark	3.226	0.001	2.073	YES	HYPER	YES
Chlorpyrifos	Standard	20	Dark	32	36	0.89	TRUE	Dark	8.935	0	1.333	YES	HYPER	YES
Chlorpyrifos	Standard	40	Dark	36	36	1	TRUE	Dark	7.736	0	-0.279	YES	HYPO	YES
Chlorpyrifos	Standard	60	Dark	22	36	0.61	FALSE	Dark	4.482	0	-0.745	NA	NA	NA
Chlorpyrifos	Standard	80	Dark	6	36	0.17	FALSE	Dark	NA	NA	NA	NA	NA	NA
Chlorpyrifos	Standard	100	Dark	1	36	0.03	FALSE	Dark	NA	NA	NA	NA	NA	NA
Chlorpyrifos	Standard	0	Light	34	36	0.94	TRUE	Light	-51.981	1	0	NA	NA	NA
Chlorpyrifos	Standard	2.54	Light	35	36	0.97	TRUE	Light	-28.373	1	0.274	NA	NA	NA
Chlorpyrifos	Standard	10	Light	36	36	1	TRUE	Light	-16.479	0.286	-0.066	NA	NA	NA
Chlorpyrifos	Standard	20	Light	32	36	0.89	TRUE	Light	-0.825	0.379	-0.517	NA	NA	NA
Chlorpyrifos	Standard	40	Light	36	36	1	TRUE	Light	5.943	0.856	-0.654	NA	NA	NA
Chlorpyrifos	Standard	60	Light	22	36	0.61	FALSE	Light	4.123	0.614	-0.384	NA	NA	NA
Chlorpyrifos	Standard	80	Light	6	36	0.17	FALSE	Light	NA	NA	NA	NA	NA	NA
Chlorpyrifos	Standard	100	Light	1	36	0.03	FALSE	Light	NA	NA	NA	NA	NA	NA
Estradiol	Chorion	0	Dark	31	36	0.86	TRUE	Dark	24.966	1	0	NA	NA	NA
Estradiol	Chorion	1	Dark	32	36	0.89	TRUE	Dark	27.105	0	0.322	YES	HYPER	YES

Test Agent	Treatment	Concentration	Phase	# viable animals	# total animals	% viable animals	More than 70% viable	interval	AUC	Pval	RelativeRatio	Significance p<0.01	activity	Sig
Estradiol	Chorion	2.54	Dark	33	36	0.92	TRUE	Dark	29.147	0	-0.81	YES	HYPO	YES
Estradiol	Chorion	5	Dark	33	36	0.92	TRUE	Dark	27.293	0.023	-0.026	NA	NA	NA
Estradiol	Chorion	7	Dark	33	36	0.92	TRUE	Dark	27.33	0	-0.224	YES	HYPO	YES
Estradiol	Chorion	9	Dark	30	36	0.83	TRUE	Dark	22.596	0	-1.139	YES	HYPO	YES
Estradiol	Chorion	12	Dark	4	36	0.11	FALSE	Dark	NA	NA	NA	NA	NA	NA
Estradiol	Chorion	16.4	Dark	3	36	0.08	FALSE	Dark	NA	NA	NA	NA	NA	NA
Estradiol	Chorion	0	Light	31	36	0.86	TRUE	Light	4.858	1	0	NA	NA	NA
Estradiol	Chorion	1	Light	32	36	0.89	TRUE	Light	16.28	0.614	-1.225	NA	NA	NA
Estradiol	Chorion	2.54	Light	33	36	0.92	TRUE	Light	33.265	0.614	10.919	NA	NA	NA
Estradiol	Chorion	5	Light	33	36	0.92	TRUE	Light	24.92	0	-1.091	YES	HYPO	YES
Estradiol	Chorion	7	Light	33	36	0.92	TRUE	Light	26.478	0.003	3.109	YES	HYPER	YES
Estradiol	Chorion	9	Light	30	36	0.83	TRUE	Light	25.221	0	-0.149	YES	HYPO	YES
Estradiol	Chorion	12	Light	4	36	0.11	FALSE	Light	NA	NA	NA	NA	NA	NA
Estradiol	Chorion	16.4	Light	3	36	0.08	FALSE	Light	NA	NA	NA	NA	NA	NA
Estradiol	DR	0	Dark	30	36	0.83	TRUE	Dark	4.586	1	0	NA	NA	NA
Estradiol	DR	1	Dark	31	36	0.86	TRUE	Dark	12.789	0.036	0.768	NA	NA	NA
Estradiol	DR	2.54	Dark	28	36	0.78	TRUE	Dark	16.464	0.001	0.627	YES	HYPER	YES
Estradiol	DR	5	Dark	28	36	0.78	TRUE	Dark	20.628	0	0.614	YES	HYPER	YES
Estradiol	DR	7	Dark	22	36	0.61	FALSE	Dark	18.979	0	-0.453	NA	NA	NA
Estradiol	DR	9	Dark	11	36	0.31	FALSE	Dark	NA	NA	NA	NA	NA	NA
Estradiol	DR	12	Dark	1	36	0.03	FALSE	Dark	NA	NA	NA	NA	NA	NA
Estradiol	DR	16.4	Dark	0	36	0	FALSE	Dark	NA	NA	NA	NA	NA	NA
Estradiol	DR	0	Light	30	36	0.83	TRUE	Light	-23.831	1	0	NA	NA	NA
Estradiol	DR	1	Light	31	36	0.86	TRUE	Light	-2.396	0.379	-0.314	NA	NA	NA
Estradiol	DR	2.54	Light	28	36	0.78	TRUE	Light	9.015	0.856	-0.745	NA	NA	NA
Estradiol	DR	5	Light	28	36	0.78	TRUE	Light	15.963	0.614	-1.544	NA	NA	NA
Estradiol	DR	7	Light	22	36	0.61	FALSE	Light	19.083	0	-1.282	NA	NA	NA
Estradiol	DR	9	Light	11	36	0.31	FALSE	Light	NA	NA	NA	NA	NA	NA
Estradiol	DR	12	Light	1	36	0.03	FALSE	Light	NA	NA	NA	NA	NA	NA
Estradiol	DR	16.4	Light	0	36	0	FALSE	Light	NA	NA	NA	NA	NA	NA
Estradiol	LD	0	Dark	33	36	0.92	TRUE	Dark	36.401	1	0	NA	NA	NA
Estradiol	LD	1	Dark	34	36	0.94	TRUE	Dark	38.902	0	0.167	YES	HYPER	YES
Estradiol	LD	2.54	Dark	34	36	0.94	TRUE	Dark	35.576	0	-0.148	YES	HYPO	YES
Estradiol	LD	5	Dark	31	36	0.86	TRUE	Dark	36.719	0	0.401	YES	HYPER	YES
Estradiol	LD	7	Dark	25	36	0.69	FALSE	Dark	34.096	0	-0.339	NA	NA	NA
Estradiol	LD	9	Dark	20	36	0.56	FALSE	Dark	NA	NA	NA	NA	NA	NA
Estradiol	LD	12	Dark	0	36	0	FALSE	Dark	NA	NA	NA	NA	NA	NA
Estradiol	LD	16.4	Dark	0	36	0	FALSE	Dark	NA	NA	NA	NA	NA	NA
Estradiol	LD	0	Light	33	36	0.92	TRUE	Light	17.925	1	0	NA	NA	NA
Estradiol	LD	1	Light	34	36	0.94	TRUE	Light	30.528	0	0.048	YES	NA	NA
Estradiol	LD	2.54	Light	34	36	0.94	TRUE	Light	33.338	0.008	-0.382	YES	HYPO	YES
Estradiol	LD	5	Light	31	36	0.86	TRUE	Light	30.984	0.072	-0.808	NA	NA	NA
Estradiol	LD	7	Light	25	36	0.69	FALSE	Light	33.855	0.49	-0.389	NA	NA	NA
Estradiol	LD	9	Light	20	36	0.56	FALSE	Light	NA	NA	NA	NA	NA	NA
Estradiol	LD	12	Light	0	36	0	FALSE	Light	NA	NA	NA	NA	NA	NA
Estradiol	LD	16.4	Light	0	36	0	FALSE	Light	NA	NA	NA	NA	NA	NA
Estradiol	Standard	0	Dark	34	36	0.94	TRUE	Dark	34.436	1	0	NA	NA	NA
Estradiol	Standard	1	Dark	31	36	0.86	TRUE	Dark	33.331	0	0.089	YES	NA	NA
Estradiol	Standard	2.54	Dark	33	36	0.92	TRUE	Dark	28.468	0	-0.184	YES	HYPO	YES
Estradiol	Standard	5	Dark	30	36	0.83	TRUE	Dark	31.587	0.164	-0.016	NA	NA	NA
Estradiol	Standard	7	Dark	26	36	0.72	TRUE	Dark	35.023	0.226	0.267	NA	NA	NA
Estradiol	Standard	9	Dark	11	36	0.31	FALSE	Dark	NA	NA	NA	NA	NA	NA
Estradiol	Standard	12	Dark	0	36	0	FALSE	Dark	NA	NA	NA	NA	NA	NA
Estradiol	Standard	16.4	Dark	0	36	0	FALSE	Dark	NA	NA	NA	NA	NA	NA
Estradiol	Standard	0	Light	34	36	0.94	TRUE	Light	10.326	1	0	NA	NA	NA
Estradiol	Standard	1	Light	31	36	0.86	TRUE	Light	23.187	0.21	-0.933	NA	NA	NA
Estradiol	Standard	2.54	Light	33	36	0.92	TRUE	Light	24.609	0.614	-1.995	NA	NA	NA
Estradiol	Standard	5	Light	30	36	0.83	TRUE	Light	27.873	0.001	1.353	YES	HYPER	YES
Estradiol	Standard	7	Light	26	36	0.72	TRUE	Light	30.672	0.379	1.262	NA	NA	NA
Estradiol	Standard	9	Light	11	36	0.31	FALSE	Light	NA	NA	NA	NA	NA	NA
Estradiol	Standard	12	Light	0	36	0	FALSE	Light	NA	NA	NA	NA	NA	NA
Estradiol	Standard	16.4	Light	0	36	0	FALSE	Light	NA	NA	NA	NA	NA	NA
Hydroxyurea	Chorion	0	Dark	34	36	0.94	TRUE	Dark	16.944	1	0	NA	NA	NA
Hydroxyurea	Chorion	1	Dark	36	36	1	TRUE	Dark	30.995	0.036	0.166	NA	NA	NA
Hydroxyurea	Chorion	2.54	Dark	35	36	0.97	TRUE	Dark	34.094	0.306	0.288	NA	NA	NA

Test Agent	Treatment	Concentration	Phase	# viable animals	# total animals	% viable animals	More than 70% viable	interval	AUC	Pval	RelativeRatio	Significance p<0.01	activity	Sig
Hydroxyurea	Chorion	6.45	Dark	35	36	0.97	TRUE	Dark	39.043	0.002	0.354	YES	HYPER	YES
Hydroxyurea	Chorion	16.4	Dark	35	36	0.97	TRUE	Dark	33.567	0.015	0.194	NA	NA	NA
Hydroxyurea	Chorion	35	Dark	33	36	0.92	TRUE	Dark	34.181	0.001	0.082	YES	NA	NA
Hydroxyurea	Chorion	74.8	Dark	34	36	0.94	TRUE	Dark	38.109	0.054	0.264	NA	NA	NA
Hydroxyurea	Chorion	100	Dark	34	36	0.94	TRUE	Dark	36.158	0.515	0.462	NA	NA	NA
Hydroxyurea	Chorion	0	Light	34	36	0.94	TRUE	Light	-24.817	1	0	NA	NA	NA
Hydroxyurea	Chorion	1	Light	36	36	1	TRUE	Light	18.518	0	-1.873	YES	HYPO	YES
Hydroxyurea	Chorion	2.54	Light	35	36	0.97	TRUE	Light	25.795	0	-1.201	YES	HYPO	YES
Hydroxyurea	Chorion	6.45	Light	35	36	0.97	TRUE	Light	33.269	0	-2.141	YES	HYPO	YES
Hydroxyurea	Chorion	16.4	Light	35	36	0.97	TRUE	Light	29.506	0	0.885	YES	HYPER	YES
Hydroxyurea	Chorion	35	Light	33	36	0.92	TRUE	Light	31.515	0.02	-1.443	NA	NA	NA
Hydroxyurea	Chorion	74.8	Light	34	36	0.94	TRUE	Light	35.334	0	-2.981	YES	HYPO	YES
Hydroxyurea	Chorion	100	Light	34	36	0.94	TRUE	Light	32.847	0.008	0.645	YES	HYPER	YES
Hydroxyurea	DR	0	Dark	32	36	0.89	TRUE	Dark	66.685	1	0	NA	NA	NA
Hydroxyurea	DR	1	Dark	31	36	0.86	TRUE	Dark	73.538	0.762	0.531	NA	NA	NA
Hydroxyurea	DR	2.54	Dark	31	36	0.86	TRUE	Dark	62.561	0.001	0.174	YES	HYPER	YES
Hydroxyurea	DR	6.45	Dark	32	36	0.89	TRUE	Dark	56.29	0.116	0.081	NA	NA	NA
Hydroxyurea	DR	16.4	Dark	33	36	0.92	TRUE	Dark	60.659	0	0.357	YES	HYPER	YES
Hydroxyurea	DR	35	Dark	32	36	0.89	TRUE	Dark	58.283	0.023	0.236	NA	NA	NA
Hydroxyurea	DR	74.8	Dark	32	36	0.89	TRUE	Dark	59.741	0.001	0.225	YES	HYPER	YES
Hydroxyurea	DR	100	Dark	32	36	0.89	TRUE	Dark	59.145	0.116	0.275	NA	NA	NA
Hydroxyurea	DR	0	Light	32	36	0.89	TRUE	Light	60.093	1	0	NA	NA	NA
Hydroxyurea	DR	1	Light	31	36	0.86	TRUE	Light	60.647	0.032	-0.192	NA	NA	NA
Hydroxyurea	DR	2.54	Light	31	36	0.86	TRUE	Light	57.87	0.072	-1.08	NA	NA	NA
Hydroxyurea	DR	6.45	Light	32	36	0.89	TRUE	Light	53.016	0.856	-1.071	NA	NA	NA
Hydroxyurea	DR	16.4	Light	33	36	0.92	TRUE	Light	56.353	0.003	-0.054	YES	NA	NA
Hydroxyurea	DR	35	Light	32	36	0.89	TRUE	Light	55.351	0.105	-0.962	NA	NA	NA
Hydroxyurea	DR	74.8	Light	32	36	0.89	TRUE	Light	57.43	0	-0.215	YES	HYPO	YES
Hydroxyurea	DR	100	Light	32	36	0.89	TRUE	Light	56.86	0.49	-0.725	NA	NA	NA
Hydroxyurea	LD	0	Dark	30	36	0.83	TRUE	Dark	34.076	1	0	NA	NA	NA
Hydroxyurea	LD	1	Dark	27	36	0.75	TRUE	Dark	41.667	0.227	0.013	NA	NA	NA
Hydroxyurea	LD	2.54	Dark	33	36	0.92	TRUE	Dark	41.943	0.638	-0.064	NA	NA	NA
Hydroxyurea	LD	6.45	Dark	27	36	0.75	TRUE	Dark	41.852	0.402	0.026	NA	NA	NA
Hydroxyurea	LD	16.4	Dark	24	36	0.67	FALSE	Dark	41.586	0.227	0.076	NA	NA	NA
Hydroxyurea	LD	35	Dark	30	36	0.83	TRUE	Dark	43.292	0.402	0.212	NA	NA	NA
Hydroxyurea	LD	74.8	Dark	31	36	0.86	TRUE	Dark	45.497	0.638	0.256	NA	NA	NA
Hydroxyurea	LD	100	Dark	30	36	0.83	TRUE	Dark	45.816	0.402	0.17	NA	NA	NA
Hydroxyurea	LD	0	Light	30	36	0.83	TRUE	Light	10.981	1	0	NA	NA	NA
Hydroxyurea	LD	1	Light	27	36	0.75	TRUE	Light	36.253	0.005	2.698	YES	HYPER	YES
Hydroxyurea	LD	2.54	Light	33	36	0.92	TRUE	Light	39.629	0	1.867	YES	HYPER	YES
Hydroxyurea	LD	6.45	Light	27	36	0.75	TRUE	Light	39.454	0.003	1.233	YES	HYPER	YES
Hydroxyurea	LD	16.4	Light	24	36	0.67	FALSE	Light	39.374	0	0.78	NA	NA	NA
Hydroxyurea	LD	35	Light	30	36	0.83	TRUE	Light	40.929	0	2.129	YES	HYPER	YES
Hydroxyurea	LD	74.8	Light	31	36	0.86	TRUE	Light	43.472	0.013	3.156	NA	NA	NA
Hydroxyurea	LD	100	Light	30	36	0.83	TRUE	Light	44.412	0.032	1.66	NA	NA	NA
Hydroxyurea	Standard	0	Dark	32	36	0.89	TRUE	Dark	31.302	1	0	NA	NA	NA
Hydroxyurea	Standard	1	Dark	32	36	0.89	TRUE	Dark	40.288	0.009	-0.009	YES	NA	NA
Hydroxyurea	Standard	2.54	Dark	34	36	0.94	TRUE	Dark	45.368	0.762	0.033	NA	NA	NA
Hydroxyurea	Standard	6.45	Dark	30	36	0.83	TRUE	Dark	38.796	0.306	0.051	NA	NA	NA
Hydroxyurea	Standard	16.4	Dark	31	36	0.86	TRUE	Dark	43.66	0.515	0.152	NA	NA	NA
Hydroxyurea	Standard	35	Dark	33	36	0.92	TRUE	Dark	46.969	0.001	0.138	YES	HYPER	YES
Hydroxyurea	Standard	74.8	Dark	31	36	0.86	TRUE	Dark	44.44	0.95	0.149	NA	NA	NA
Hydroxyurea	Standard	100	Dark	28	36	0.78	TRUE	Dark	39.251	0.002	-0.13	YES	HYPO	YES
Hydroxyurea	Standard	0	Light	32	36	0.89	TRUE	Light	-11.047	1	0	NA	NA	NA
Hydroxyurea	Standard	1	Light	32	36	0.89	TRUE	Light	29.384	0	-3.312	YES	HYPO	YES
Hydroxyurea	Standard	2.54	Light	34	36	0.94	TRUE	Light	39.23	0	-4.168	YES	HYPO	YES
Hydroxyurea	Standard	6.45	Light	30	36	0.83	TRUE	Light	33.275	0.379	2.556	NA	NA	NA
Hydroxyurea	Standard	16.4	Light	31	36	0.86	TRUE	Light	39.085	0	-4.748	YES	HYPO	YES
Hydroxyurea	Standard	35	Light	33	36	0.92	TRUE	Light	43.616	0.008	-4.909	YES	HYPO	YES
Hydroxyurea	Standard	74.8	Light	31	36	0.86	TRUE	Light	41.348	0.013	1.362	NA	NA	NA
Hydroxyurea	Standard	100	Light	28	36	0.78	TRUE	Light	37.596	0.21	4.271	NA	NA	NA
MWCNTs	Chorion	0	Dark	43	48	0.9	TRUE	Dark	14.749	1	0	NA	NA	NA
MWCNTs	Chorion	10	Dark	48	48	1	TRUE	Dark	19.849	0.055	0.089	NA	NA	NA
MWCNTs	Chorion	23.2	Dark	43	48	0.9	TRUE	Dark	19.815	0.023	-0.25	NA	NA	NA
MWCNTs	Chorion	50	Dark	47	48	0.98	TRUE	Dark	22.099	0.869	0.043	NA	NA	NA

Test Agent	Treatment	Concentration	Phase	# viable animals	# total animals	% viable animals	More than 70% viable	interval	AUC	Pval	RelativeRatio	Significance p<0.01	activity	Sig
MWCNTs	Chorion	75	Dark	45	48	0.94	TRUE	Dark	20.493	0.164	0.158	NA	NA	NA
MWCNTs	Chorion	100	Dark	47	48	0.98	TRUE	Dark	19.763	0.512	0.081	NA	NA	NA
MWCNTs	Chorion	0	Light	43	48	0.9	TRUE	Light	-37.789	1	0	NA	NA	NA
MWCNTs	Chorion	10	Light	48	48	1	TRUE	Light	2.031	0.999	-0.381	NA	NA	NA
MWCNTs	Chorion	23.2	Light	43	48	0.9	TRUE	Light	13.692	0.614	-0.711	NA	NA	NA
MWCNTs	Chorion	50	Light	47	48	0.98	TRUE	Light	15.228	0	-0.675	YES	HYPO	YES
MWCNTs	Chorion	75	Light	45	48	0.94	TRUE	Light	14.112	0.286	0.317	NA	NA	NA
MWCNTs	Chorion	100	Light	47	48	0.98	TRUE	Light	14.95	0.941	0.071	NA	NA	NA
MWCNTs	DR	0	Dark	43	48	0.9	TRUE	Dark	52.836	1	0	NA	NA	NA
MWCNTs	DR	10	Dark	44	48	0.92	TRUE	Dark	60.008	0.872	0.088	NA	NA	NA
MWCNTs	DR	23.2	Dark	40	48	0.83	TRUE	Dark	60.555	0	0.002	YES	NA	NA
MWCNTs	DR	50	Dark	35	48	0.73	TRUE	Dark	62.683	0.015	-0.065	NA	NA	NA
MWCNTs	DR	75	Dark	31	48	0.65	FALSE	Dark	63.194	0	0.018	NA	NA	NA
MWCNTs	DR	100	Dark	26	48	0.54	FALSE	Dark	NA	NA	NA	NA	NA	NA
MWCNTs	DR	0	Light	43	48	0.9	TRUE	Light	11.513	1	0	NA	NA	NA
MWCNTs	DR	10	Light	44	48	0.92	TRUE	Light	45.869	0.013	1.774	NA	NA	NA
MWCNTs	DR	23.2	Light	40	48	0.83	TRUE	Light	53.801	0.614	1.517	NA	NA	NA
MWCNTs	DR	50	Light	35	48	0.73	TRUE	Light	59.062	0	3.352	YES	HYPER	YES
MWCNTs	DR	75	Light	31	48	0.65	FALSE	Light	59.561	0.048	2.004	NA	NA	NA
MWCNTs	DR	100	Light	26	48	0.54	FALSE	Light	NA	NA	NA	NA	NA	NA
MWCNTs	LD	0	Dark	46	48	0.96	TRUE	Dark	44.344	1	0	NA	NA	NA
MWCNTs	LD	10	Dark	42	48	0.88	TRUE	Dark	42.048	0.036	-0.001	NA	NA	NA
MWCNTs	LD	23.2	Dark	43	48	0.9	TRUE	Dark	40.787	0	0.279	YES	HYPER	YES
MWCNTs	LD	50	Dark	40	48	0.83	TRUE	Dark	43.699	0	0.091	YES	NA	NA
MWCNTs	LD	75	Dark	39	48	0.81	TRUE	Dark	41.64	0	0.182	YES	HYPER	YES
MWCNTs	LD	100	Dark	38	48	0.79	TRUE	Dark	39.745	0.001	0.112	YES	HYPER	YES
MWCNTs	LD	0	Light	46	48	0.96	TRUE	Light	22.032	1	0	NA	NA	NA
MWCNTs	LD	10	Light	42	48	0.88	TRUE	Light	33.871	0.048	-0.413	NA	NA	NA
MWCNTs	LD	23.2	Light	43	48	0.9	TRUE	Light	31.895	0.048	-1.396	NA	NA	NA
MWCNTs	LD	50	Light	40	48	0.83	TRUE	Light	39.558	0	0.461	YES	HYPER	YES
MWCNTs	LD	75	Light	39	48	0.81	TRUE	Light	37.513	0.379	-1.544	NA	NA	NA
MWCNTs	LD	100	Light	38	48	0.79	TRUE	Light	36.618	0.74	-1.618	NA	NA	NA
MWCNTs	Standard	0	Dark	44	48	0.92	TRUE	Dark	44.815	1	0	NA	NA	NA
MWCNTs	Standard	10	Dark	47	48	0.98	TRUE	Dark	47.659	0.001	0.273	YES	HYPER	YES
MWCNTs	Standard	23.2	Dark	40	48	0.83	TRUE	Dark	44.277	0.227	0.028	NA	NA	NA
MWCNTs	Standard	50	Dark	44	48	0.92	TRUE	Dark	44.551	0	0.095	YES	NA	NA
MWCNTs	Standard	75	Dark	31	48	0.65	FALSE	Dark	49.502	0.002	0.232	NA	NA	NA
MWCNTs	Standard	100	Dark	28	48	0.58	FALSE	Dark	NA	NA	NA	NA	NA	NA
MWCNTs	Standard	0	Light	44	48	0.92	TRUE	Light	-2.557	1	0	NA	NA	NA
MWCNTs	Standard	10	Light	47	48	0.98	TRUE	Light	24.438	0.74	5.381	NA	NA	NA
MWCNTs	Standard	23.2	Light	40	48	0.83	TRUE	Light	34.173	0.105	6.734	NA	NA	NA
MWCNTs	Standard	50	Light	44	48	0.92	TRUE	Light	36.495	0.21	2.988	NA	NA	NA
MWCNTs	Standard	75	Light	31	48	0.65	FALSE	Light	42.381	0.002	-10.785	NA	NA	NA
MWCNTs	Standard	100	Light	28	48	0.58	FALSE	Light	NA	NA	NA	NA	NA	NA
Napthalene	Chorion	0	Dark	33	36	0.92	TRUE	Dark	32.972	1	0	NA	NA	NA
Napthalene	Chorion	1	Dark	33	36	0.92	TRUE	Dark	19.94	0.015	-0.15	NA	NA	NA
Napthalene	Chorion	2.54	Dark	34	36	0.94	TRUE	Dark	17.184	0.015	-0.115	NA	NA	NA
Napthalene	Chorion	6.45	Dark	35	36	0.97	TRUE	Dark	21.834	0.055	-0.038	NA	NA	NA
Napthalene	Chorion	16.4	Dark	33	36	0.92	TRUE	Dark	23.752	0.023	0.492	NA	NA	NA
Napthalene	Chorion	35	Dark	33	36	0.92	TRUE	Dark	26.247	0.009	0.157	YES	HYPER	YES
Napthalene	Chorion	74.8	Dark	33	36	0.92	TRUE	Dark	27.622	0.009	0.26	YES	HYPER	YES
Napthalene	Chorion	100	Dark	35	36	0.97	TRUE	Dark	23.132	0.023	-0.97	NA	NA	NA
Napthalene	Chorion	0	Light	33	36	0.92	TRUE	Light	20.175	1	0	NA	NA	NA
Napthalene	Chorion	1	Light	33	36	0.92	TRUE	Light	13.62	0.941	-2.243	NA	NA	NA
Napthalene	Chorion	2.54	Light	34	36	0.94	TRUE	Light	12.52	0.379	-1.851	NA	NA	NA
Napthalene	Chorion	6.45	Light	35	36	0.97	TRUE	Light	18.66	0.013	0.364	NA	NA	NA
Napthalene	Chorion	16.4	Light	33	36	0.92	TRUE	Light	18.803	0.856	-1.27	NA	NA	NA
Napthalene	Chorion	35	Light	33	36	0.92	TRUE	Light	23.819	0.941	0.214	NA	NA	NA
Napthalene	Chorion	74.8	Light	33	36	0.92	TRUE	Light	25.312	0	-0.301	YES	HYPO	YES
Napthalene	Chorion	100	Light	35	36	0.97	TRUE	Light	24.583	0.005	-1.89	YES	HYPO	YES
Napthalene	DR	0	Dark	34	36	0.94	TRUE	Dark	39.323	1	0	NA	NA	NA
Napthalene	DR	1	Dark	33	36	0.92	TRUE	Dark	33.157	0.305	0.147	NA	NA	NA
Napthalene	DR	2.54	Dark	32	36	0.89	TRUE	Dark	31.925	0.036	0.004	NA	NA	NA
Napthalene	DR	6.45	Dark	32	36	0.89	TRUE	Dark	33.494	0.009	0.056	YES	NA	NA
Napthalene	DR	16.4	Dark	33	36	0.92	TRUE	Dark	34.343	0.001	0.327	YES	HYPER	YES

Test Agent	Treatment	Concentration	Phase	# viable animals	# total animals	% viable animals	More than 70% viable	interval	AUC	Pval	RelativeRatio	Significance p<0.01	activity	Sig
Napthalene	DR	35	Dark	33	36	0.92	TRUE	Dark	34.998	0.226	-0.156	NA	NA	NA
Napthalene	DR	74.8	Dark	33	36	0.92	TRUE	Dark	35.213	0.001	-0.106	YES	HYPO	YES
Napthalene	DR	100	Dark	35	36	0.97	TRUE	Dark	31.543	0.635	-0.237	NA	NA	NA
Napthalene	DR	0	Light	34	36	0.94	TRUE	Light	31.013	1	0	NA	NA	NA
Napthalene	DR	1	Light	33	36	0.92	TRUE	Light	25.994	0.013	-1.021	NA	NA	NA
Napthalene	DR	2.54	Light	32	36	0.89	TRUE	Light	28.747	0.856	-0.642	NA	NA	NA
Napthalene	DR	6.45	Light	32	36	0.89	TRUE	Light	31.094	0.49	-0.158	NA	NA	NA
Napthalene	DR	16.4	Light	33	36	0.92	TRUE	Light	31.136	0.49	-0.604	NA	NA	NA
Napthalene	DR	35	Light	33	36	0.92	TRUE	Light	34.526	0	0.172	YES	HYPER	YES
Napthalene	DR	74.8	Light	33	36	0.92	TRUE	Light	34.645	0.286	-0.019	NA	NA	NA
Napthalene	DR	100	Light	35	36	0.97	TRUE	Light	31.223	0	-1.795	YES	HYPO	YES
Napthalene	LD	0	Dark	31	36	0.86	TRUE	Dark	44.649	1	0	NA	NA	NA
Napthalene	LD	1	Dark	35	36	0.97	TRUE	Dark	40.141	0.002	0.032	YES	NA	NA
Napthalene	LD	2.54	Dark	35	36	0.97	TRUE	Dark	40.425	0.009	0.266	YES	HYPER	YES
Napthalene	LD	6.45	Dark	30	36	0.83	TRUE	Dark	37.656	0.306	0.012	NA	NA	NA
Napthalene	LD	16.4	Dark	31	36	0.86	TRUE	Dark	39.069	0.003	0.08	YES	NA	NA
Napthalene	LD	35	Dark	30	36	0.83	TRUE	Dark	40.532	0.227	0.102	NA	NA	NA
Napthalene	LD	74.8	Dark	33	36	0.92	TRUE	Dark	40.243	0.306	-0.05	NA	NA	NA
Napthalene	LD	100	Dark	35	36	0.97	TRUE	Dark	38.978	0	-0.138	YES	HYPO	YES
Napthalene	LD	0	Light	31	36	0.86	TRUE	Light	26.501	1	0	NA	NA	NA
Napthalene	LD	1	Light	35	36	0.97	TRUE	Light	31.914	0.001	-0.757	YES	HYPO	YES
Napthalene	LD	2.54	Light	35	36	0.97	TRUE	Light	32.605	0	-0.907	YES	HYPO	YES
Napthalene	LD	6.45	Light	30	36	0.83	TRUE	Light	33.953	0.986	-1.184	NA	NA	NA
Napthalene	LD	16.4	Light	31	36	0.86	TRUE	Light	35.876	0.001	-0.183	YES	HYPO	YES
Napthalene	LD	35	Light	30	36	0.83	TRUE	Light	37.924	0	-0.001	YES	NA	NA
Napthalene	LD	74.8	Light	33	36	0.92	TRUE	Light	38.75	0	-0.345	YES	HYPO	YES
Napthalene	LD	100	Light	35	36	0.97	TRUE	Light	37.966	0.49	-0.77	NA	NA	NA
Napthalene	Standard	0	Dark	29	36	0.81	TRUE	Dark	19.19	1	0	NA	NA	NA
Napthalene	Standard	1	Dark	30	36	0.83	TRUE	Dark	35.986	0.081	-0.074	NA	NA	NA
Napthalene	Standard	2.54	Dark	28	36	0.78	TRUE	Dark	36.203	0.005	-0.083	YES	NA	NA
Napthalene	Standard	6.45	Dark	30	36	0.83	TRUE	Dark	37.66	0.003	0.21	YES	HYPER	YES
Napthalene	Standard	16.4	Dark	26	36	0.72	TRUE	Dark	41.834	0.116	0.177	NA	NA	NA
Napthalene	Standard	35	Dark	28	36	0.78	TRUE	Dark	40.339	0	-0.196	YES	HYPO	YES
Napthalene	Standard	74.8	Dark	32	36	0.89	TRUE	Dark	39.522	0	-0.319	YES	HYPO	YES
Napthalene	Standard	100	Dark	33	36	0.92	TRUE	Dark	37.661	0	0.093	YES	NA	NA
Napthalene	Standard	0	Light	29	36	0.81	TRUE	Light	-34.068	1	0	NA	NA	NA
Napthalene	Standard	1	Light	30	36	0.83	TRUE	Light	25.63	0	-2.13	YES	HYPO	YES
Napthalene	Standard	2.54	Light	28	36	0.78	TRUE	Light	30.153	0.008	-1.2	YES	HYPO	YES
Napthalene	Standard	6.45	Light	30	36	0.83	TRUE	Light	30.514	0	-0.894	YES	HYPO	YES
Napthalene	Standard	16.4	Light	26	36	0.72	TRUE	Light	37.005	0	-1.932	YES	HYPO	YES
Napthalene	Standard	35	Light	28	36	0.78	TRUE	Light	38.709	0	-1.219	YES	HYPO	YES
Napthalene	Standard	74.8	Light	32	36	0.89	TRUE	Light	38.765	0	-1.584	YES	HYPO	YES
Napthalene	Standard	100	Light	33	36	0.92	TRUE	Light	34.892	0	-0.122	YES	HYPO	YES
Permethrin	Chorion	0	Dark	32	36	0.89	TRUE	Dark	23.504	1	0	NA	NA	NA
Permethrin	Chorion	1	Dark	34	36	0.94	TRUE	Dark	42.611	0	1.122	YES	HYPER	YES
Permethrin	Chorion	2.54	Dark	32	36	0.89	TRUE	Dark	43.25	0	0.96	YES	HYPER	YES
Permethrin	Chorion	5	Dark	24	36	0.67	FALSE	Dark	52.843	0	1.454	NA	NA	NA
Permethrin	Chorion	8	Dark	6	36	0.17	FALSE	Dark	NA	NA	NA	NA	NA	NA
Permethrin	Chorion	12	Dark	6	36	0.17	FALSE	Dark	NA	NA	NA	NA	NA	NA
Permethrin	Chorion	16.4	Dark	3	36	0.08	FALSE	Dark	NA	NA	NA	NA	NA	NA
Permethrin	Chorion	35	Dark	0	36	0	FALSE	Dark	NA	NA	NA	NA	NA	NA
Permethrin	Chorion	0	Light	32	36	0.89	TRUE	Light	11.753	1	0	NA	NA	NA
Permethrin	Chorion	1	Light	34	36	0.94	TRUE	Light	31.88	0	3.138	YES	HYPER	YES
Permethrin	Chorion	2.54	Light	32	36	0.89	TRUE	Light	38.081	0	0.698	YES	HYPER	YES
Permethrin	Chorion	5	Light	24	36	0.67	FALSE	Light	48.033	0	5.529	NA	NA	NA
Permethrin	Chorion	8	Light	6	36	0.17	FALSE	Light	NA	NA	NA	NA	NA	NA
Permethrin	Chorion	12	Light	6	36	0.17	FALSE	Light	NA	NA	NA	NA	NA	NA
Permethrin	Chorion	16.4	Light	3	36	0.08	FALSE	Light	NA	NA	NA	NA	NA	NA
Permethrin	Chorion	35	Light	0	36	0	FALSE	Light	NA	NA	NA	NA	NA	NA
Permethrin	DR	0	Dark	31	36	0.86	TRUE	Dark	37.035	1	0	NA	NA	NA
Permethrin	DR	1	Dark	28	36	0.78	TRUE	Dark	51.448	0.402	0.213	NA	NA	NA
Permethrin	DR	2.54	Dark	9	36	0.25	FALSE	Dark	NA	NA	NA	NA	NA	NA
Permethrin	DR	5	Dark	6	36	0.17	FALSE	Dark	NA	NA	NA	NA	NA	NA
Permethrin	DR	8	Dark	1	36	0.03	FALSE	Dark	NA	NA	NA	NA	NA	NA
Permethrin	DR	12	Dark	1	36	0.03	FALSE	Dark	NA	NA	NA	NA	NA	NA

Test Agent	Treatment	Concentration	Phase	# viable animals	# total animals	% viable animals	More than 70% viable	interval	AUC	Pval	RelativeRatio	Significance p<0.01	activity	Sig
Permethrin	DR	16.4	Dark	1	36	0.03	FALSE	Dark	NA	NA	NA	NA	NA	NA
Permethrin	DR	35	Dark	1	36	0.03	FALSE	Dark	NA	NA	NA	NA	NA	NA
Permethrin	DR	0	Light	31	36	0.86	TRUE	Light	12.907	1	0	NA	NA	NA
Permethrin	DR	1	Light	28	36	0.78	TRUE	Light	43.875	0	3.459	YES	HYPER	YES
Permethrin	DR	2.54	Light	9	36	0.25	FALSE	Light	NA	NA	NA	NA	NA	NA
Permethrin	DR	5	Light	6	36	0.17	FALSE	Light	NA	NA	NA	NA	NA	NA
Permethrin	DR	8	Light	1	36	0.03	FALSE	Light	NA	NA	NA	NA	NA	NA
Permethrin	DR	12	Light	1	36	0.03	FALSE	Light	NA	NA	NA	NA	NA	NA
Permethrin	DR	16.4	Light	1	36	0.03	FALSE	Light	NA	NA	NA	NA	NA	NA
Permethrin	DR	35	Light	1	36	0.03	FALSE	Light	NA	NA	NA	NA	NA	NA
Permethrin	LD	0	Dark	32	36	0.89	TRUE	Dark	63.903	1	0	NA	NA	NA
Permethrin	LD	1	Dark	28	36	0.78	TRUE	Dark	54.024	0.402	-0.13	NA	NA	NA
Permethrin	LD	2.54	Dark	26	36	0.72	TRUE	Dark	55.131	0.402	-0.213	NA	NA	NA
Permethrin	LD	5	Dark	27	36	0.75	TRUE	Dark	52.322	0.227	-0.12	NA	NA	NA
Permethrin	LD	8	Dark	25	36	0.69	FALSE	Dark	54.285	0	0.119	NA	NA	NA
Permethrin	LD	12	Dark	17	36	0.47	FALSE	Dark	NA	NA	NA	NA	NA	NA
Permethrin	LD	16.4	Dark	12	36	0.33	FALSE	Dark	NA	NA	NA	NA	NA	NA
Permethrin	LD	35	Dark	0	36	0	FALSE	Dark	NA	NA	NA	NA	NA	NA
Permethrin	LD	0	Light	32	36	0.89	TRUE	Light	61.51	1	0	NA	NA	NA
Permethrin	LD	1	Light	28	36	0.78	TRUE	Light	52.81	0.007	-0.502	YES	HYPO	YES
Permethrin	LD	2.54	Light	26	36	0.72	TRUE	Light	55.717	0.381	0.016	NA	NA	NA
Permethrin	LD	5	Light	27	36	0.75	TRUE	Light	51.461	0.858	-0.521	NA	NA	NA
Permethrin	LD	8	Light	25	36	0.69	FALSE	Light	52.074	0	-0.186	NA	NA	NA
Permethrin	LD	12	Light	17	36	0.47	FALSE	Light	NA	NA	NA	NA	NA	NA
Permethrin	LD	16.4	Light	12	36	0.33	FALSE	Light	NA	NA	NA	NA	NA	NA
Permethrin	LD	35	Light	0	36	0	FALSE	Light	NA	NA	NA	NA	NA	NA
Permethrin	Standard	0	Dark	30	36	0.83	TRUE	Dark	25.377	1	0	NA	NA	NA
Permethrin	Standard	1	Dark	25	36	0.69	FALSE	Dark	48.58	0	0.463	NA	NA	NA
Permethrin	Standard	2.54	Dark	26	36	0.72	TRUE	Dark	38.66	0	0.353	YES	HYPER	YES
Permethrin	Standard	5	Dark	23	36	0.64	FALSE	Dark	36.331	0.006	-0.401	NA	NA	NA
Permethrin	Standard	8	Dark	21	36	0.58	FALSE	Dark	NA	NA	NA	NA	NA	NA
Permethrin	Standard	12	Dark	7	36	0.19	FALSE	Dark	NA	NA	NA	NA	NA	NA
Permethrin	Standard	16.4	Dark	9	36	0.25	FALSE	Dark	NA	NA	NA	NA	NA	NA
Permethrin	Standard	35	Dark	1	36	0.03	FALSE	Dark	NA	NA	NA	NA	NA	NA
Permethrin	Standard	0	Light	30	36	0.83	TRUE	Light	-0.623	1	0	NA	NA	NA
Permethrin	Standard	1	Light	25	36	0.69	FALSE	Light	39.717	0	-110.856	NA	NA	NA
Permethrin	Standard	2.54	Light	26	36	0.72	TRUE	Light	32.489	0.032	50.196	NA	NA	NA
Permethrin	Standard	5	Light	23	36	0.64	FALSE	Light	37.123	0.74	-45.811	NA	NA	NA
Permethrin	Standard	8	Light	21	36	0.58	FALSE	Light	NA	NA	NA	NA	NA	NA
Permethrin	Standard	12	Light	7	36	0.19	FALSE	Light	NA	NA	NA	NA	NA	NA
Permethrin	Standard	16.4	Light	9	36	0.25	FALSE	Light	NA	NA	NA	NA	NA	NA
Permethrin	Standard	35	Light	1	36	0.03	FALSE	Light	NA	NA	NA	NA	NA	NA
Pyrene	Chorion	0	Dark	35	36	0.97	TRUE	Dark	43.395	1	0	NA	NA	NA
Pyrene	Chorion	1	Dark	34	36	0.94	TRUE	Dark	46.059	0.001	0.271	YES	HYPER	YES
Pyrene	Chorion	5	Dark	34	36	0.94	TRUE	Dark	45.278	0.023	0.003	NA	NA	NA
Pyrene	Chorion	16.5	Dark	33	36	0.92	TRUE	Dark	43.186	0	-0.227	YES	HYPO	YES
Pyrene	Chorion	30	Dark	36	36	1	TRUE	Dark	38.102	0	-1.15	YES	HYPO	YES
Pyrene	Chorion	50	Dark	31	36	0.86	TRUE	Dark	40.865	0	-0.609	YES	HYPO	YES
Pyrene	Chorion	65	Dark	13	36	0.36	FALSE	Dark	NA	NA	NA	NA	NA	NA
Pyrene	Chorion	100	Dark	4	36	0.11	FALSE	Dark	NA	NA	NA	NA	NA	NA
Pyrene	Chorion	0	Light	35	36	0.97	TRUE	Light	14.558	1	0	NA	NA	NA
Pyrene	Chorion	1	Light	34	36	0.94	TRUE	Light	30.818	0.21	-0.611	NA	NA	NA
Pyrene	Chorion	5	Light	34	36	0.94	TRUE	Light	39.846	0.105	0.03	NA	NA	NA
Pyrene	Chorion	16.5	Light	33	36	0.92	TRUE	Light	41.382	0.74	0.237	NA	NA	NA
Pyrene	Chorion	30	Light	36	36	1	TRUE	Light	43.538	0	2.184	YES	HYPER	YES
Pyrene	Chorion	50	Light	31	36	0.86	TRUE	Light	42.011	0	4.572	YES	HYPER	YES
Pyrene	Chorion	65	Light	13	36	0.36	FALSE	Light	NA	NA	NA	NA	NA	NA
Pyrene	Chorion	100	Light	4	36	0.11	FALSE	Light	NA	NA	NA	NA	NA	NA
Pyrene	DR	0	Dark	30	36	0.83	TRUE	Dark	49.148	1	0	NA	NA	NA
Pyrene	DR	1	Dark	33	36	0.92	TRUE	Dark	51.189	0	0.17	YES	HYPER	YES
Pyrene	DR	5	Dark	33	36	0.92	TRUE	Dark	42.933	0	-0.759	YES	HYPO	YES
Pyrene	DR	16.5	Dark	3	36	0.08	FALSE	Dark	NA	NA	NA	NA	NA	NA
Pyrene	DR	30	Dark	2	36	0.06	FALSE	Dark	NA	NA	NA	NA	NA	NA
Pyrene	DR	50	Dark	3	36	0.08	FALSE	Dark	NA	NA	NA	NA	NA	NA
Pyrene	DR	65	Dark	2	36	0.06	FALSE	Dark	NA	NA	NA	NA	NA	NA

Test Agent	Treatment	Concentration	Phase	# viable animals	# total animals	% viable animals	More than 70% viable	interval	AUC	Pval	RelativeRatio	Significance p<0.01	activity	Sig
Pyrene	DR	100	Dark	0	36	0	FALSE	Dark	NA	NA	NA	NA	NA	NA
Pyrene	DR	0	Light	30	36	0.83	TRUE	Light	26.658	1	0	NA	NA	NA
Pyrene	DR	1	Light	33	36	0.92	TRUE	Light	40.308	0.072	-0.151	NA	NA	NA
Pyrene	DR	5	Light	33	36	0.92	TRUE	Light	48.072	0	0.336	YES	HYPER	YES
Pyrene	DR	16.5	Light	3	36	0.08	FALSE	Light	NA	NA	NA	NA	NA	NA
Pyrene	DR	30	Light	2	36	0.06	FALSE	Light	NA	NA	NA	NA	NA	NA
Pyrene	DR	50	Light	3	36	0.08	FALSE	Light	NA	NA	NA	NA	NA	NA
Pyrene	DR	65	Light	2	36	0.06	FALSE	Light	NA	NA	NA	NA	NA	NA
Pyrene	DR	100	Light	0	36	0	FALSE	Light	NA	NA	NA	NA	NA	NA
Pyrene	LD	0	Dark	32	36	0.89	TRUE	Dark	47.349	1	0	NA	NA	NA
Pyrene	LD	1	Dark	31	36	0.86	TRUE	Dark	51.385	0.402	0.05	NA	NA	NA
Pyrene	LD	5	Dark	36	36	1	TRUE	Dark	53.396	0.003	0	YES	NA	NA
Pyrene	LD	16.5	Dark	32	36	0.89	TRUE	Dark	43.816	0.009	0.227	YES	HYPER	YES
Pyrene	LD	30	Dark	29	36	0.81	TRUE	Dark	40.53	0.003	-0.304	YES	HYPO	YES
Pyrene	LD	50	Dark	21	36	0.58	FALSE	Dark	NA	NA	NA	NA	NA	NA
Pyrene	LD	65	Dark	9	36	0.25	FALSE	Dark	NA	NA	NA	NA	NA	NA
Pyrene	LD	100	Dark	0	36	0	FALSE	Dark	NA	NA	NA	NA	NA	NA
Pyrene	LD	0	Light	32	36	0.89	TRUE	Light	37.972	1	0	NA	NA	NA
Pyrene	LD	1	Light	31	36	0.86	TRUE	Light	48.657	0	0.35	YES	HYPER	YES
Pyrene	LD	5	Light	36	36	1	TRUE	Light	52.735	0.032	0.531	NA	NA	NA
Pyrene	LD	16.5	Light	32	36	0.89	TRUE	Light	40.131	0.072	-2.039	NA	NA	NA
Pyrene	LD	30	Light	29	36	0.81	TRUE	Light	40.644	0	-0.598	YES	HYPO	YES
Pyrene	LD	50	Light	21	36	0.58	FALSE	Light	NA	NA	NA	NA	NA	NA
Pyrene	LD	65	Light	9	36	0.25	FALSE	Light	NA	NA	NA	NA	NA	NA
Pyrene	LD	100	Light	0	36	0	FALSE	Light	NA	NA	NA	NA	NA	NA
Pyrene	Standard	0	Dark	34	36	0.94	TRUE	Dark	43.378	1	0	NA	NA	NA
Pyrene	Standard	1	Dark	35	36	0.97	TRUE	Dark	43.558	0.001	0.124	YES	HYPER	YES
Pyrene	Standard	5	Dark	35	36	0.97	TRUE	Dark	39.585	0.005	0.083	YES	NA	NA
Pyrene	Standard	16.5	Dark	29	36	0.81	TRUE	Dark	33.09	0	-0.451	YES	HYPO	YES
Pyrene	Standard	30	Dark	31	36	0.86	TRUE	Dark	30.915	0	-0.195	YES	HYPO	YES
Pyrene	Standard	50	Dark	17	36	0.47	FALSE	Dark	NA	NA	NA	NA	NA	NA
Pyrene	Standard	65	Dark	8	36	0.22	FALSE	Dark	NA	NA	NA	NA	NA	NA
Pyrene	Standard	100	Dark	2	36	0.06	FALSE	Dark	NA	NA	NA	NA	NA	NA
Pyrene	Standard	0	Light	34	36	0.94	TRUE	Light	18.348	1	0	NA	NA	NA
Pyrene	Standard	1	Light	35	36	0.97	TRUE	Light	32.45	0.005	-0.423	YES	HYPO	YES
Pyrene	Standard	5	Light	35	36	0.97	TRUE	Light	32.679	0.002	-1.591	YES	HYPO	YES
Pyrene	Standard	16.5	Light	29	36	0.81	TRUE	Light	32.452	0	-1.564	YES	HYPO	YES
Pyrene	Standard	30	Light	31	36	0.86	TRUE	Light	28.235	0.02	-1.578	NA	NA	NA
Pyrene	Standard	50	Light	17	36	0.47	FALSE	Light	NA	NA	NA	NA	NA	NA
Pyrene	Standard	65	Light	8	36	0.22	FALSE	Light	NA	NA	NA	NA	NA	NA
Pyrene	Standard	100	Light	2	36	0.06	FALSE	Light	NA	NA	NA	NA	NA	NA
Retene	Chorion	0	Dark	32	36	0.89	TRUE	Dark	26.38	1	0	NA	NA	NA
Retene	Chorion	1	Dark	34	36	0.94	TRUE	Dark	23.81	0.055	-0.561	NA	NA	NA
Retene	Chorion	5	Dark	34	36	0.94	TRUE	Dark	36.307	0.305	0.715	NA	NA	NA
Retene	Chorion	20	Dark	31	36	0.86	TRUE	Dark	29.779	0	-1.17	YES	HYPO	YES
Retene	Chorion	30	Dark	26	36	0.72	TRUE	Dark	28.292	0	0.785	YES	HYPER	YES
Retene	Chorion	45	Dark	8	36	0.22	FALSE	Dark	NA	NA	NA	NA	NA	NA
Retene	Chorion	65	Dark	4	36	0.11	FALSE	Dark	NA	NA	NA	NA	NA	NA
Retene	Chorion	100	Dark	1	36	0.03	FALSE	Dark	NA	NA	NA	NA	NA	NA
Retene	Chorion	0	Light	32	36	0.89	TRUE	Light	12.63	1	0	NA	NA	NA
Retene	Chorion	1	Light	34	36	0.94	TRUE	Light	25.878	0.02	0.969	NA	NA	NA
Retene	Chorion	5	Light	34	36	0.94	TRUE	Light	29.804	0.151	3.258	NA	NA	NA
Retene	Chorion	20	Light	31	36	0.86	TRUE	Light	35.006	0	1.154	YES	HYPER	YES
Retene	Chorion	30	Light	26	36	0.72	TRUE	Light	23.475	0	-3.134	YES	HYPO	YES
Retene	Chorion	45	Light	8	36	0.22	FALSE	Light	NA	NA	NA	NA	NA	NA
Retene	Chorion	65	Light	4	36	0.11	FALSE	Light	NA	NA	NA	NA	NA	NA
Retene	Chorion	100	Light	1	36	0.03	FALSE	Light	NA	NA	NA	NA	NA	NA
Retene	DR	0	Dark	30	36	0.83	TRUE	Dark	9.949	1	0	NA	NA	NA
Retene	DR	1	Dark	23	36	0.64	FALSE	Dark	32.963	0.762	0.272	NA	NA	NA
Retene	DR	5	Dark	26	36	0.72	TRUE	Dark	37.365	0.116	0.523	NA	NA	NA
Retene	DR	20	Dark	0	36	0	FALSE	Dark	NA	NA	NA	NA	NA	NA
Retene	DR	30	Dark	0	36	0	FALSE	Dark	NA	NA	NA	NA	NA	NA
Retene	DR	45	Dark	0	36	0	FALSE	Dark	NA	NA	NA	NA	NA	NA
Retene	DR	65	Dark	0	36	0	FALSE	Dark	NA	NA	NA	NA	NA	NA
Retene	DR	100	Dark	0	36	0	FALSE	Dark	NA	NA	NA	NA	NA	NA

Test Agent	Treatment	Concentration	Phase	# viable animals	# total animals	% viable animals	More than 70% viable	interval	AUC	Pval	RelativeRatio	Significance p<0.01	activity	Sig
Retene	DR	0	Light	30	36	0.83	TRUE	Light	-39.787	1	0	NA	NA	NA
Retene	DR	1	Light	23	36	0.64	FALSE	Light	18.635	0	-1.905	NA	NA	NA
Retene	DR	5	Light	26	36	0.72	TRUE	Light	26.66	0.005	-1.036	YES	HYPO	YES
Retene	DR	20	Light	0	36	0	FALSE	Light	NA	NA	NA	NA	NA	NA
Retene	DR	30	Light	0	36	0	FALSE	Light	NA	NA	NA	NA	NA	NA
Retene	DR	45	Light	0	36	0	FALSE	Light	NA	NA	NA	NA	NA	NA
Retene	DR	65	Light	0	36	0	FALSE	Light	NA	NA	NA	NA	NA	NA
Retene	DR	100	Light	0	36	0	FALSE	Light	NA	NA	NA	NA	NA	NA
Retene	LD	0	Dark	29	36	0.81	TRUE	Dark	56.769	1	0	NA	NA	NA
Retene	LD	1	Dark	28	36	0.78	TRUE	Dark	47.441	0.001	-0.047	YES	NA	NA
Retene	LD	5	Dark	23	36	0.64	FALSE	Dark	54.122	0.515	0.151	NA	NA	NA
Retene	LD	20	Dark	21	36	0.58	FALSE	Dark	NA	NA	NA	NA	NA	NA
Retene	LD	30	Dark	6	36	0.17	FALSE	Dark	NA	NA	NA	NA	NA	NA
Retene	LD	45	Dark	2	36	0.06	FALSE	Dark	NA	NA	NA	NA	NA	NA
Retene	LD	65	Dark	0	36	0	FALSE	Dark	NA	NA	NA	NA	NA	NA
Retene	LD	100	Dark	0	36	0	FALSE	Dark	NA	NA	NA	NA	NA	NA
Retene	LD	0	Light	29	36	0.81	TRUE	Light	60.958	1	0	NA	NA	NA
Retene	LD	1	Light	28	36	0.78	TRUE	Light	46.556	0	-0.571	YES	HYPO	YES
Retene	LD	5	Light	23	36	0.64	FALSE	Light	52.842	0.614	0.221	NA	NA	NA
Retene	LD	20	Light	21	36	0.58	FALSE	Light	NA	NA	NA	NA	NA	NA
Retene	LD	30	Light	6	36	0.17	FALSE	Light	NA	NA	NA	NA	NA	NA
Retene	LD	45	Light	2	36	0.06	FALSE	Light	NA	NA	NA	NA	NA	NA
Retene	LD	65	Light	0	36	0	FALSE	Light	NA	NA	NA	NA	NA	NA
Retene	LD	100	Light	0	36	0	FALSE	Light	NA	NA	NA	NA	NA	NA
Retene	Standard	0	Dark	31	36	0.86	TRUE	Dark	41.785	1	0	NA	NA	NA
Retene	Standard	1	Dark	22	36	0.61	FALSE	Dark	40.038	0.635	-0.241	NA	NA	NA
Retene	Standard	5	Dark	29	36	0.81	TRUE	Dark	45.084	0.015	0.932	NA	NA	NA
Retene	Standard	20	Dark	20	36	0.56	FALSE	Dark	NA	NA	NA	NA	NA	NA
Retene	Standard	30	Dark	5	36	0.14	FALSE	Dark	NA	NA	NA	NA	NA	NA
Retene	Standard	45	Dark	0	36	0	FALSE	Dark	NA	NA	NA	NA	NA	NA
Retene	Standard	65	Dark	0	36	0	FALSE	Dark	NA	NA	NA	NA	NA	NA
Retene	Standard	100	Dark	0	36	0	FALSE	Dark	NA	NA	NA	NA	NA	NA
Retene	Standard	0	Light	31	36	0.86	TRUE	Light	37.346	1	0	NA	NA	NA
Retene	Standard	1	Light	22	36	0.61	FALSE	Light	41.686	0	0.111	NA	NA	NA
Retene	Standard	5	Light	29	36	0.81	TRUE	Light	36.241	0	-0.436	YES	HYPO	YES
Retene	Standard	20	Light	20	36	0.56	FALSE	Light	NA	NA	NA	NA	NA	NA
Retene	Standard	30	Light	5	36	0.14	FALSE	Light	NA	NA	NA	NA	NA	NA
Retene	Standard	45	Light	0	36	0	FALSE	Light	NA	NA	NA	NA	NA	NA
Retene	Standard	65	Light	0	36	0	FALSE	Light	NA	NA	NA	NA	NA	NA
Retene	Standard	100	Light	0	36	0	FALSE	Light	NA	NA	NA	NA	NA	NA

Abamectin

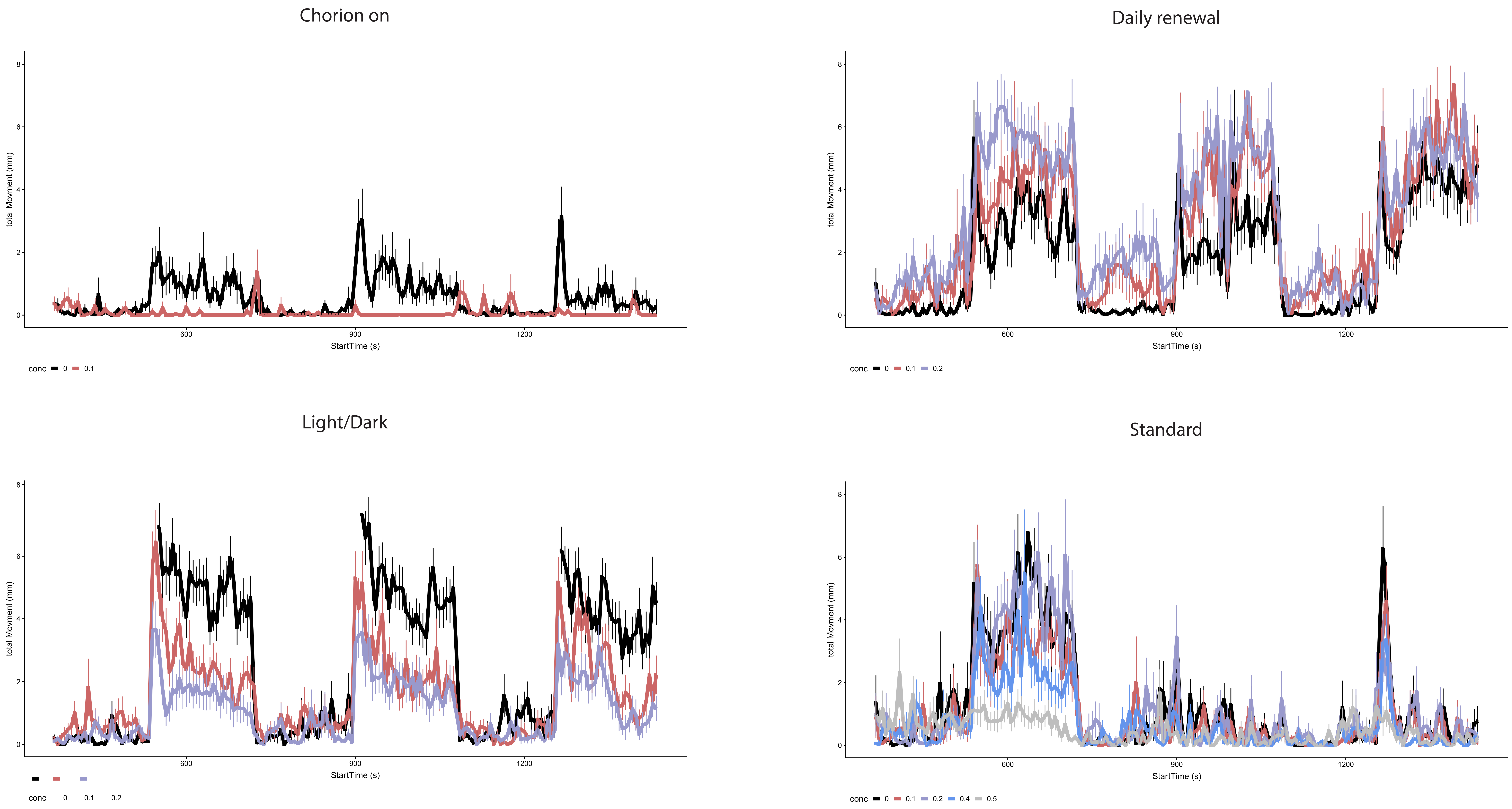

Chorion on
total Movment (mm)
StartTime (s)
conc 0 0.1
Daily renewal
total Movment (mm)
StartTime (s)
conc 0 0.1 0.2
Light/Dark
total Movment (mm)
StartTime (s)
conc 0 0.1 0.2
Standard
total Movment (mm)
StartTime (s)
conc 0 0.1 0.2 0.4 0.5

Chlorpyrifos

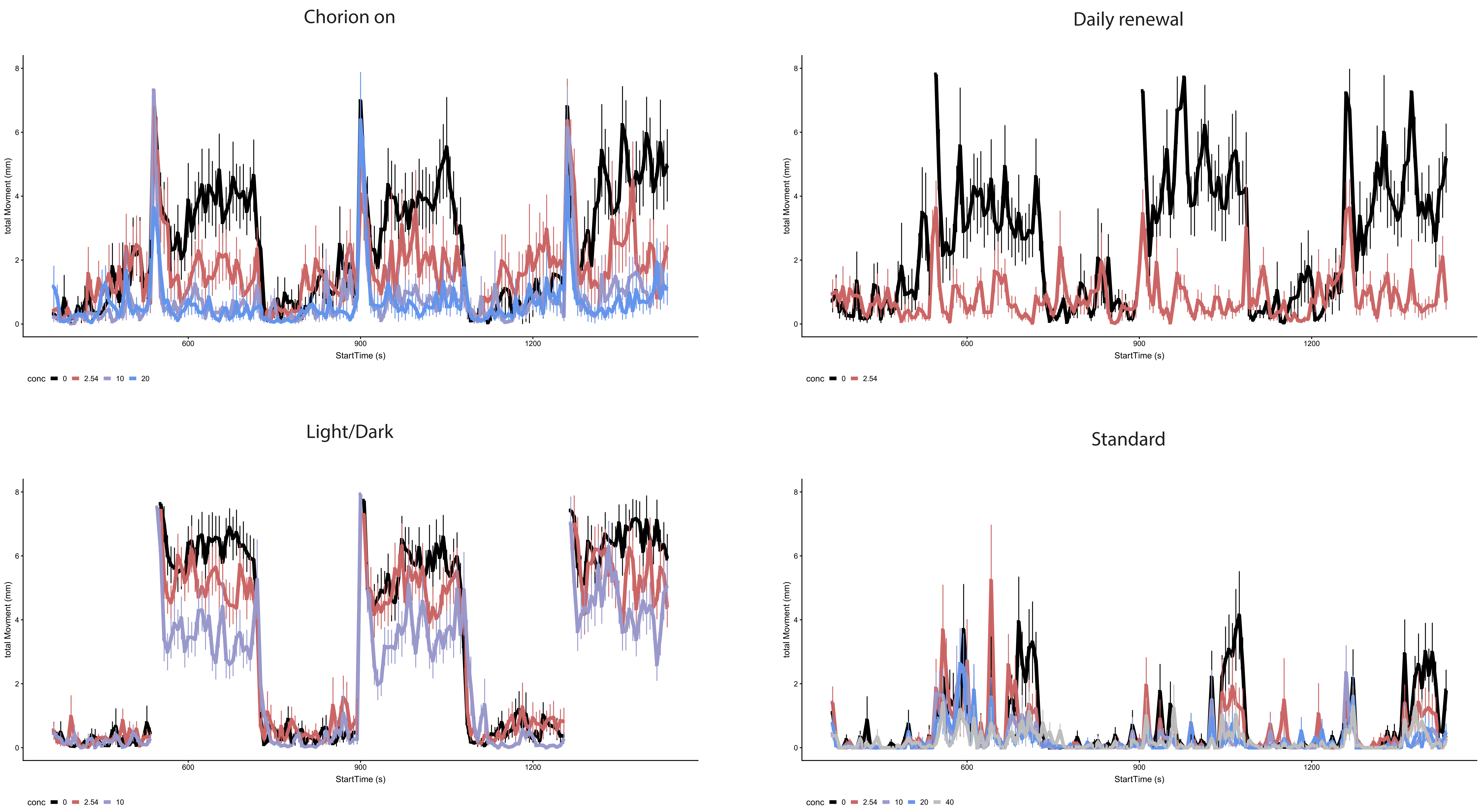

Estradiol

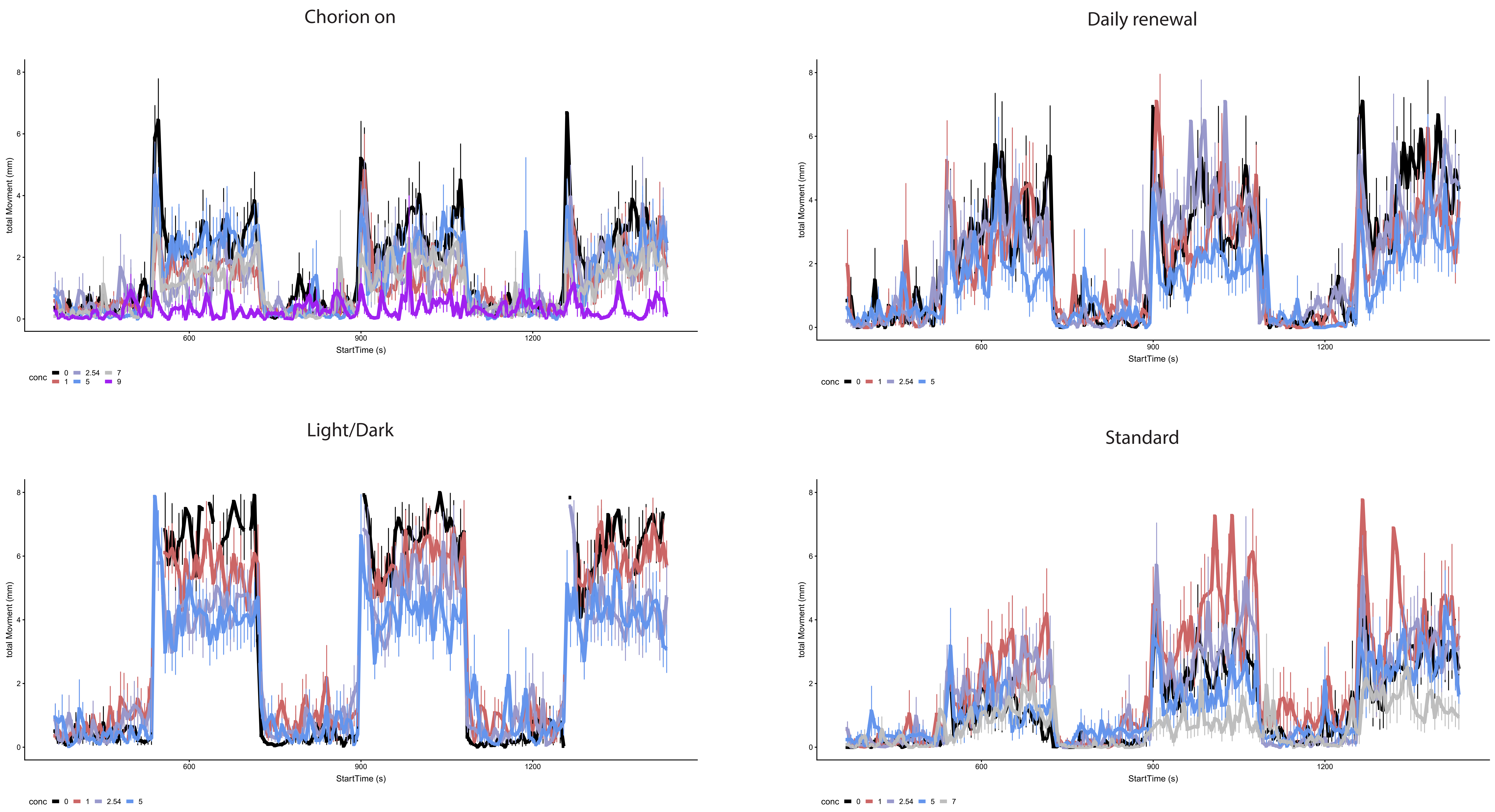

Chorion on
Daily renewal
Light/Dark
Standard
total Movment (mm)
StartTime (s)
0
2
4
6
8
600
900
1200
conc
0
1
2.54
5
7
9

Hydroxyurea

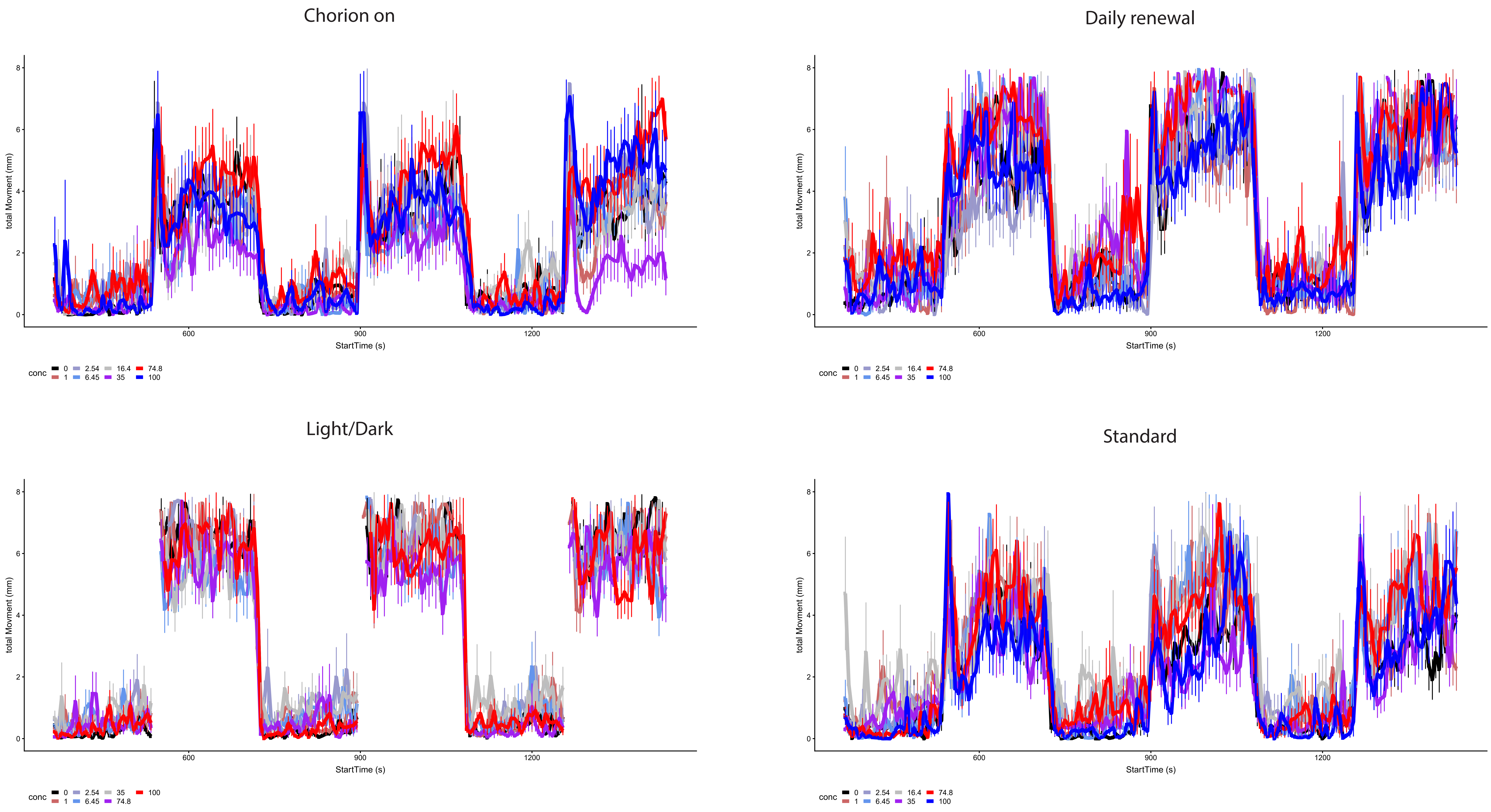

MWCNTs

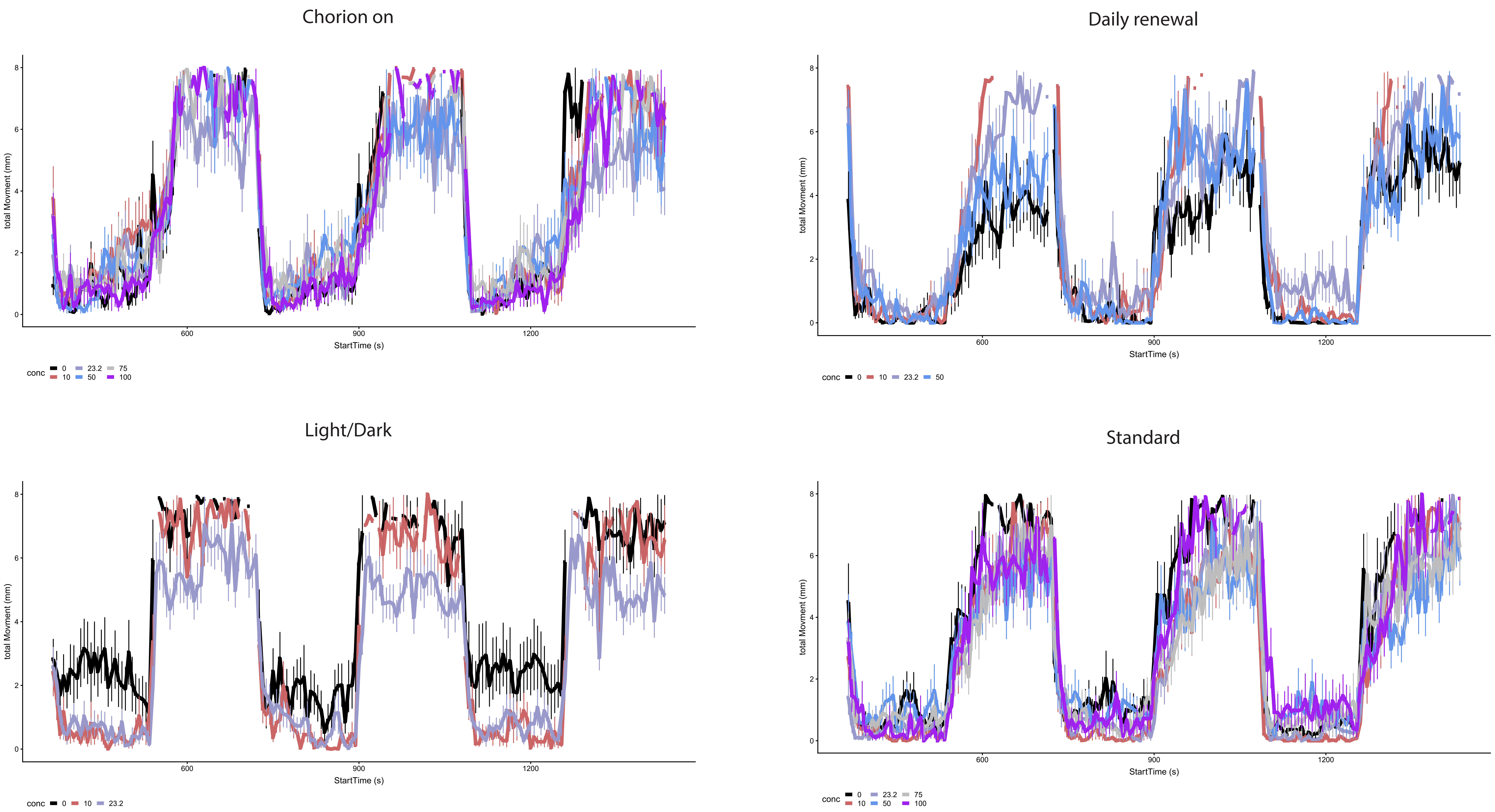

Chorion on
total Movment (mm)
StartTime (s)
conc 0 10 23.2 50 75 100
Daily renewal
total Movment (mm)
StartTime (s)
conc 0 10 23.2 50
Light/Dark
total Movment (mm)
StartTime (s)
conc 0 10 23.2
Standard
total Movment (mm)
StartTime (s)
conc 0 10 23.2 50 75 100

Napthalene

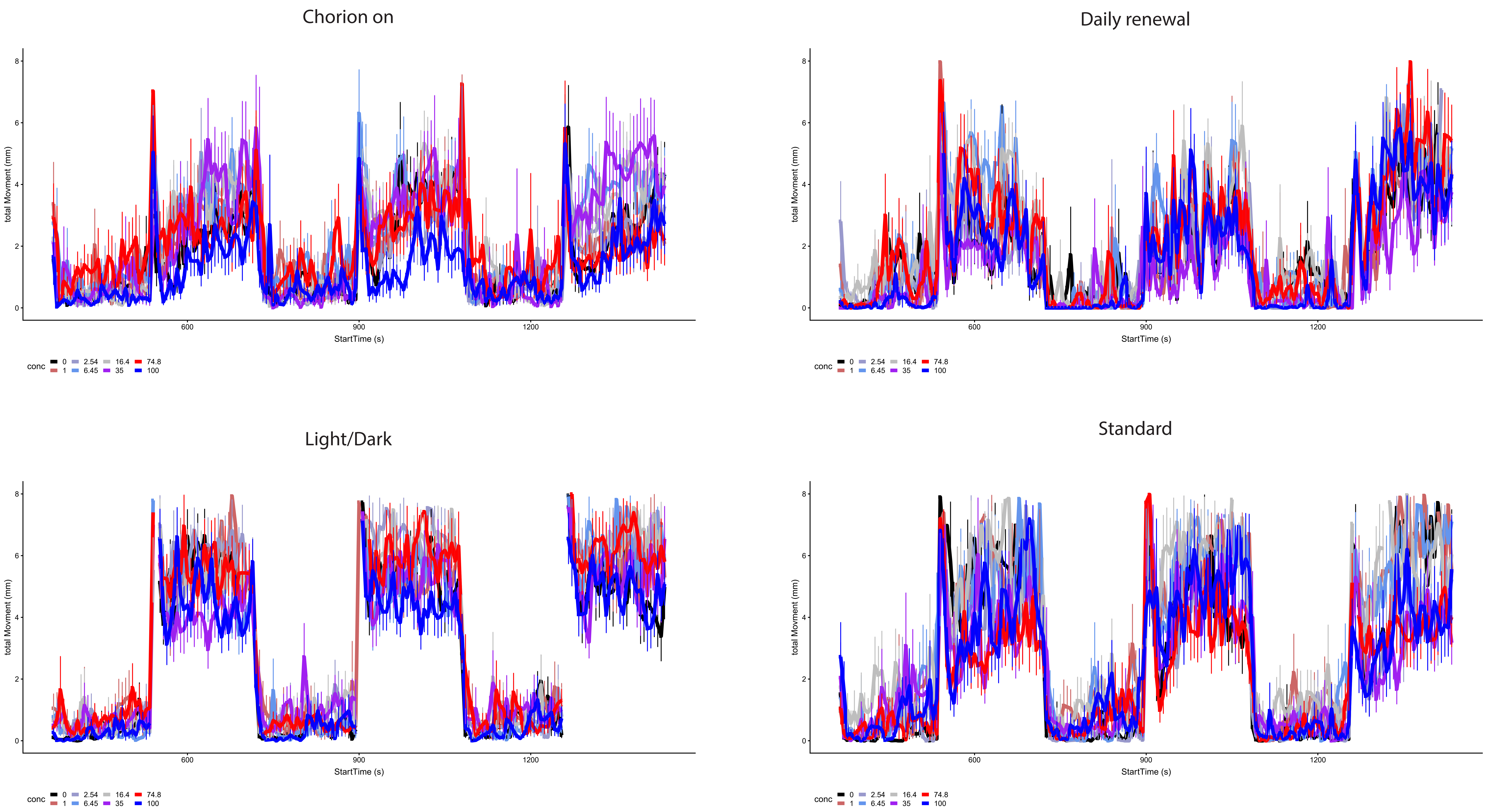

Chorion on
Daily renewal
Light/Dark
Standard
total Movment (mm)
StartTime (s)
0
2
4
6
8
600
900
1200
conc
0
1
2.54
6.45
16.4
35
74.8
100

Permethrin

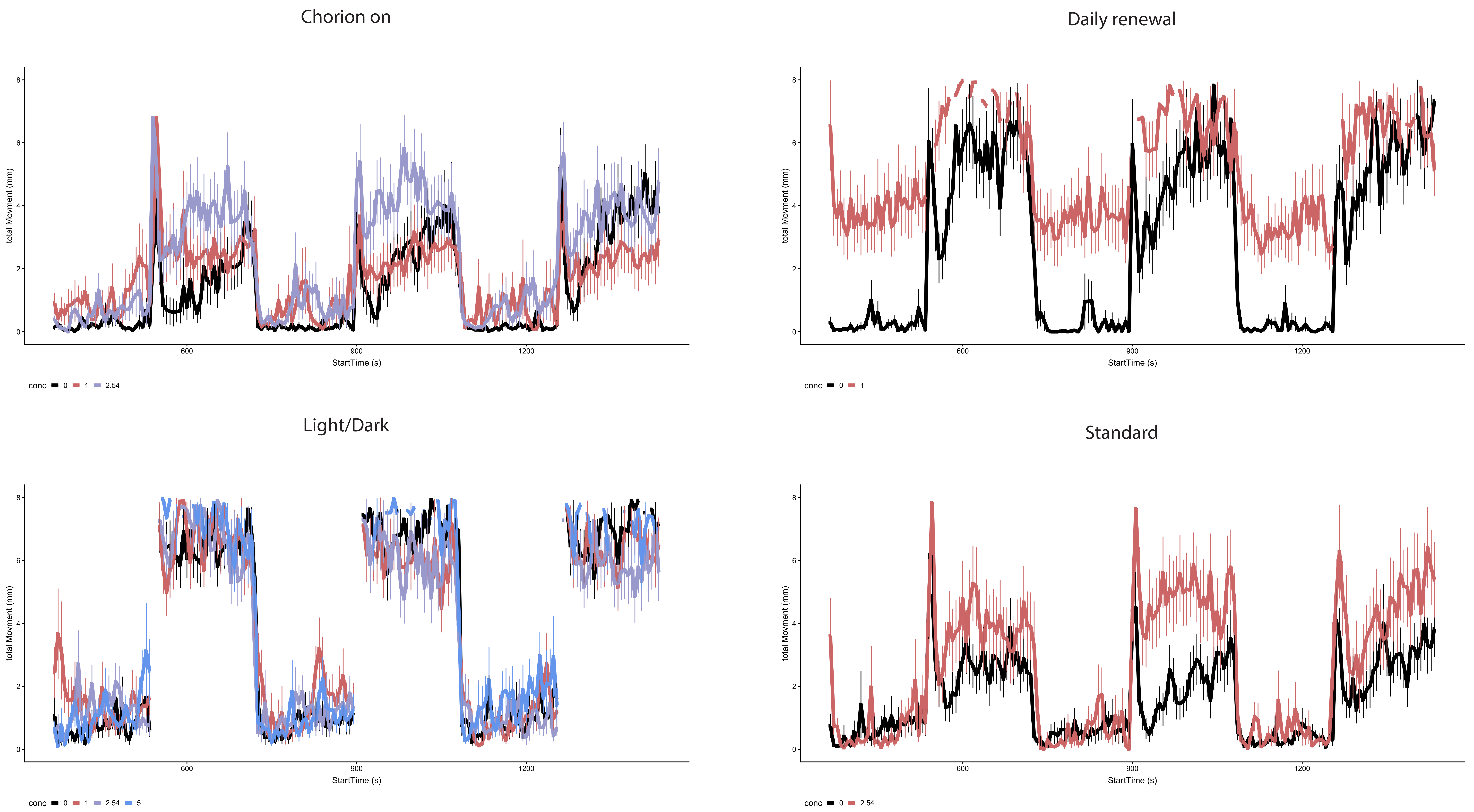

Chorion on
Daily renewal
Light/Dark
Standard
total Movment (mm)
StartTime (s)
0
2
4
6
8
600
900
1200
conc 0 1 2.54
conc 0 1
conc 0 1 2.54 5
conc 0 2.54

Pyrene

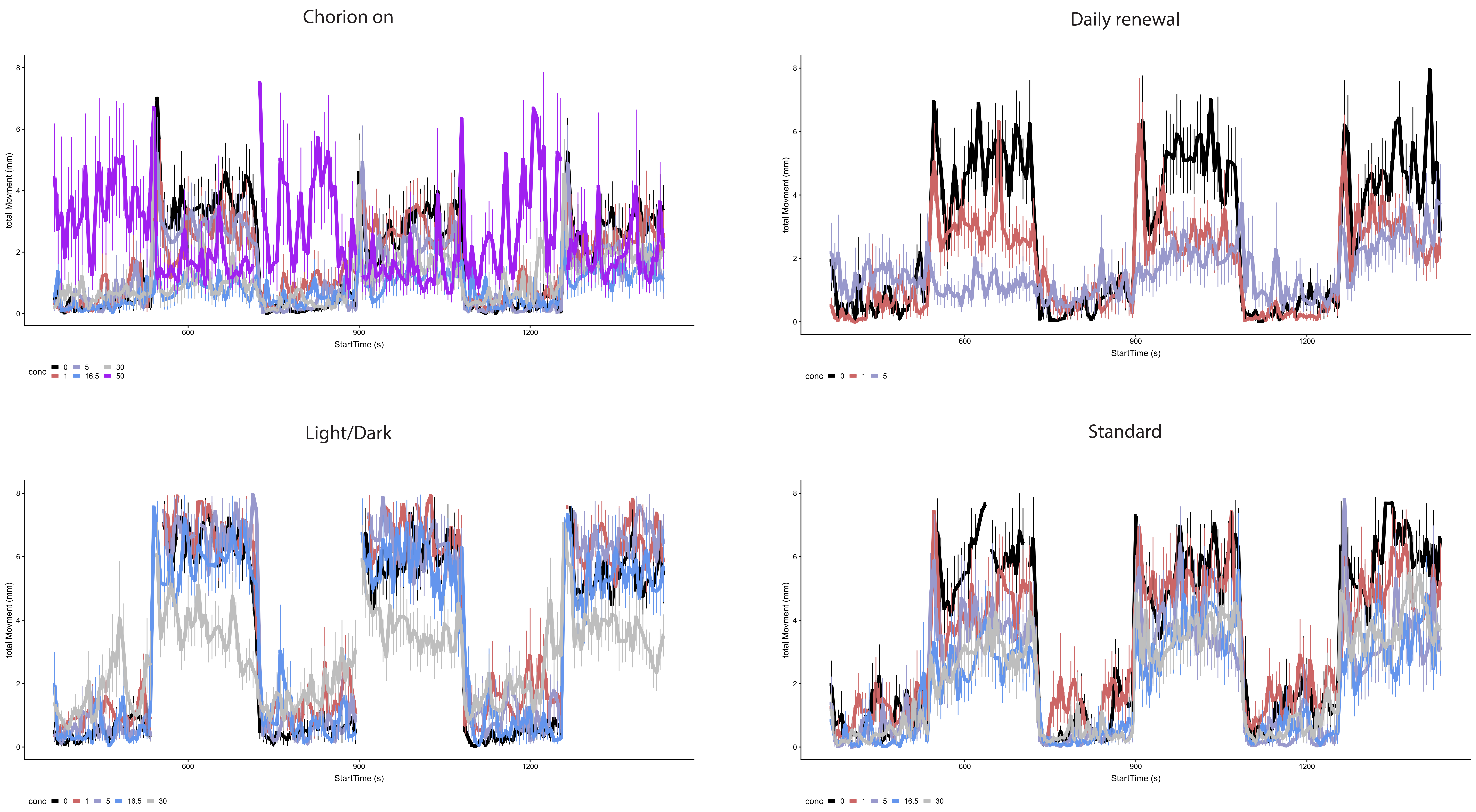
Chorion on
Daily renewal
Light/Dark
Standard
total Movment (mm)
StartTime (s)
0
2
4
6
8
600
900
1200
conc
0
1
5
16.5
30
50

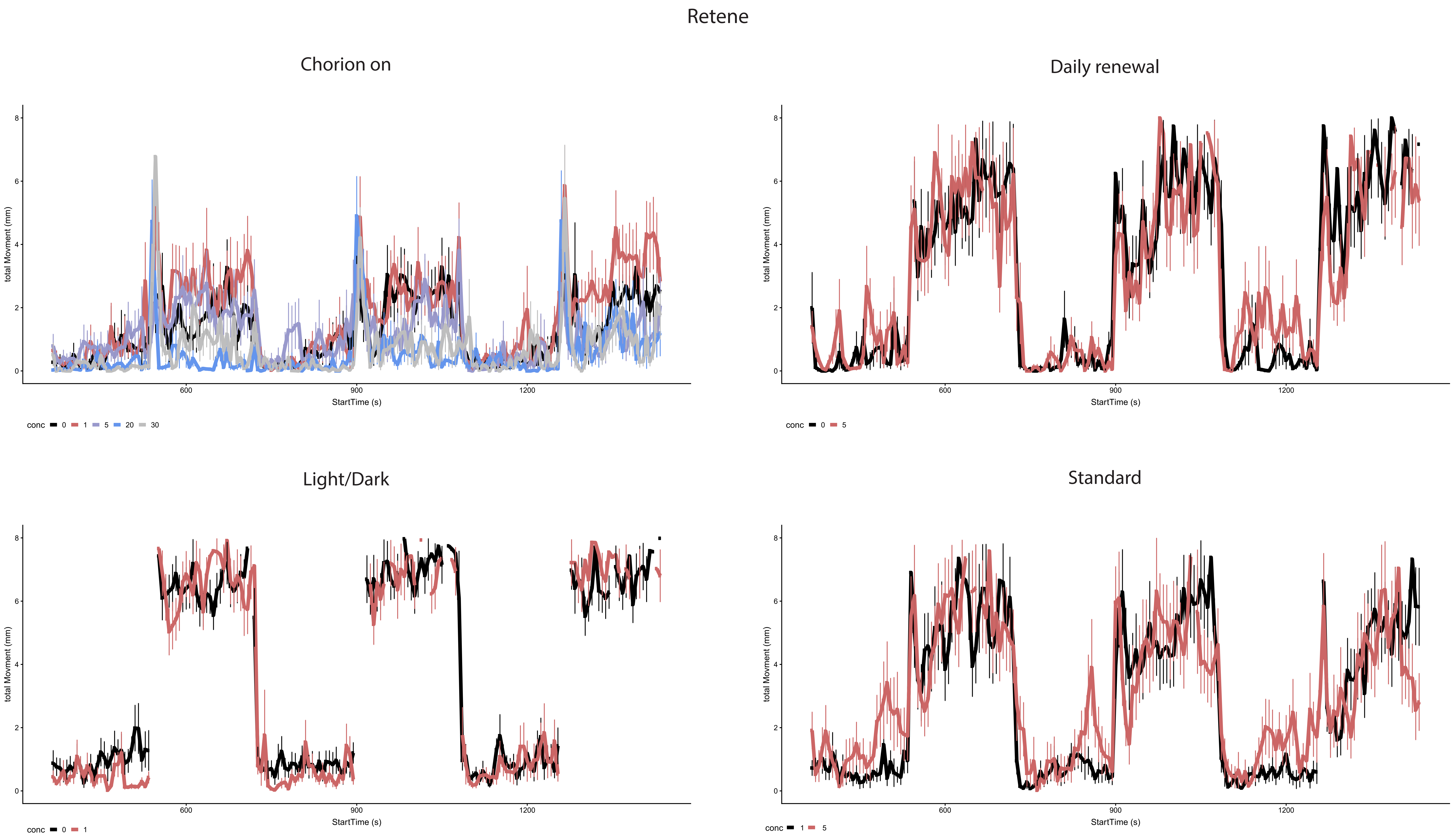

Figure S3. Larval photomotor response (LPR) compiled data. Each panel shows total movement (mm) vs. time. Each panel shows three light-dark cycles. For all chemicals, units are micromolar. For MWCNTs, concentrations are micrograms per milliliter. Data for concentrations resulting in less than 70% normally-developed fish were not analyzed.
